# Highly enantioselective construction of tertiary thioethers and alcohols *via* phosphine-catalyzed asymmetric γ-addition reactions of 5*H*-thiazol-4-ones and 5*H*-oxazol-4-ones: scope and mechanistic understandings†
†Electronic supplementary information (ESI) available: Complete experimental procedures and characterization data for the prepared compounds. See DOI: 10.1039/c5sc01614b



**DOI:** 10.1039/c5sc01614b

**Published:** 2015-06-02

**Authors:** Tianli Wang, Zhaoyuan Yu, Ding Long Hoon, Kuo-Wei Huang, Yu Lan, Yixin Lu

**Affiliations:** a Department of Chemistry , National University of Singapore , 3 Science Drive 3 , Singapore 117543 , Singapore . Email: chmlyx@nus.edu.sg; b School of Chemistry and Chemical Engineering , Chongqing University , Chongqing 400030 , P. R. China . Email: lanyu@cqu.edu.cn; c Division of Physical Sciences and Engineering , KAUST Catalysis Center , King Abdullah University of Science and Technology , Thuwal 23955-6900 , Saudi Arabia

## Abstract

Phosphine-catalyzed highly enantioselective γ-additions of 5*H*-thiazol-4-ones and 5*H*-oxazol-4-ones to allenoates have been developed for the first time. With the employment of amino-acid derived bifunctional phosphines, a wide range of substituted 5*H*-thiazol-4-one and 5*H*-oxazol-4-one derivatives bearing heteroatom (S or O)-containing tertiary chiral centers were constructed in high yields and excellent enantioselectivities. The reported method provides facile access to enantioenriched tertiary thioethers/alcohols. The mechanism of the γ-addition reaction was investigated by performing DFT calculations, and the hydrogen bonding interactions between the Brønsted acid moiety of the phosphine catalysts and the “C=O” unit of the donor molecules were shown to be crucial in asymmetric induction.

## Abstract 2

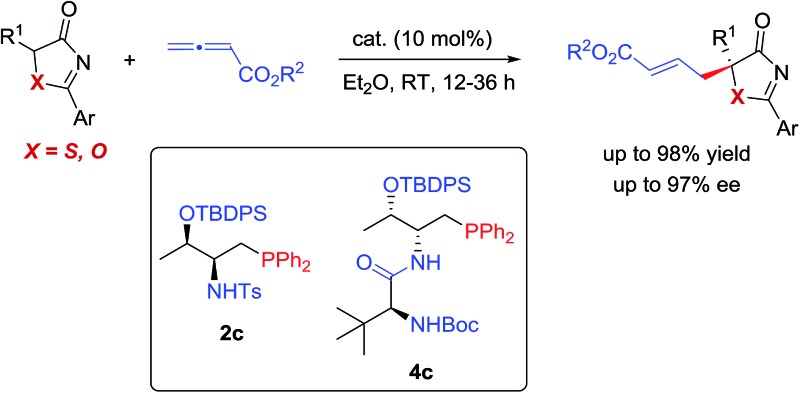
A new method for facile access to enantioenriched tertiary thioethers/alcohols.

## 1. Introduction

**Fig. 1:**
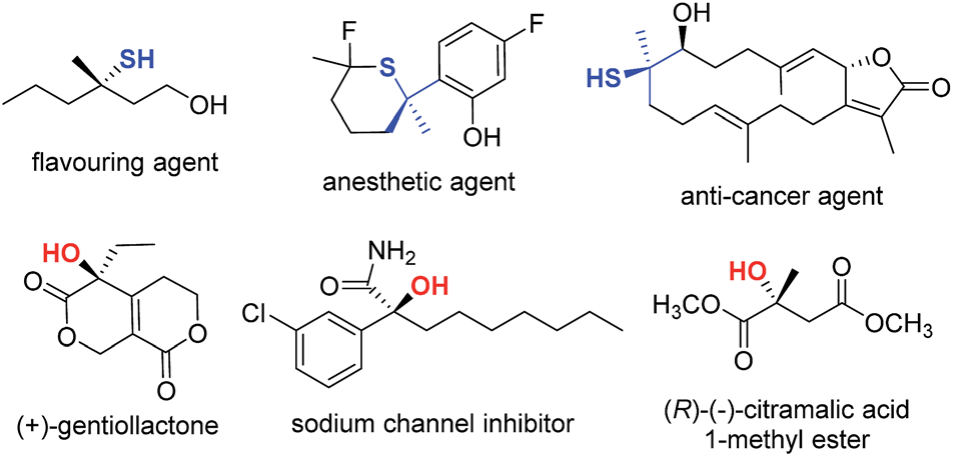
Representative bioactive tertiary thiol(ether)s and alcohols.

Organosulfur compounds are important molecular architectures in synthetic organic chemistry^1^ and chemical biology.^2^ Therefore, it is not surprising that many methods have been devised in recent years to access optically active thiol derivatives. Common approaches include: conjugate addition of thiols,^3^ employment of sulfur-containing pronucleophiles,^4^ kinetic resolution of racemic thiols,^5^ and electrophilic sulfenylation reactions.^6^ In this context, the catalytic synthesis of chiral tertiary thiols is a challenging task and remains largely unexplored.^7^ Analogously, tertiary alcohol-containing structures are of great importance in the biological sciences and the pharmaceutical industry,^8^ and asymmetric synthesis of chiral tertiary alcohols is an intensively investigated area.^9^ A few selected biologically important tertiary thiols and alcohols are illustrated in Fig. 1.^10^ From the outset of this research, we aimed to devise a versatile catalytic approach that would allow us to access both tertiary thiols and alcohols in an enantioselective manner.

To develop a method for the asymmetric synthesis of tertiary thiol molecules, it seems ideal to employ a readily accessible prochiral organosulfur compound. 5*H*-Thiazol-4-one and its derivatives, found to be useful in medicinal chemistry,^11^ are suitable donors; the acidic protons at the 5-position can be readily removed to facilitate their reactions with various electrophiles. Surprisingly, 5*H*-thiazol-4-ones have been rarely used in asymmetric synthesis, and there are only three examples to date describing their applications in asymmetric catalysis. Palomo and co-workers have reported the Brønsted base-catalyzed Michael additions of thiazolones to nitroalkenes^12*a*^ and α′-silyloxy enone,^12*b*^ for the synthesis of tertiary thiols. Very recently, Hartwig disclosed an Ir-catalyzed allylation of 5*H*-thiazol-4-ones to form enantioenriched tertiary thioethers.^12*c*^ We envisioned that careful selection of the electrophilic reaction partners and catalytic systems, in combination with the utilization of pronucleophilic 5*H*-thiazol-4-ones, would lead to the discovery of novel synthetic methods for the asymmetric construction of tertiary thiols. Moreover, given the ready availability of the analogous 5*H*-oxazol-4-ones,^13^ we anticipated that the methodology developed for the thiol synthesis could be easily adapted to include α-oxygenated carboxylate surrogates, thus allowing facile preparation of chiral tertiary alcohols as well.

**Scheme 1:**
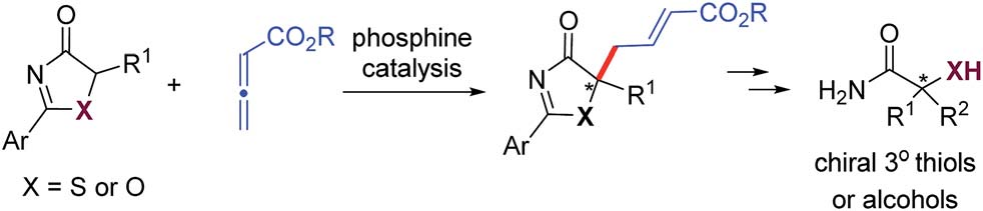
Construction of tertiary thiols/alcohols *via* phosphine-catalyzed γ-additions of 5*H*-thia(oxa)zol-4-ones.

Our group has been actively investigating asymmetric phosphine catalysis^14^ in the past few years. We designed a series of amino acid-based bifunctional phosphine catalysts, and demonstrated their applications in a wide range of asymmetric transformations, including: (aza)-MBH reactions,^15^ [3 + 2], [4 + 2], and [4 + 1] annulations,^16^ allylic alkylations,^17^ and Michael additions.^18^ Very recently, we disclosed the utilization of 2,3-butadienoates in phosphine-catalyzed enantioselective γ-addition reactions.^19,20^ To further expand the range of phosphine-mediated asymmetric reactions, we envisaged that 5*H*-thiazol-4-ones and 5*H*-oxazol-4-ones could serve as valuable donors in phosphine-catalyzed γ-addition reactions to allenoates (Scheme 1). The chiral heteroatom-containing adducts formed have masked functionalities, and can be manipulated easily to give tertiary thiols/thioethers and alcohols.

In this article, we disclose the first utilization of 5*H*-thiazol-4-ones and 5*H*-oxazol-4-ones in phosphine-catalyzed asymmetric γ-addition reactions, and the products can be readily converted to optically enriched tertiary thioethers and alcohols. In addition, we have also carried out DFT calculations to gain insights into the reaction mechanism and understand the origin of the stereochemical outcome of the reaction.

## 2. Results and discussion

### 2.1 Phosphine-catalyzed enantioselective γ-addition of 5*H*-thiazol-4-ones

**Scheme 2:**
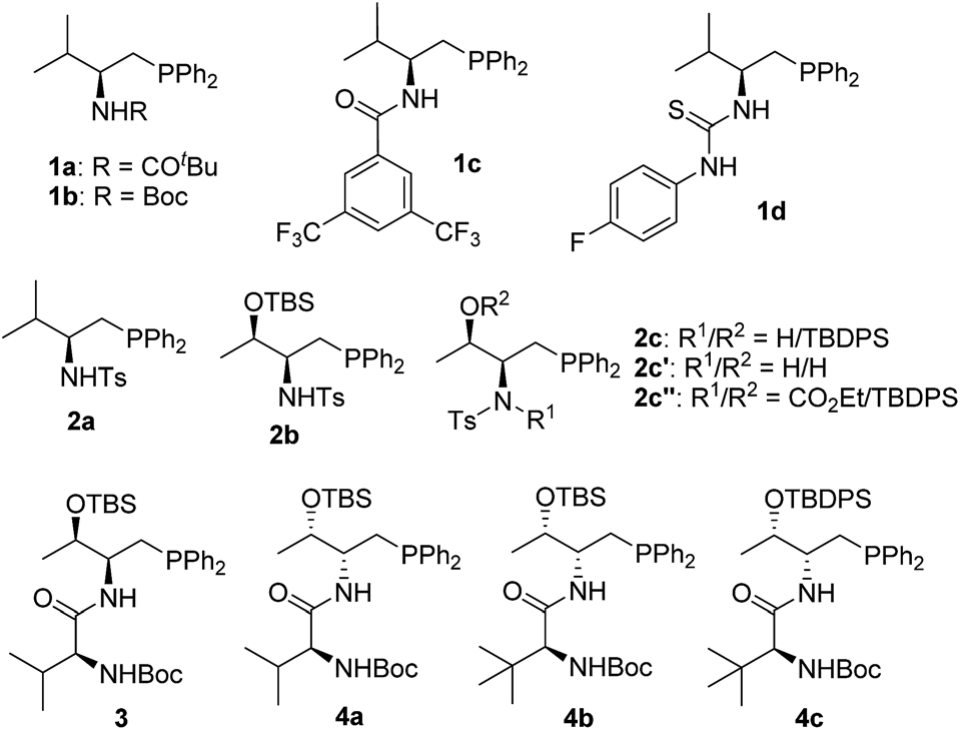
Phosphine catalysts investigated.

In the past few years, amino acid-based bifunctional phosphines have been shown to be very powerful in phosphine catalysis. In this study, readily available l-valine and l-threonine were chosen as the starting chiral skeletons for the preparation of the phosphine catalysts. By installing different hydrogen bond donating groups and introducing various *O*-silyl protecting groups, we prepared a wide range of amino acid-derived bifunctional phosphines (Scheme 2), which were used for subsequent studies.

We began our investigations by choosing 5-methyl-2-phenylthiazol-4(5*H*)-one **5a** and allenoate **6c** as substrates to evaluate the catalytic effects of the phosphine catalysts for the projected γ-addition (Table 1). To our delight, all the bifunctional phosphines could effectively promote the reaction. Among all the l-valine-derived phosphines, sulfonamide–phosphine **2a** was found to be the most efficient (entries 1–5). l-Threonine-derived phosphine sulfonamide catalysts (**2b** & **2c**) were then employed, and the enantioselectivity of the reaction could be improved to 89% ee (entries 6 and 7). The dipeptide phosphines were found to be less effective (entries 8–11).

**Table 1:** Enantioselective γ-addition of 5*H*-thiazol-4-one **5a** to allenoate **6c** catalyzed by different chiral phosphines^*a*^

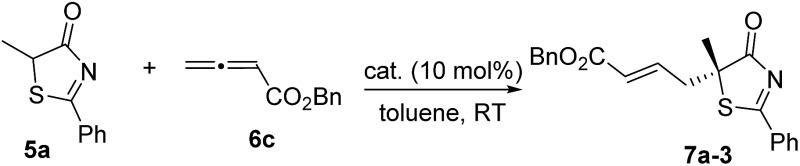				
Entry	Cat.	Time (h)	Yield^*b*^ (%)	ee^*c*^ (%)
1	**1a**	12	86	10
2	**1b**	12	90	62
3	**1c**	12	86	34
4	**1d**	12	92	34
5	**2a**	12	91	66
6	**2b**	12	90	78
7	**2c**	12	95	89
8	**3**	12	88	47
9	**4a**	12	92	–57
10	**4b**	12	88	–65
11	**4c**	12	92	–68

^*a*^Reactions were performed with **5a** (0.1 mmol), **6c** (0.12 mmol) and the catalyst (0.01 mmol) in toluene (1.0 mL) at room temperature.

^*b*^Isolated yield.

^*c*^Determined by HPLC analysis on a chiral stationary phase.

Subsequently, we further optimized the reaction conditions by varying the ester moiety in the allenoate structure (Table 2, entries 1–8). Among all the allenoates examined, the dibenzosuberyl ester proved to be the best, and the ee value of the reaction could be further improved to 91% (entry 6). Furthermore, solvent screening revealed that diethyl ether was the optimal solvent, and the desired product was obtained in 97% yield and with 95% ee under the optimized conditions (Table 2, entries 9–12). The use of different molecular sieves as additives did not result in a further improvement in enantioselectivity (entries 13–15). In addition, lowering the reaction temperature resulted in a significant decrease in reactivity coupled with reduced enantioselectivity (entry 16).

**Table 2:** Optimization of reaction conditions^*a*^

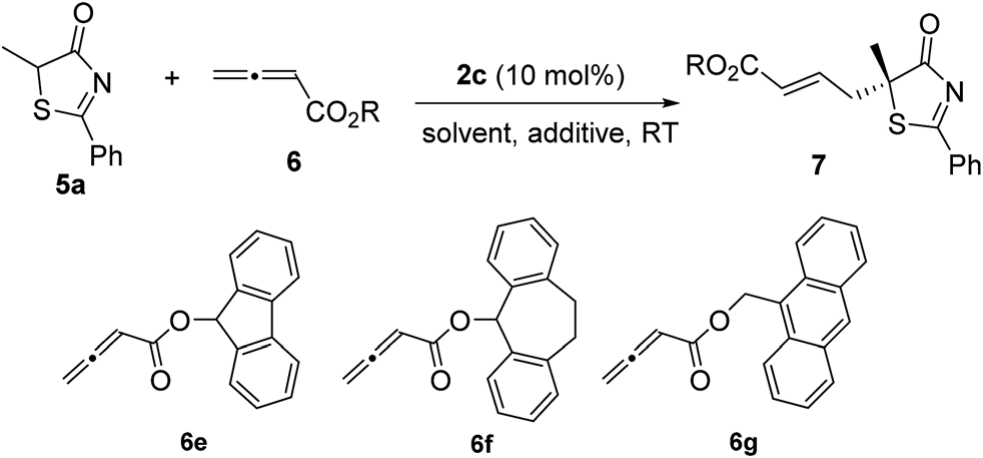				
Entry	R/**6**	Solvent	Yield^*b*^ (%)	ee^*c*^ (%)
1	Et/**6a**	Toluene	95	87
2	^*t*^Bu/**6b**	Toluene	95	88
3	Bn/**6c**	Toluene	95	89
4	CHPh_2_/**6d**	Toluene	86	90
5	**6e**	Toluene	88	70
6	**6f**	Toluene	96	91
7	**6g**	Toluene	93	87
8	Ph/**6h**	Toluene	91	57
9	**6f**	Xylene	95	93
10	**6f**	Et_2_O	97	95
11	**6f**	CHCl_3_	94	83
12^*d*^	**6f**	CH_2_Cl_2_	87	92
13^*e*^	**6f**	Et_2_O	97	94
14^*f*^	**6f**	Et_2_O	97	94
15^*g*^	**6f**	Et_2_O	96	94
16^*h*^	**6f**	Et_2_O	86	90

^*a*^Reactions were performed with **5a** (0.1 mmol), **6** (0.12 mmol) and **2c** (0.01 mmol) in the solvent specified (1.0 mL) at room temperature for 12 h.

^*b*^Isolated yield.

^*c*^Determined by HPLC analysis on a chiral stationary phase.

^*d*^The reaction was stirred for 15 h.

^*e*^3 Å-MS were added.

^*f*^4 Å-MS were added.

^*g*^5 Å-MS were added.

^*h*^The reaction was stirred at 0 °C for 36 h.

Having established the optimal reaction conditions, the substrate scope for the γ-addition of thiazolones to allenoates was then evaluated (Table 3). A wide range of 5-alkyl substituted 5*H*-thiazol-4-ones could be employed, the reaction was insensitive to the length of the alkyl chain, and both linear and branched alkyl groups were well tolerated (entries 1–10). In addition, benzyl and 2-(naphthalen-2-yl)-substituted thiazolones also proved to be suitable substrates (entries 11 and 12).

**Table 3:** Substrate scope for the enantioselective γ-addition of 5*H*-thiazol-4-ones to allenoate **6f** ^*a*^

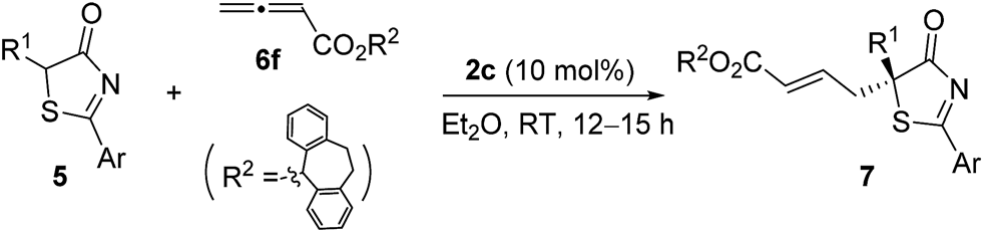				
Entry	Ar/R^1^	Product	Yield^*b*^ (%)	ee^*c*^ (%)
1	Ph/Me	**7a**	97	95
2	Ph/Et	**7b**	95	94
3	Ph/*n*-Pr	**7c**	97	94
4	Ph/*i*-Pr	**7d**	92	92
5	Ph/*n*-Bu	**7e**	94	94
6	Ph/*i*-Bu	**7f**	89	88
7	Ph/*n*-C_6_H_13_	**7g**	96	94
8	Ph/CH(CH_2_)_5_	**7h**	95	93
9	Ph/(CH_2_)_2_SCH_3_	**7i**	93	90
10	Ph/*n*-C_10_H_21_	**7j**	86	93
11	Ph/Bn	**7k**	90	92
12	2-Nap/Me	**7l**	96	89

^*a*^Reactions were performed with **5** (0.1 mmol), **6f** (0.12 mmol) and **2c** (0.01 mmol) in Et_2_O (1.0 mL) at room temperature for 12–15 h.

^*b*^Isolated yield.

^*c*^Determined by HPLC analysis on a chiral stationary phase.

### 2.2. Enantioselective γ-addition of 5*H*-oxazol-4-ones

With the established protocol for the asymmetric γ-addition of 5*H*-thiazol-4-ones in hand, we next targeted access to the analogous α-oxygenated carboxylate surrogates by employing 5*H*-oxazol-4-ones. This task could be challenging as reports containing both sulfur- and oxygen-substituted substrates are rare.^12*c*^ We hypothesized that the high tunability of our amino acid-based phosphine systems may provide a practical solution to this problem. The same set of phosphine catalysts were screened for the γ-addition of 5*H*-oxazol-4-ones to allenoate **6c**, and the results are summarized in Table 4. The best catalyst for the previous addition of 5*H*-thiazol-4-ones, **2c**, only afforded moderate enantioselectivity (entry 1). Switching to the dipeptide phosphines resulted in highly effective catalytic systems. While *O*-TBDPS-l-Thr-l-*tert*-Leu-based **3** led to the desired adduct with a slightly improved ee value, *O*-silyl-d-Thr-l-*tert*-Leu-derived phosphines offered excellent catalytic effects (entries 9–11). Finally, phosphine **4c** was found to be the best catalyst, affording **9a** in 95% yield and 76% ee (entry 11).

**Table 4:** Catalyst screening for the enantioselective γ-addition of 5*H*-oxazol-4-one **8a** to allenoate **6c** ^*a*^

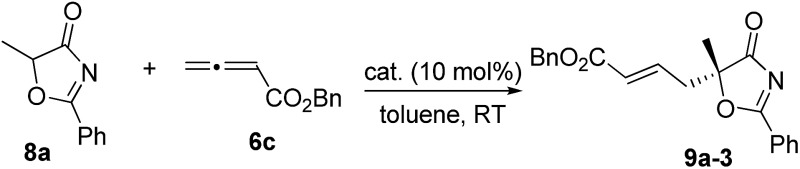				
Entry	Cat.	Time (h)	Yield^*b*^ (%)	ee^*c*^ (%)
1	**2c**	12	89	65
2	**1a**	12	89	60
3	**1b**	12	91	63
4	**1c**	12	92	34
5	**1d**	12	87	12
6	**2a**	12	89	58
7	**2b**	12	88	63
8	**3**	12	93	59
9	**4a**	12	94	–70
10	**4b**	12	94	–73
11	**4c**	12	95	–76

^*a*^Reactions were performed with **8a** (0.1 mmol), **6c** (0.12 mmol) and the catalyst (0.01 mmol) in toluene (1.0 mL) at room temperature for 12 h.

^*b*^Isolated yield.

^*c*^Determined by HPLC analysis on a chiral stationary phase.

To further improve the enantioselectivity, we next optimized the ester moiety of the allenoate (Table 5). Among the different allenoate esters, the dibenzosuberyl ester was most ideal, affording the desired adduct in 96% yield and 86% ee (entry 6). Solvent screening identified diethyl ether as the most suitable solvent for the reaction. When the reaction was performed in the presence of 3 Å molecular sieves in diethyl ether, the γ-addition product was obtained in 97% yield with 92% ee (entry 14).

**Table 5:** Optimization of reaction conditions for γ-addition of 5*H*-oxazol-4-one^*a*^

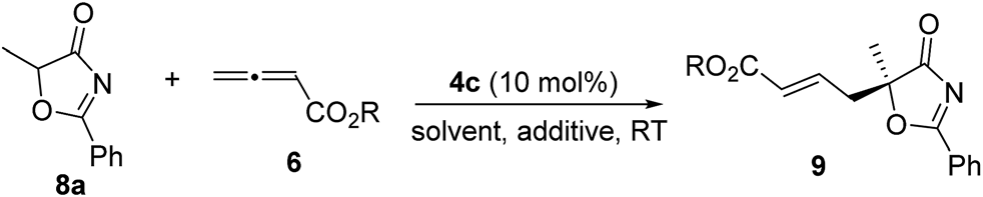				
Entry	Allenoate	Solvent	Yield^*b*^ (%)	ee^*c*^ (%)
1	**6a**	Toluene	93	77
2	**6b**	Toluene	95	82
3	**6c**	Toluene	95	76
4	**6d**	Toluene	96	84
5	**6e**	Toluene	95	84
6	**6f**	Toluene	96	86
7	**6g**	Toluene	85	79
8	**6h**	Toluene	89	67
9	**6f**	Xylene	95	85
10	**6f**	Et_2_O	97	88
11	**6f**	CHCl_3_	92	46
12	**6f**	CH_2_Cl_2_	90	50
13	**6f**	CH_3_CN	82	67
14^*d*^	**6f**	Et_2_O	97	92
15^*e*^	**6f**	Et_2_O	96	91
16^*f*^	**6f**	Et_2_O	96	90
17^*d*^ ^,^ ^*g*^	**6f**	Et_2_O	86	91

^*a*^Reactions were performed with **8a** (0.1 mmol), **6** (0.12 mmol) and **4c** (0.01 mmol) in the solvent specified (1.0 mL) at room temperature overnight.

^*b*^Isolated yield.

^*c*^Determined by HPLC analysis on a chiral stationary phase.

^*d*^3 Å molecular sieves were added.

^*e*^4 Å molecular sieves were added.

^*f*^5 Å molecular sieves were added.

^*g*^The reaction was stirred at 0 °C for 20 h.

Under the optimal reaction conditions, the reaction was applicable to a wide variety of 5-alkyl substituted 5*H*-oxazol-4-ones. As shown in Table 6, the length of the alkyl chain can be varied, and both linear and branched alkyl groups can be employed, and high yields and excellent ee values were attainable in all cases (entries 1–10). When the 5-benzyl substrate was used, the enantioselectivity of the reaction dropped slightly (entry 11), which may be due to the unfavourable aromatic interactions induced by the Bn group. The absolute configuration of the γ-addition products was assigned by comparing the optical rotation of a derivative of **9b** with the value reported in the literature.^21^

**Table 6:** Substrate scope for the enantioselective γ-addition of 5*H*-oxazol-4-ones to allenoates^*a*^

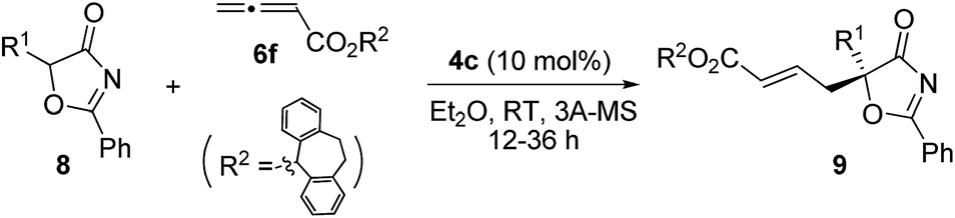				
Entry	R^1^	Time (h)	**9**/Yield^*b*^ (%)	ee^*c*^ (%)
1	Me	12	**9a**/97	92
2	Et	12	**9b**/93	93
3	*n*-Pr	12	**9c**/94	92
4	*i*-Pr	20	**9d**/93	93
5	*n*-Bu	12	**9e**/95	91
6	*i*-Bu	12	**9f**/96	93
7	*t*-Bu	36	**9g**/89	97
8	*n*-C_6_H_13_	20	**9h**/94	93
9	(CH_2_)_2_SCH_3_	12	**9i**/98	94
10	*n*-C_10_H_21_	20	**9j**/91	92
11	Bn	20	**9k**/94	81

^*a*^Reactions were performed with **8** (0.1 mmol), **6f** (0.12 mmol) and **4c** (0.01 mmol) in the solvent specified (1.0 mL) at room temperature overnight.

^*b*^Isolated yield.

^*c*^Determined by HPLC analysis on a chiral stationary phase.

### 2.3. Scope of substrates and synthesis of tertiary thioethers and alcohols

Alkynes are common starting materials in organic synthesis, and the reaction here could be extended to alkyne substrates. Alkynoate (**6′**) could be employed, instead of allenoates, in the γ-addition reactions of both 5*H*-thiazol-4-ones and 5*H*-oxazol-4-ones. Although the reactions were slower, the chemical yields and enantioselectivities were the same [eqn (1) and (2)].
1

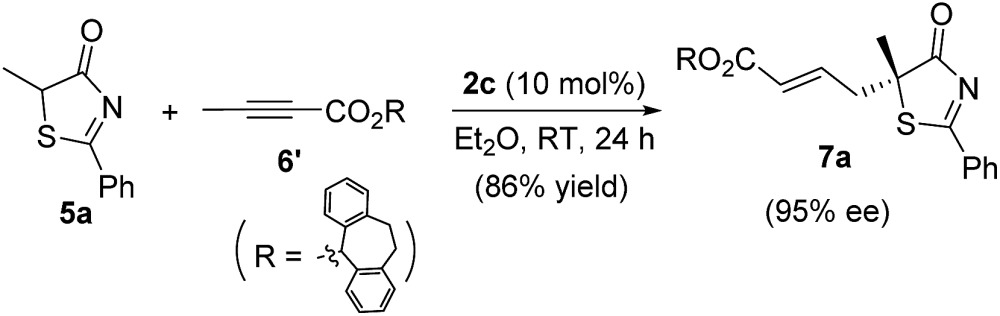



2

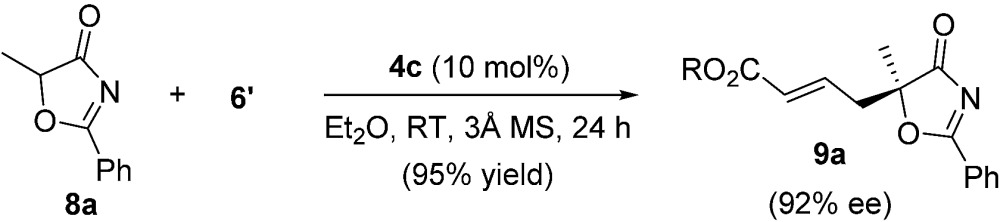




**Scheme 3:**
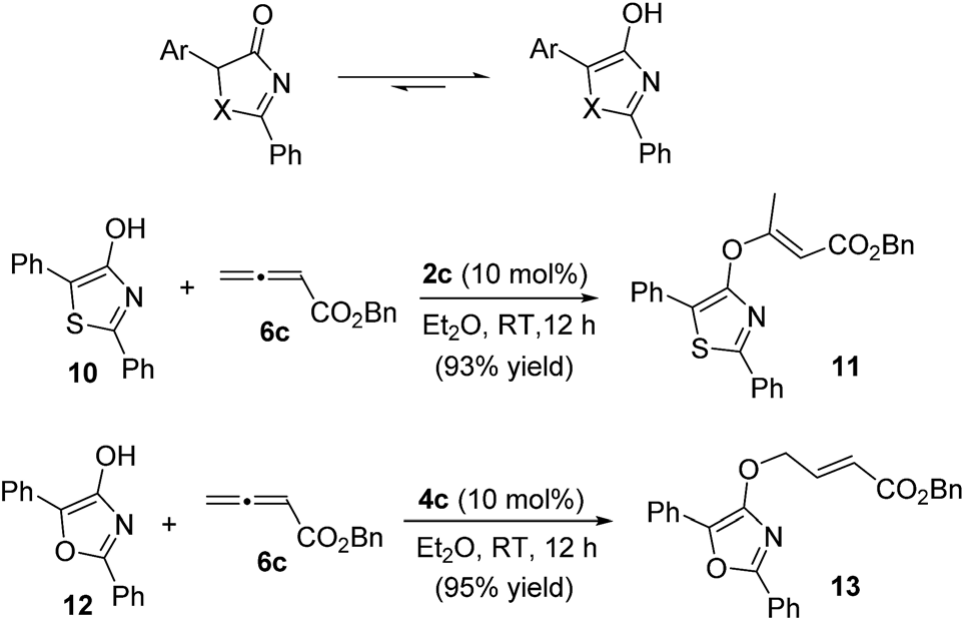
Reactions of 2,5-diphenyl-thiazol-4-ol **10** and 2,5-diphenyl-oxazol-4-ol **12** with allenoate **6c**.

For the thia(oxa)zolone substrates, the inclusion of 5-aryl-substituted thiazol-4-ones and oxazol-4-ones was unsuccessful. Thia(oxa)zolones are known to exist in tautomeric forms as thia(oxa)zoles,^22^ and the presence of a 5-aryl group makes the enol forms far more dominating. Indeed, the γ-addition products were not observed when 5-aryl substituted substrates were used. Instead, *O*-attack of the tautomeric thiazole/oxazole to allenoates took place, and the corresponding achiral adducts were obtained in high yields (Scheme 3).

**Scheme 4:**
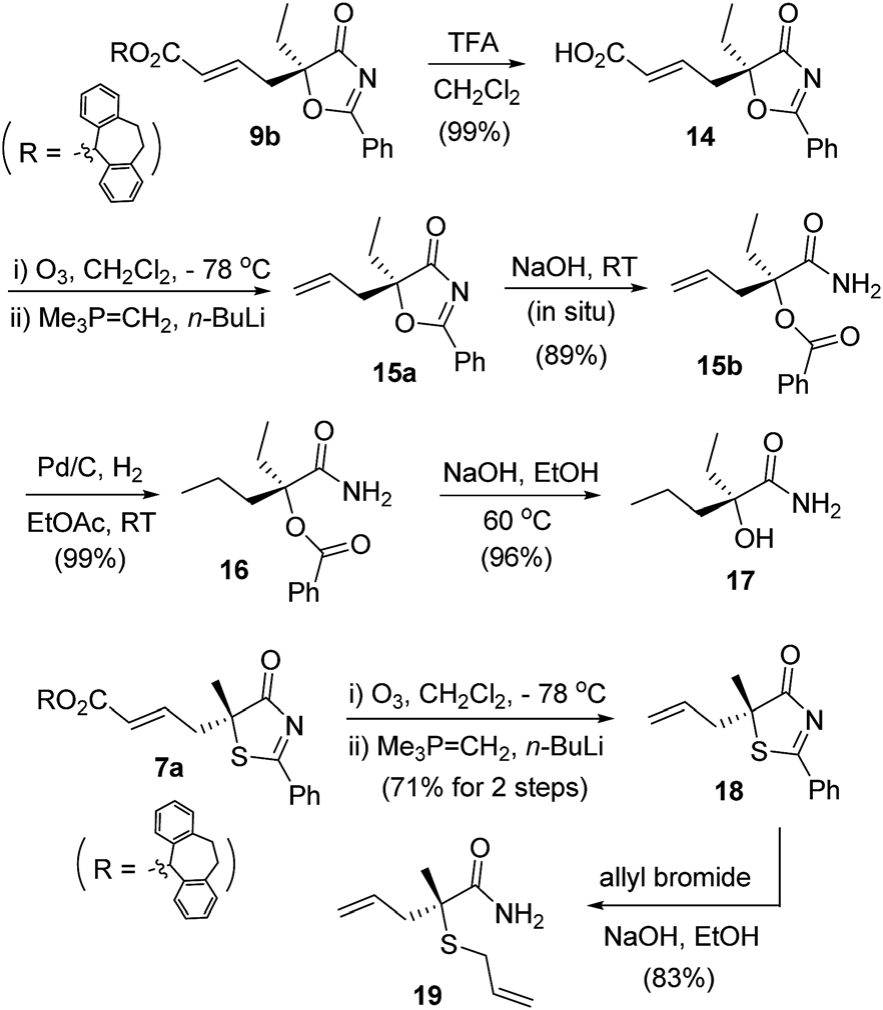
Elaboration of the γ-addition adducts into enantioenriched tertiary alcohols/thioethers.

The γ-addition products obtained possess a tertiary stereogenic center linked to a buried heteroatom, and they are valuable precursors for the convenient synthesis of enantiomerically enriched tertiary thiols/alcohols. As illustrated in Scheme 4, adduct **9b** was converted to allyl oxazolone **15a**, which was then treated with base to effect a ring opening, leading to the formation of a masked tertiary alcohol **15b** in excellent yield. Reduction of the double bond and cleavage of the ester afforded a tertiary α-hydroxy acid derivative **17**, which has both an ethyl and a propyl group present in the tertiary alcohol structure. Similarly, thiazolone **7a** was transformed to allyl-substituted **18**, which was readily converted to an enantiomerically enriched tertiary thioether **19** *via* a base-catalyzed ring opening.^23^

### 2.4. Mechanistic insights and DFT calculations

**Scheme 5:**
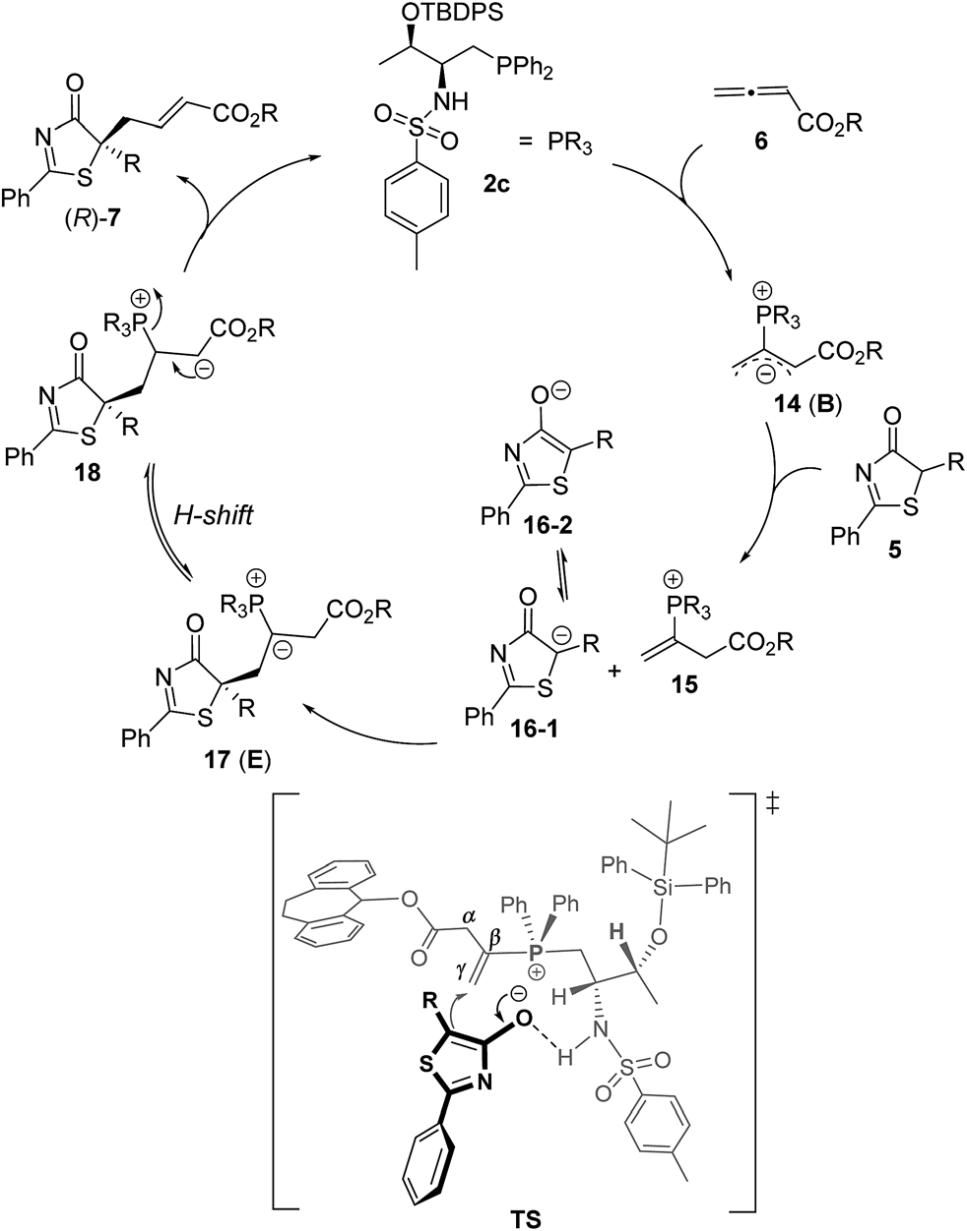
Proposed mechanism for the **2c**-catalyzed γ-addition of 5*H*-thiazol-4-one to allenoate.

Despite the popularity of phosphine-catalyzed organic reactions, mechanistic investigations remain very limited; a few theoretical studies appearing in the literature were disclosed by the groups of Yu,^24a–c^ Kwon,^24d,e^ and others.^24f–h^ We hypothesized that the hydrogen-bonding interactions between the Brønsted acid moiety of the phosphine catalyst and the donor molecules were essential for inducing enantioselectivity in our early reports on bifunctional phosphine catalyzed Michael and γ-addition reactions,^18,20^ and we believe such interactions are also crucial in our current reaction systems. The mechanism of the γ-addition of 5*H*-thiazol-4-one to allenoate is shown in Scheme 5, which follows the general mechanism described in the literature for γ-addition reactions.^19,20^ The reaction is initiated by the nucleophilic attack of the phosphorus atom on allenoate to form intermediate **B**, which is weakly basic. Deprotonation of the donor 5*H*-thiazol-4-one by **B** then affords the corresponding enolate, which subsequently attacks the γ-carbon of the allenoate to give intermediate **E**. Proton transfer takes place to afford **18**,^20^ and this is followed by the elimination of the phosphine catalyst to furnish the final addition product. We propose that a hydrogen bonding interaction between the sulfonamide N–H and the thiazolone enolate dictates its addition to the C–C double bond, which is the key step for asymmetric induction.

In an effort to provide theoretical support for the proposed reaction mechanism and to rationalize the origin of enantioselectivity, detailed density functional theory (DFT) calculations were conducted. DFT methods, as implemented in the Gaussian 09 (ref. 25) program, have been employed to study the model reaction involving reactants 5*H*-thiazol-4-ones **5a**, allenoate **6c** and catalyst **2c**. All the stationary points were optimized at the B3LYP^26^/6-31G (d) level^27^ of theory. The vibrational frequencies were computed at the same level of theory to determine whether the optimized structure was at an energy minimum or a transition state and to evaluate the corrections of enthalpy and Gibbs free energy. Solvent effects were computed by the IEFPCM^28^ solvation model at the M11 (ref. 29)/6-311+G (d)^30^ and B3LYP-D3 (ref. 31)/6-311+G (d) levels of theory using the gas phase optimized structures. The conclusions are similar for both methods, and the B3LYP-D3 calculated Gibbs free energies in toluene are discussed in the text.

**Fig. 2:**
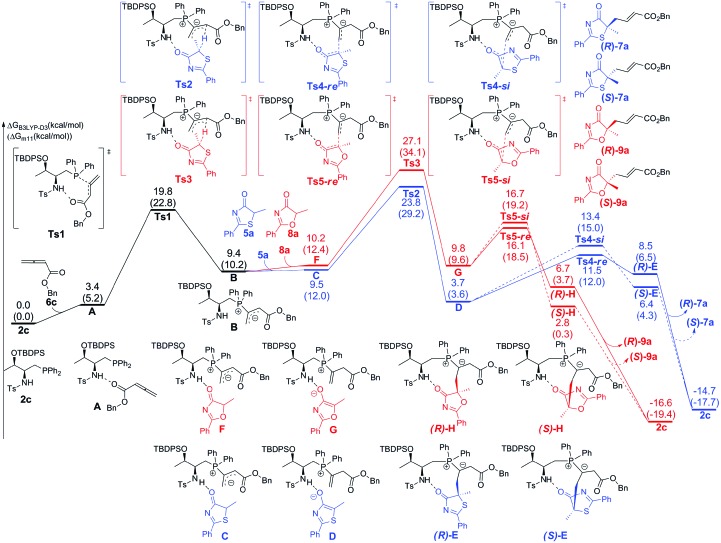
The DFT computed energy surfaces of the γ-addition reaction of **5a** and **8a** to allenoate **6c**. The values given in kcal mol^–1^ are the B3LYP-D3 calculated relative free energies in toluene. The values in parentheses are the M11 calculated relative free energies in toluene.

The calculated Gibbs free energy profiles for the phosphine-catalyzed γ-addition of **5a** to **6c** are summarized in Fig. 2 (blue line). As proposed, the reaction is initiated by the nucleophilic attack of the phosphine catalyst **2c** on allenoate **6c** *via* a transition state **Ts1** with a barrier of 19.8 kcal mol^–1^. This process is facilitated by the NH···O hydrogen bond, which brings the phosphine and the allene groups into proximity. A zwitterionic intermediate **B** is first formed, endothermically and reversibly, with an overall barrier of 9.4 kcal mol^–1^. Subsequent proton transfer between intermediate **B** and reactant **5a** takes place *via* transition state **Ts2** with a barrier of 14.4 kcal mol^–1^ (an overall barrier of 23.8 kcal mol^–1^). The nucleophilic attack can then occur *via* two possible pathways: the *Re*-face attack occurs through transition state **Ts4-*Re*** with a barrier of 7.8 kcal mol^–1^ to give intermediate **E** with *R*-configuration ((*R*)-**E**); and the alternative *Si*-face attack proceeds *via* transition state **Ts4-*Si*** with a barrier of 9.7 kcal mol^–1^, 1.9 kcal mol^–1^ higher than that of **Ts4-*Re***, leading to an intermediate with *S*-configuration ((*S*)-**E**). The corresponding addition product **7a** can be generated by proton transfer and elimination of the catalyst, the pathway similar to those reported by Yu and co-workers.^24a–c^ These observations suggest that the enantioselectivity is determined by the nucleophilic attack step and a value of 92% ee predicted by the B3LYP-D3 method based on the energy difference of **Ts4-*Re*** and **Ts4-*Si*** is in good agreement with the experimental result, where the *R*-product was formed preferentially (89% ee, entry 7 in Table 1). When sulfur is replaced by oxygen (5-methyl-2-phenyloxazol-4(5*H*)-one (**8a**)), the B3LYP-D3 calculations predict a value of 47% ee for the *R*-isomer (based on the energy difference of 0.6 kcal mol^–1^ between transition states **Ts5-*Re*** and **Ts5-*Si***), consistent with the experimental observation (65% ee, entry 1 in Table 4).

**Fig. 3:**
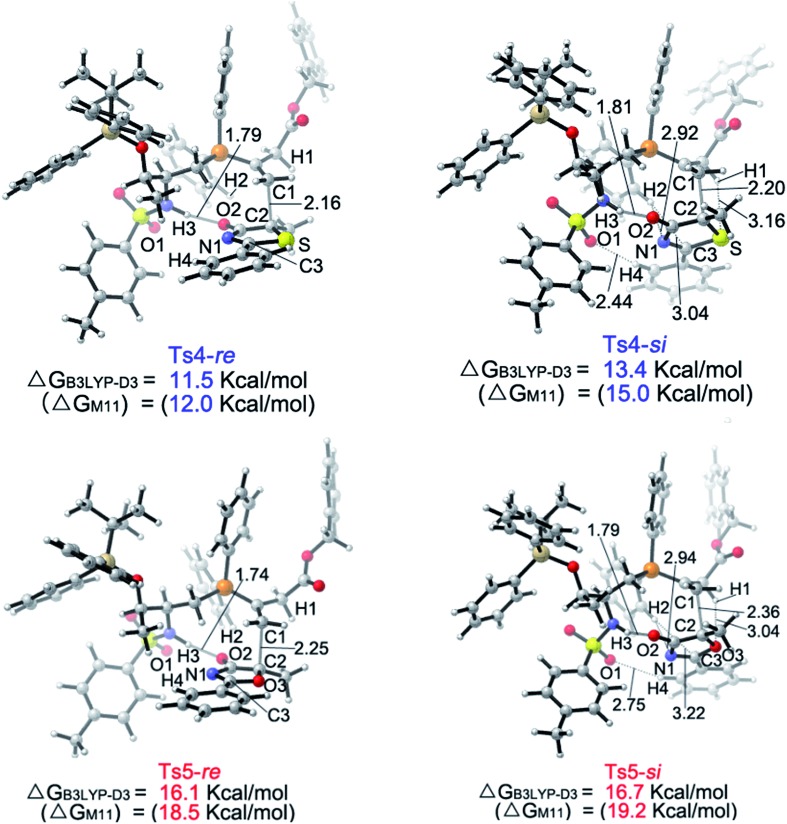
Geometries of the **Ts4-*Re***, **Ts4-*Si***, **Ts5-*Re*** and **Ts5-*Si*** transition states. The values for the bond lengths are given in angstroms.

**Fig. 4:**
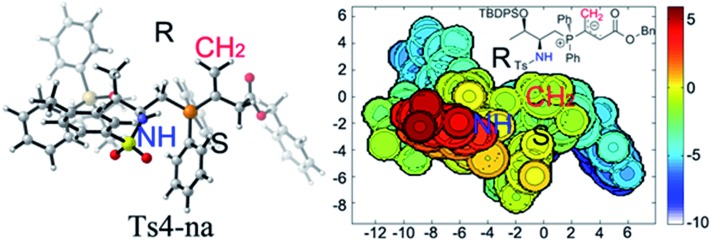
2D contour map of the van der Waals surface of catalyst **2c** and allenoate **6c**. Distances are reported in Å. The C atom of the CH_2_ group (labelled by red “CH_2_”) is located at the origin of the coordinate system in the contour map. A contour line of zero is defined as being in the same plane of the C atom. A negative distance (blue) indicates the atoms on the complex are farther away from substrate; a positive distance (red) indicates the atoms on complex are closer to substrate.

Upon evaluating transition states **Ts4-*Re*** and **Ts4-*Si*** (Fig. 3), it was found that the bond lengths for the forming C1–C2 bond are similar, in addition to the distances of the hydrogen bonds between H3 and O2 (about 1.8 Å). However, the short H2···C3 distance of 3.04 Å in **Ts4-*Si*** suggests a repulsion between the phenyl group of the reacting thiazolone and one of the phenyl groups in the phosphine catalyst, resulting in a higher transition state barrier. To better illustrate the steric repulsions in the nucleophilic addition step, a 2D contour map along the *z*-axis (defined as the forming C–C bond) of the van der Waals^32^ surface of **Ts4-na** is plotted (Fig. 4), representing the nucleophile moiety of transition state **Ts4-*Re*** without the thiazolone substrate. When the thiazolone group is deprotonated and bound to the catalyst *via* the N–H···O hydrogen bond during the formation of the C–C bond along the *z*-axis, the steric hindrance for the *Re*-face attack (labelled as *R*) is smaller than that for the *Si*-face attack (labelled as *S*). As such, *Re*-face attack becomes more favorable.

### 2.5. Experimental confirmation

The importance of hydrogen bonding interactions for asymmetric induction has been clearly demonstrated in the above computational studies. Sulfonamide **2c** and its close structural analogs **2c′** and **2c′′** were synthesized and applied to the γ-addition of 5*H*-thiazol-4-one **5a** to allenoate **6f** (Table 7). Blockage of the sulfonamide N–H led to a dramatic decrease in reactivity and enantioselectivity (entry 2). When the sterically hindered *O*-silyl group was replaced by a free OH, not only the enantioselectivity, but also the reactivity of the reaction decreased significantly (entry 3), suggesting that the bulky silyl group may be crucial for locking the transition state geometry and differentiating the *Re*- and *Si*-face attacks.

**Table 7:** Asymmetric γ-addition of 5*H*-thiazol-4-one **5a** promoted by different phosphines^*a*^

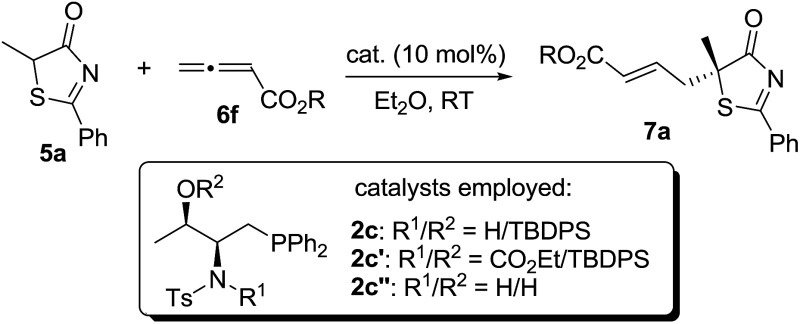				
Entry	Catalyst	*t* (h)	Yield^*b*^ (%)	ee^*c*^ (%)
1	**2c**	12	97	95
2	**2c′**	30	82	37
3	**2c′′**	24	95	53

^*a*^Reactions were performed with **5a** (0.1 mmol), **6f** (0.12 mmol) and the catalyst (0.01 mmol) in Et_2_O (1.0 mL) at room temperature.

^*b*^Isolated yield.

^*c*^Determined by HPLC analysis on a chiral stationary phase.

## 3. Conclusions

In summary, we have developed the first phosphine-catalyzed highly enantioselective γ-addition of 5*H*-thiazol-4-ones and 5*H*-oxazol-4-ones to 2,3-butadienoates. In the presence of amino acid-derived bifunctional phosphine catalysts, chiral thiazolones and oxazolones with a heteroatom (S or O)-containing tertiary chiral center were obtained in high yields and with excellent enantioselectivities. The optically enriched adducts are synthetically valuable, enabling the facile synthesis of optically enriched tertiary alcohols and thioethers. The method described in this report represents a method for rapid access to enantioenriched tertiary alcohol and thiol derivatives bearing an allylic chain, and may find wide applications in synthetic organic chemistry. DFT calculations for understanding the mechanism revealed that the observed enantioselectivity results from a combination of three factors: (1) the hydrogen-bonding interaction between the amino moiety of the phosphine catalyst and the “C=O” unit of the thiazolone to activate the Michael donor, (2) the N–H···O interaction and the bulky *O*-silyl group to lock the conformation, and (3) the phenyl group of the thiazolone to differentiate the stereochemistry. It is noteworthy that this is the first complete theoretical study for phosphine-catalyzed γ-addition reactions. The theoretical results presented here are expected to offer new insight into the mechanisms of other phosphine-catalyzed asymmetric reactions, particularly those triggered by amino acid-derived phosphine catalysts.
