# Supplementary material for: Highly enantioselective construction of tertiary thioethers and alcohols via phosphine-catalyzed asymmetric γ-addition reactions of 5H-thiazol-4-ones and 5H-oxazol-4-ones: scope and mechanistic understandings
Source: Chem Sci. 2015 Jun 2;6(8):4912–22. doi: 10.1039/c5sc01614b (PMC5664357; doi:10.1039/c5sc01614b)
Supplement: Supplementary file 1 [file SC-006-C5SC01614B-s001.pdf]

Electronic Supplementary Material (ESI) for Chemical Science.
This journal is © The Royal Society of Chemistry 2015

SUPPORTING INFORMATION

Highly Enantioselective Construction of Tertiary Thioethers and Alcohols via Phosphine-Catalyzed Asymmetric γ-Addition reactions of 5H-Thiazol-4-ones and 5H-Oxazol-4-ones: Scope and Mechanistic Understandings

Tianli Wang,[a] Zhaoyuan Yu,[b] Ding Long Hoon,[a] Kuo-Wei Huang,[c] Yu Lan,[b,*] Yixin Lu[a,*]

[a] Department of Chemistry, National University of Singapore 3 Science Drive 3, Singapore 117543 (Singapore).
[b] School of Chemistry and Chemical Engineering, Chongqing University, Chongqing 400030, P. R. China.
[c] Division of Physical Sciences and Engineering and KAUST Catalysis Center, King Abdullah University of Science and Technology, Thuwal 23955-6900, Saudi Arabia.

E-mail: chmlyx@nus.edu.sg; lanyu@cqu.edu.cn

1. General Information --2
2. Optimization of Reaction Conditions and Determination of Adducts' Configurations -------------3
3. Proposed Reaction Cycle and Experimental Studies on Mechanism ----------------------------------8
4. Preparation of the Catalysts --9
5. Preparation of Allenoates **6** and 2-Butynoate **6'**--11
6. Preparation of Substituted 5*H*-Thiazol-4-ones ---12
7. Preparation of Substituted 5*H*-Oxazol-4-ones ---15
8. Representative Procedure for the γ-Addition of 5*H*-Thiazol-4-ones --------------------------------17
9. Representative Procedure for the γ-Addition of 5*H*-Oxazol-4-ones ----------------------------------37
10. Asymmetric Synthesis of Chiral Tertiary Alcohols and Thioethers ------------------------------------55
11. DFT Study on Mechanism---59
12. References---110
13. NMR Spectra of the Products---111

1. General Information

All the starting materials were obtained from commercial sources and used without further purification unless otherwise stated. ^1H and ^{13}C NMR spectra were recorded at ambient temperature in $CDCl_3$ or d_6-DMSO on a Bruker ACF300 or AMX500 (500 MHz) spectrometer. The chemical shifts are reported in parts permillion (ppm) relative to CDC_3 (δ = 7.26) and to DMSO (δ = 2.50) for ^1H-NMR and relative to the central resonances of $CDCl_3$ (δ = 77.0) and to DMSO (δ = 39.5) for ^{13}C-NMR. Multiplicity was indicated as follows: s (singlet), d (doublet), t (triplet), q (quartet), m (multiplet), dd (doublet of doublet), br s (broad singlet). Coupling constants (*J*) were reported in Hertz (Hz). Low resolution mass spectra were obtained on a Finnigan/MAT LCQ pectrometer in ESI mode, and a Finnigan/MAT 95–T mass spectrometer in FAB mode. All high resolution mass spectra were obtained on a Finnigan/MAT 95XL–T spectrometer. For thin layer chromatography (TLC), Merck pre-coated TLC plates (Merck 60 F254) were used, and compounds were visualized with a UV light at 254 nm. Further visualization was achieved by staining with iodine, or ceric ammonium molybdate followed by heating on a hot plate. Flash chromatographic separations were performed on Merck 60 (0.040–0.063 mm) mesh silica gel. Enantiomeric excess was determined by HPLC analysis using chiral column described below in detail. Optical rotations were measured with polarimeter.

The catalysts **1-4** were prepared by following our previously reported procedures.[1] The substituted 5*H*-thiazol-4-ones[2,3] and 5*H*-oxazol-4-ones[4] were synthesized following the methods reported in the literatures, respectively.

2. Optimization of Reaction Conditions

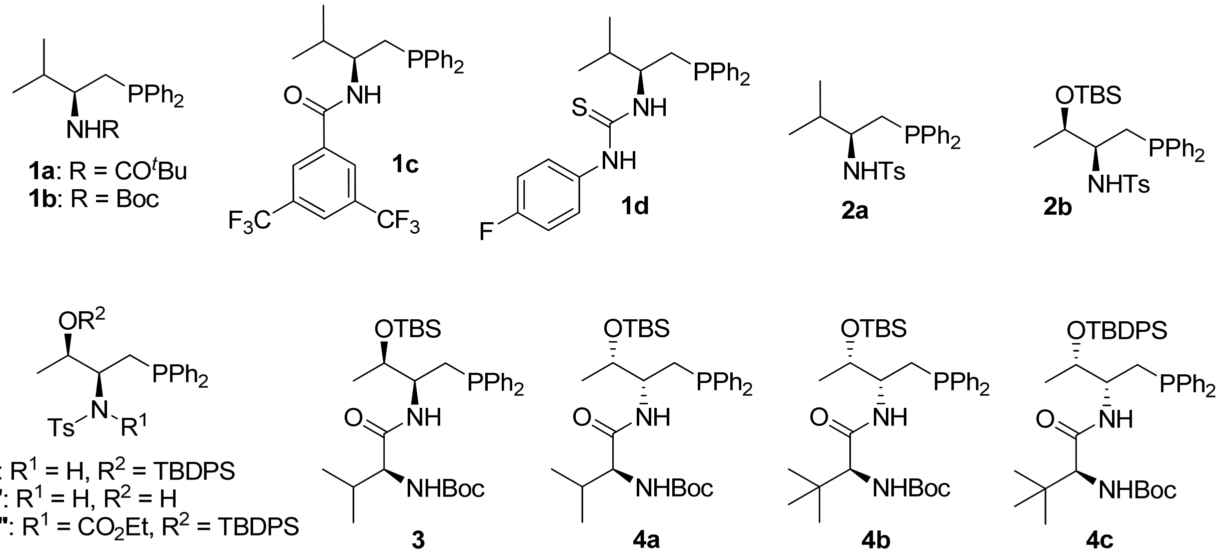

Scheme S1: Chiral phosphine catalysts used in this study.

(TBDPS = *tert*-butyldiphenylsilyl, TBS = *tert*-butyldimethylsilyl, Ts = 4-toluenesulfonyl)

A. Optimization of conditions for γ-addition of 5*H*-thiazol-4-ones

***Table S1*: Asymmetric γ-addition of 5*H*-thiazol-4-one **5a** with allenoate **6c** catalyzed by different chiral phosphines in toluene [a]

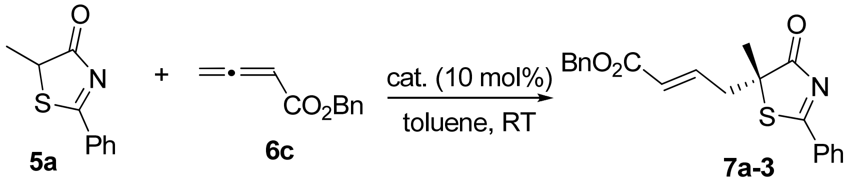

Entry	Cat.	*t* [h]	Yield [%][b]	*ee* [%][c]
1	**1a**	12	86	10
2	**1b**	12	90	62
3	**1c**	12	86	34
4	**1d**	12	92	34
5	**2a**	12	91	66
6	**2b**	12	90	78
**7**	**2c**	**12**	**95**	**89**

8	**3**	12	88	47
9	**4a**	12	92	-57
10	**4b**	12	88	-65
11	**4c**	12	92	-68

[a] Reactions were performed with **5a** (0.1 mmol), **6c** (0.12 mmol) and catalyst (0.01 mmol) in toluene (1.0 mL) at room temperature. [b] Yields of isolated products. [c] Determined by HPLC analysis on a chiral stationary phase.

***Table S2*:** Asymmetric γ-addition of 5*H*-thiazol-4-one **5a** with different allenoates (**6**) catalyzed by **2c** in toluene [a]

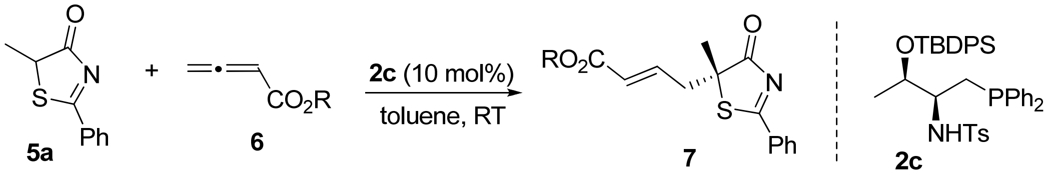

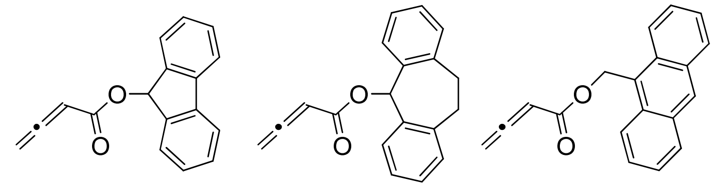

6e 6f 6g

Entry	R (**6**)	*t* [h]	Yield [%][b]	*ee* [%][c]
1	Et (**6a**)	12	95	87
2	^{t}Bu (**6b**)	12	95	88
3	Bn (**6c**)	12	95	89
4	$CHPh_2$(**6d**)	12	86	90
5	**6e**	12	88	70
**6**	**6f**	**12**	**96**	**91**
7	**6g**	12	93	87
8	Ph (**6h**)	12	91	57

[a] Reaction conditions: **5a** (0.10 mmol), **6** (0.12 mmol), and the catalyst **2c** (0.01 mmol) in toluene (1.0 mL). [b] Yields of isolated products. [c] Determined by HPLC analysis on a chiral stationary phase.

Table S3: Asymmetric γ-addition 5*H*-thiazol-4-one **5a** with allenoate **6f** catalyzed by **2c**: optimizing of the solvents and other reaction conditions [a]

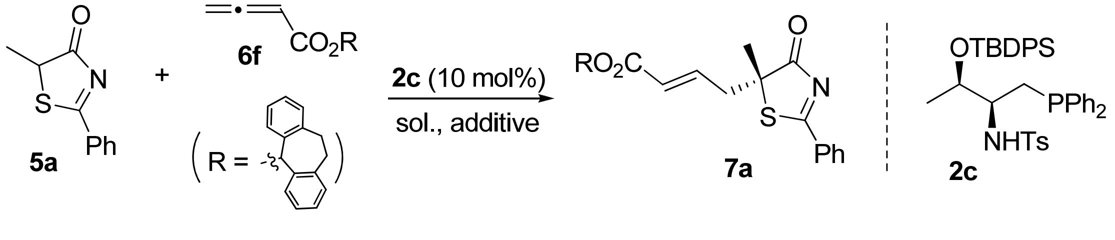

Entry	Solvent	Additive	T [°C]	*t* [h]	Yield [%][b]	*ee* [%][c]
1	toluene	none	RT	12	96	91
2	xylene	none	RT	12	95	93
**3**	**Et_2O**	**none**	**RT**	**12**	**97**	**95**
4	$CHCl_3$	none	RT	12	94	83
5	CH_2Cl_2	none	RT	15	87	92
6	Et_2O	3Å-MS	RT	12	97	94
7	Et_2O	4Å−MS	RT	12	97	94
8	Et_2O	5Å−MS	RT	12	96	94
9	toluene	none	0	36	86	90

[a] Reaction conditions: **5a** (0.10 mmol), **6f** (0.12 mmol), and the catalyst **2c** (0.01 mmol) in solvent (1.0 mL). [b] Yields of isolated products. [c] Determined by HPLC analysis on a chiral stationary phase.

B. Optimization of conditions for γ-addition of 5*H*-oxazol-4-ones

Table S4: Asymmetric γ-addition of 5*H*-oxazol-4-one **8a** with allene **6c** or **6f** in toluene: screening of the catalysts [a]

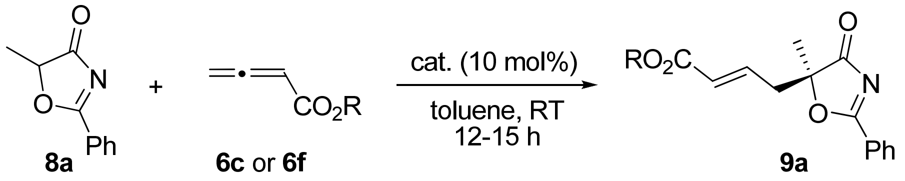

Entry	Cat.	Allene (**6c**)		Allene (**6f**)	
		Yield [%][b]	*ee* [%][c]	Yield [%][b]	*ee* [%][c]
1	**1a**	89	-60	90	-66
2	**1b**	91	-63	94	-72
3	**1c**	92	-34	89	-47
4	**1d**	87	-12	89	-27
5	**2a**	89	-58	96	-69
6	**2b**	88	-63	89	-70
7	**2c**	89	-65	90	-71
8	**3**	93	-59	94	-70
9	**4a**	94	70	93	77
10	**4b**	94	73	91	81
**11**	**4c**	**95**	**76**	**95**	**86**

[a] Reaction conditions: **8a** (0.10 mmol), **6** (0.12 mmol), and the catalyst (0.01 mmol) in toluene (1.0 mL) at room temperature. [b] Yields of isolated products. [c] Determined by HPLC analysis on a chiral stationary phase.

***Table S5*: Asymmetric γ-addition of 5*H*-oxazol-4-one 8a with different allenes (6) by 4c** [a]

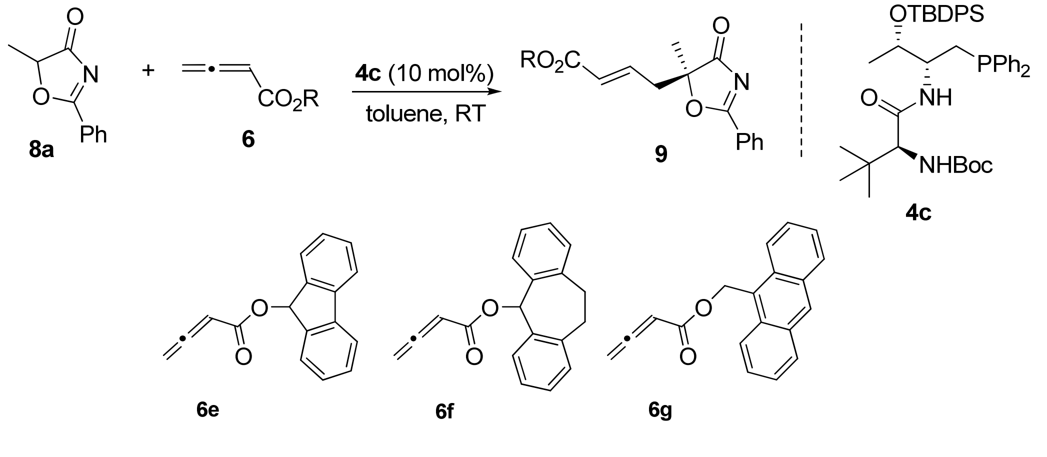

Entry	R (**6**)	*t* [h]	Yield [%][b]	*ee* [%][c]
1	Et (**6a**)	12	93	77
2	^{t}Bu (**6b**)	12	95	82
3	Bn (**6c**)	12	95	76
4	$CHPh_2$(**6d**)	12	96	84
5	**6e**	12	95	84

**6**	**6f**	**12**	**96**	**86**
7	**6g**	12	85	79
8	Ph (**6h**)	12	89	67

[a] Reaction conditions: **8a** (0.10 mmol), **6** (0.12 mmol), and the catalyst **4c** (0.01 mmol) in toluene (1.0 mL). [b] Yields of isolated products. [c] Determined by HPLC analysis on a chiral stationary phase.

***Table S6*:** Asymmetric γ-addition 5*H*-oxazol-4-one **8a** with allene **6f** catalyzed by **4c**: screening solvents, additives and reaction temperature[a]

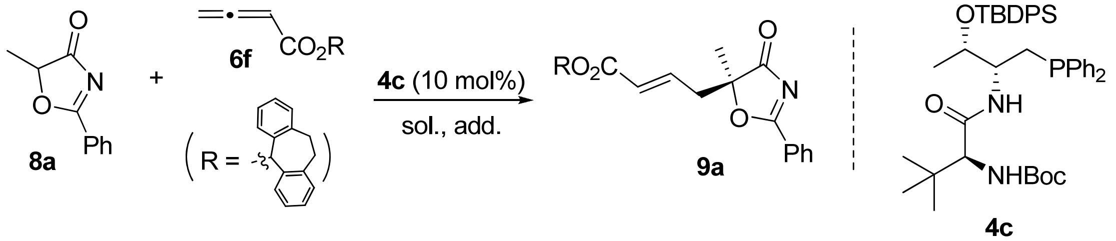

Entry	Solvent	Additive	T (°C)	*t* [h]	Yield [%][b]	*ee* [%][c]
1	toluene	none	RT	12	96	86
2	xylene	none	RT	12	95	85
**3**	**Et_2O**	**none**	**RT**	**12**	**97**	**88**
4	$CHCl_3$	none	RT	12	92	46
5	CH_2Cl_2	none	RT	12	90	50
6	CH_3CN	none	RT	12	82	67
**7**	**Et_2O**	**3Å-MS**	**RT**	**12**	**97**	**92**
8	Et_2O	4Å−MS	RT	12	96	91
9	Et_2O	5Å−MS	RT	12	96	90
10	Et_2O	3Å-MS	0	20	86	91

[a] Reaction conditions: **8a** (0.10 mmol), **6f** (0.12 mmol), and the catalyst **4c** (0.01 mmol) in solvent (1.0 mL). [b] Yields of isolated products. [c] Determined by HPLC analysis on a chiral stationary phase.

C. Determination of Adducts' Configurations

The absolute configuration of γ-addition of 5*H*-oxazol-4-one adduct **9b** was assigned to be *S* by comparing optical rotation of the corresponding derivative **13** (Scheme S3) reported in the literature,[5] and other configurations of γ-addition products **9** were assigned by analogy. Accordingly, the absolute configuration of γ-addition of 5*H*-thiazol-4-ones adducts **7** were tentatively proposed to be *R* by analogy.

3. Proposed Mechanism and Mechanism Studies

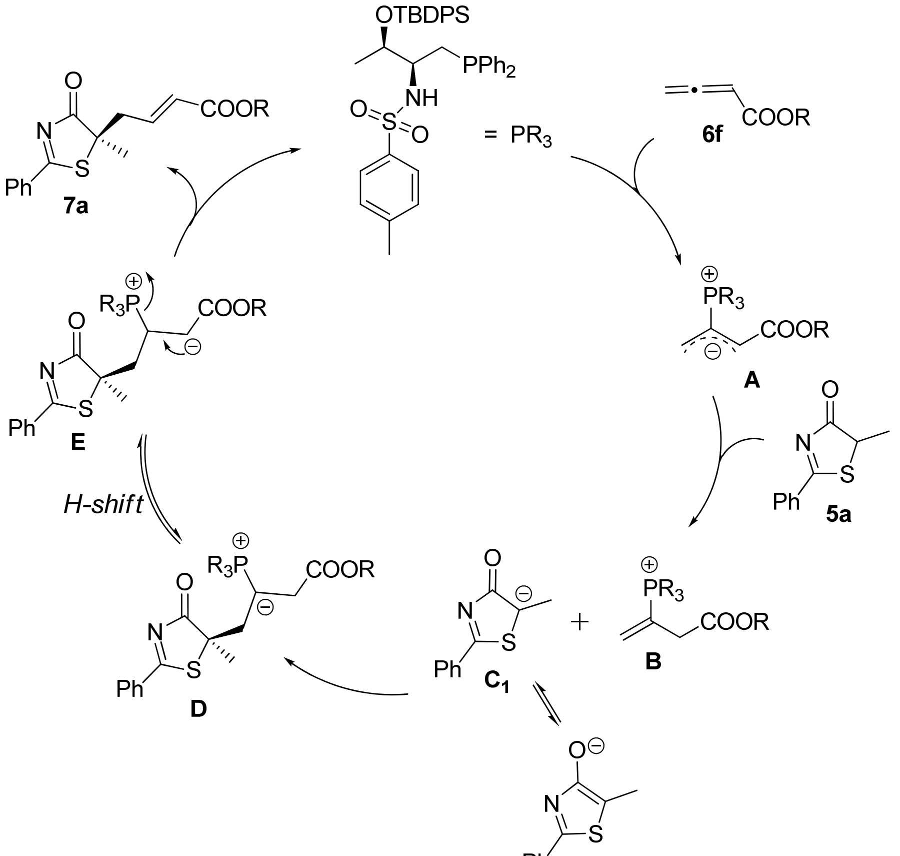

Figure S1: Proposed mechanism.

Table S7. Asymmetric γ-addition promoted by different phosphine catalysts.[a]

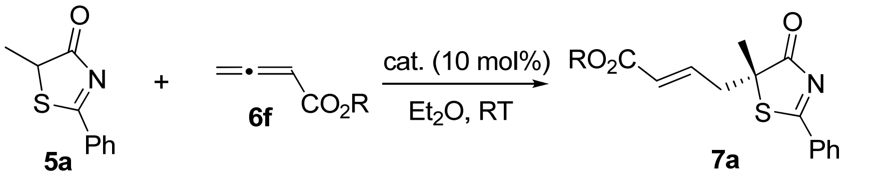

Entry	Cat.	t [h]	Yield [%][b]	ee [%][c]
1	**2c**	12	97	95
2	**2c’**	30	82	37
3	**2c”**	24	95	53

[a] Reaction conditions: **5a** (0.10 mmol), **6f** (0.12 mmol), and catalyst (0.01 mmol) in Et_2O (1.0 mL). [b] Isolated yield. [c] Determined by HPLC analysis on a chiral stationary phase.

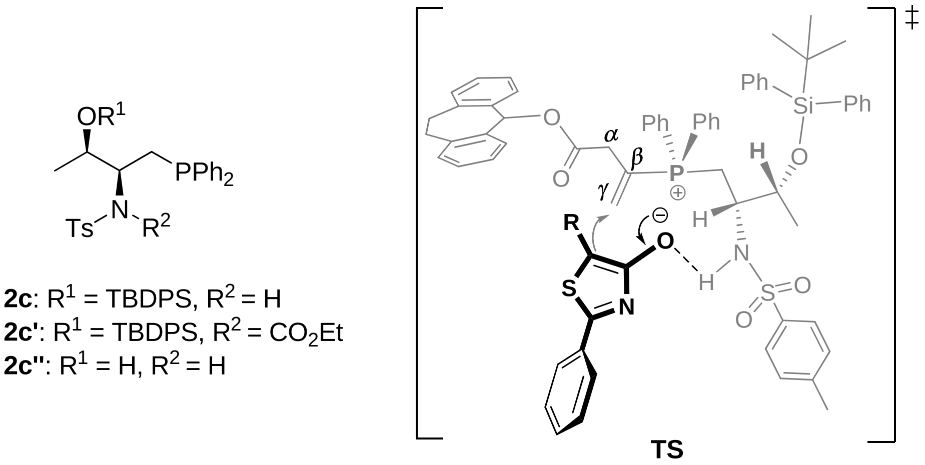

Scheme S2. Proposed transition-state model.

4. Preparation of the Catalysts

All known phosphine catalysts **1-4** were synthesized according to the reported methods described in our previous publications.[1] Catalyst **2c’** and **2c’’** were firstly prepared from **2c** via the following procedures.

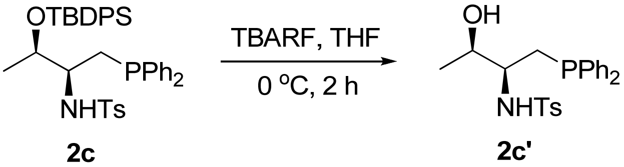

To a stirred solution of compound **2c** (133 mg, 0.2 mmol) in anhydrous THF (2 mL) was added a solution of 1.0 M TBARF in THF (0.4 mL, 0.4 mmol, 2.0 eq.) at 0 °C under N_2. The resulting mixture was stirred at the same temperature until the reaction was complete (monitored by TLC). Then, the solvent of THF was removed under reduced pressure and the residue was purified directly by flash

column chromatography (hexane/ethyl acetate = 5 : 1) to afford **2c'** as a white foam (79 mg, 92% yield).

N-((2S,3R)-1-(Diphenylphosphino)-3-hydroxybutan-2-yl)-4-methylbenzenesulfonamide (2c')

^{1}H NMR (500 MHz, $CDCl_3$) δ 7.62 (d, *J* = 8.2 Hz, 2H), 7.36-7.27 (m, 10H), 7.19 (d, *J* = 7.6 Hz, 2H), 5.42 (d, *J* = 8.2 Hz, 1H), 4.16-4.11 (m, 1H), 3.19-3.17 (m, 1H), 2.42-2.34 (m, 4H), 2.18 (d, *J* = 10.8 Hz, 1H), 1.00 (d, *J* = 6.3 Hz, 3H); ^{13}C NMR (125 MHz, $CDCl_3$) δ 143.13, 137.72, 133.02 (dd, $J_{1,2}$ = 18.9 Hz, $J_{1,3}$ = 70.1 Hz), 129.52, 129.11, 128.79, 128.61 (dd, $J_{1,2}$ = 7.3 Hz, $J_{1,3}$ = 13.7 Hz), 127.10, 67.83 (d, *J* = 9.1 Hz), 56.72 (d, *J* = 15.3 Hz), 31.91 (d, *J* = 13.6 Hz), 21.47, 19.98; ^{31}P NMR (121 MHz, $CDCl_3$) δ -23.48; HRMS (ESI) *m/z* calcd $C_{23}H_{27}NO_3PS$ $[M+H]^+$ = 428.1444, found = 428.1455.

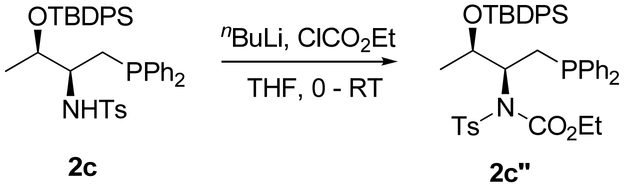

To a stirred solution of **2c** (65 mg, 0.1 mmol) in anhydrous THF (1.0 mL) at 0 °C was added a solution of 2.0 M ^{n}BuLi in hexane (0.2 mL, 0.4 mmol, 4.0 eq.) by dropwise under the N_2. The resulting solution was stirred for 30 min, and then warmed to room temperature and stirred for another 2 h. Then, the mixture was cooled to 0 °C with an ice bath and ethylchloroformate (43 mg, 0.4 mmol) was added, and the reaction system was stirred at RT for further 1 h. The reaction was then quenched with saturated saturated ammonium chloride (1 mL), and extracted with CH_2Cl_2 several times (3 × 2 mL). The combined organic extracts were dried over Na_2SO_4, filtered and concentrated. The crude product was purified directly by flash column chromatography (hexane/ethyl acetate = 15 : 1) to afford **2c''** as a white solid (60 mg, 81% yield) with *dr* = 85/15 (based on the ^{31}P-NMR).

Ethyl (2*S*,3*R*)-3-(*tert*-butyldiphenylsilyloxy)-1-(diphenylphosphino)butan-2-yl(tosyl)carbamate (2c'')

^{1}H NMR (500 MHz, $CDCl_3$) δ 7.69-7.66 (m, 5H), 7.53 (t, *J* = 5.7 Hz, 2H), 7.51-7.29 (m, 16H), 7.20 (d, *J* = 8.9 Hz, 1H), 4.62 (br, 1H), 4.34-4.30 (m, 3H), 4.04-4.00 (m, 2H), 2.40 (s, 3H), 1.34 (t, *J* = 7.0 Hz, 3H), 0.98 (s, 9H), 0.93 (d, *J* = 6.3 Hz, 3H); ^{13}C NMR (125 MHz, $CDCl_3$) δ 167.52, 143.33, 135.95 (d, *J* = 4.6 Hz), 133.50, 133.35, 132.48 (d, *J* = 17.3 Hz), 130.48, 129.52, 129.33, 129.07, 128.63 (dd, $J_{1,2}$ = 12.3 Hz, $J_{1,3}$ = 30.1 Hz), 127.51, 127.33, 63.16, 62.22, 29.68, 26.87, 22.68, 21.28, 19.17, 13.92, 13.63; ^{31}P NMR (121 MHz, $CDCl_3$) δ -22.03 (major isomer), -22.66 (minor isomer); HRMS (ESI) *m/z* calcd

$C_{42}H_{48}NNaO_5PSSi$ $[M+Na]^+$ = 760.2652, found = 760.2664.

5. Preparation of Allenoate 6f and 2-Butynoate 6‘

The allenoates (**6**) were synthesized according to the following procedure, and all allenoates used in this study were known compounds.[1g]

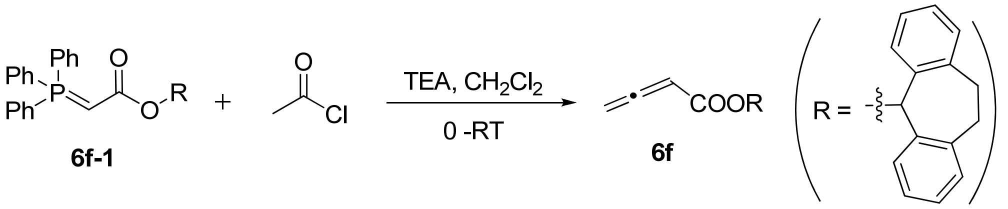

To a solution of **6f-1**(3.76 g, 7.34 mol) and TEA (0.74 g, 7.34 mol) in dry CH_2Cl_2 (150 mL) was dropwise added the acetyl chloride (0.58 g, 7.34 mol) at 0 °C, and the reaction mixture was stirred at 0 °C for 2 h. Then, saturated aqueous NH_4Cl was added to quench the reaction, and the resulting mixture was extracted with CH_2Cl_2 several times (3 × 50 mL). The combined organic extracts were dried over Na_2SO_4, filtered and concentrated. The residue was purified by flash column chromatography (hexane/ethyl acetate = 5:1) to afford the title **6f** as a white solid (1.78 g, 88% yield). ^1H NMR (500 MHz, $CDCl_3$) δ 7.56 (dd, $J_{1,2}$ = 3.2 Hz, $J_{1,3}$ = 7.6 Hz, 2H), 7.34-7.31 (m, 2H), 7.27-7.25 (m, 4H), 7.10 (s, 1H), 5.75 (t, J = 6.3 Hz, 1H), 5.26 (d, J = 6.3 Hz, 2H), 3.68-3.61 (m, 2H), 3.15-3.08 (m, 2H); ^{13}C NMR (125 MHz, $CDCl_3$) δ 215.92, 164.34, 139.85, 136.43, 130.14, 129.35, 128.58, 125.98, 88.09, 79.17, 79.09, 32.14; HRMS (ESI) *m/z* calcd for $C_{19}H_{16}NaO_2$ $[M+Na]^+$ = 299.1043, found = 299.1053.

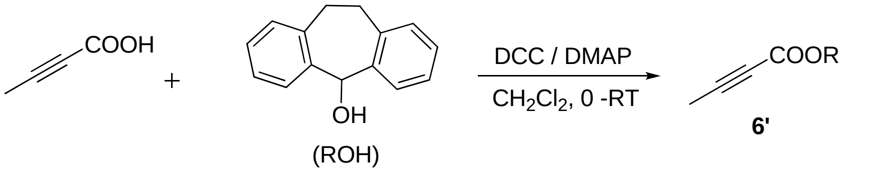

To a stirred solution of but-2-ynoic acid (220 mg, 2.62 mmol) and dibenzosuberol (550 mg, 2.62 mmol) in anhydrous CH_2Cl_2 (5 mL) was added a solution of DCC (647 mg, 3.14 mmol, 1.2 eq.) and DMAP (30 mg, 0.1 eq.) in CH_2Cl_2 (3 mL) by dropwise at 0 °C, and the resulting mixture was stirred at room temperature for 4 h. Then, water (5 mL) was added to quench the reaction, and the resulting mixture was extracted with dichloromethane several times (3 x 4 mL). The combined organic extracts were dried over sodium sulfate, filtered and concentrated, and the residue was purified by flash column chromatography (hexane: ethyl acetate = 5:1) to afford **6’**as white solid (528 mg, 73%). ^1H NMR (500

MHz, $CDCl_3$) δ 7.44 (d, J = 7.6 Hz, 2H), 7.28-7.24 (m, 2H), 7.20-7.17 (m, 4H), 6.94 (s, 1H), 3.62-3.56 (m, 2H), 3.07-3.00 (m, 2H), 1.95 (s, 3H); ^{13}C NMR (125 MHz, $CDCl_3$) δ 152.63, 140.16, 135.88, 130.31, 129.86, 128.92, 126.14, 85.76, 80.51, 72.61, 32.35, 3.78; HRMS (ESI) *m/z* calcd for $C_{19}H_{16}NaO_2$ $[M+Na]^+$ = 299.1043, found = 299.1039.

6. Preparation of Substituted 5*H*-Thiazol-4-ones (5)

General procedure for preparing **5a**, **5b** and **5l**:[2b-d]

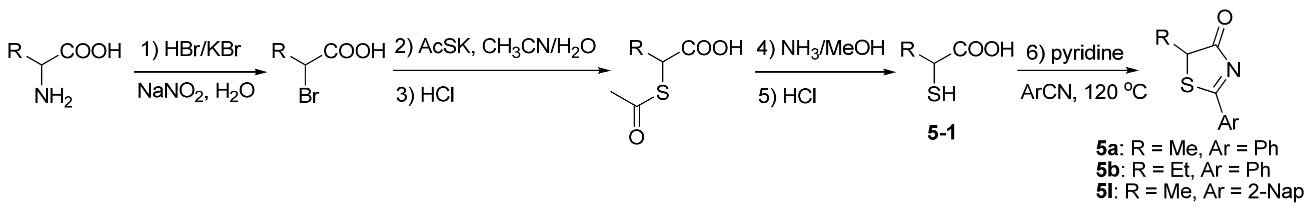

The substrates **5a**, **5b** and **5l** were prepared from compounds **5-1**[2d] following literature procedure.[2b,c] They are known compounds, and their characterization data were in agreement with those reported in Ref. 2a.

Under the N_2 atmosphere, to a mixture of ArCN (1.0 eq.) and pyridine (20 mol %) was added the corresponding α-mercaptocarboxylic acid (1.0 eq.) at room temperature, and the mixture was stirred overnight at 120 ºC. After cooling to room temperature, a yellow solid could be observed, and was collected by filtration and washed with cold methanol to give the pure product.[3]

General procedure for preparing **5e** and **10**:[2a]

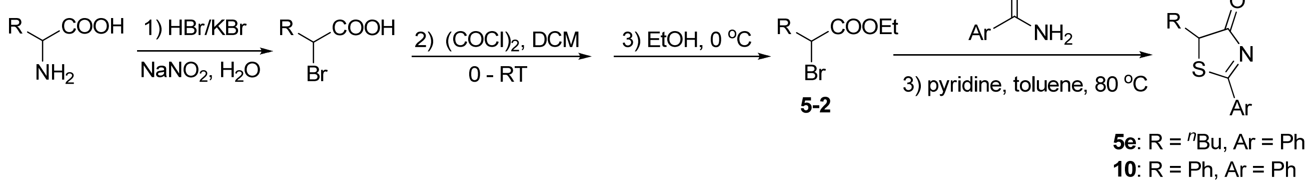

The know substrate **5e** and **10** were prepared from compounds **5-2** following literature procedure, and their characterization data were in agreement with those reported in the literature.[2a]

To a solution of thiobenzamide (1.37 g, 10 mmol) and pyridine (3.16 g, 40 mmol) in toluene (150 mL) was dropwise added the ethyl 2-bromohexanoate **5-2** (2.22 g, 10 mmol) at room temperature,

and the reaction mixture was stirred at 80 °C for overnight. Then, after cooling to RT, the precipitate was formed and recrystalliized from ethanol to afford **5e** as yellow solid (1.34 g, 58% yield).

General procedure for preparing **5c, 5d and 5f-k**:

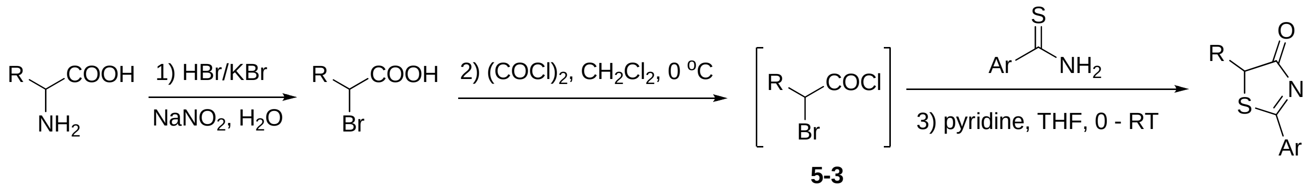

To a solution of thiobenzamide (0.68 g, 5 mmol) and pyridine (0.48 g, 6 mmol) in anhydrous THF (100 mL) at 0 °C was dropwise added the solution of 2-bromopentanoyl chloride (6 mmol) in CH_2Cl_2 (5 mL) which was *in situ* prepared from 2-bromopentanoic acid, and the reaction mixture was stirred at 0 °C for 1 h and at room temperature for another 2 h. During this time, a white salt of pyridine hydrochloride was formed which was then removed by filtration. The solvent of THF was removed under reduced pressure, and the residue was purified directly by flash column chromatography (hexane/ethyl acetate = 5 : 1) to afford a yellow foam which was further recrystalliized from ether/hexane to give pure **5c** as yellow solid (667 mg, 61% yield).

The substrates including **5c**, **5d** and **5f-k** were prepared from **5-3** via above shown procedure, which is the similar method to that reported in literature.[2a] Compounds **5c** and **5k** are known compounds, and their characterization data were in agreement with those reported in the literature.[2a] Unknown compounds **5d**, **5f-k** and **5j** were fully characterized.[3]

5-isopropyl-2-phenylthiazol-4(5*H*)-one (5d)

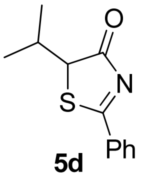

5d

A yellow solid; 41% yield; ^{1}H NMR (300 MHz, d_6-DMSO) δ 10.36 (bs, 1H), 7.80 (d, J = 7.7 Hz, 2H), 7.48-7.41 (m, 3H), 3.22-3.13 (m, 1H), 1.22 (d, J = 6.7 Hz, 6H); ^{13}C NMR (75 MHz, d_6-DMSO) δ

158.27, 157.54, 133.86, 129.81, 129.50, 125.15, 116.08, 25.27, 24.49; HRMS (ESI) *m/z* calcd $C_{12}H_{12}NOS$ [M-H]$^-$ = 218.0645, found = 218.0654.

5-isobutyl-2-phenylthiazol-4(5*H)*-one (5f)

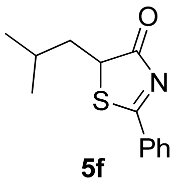

5f

A yellow solid; 41% yield; ^{1}H NMR (300 MHz, d_6-DMSO) δ 10.58 (bs, 1H), 7.79 (d, *J* = 6.8 Hz, 2H), 7.48-7.41 (m, 3H), 2.51 (d, *J* = 6.9 Hz, 2H), 1.84-1.75 (m, 1H), 0.90 (d, *J* = 6.6 Hz, 6H); ^{13}C NMR (75 MHz, d_6-DMSO) δ 159.35, 159.03, 133.69, 129.88, 129.50, 125.19, 107.35, 33.12, 30.15, 22.35; HRMS (ESI) *m/z* calcd $C_{13}H_{14}NOS$ [M-H]$^-$ = 232.0979, found = 232.0974.

5-hexyl-2-phenylthiazol-4(5*H*)-one (5g)

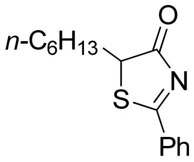

5g

A yellow solid; 66% yield; ^{1}H NMR (500 MHz, d_6-DMSO) δ 10.31 (bs, 1H), 7.80 (d, *J* = 7.6 Hz, 2H), 7.46-7.38 (m, 3H), 2.62 (t, *J* = 7.6 Hz, 2H), 1.56-1.50 (m, 2H), 1.32-1.21 (m, 6H), 0.84 (t, *J* = 6.9 Hz, 3H); ^{13}C NMR (125 MHz, d_6-DMSO) δ 158.91, 158.87, 133.95, 129.85, 129.54, 125.24, 108.87, 31.44, 31.16, 28.61, 24.26, 22.52, 14.35; HRMS (ESI) *m/z* calcd $C_{15}H_{18}NOS$ [M-H]$^-$ = 260.1115, found = 260.1103.

5-cyclohexyl-2-phenylthiazol-4(5*H*)-one (5h)

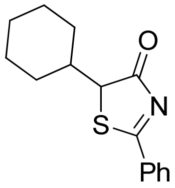

5h

A yellow solid; 57% yield; ^{1}H NMR (500 MHz, d_6-DMSO) δ 10.35 (bs, 1H), 7.79 (d, *J* = 7.6 Hz, 2H), 7.47-7.39 (m, 3H), 2.85-2.81 (m, 1H), 1.92-1.89 (m, 2H), 1.76-1.74 (m, 2H), 1.68-1.65 (m, 1H), 1.36-1.19 (m, 5H); ^{13}C NMR (125 MHz, d_6-DMSO) δ 158.50, 157.71, 133.99, 129.86, 129.56, 125.24, 115.22, 34.95, 34.68, 26.49, 25.84; HRMS (ESI) *m/z* calcd $C_{15}H_{16}NOS$ [M-H]$^-$ = 258.0958, found = 258.0964.

5-(2-(methylthio)ethyl)-2-phenylthiazol-4(5*H*)-one (5i)

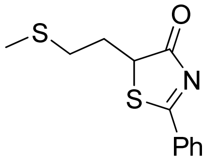

5i

A yellow solid; 42% yield; ^{1}H NMR (500 MHz, d_6-DMSO) δ 10.48 (bs, 1H), 7.81 (d, *J* = 6.9 Hz, 2H), 7.49-7.43 (m, 3H), 2.92 (t, *J* = 7.0 Hz, 2H), 2.70 (t, *J* = 6.9 Hz, 2H), 2.10 (s, 3H); ^{13}C NMR (125 MHz, d_6-DMSO) δ 157.50, 156.97, 133.92, 129.55, 128.69, 125.17, 117.78, 32.25, 30.74, 26.94; HRMS (ESI) *m/z* calcd $C_{12}H_{12}NOS_2$ [M-H]$^-$ = 250.0360, found = 250.0361.

5-decyl-2-phenylthiazol-4(5*H*)-one (5j)

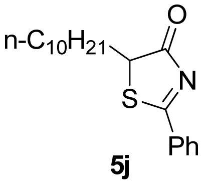

5j

A yellow foam; 38% yield; ^{1}H NMR (500 MHz, d_6-DMSO) δ 10.32 (bs, 1H), 7.79 (d, *J* = 7.0 Hz, 2H), 7.47-7.41 (m, 3H), 2.63 (t, *J* = 7.0 Hz, 2H), 1.57-1.51 (m, 5H), 1.23 (bs, 14H), 0.84 (t, *J* = 6.3 Hz, 3H); ^{13}C NMR (125 MHz, d_6-DMSO) δ 158.90, 158.85, 133.93, 129.88, 129.56, 125.24, 108.82, 65.34, 48.27, 31.79, 31.16, 29.45, 29.19, 28.89, 24.23, 22.57, 15.62, 14.39; HRMS (ESI) *m/z* calcd $C_{19}H_{26}NOS$ [M-H]$^-$ = 316.1741, found = 316.1739.

7. Preparation of Substituted 5*H*-Oxazol-4-ones

General procedure for preparing 5*H*-oxazol-4-ones **8** and **12**:[4]

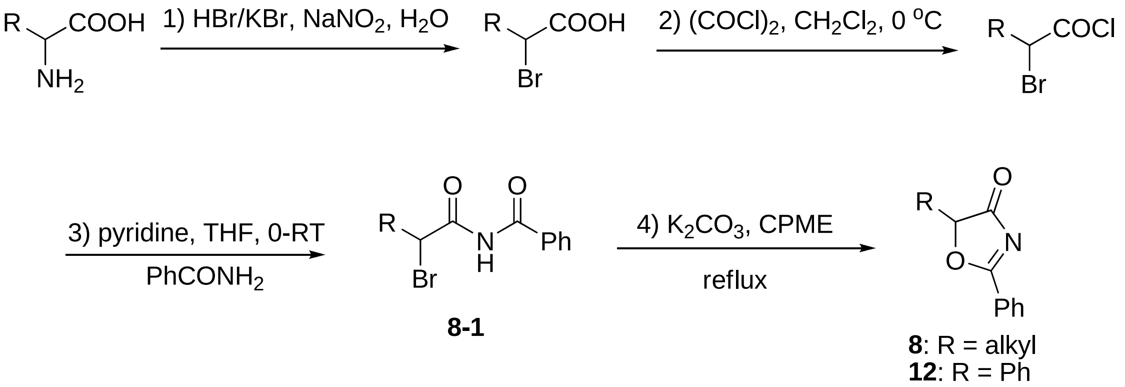

All substituted 5*H*-oxazol-4-ones were prepared from compounds **8-1** via known cyclization reaction in good yields, and the intermediates **8-1** were prepared following literature procedure.[4a] Substrates **8a-f**, **8k** and **8l**[4c] are known compounds, and their characterization data were in agreement with those reported in the literature. Unknown compounds **8g-j** were fully characterized.

5-*tert*-butyl-2-phenyloxazol-4(5H)-one (8g)

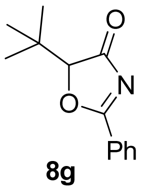

8g

A white solid; 70% yield; ^{1}H NMR (500 MHz, $CDCl_3$) δ 8.23 (d, *J* = 7.6 Hz, 2H), 7.70 (t, *J* = 7.6 Hz, 1H), 7.54 (t, *J* = 7.6 Hz, 2H), 4.43 (s, 1H), 1.13 (s, 9H); ^{13}C NMR (125 MHz, $CDCl_3$) δ 190.32, 186.05, 135.08, 129.96, 128.95, 125.70, 88.42, 35.19, 25.00; HRMS (ESI) *m/z* calcd for $C_{13}H_{15}NNaO_2$ $[M+Na]^+$ = 240.0995, found = 240.1007.

5-hexyl-2-phenyloxazol-4(5H)-one (8h)

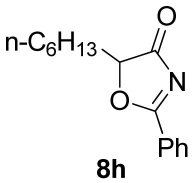

8h

A white solid; 82% yield; ^{1}H NMR (500 MHz, $CDCl_3$) δ 8.18 (d, J = 6.9 Hz, 2H), 7.67-7.64 (m, 1H), 7.51-7.49 (m, 2H), 4.76-4.73 (m, 1H), 2.08-2.00 (m, 1H), 1.85-1.78 (m, 1H), 1.48-1.42 (m, 2H), 1.33-1.27 (m, 2H), 1.24-1.21 (m, 4H), 0.82 (t, J = 6.3 Hz, 3H); ^{13}C NMR (125 MHz, $CDCl_3$) δ 191.78, 186.34, 135.00, 129.90, 128.79, 125.66, 81.78, 77.26, 77.00, 76.75, 31.27, 30.98, 28.58, 24.38, 22.31, 13.83; HRMS (ESI) m/z calcd for $C_{15}H_{19}NNaO_2$ $[M+Na]^+$ = 268.1308, found = 268.1319.

5-(2-(methylthio)ethyl)-2-phenyloxazol-4(5H)-one (8i)

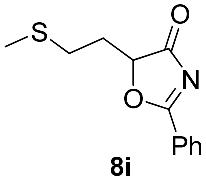

8i

A white solid; 76% yield; ^{1}H NMR (500 MHz, $CDCl_3$) δ 8.22 (d, J = 8.2 Hz, 2H), 7.70 (t, J = 7.6 Hz, 1H), 7.54 (t, J = 7.6 Hz, 2H), 5.00-4.98 (m, 1H), 2.77-2.67 (m, 2H), 2.38-2.31 (m, 1H), 2.17-2.09 (m, 4H); ^{13}C NMR (125 MHz, $CDCl_3$) δ 191.44, 186.52, 135.28, 130.11, 128.96, 125.63, 79.98, 30.58, 29.41, 15.29; HRMS (ESI) m/z calcd for $C_{12}H_{13}NNaO_2S$ $[M+Na]^+$ = 258.0559, found = 258.0572.

5-decyl-2-phenyloxazol-4(5*H*)-one (8j)

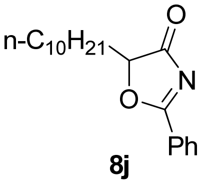

8j

A white solid; 67% yield; ^{1}H NMR (500 MHz, $CDCl_3$) δ 8.22 (dd, $J_{1,2}$ = 1.3 Hz, $J_{1,3}$ = 8.2 Hz, 2H), 7.70-7.67 (m, 1H), 7.53 (t, J = 8.2 Hz, 2H), 4.78-4.76 (m, 1H), 2.11-2.04 (m, 1H), 1.88-1.81 (m, 1H), 1.52-1.46 (m, 2H), 1.36-1.33 (m, 2H), 1.31-1.19 (m, 12H), 0.85 (t, J = 7.0 Hz, 3H); ^{13}C NMR (125 MHz, $CDCl_3$) δ 191.87, 186.46, 135.09, 130.02, 128.88, 125.78, 81.90, 31.79, 31.09, 29.45, 29.40, 29.20, 29.03, 24.56, 22.59, 14.03; HRMS (ESI) m/z calcd for $C_{19}H_{27}NNaO_2$ $[M+Na]^+$ = 324.1934, found = 324.1948.

8. Representative Procedure for the γ-Addition of 5*H*-Thiazol-4-ones

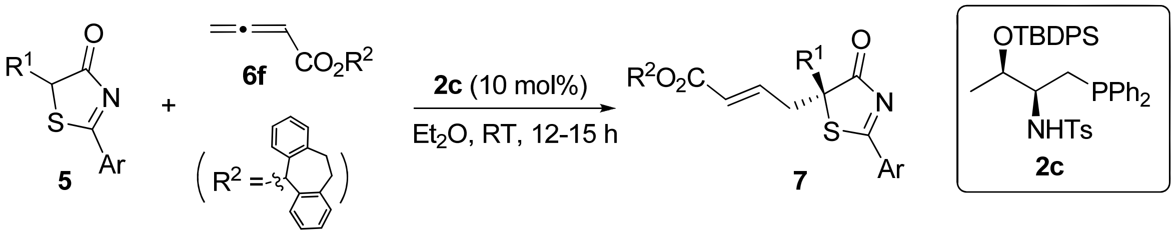

To a flame-dried round bottle flask with a magnetic stirring bar were added 5*H*-thiazol-4-one **5a** (19.1 mg, 0.10 mmol), allenoate **6f** (33.1 mg, 0.12 mmol) and catalyst **2c** (6.6 mg, 0.01 mmol), followed by addition of dry Et_2O (1.0 mL). The flask was sealed, and the reaction mixture was stirred at room temperature for overnight. The solvent of ether was removed under reduced pressure, and the residue was directly purified by column chromatography on silica gel (hexane/ethyl acetate = 15:1 to 10:1) to afford **7a** (45.2 mg, 97% yield) as a white foam.

(*R*,*E*)-10,11-dihydro-5*H*-dibenzo[a,d]cyclohepten-5-yl 4-(5-methyl-4-oxo-2-phenyl-4,5-dihydrothiazol-5-yl)but-2-enoate (7a)

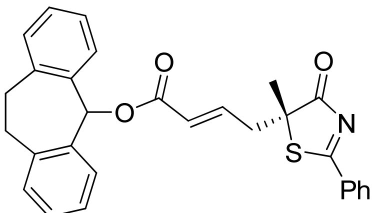

7a

A white foam; $[\alpha]^{25}_D$ = +33.9 (*c* 1.40, $CHCl_3$); ^{1}H NMR (500 MHz, $CDCl_3$) δ 8.11 (d, *J* = 8.2 Hz, 2H), 7.68 (t, *J* = 7.6 Hz, 1H), 7.52 (t, *J* = 8.2 Hz, 2H), 7.40-7.38 (m, 2H), 7.23-7.20 (m, 2H), 7.15-7.12 (m, 4H), 6.91 (s, 1H), 6.86-6.80 (m, 1H), 6.00 (d, *J* = 15.1 Hz, 1H), 3.53-3.45 (m, 2H), 3.00-2.93 (m, 2H), 2.86-2.75 (m, 2H), 1.70 (s, 3H); ^{13}C NMR (125 MHz, $CDCl_3$) δ 194.73, 194.70, 164.35, 141.82, 139.98, 136.40, 135.20, 131.98, 130.28, 130.25, 129.63, 129.02, 128.92, 128.72, 128.70, 126.32, 126.06, 79.20, 63.16, 41.93, 32.28, 32.27, 25.41; HRMS (ESI) *m/z* calcd for $C_{29}H_{25}NNaO_3S$ $[M+Na]^+$ = 490.1447, found = 490.1449; The ee value was 95%, t_R (major) = 48.0 min, t_R (minor) = 30.7 min (Chiralcel IC, λ = 254 nm, 30% *i*-PrOH/hexanes, flow rate = 1.0 mL/min).

<Chromatogram>

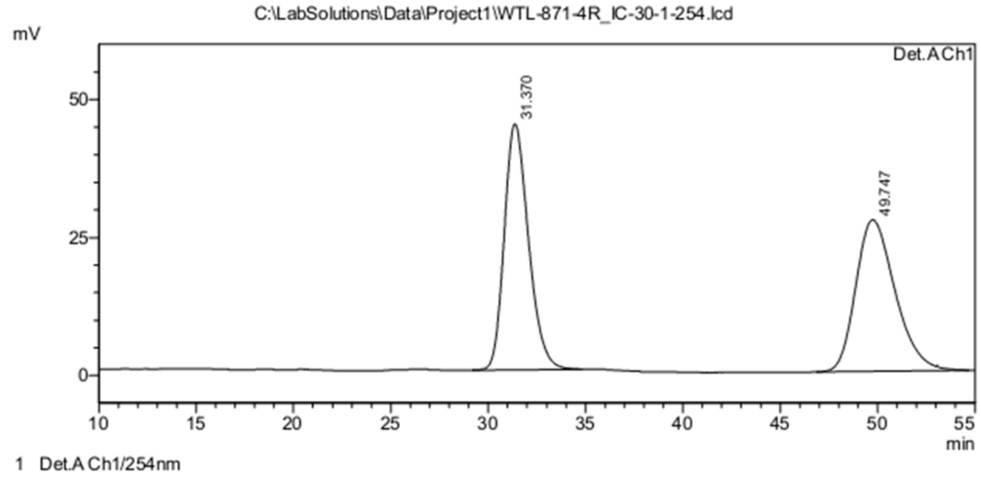

PeakTable

Detector A Ch1 254nm

Peak#	Ret. Time	Area	Height	Area %	Height %
1	31.370	3865420	44612	50.040	61.890
2	49.747	3859294	27471	49.960	38.110
Total		7724713	72084	100.000	100.000

Racemic **7a**

<Chromatogram>

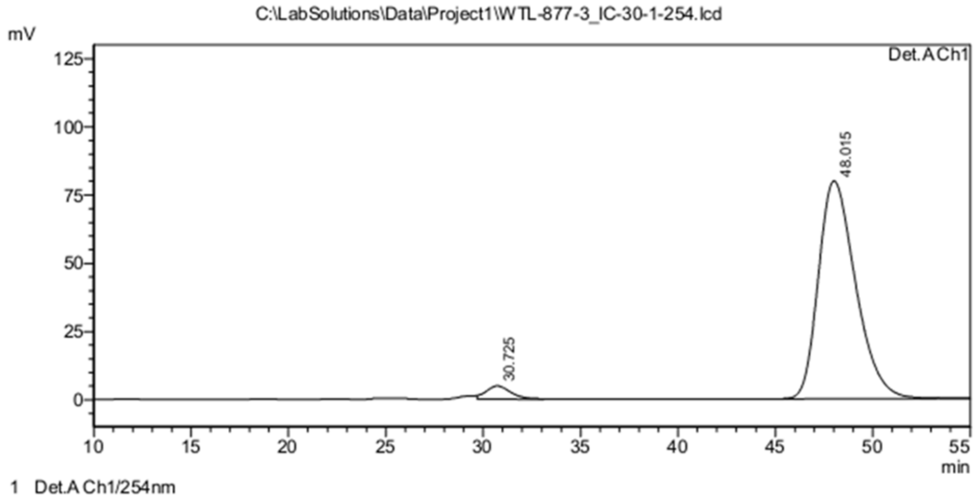

PeakTable

Detector A Ch1 254nm

Peak#	Ret. Time	Area	Height	Area %	Height %
1	30.725	271843	3803	2.495	4.547
2	48.015	10625041	79822	97.505	95.453
Total		10896883	83625	100.000	100.000

Enantiomerically enriched **7a**

(*R*,*E*)-10,11-dihydro-5*H*-dibenzo[a,d]cyclohepten-5-yl 4-(5-ethyl-4-oxo-2-phenyl-4,5-dihydrothiazol-5-yl)but-2-enoate (7b)

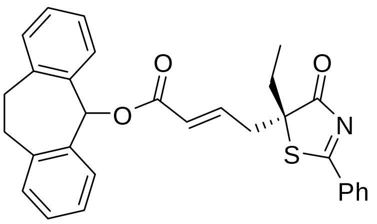

7b

A white foam; $[\alpha]^{25}_{D}$ = +37.9 (*c* 1.00, $CHCl_3$); ^{1}H NMR (500 MHz, $CDCl_3$) δ 8.16 (d, *J* = 7.6 Hz, 2H), 7.72 (t, *J* = 6.9 Hz, 1H), 7.55 (t, *J* = 7.6 Hz, 2H), 7.40 (t, *J* = 6.9 Hz, 2H), 7.23 (t, *J* = 7.0 Hz, 2H), 7.17-7.13 (m, 4H), 6.92 (s, 1H), 6.87-6.81 (m, 1H), 6.00 (d, *J* = 15.8 Hz, 1H), 3.53-3.48 (m, 2H), 2.99-2.92 (m, 2H), 2.90-2.81 (m, 2H), 2.11-2.00 (m, 2H), 1.70 (t, *J* = 6.3 Hz, 3H); ^{13}C NMR (125 MHz, $CDCl_3$) δ 195.37, 194.35, 164.37, 141.84, 139.97, 136.46, 135.85, 135.71, 135.19, 130.28, 130.25, 129.59, 129.02, 128.97, 128.69, 126.19, 126.07, 79.12, 69.36, 41.22, 32.27, 31.44, 9.18; HRMS (ESI) *m/z* calcd for $C_{30}H_{27}NNaO_3S$ $[M+Na]^+$ = 504.1604, found = 504.1619; The ee value was 94%, t_R (major) = 22.5 min, t_R (minor) = 19.0 min (Chiralcel IC, λ = 254 nm, 40% *i*-PrOH/hexanes, flow rate = 1.0 mL/min).

<Chromatogram>

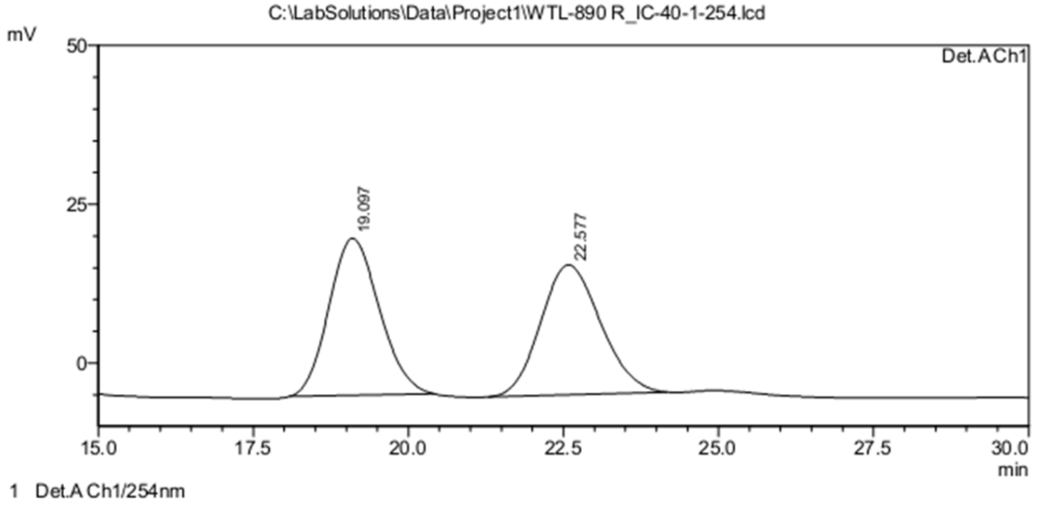

PeakTable

Detector A Ch1 254nm

Peak#	Ret. Time	Area	Height	Area %	Height %
1	19.097	1398027	24723	50.327	54.688
2	22.577	1379849	20484	49.673	45.312
Total		2777876	45207	100.000	100.000

Racemic **7b**

<Chromatogram>

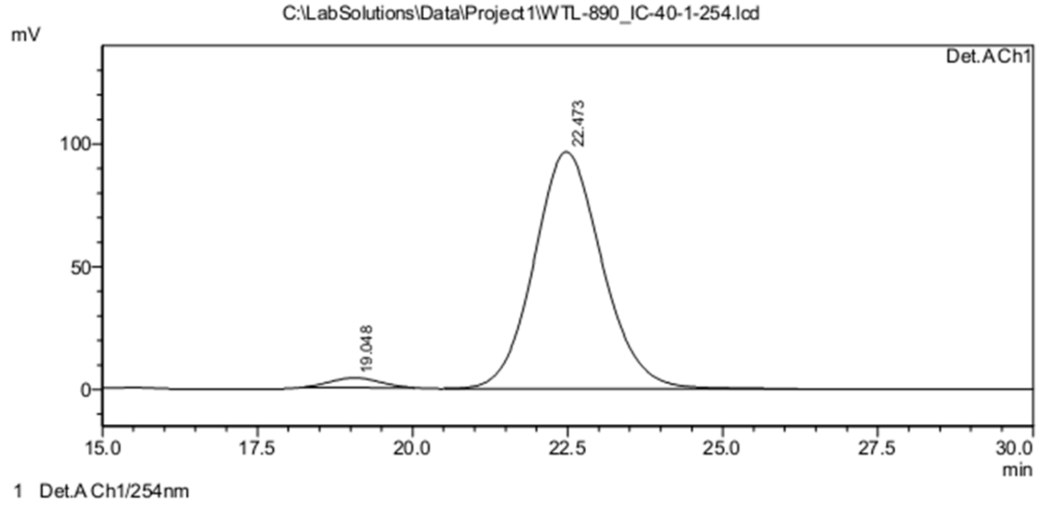

PeakTable

Detector A Ch1 254nm

Peak#	Ret. Time	Area	Height	Area %	Height %
1	19.048	218277	3967	2.874	3.946
2	22.473	7376330	96561	97.126	96.054
Total		7594607	100527	100.000	100.000

Enantiomerically enriched **7b**

(*R*,*E*)-10,11-dihydro-5*H*-dibenzo[a,d]cyclohepten-5-yl 4-(4-oxo-2-phenyl-5-propyl-4,5-dihydrothiazol-5-yl)but-2-enoate (7c)

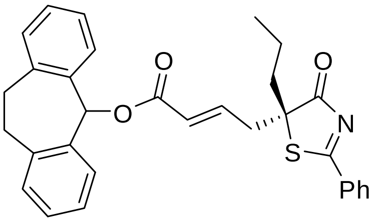

7c

A white solid; $[\alpha]^{25}_{D}$ = +39.8 (*c* 1.30, $CHCl_3$); ^{1}H NMR (500 MHz, $CDCl_3$) δ 8.12 (d, *J* = 7.6 Hz, 2H), 7.68 (t, *J* = 7.6 Hz, 1H), 7.52 (t, *J* = 8.2 Hz, 2H), 7.39-7.36 (m, 2H), 7.23-7.20 (m, 2H), 7.19-7.10 (m, 4H), 6.90 (s, 1H), 6.84-6.78 (m, 1H), 5.97 (d, *J* = 15.2 Hz, 1H), 3.51-3.44 (m, 2H), 2.98-2.84 (m, 2H), 2.80-2.76 (m, 2H), 2.01-1.90 (m, 2H), 1.46-1.38 (m, 1H), 1.24-1.18 (m, 1H), 0.90 (t, *J* = 7.6 Hz, 3H); ^{13}C NMR (125 MHz, $CDCl_3$) δ 195.27, 194.45, 164.37, 141.87, 139.97, 139.96, 136.42, 135.18, 132.02, 130.28, 130.23, 129.60, 129.01, 128.95, 128.70, 128.68, 126.18, 126.07, 126.05, 79.13, 68.57, 41.46, 40.28, 32.27, 32.24, 18.24, 13.76; HRMS (ESI) *m/z* calcd for $C_{31}H_{29}NNaO_3S$ $[M+Na]^+$ = 518.1760, found = 518.1774; The ee value was 94%, t_R (major) = 19.9 min, t_R (minor) = 15.5 min (Chiralcel IC, λ = 254 nm, 40% *i*-PrOH/hexanes, flow rate = 1.0 mL/min).

<Chromatogram>

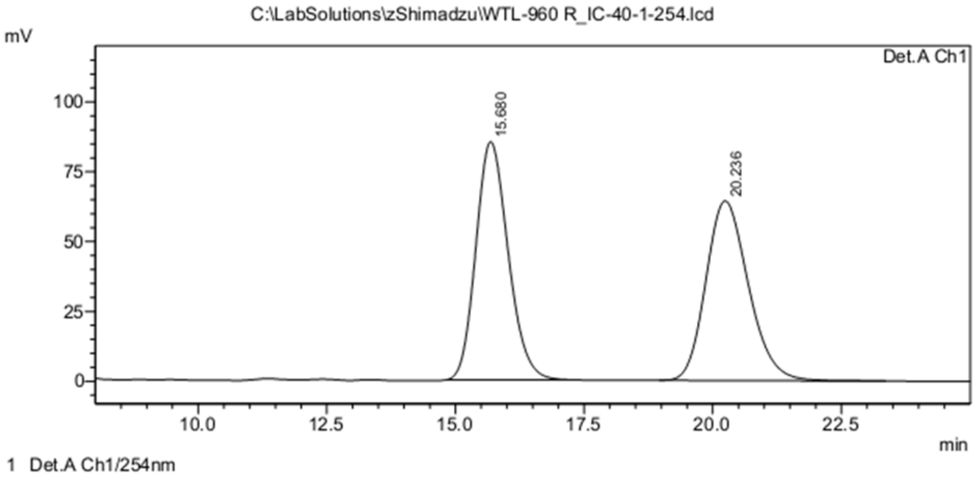

PeakTable

Detector A Ch1 254nm

Peak#	Ret. Time	Area	Height	Area %	Height %
1	15.680	3725616	85237	50.177	57.005
2	20.236	3699381	64289	49.823	42.995
Total		7424997	149526	100.000	100.000

Racemic **7c**

<Chromatogram>

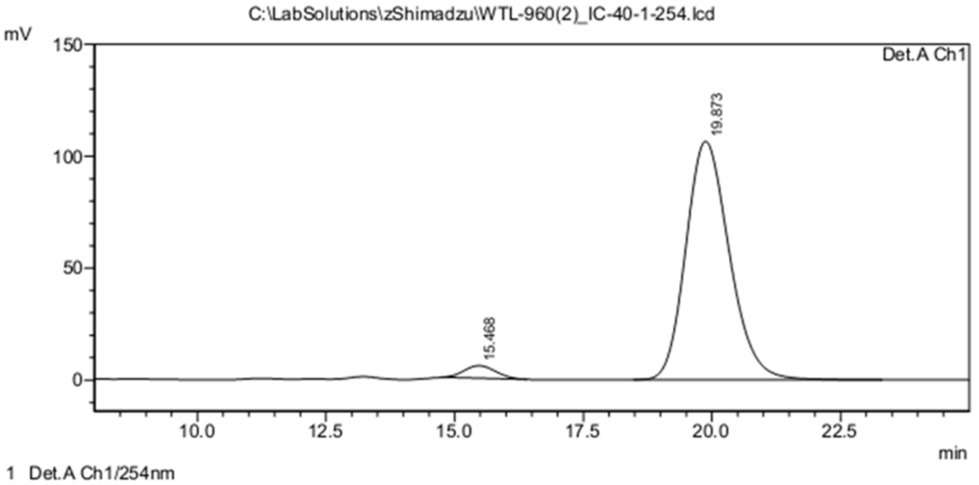

PeakTable

Detector A Ch1 254nm

Peak#	Ret. Time	Area	Height	Area %	Height %
1	15.468	187446	4901	2.906	4.399
2	19.873	6263822	106510	97.094	95.601
Total		6451268	111411	100.000	100.000

Enantiomerically enriched **7c**

(*R*,*E*)-10,11-dihydro-5*H*-dibenzo[a,d]cyclohepten-5-yl 4-(5-isopropyl-4-oxo-2-phenyl-4,5-dihydrothiazol-5-yl)but-2-enoate (7d)

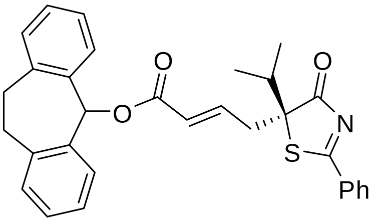

7d

A white solid; $[\alpha]^{25}_{D}$ = +34.7 (*c* 1.50, $CHCl_3$); ^{1}H NMR (500 MHz, $CDCl_3$) δ 8.12 (d, *J* = 7.6 Hz, 2H), 7.69 (t, *J* = 7.6 Hz, 1H), 7.52 (t, *J* = 7.6 Hz, 2H), 7.33 (t, *J* = 7.0 Hz, 2H), 7.20-7.16 (m, 2H), 7.10-7.06 (m, 4H), 6.85 (s, 1H), 6.78-6.72 (m, 1H), 5.96 (d, *J* = 15.1 Hz, 1H), 3.44-3.37 (m, 2H), 2.98-2.84 (m, 2H), 2.35-2.30 (m, 1H), 1.12 (d, *J* = 6.3 Hz, 3H), 0.90 (d, *J* = 6.3 Hz, 3H); ^{13}C NMR (125 MHz, $CDCl_3$) δ 195.71, 194.48, 164.25, 141.48, 139.84, 139.81, 136.51, 135.13, 132.05, 130.25, 130.18, 129.37, 128.99, 128.95, 128.60, 128.57, 126.15, 126.02, 126.00, 78.85, 74.45, 39.44, 36.32, 32.22, 32.18, 19.33, 17.76; HRMS (ESI) *m/z* calcd for $C_{31}H_{29}NNaO_3S$ $[M+Na]^+$ = 518.1760, found = 518.1769; The ee value was 92%, t_R (major) = 19.9 min, t_R (minor) = 16.4 min (Chiralcel IC, λ = 254 nm, 40% *i*-PrOH/hexanes, flow rate = 1.0 mL/min).

<Chromatogram>

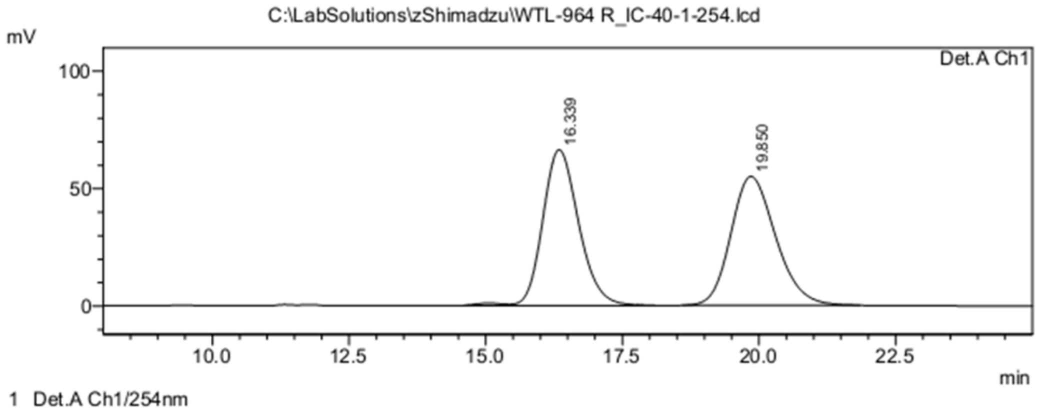

PeakTable

Detector A Ch1 254nm

Peak#	Ret. Time	Area	Height	Area %	Height %
1	16.339	3066922	66479	49.417	54.750
2	19.850	3139313	54944	50.583	45.250
Total		6206235	121422	100.000	100.000

Racemic **7d**

<Chromatogram>

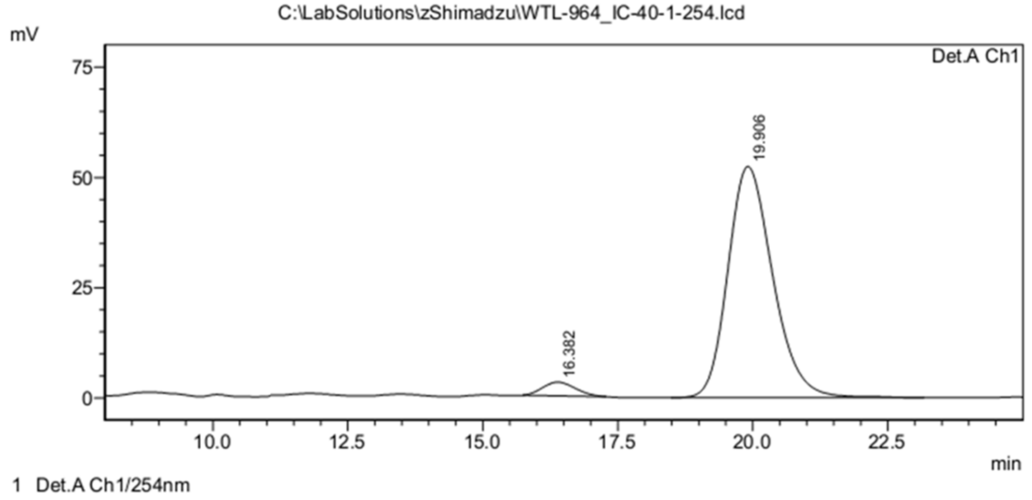

PeakTable

Detector A Ch1 254nm

Peak#	Ret. Time	Area	Height	Area %	Height %
1	16.382	131513	3103	4.156	5.591
2	19.906	3033110	52399	95.844	94.409
Total		3164623	55502	100.000	100.000

Enantiomerically enriched **7d**

(*R*,*E*)-10,11-dihydro-5*H*-dibenzo[a,d]cyclohepten-5-yl 4-(5-butyl-4-oxo-2-phenyl-4,5-dihydrothiazol-5-yl)but-2-enoate (7e)

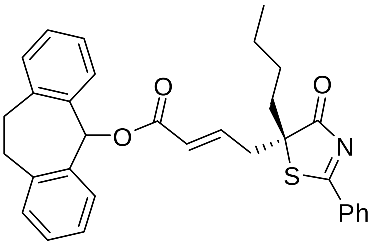

7e

A white foam; $[\alpha]^{25}_{D}$ = +40.1 (*c* 1.20, $CHCl_3$); ^{1}H NMR (500 MHz, $CDCl_3$) δ 8.13 (d, *J* = 7.0 Hz, 2H), 7.69 (t, *J* = 7.6 Hz, 1H), 7.53 (t, *J* = 7.6 Hz, 2H), 7.38-7.36 (m, 2H), 7.23-7.19 (m, 2H), 7.14-7.10 (m, 4H), 6.89 (s, 1H), 6.84-6.78 (m, 1H), 5.98 (d, *J* = 15.1 Hz, 1H), 3.51-3.45 (m, 2H), 2.98-2.91 (m, 2H), 2.89-2.77 (m, 2H), 2.03-1.92 (m, 2H), 1.37-1.34 (m, 1H), 1.32-1.26 (m, 2H), 1.19-1.12 (m, 1H), 0.85 (t, *J* = 7.0 Hz, 3H); ^{13}C NMR (125 MHz, $CDCl_3$) δ 195.29, 194.46, 164.37, 141.86, 139.96, 136.44, 135.18, 132.03, 130.28, 130.23, 129.57, 129.01, 128.96, 128.69, 128.67, 126.16, 126.06, 126.04, 79.12, 68.59, 41.48, 37.99, 32.27, 32.24, 26.92, 22.44, 13.76; HRMS (ESI) *m/z* calcd for $C_{32}H_{31}NNaO_3S$

$[M+Na]^+$ = 532.1917, found = 532.1922; The ee value was 94%, t_R (major) = 19.0 min, t_R (minor) = 14.4 min (Chiralcel IC, λ = 254 nm, 40% *i*-PrOH/hexanes, flow rate = 1.0 mL/min).

<Chromatogram>

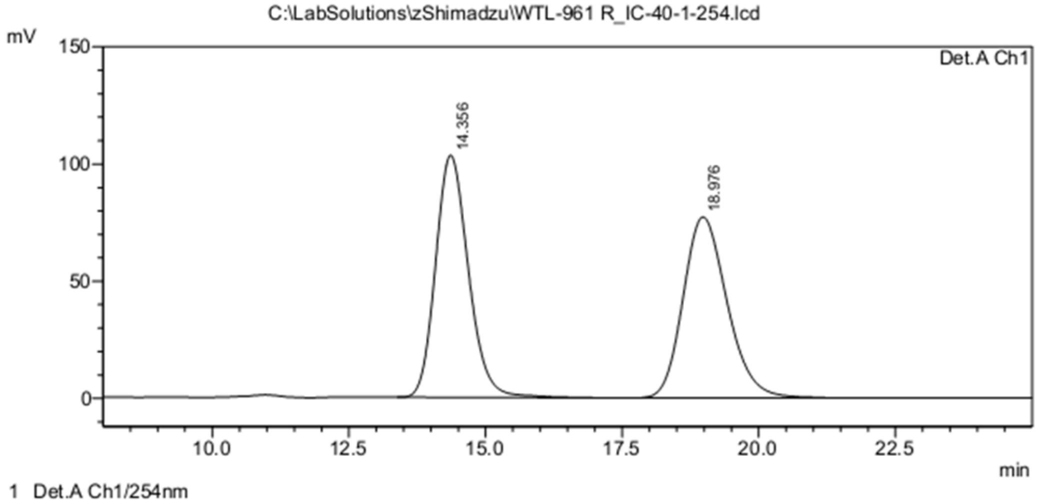

PeakTable

Detector A Ch1 254nm

Peak#	Ret. Time	Area	Height	Area %	Height %
1	14.356	4332950	103381	49.992	57.218
2	18.976	4334270	77299	50.008	42.782
Total		8667220	180680	100.000	100.000

Racemic **7e**

<Chromatogram>

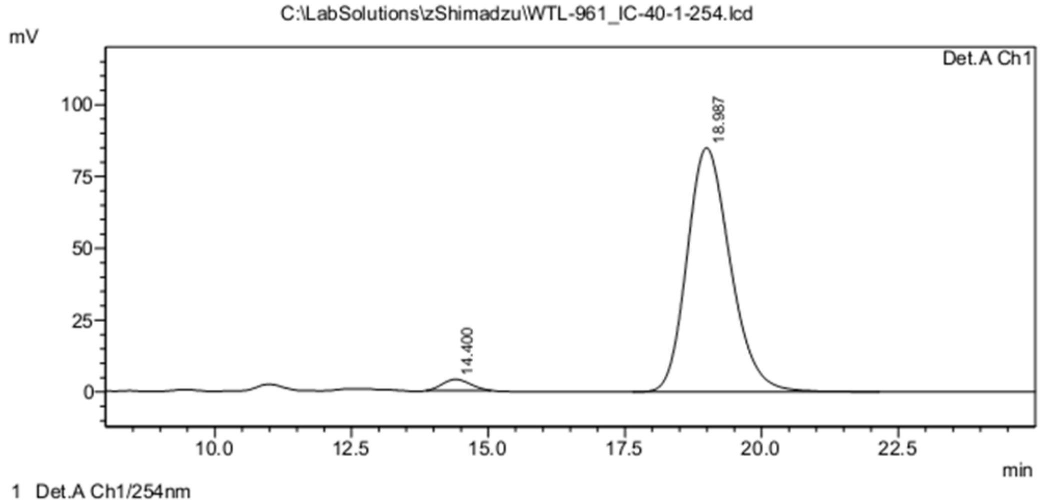

PeakTable

Detector A Ch1 254nm

Peak#	Ret. Time	Area	Height	Area %	Height %
1	14.400	135503	3835	2.809	4.321
2	18.987	4689095	84923	97.191	95.679
Total		4824597	88758	100.000	100.000

Enantiomerically enriched **7e**

(*R*,*E*)-10,11-dihydro-5*H*-dibenzo[a,d]cyclohepten-5-yl 4-(5-isobutyl-4-oxo-2-phenyl-4,5-dihydrothiazol-5-yl)but-2-enoate (7f)

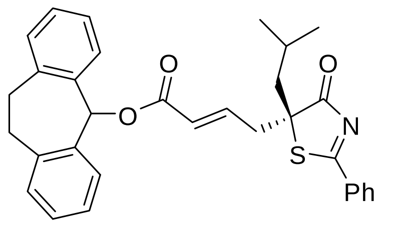

7f

A white solid; $[\alpha]^{25}_{D}$ = +34.0 (*c* 1.00, $CHCl_3$); ^{1}H NMR (500 MHz, $CDCl_3$) δ 8.13 (d, *J* = 7.0 Hz, 2H), 7.69 (t, *J* = 7.6 Hz, 1H), 7.52 (t, *J* = 7.6 Hz, 2H), 7.38-7.36 (m, 2H), 7.23-7.19 (m, 2H), 7.13-7.10 (m, 4H), 6.89 (s, 1H), 6.81-6.75 (m, 1H), 5.99 (d, *J* = 15.8 Hz, 1H), 3.50-3.43 (m, 2H), 2.97-2.90 (m, 2H), 2.83-2.73 (m, 2H), 2.07-2.03 (m, 1H), 1.97-1.93 (m, 1H), 1.76-1.71 (m, 1H), 0.94 (d, *J* = 6.3 Hz, 3H), 0.83 (d, *J* = 6.3 Hz, 3H); ^{13}C NMR (125 MHz, $CDCl_3$) δ 195.12, 194.91, 164.31, 141.62, 139.93, 136.44, 136.42, 135.13, 132.04, 130.27, 130.21, 129.53, 129.01, 128.93, 128.68, 128.64, 126.36, 126.05, 126.02, 79.08, 67.73, 46.40, 42.42, 32.25, 32.22, 26.06, 24.38, 22.80; HRMS (ESI) *m/z* calcd for $C_{32}H_{31}NNaO_3S$ $[M+Na]^+$ = 532.1917, found = 532.1921; The ee value was 88%, t_R (major) = 18.4 min, t_R (minor) = 14.8 min (Chiralcel IC, λ = 254 nm, 40% *i*-PrOH/hexanes, flow rate = 1.0 mL/min).

<Chromatogram>

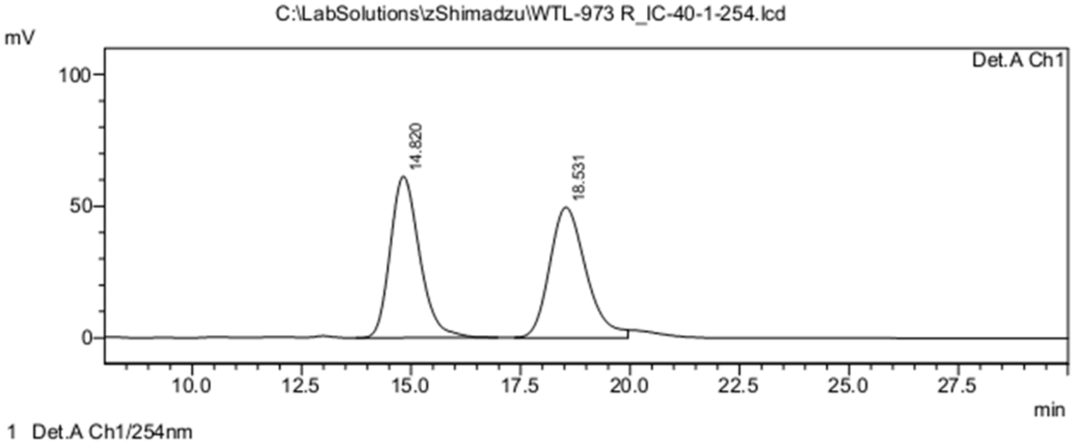

PeakTable

Detector A Ch1 254nm

Peak#	Ret. Time	Area	Height	Area %	Height %
1	14.820	2853988	61344	49.804	55.266
2	18.531	2876470	49654	50.196	44.734
Total		5730459	110998	100.000	100.000

Racemic **7f**

<Chromatogram>

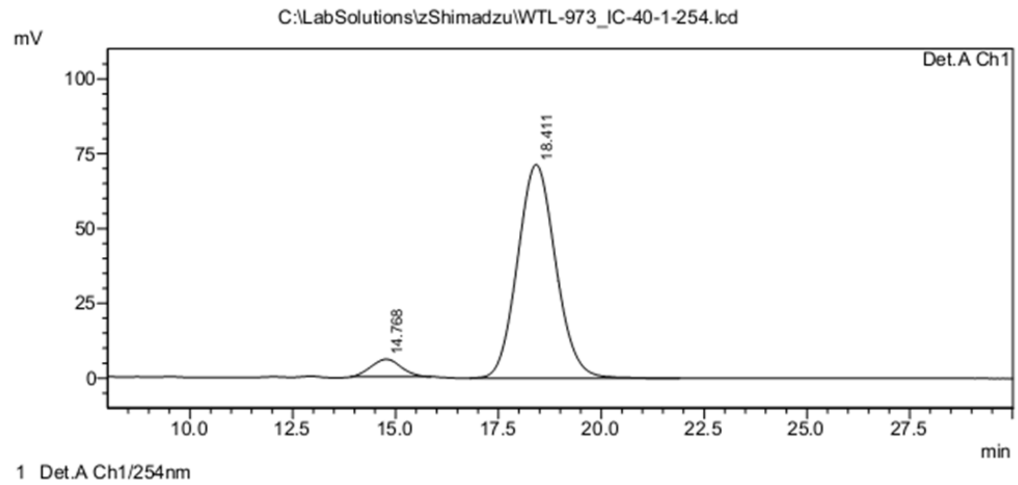

PeakTable

Detector A Ch1 254nm

Peak#	Ret. Time	Area	Height	Area %	Height %
1	14.768	298382	5850	6.079	7.579
2	18.411	4609865	71345	93.921	92.421
Total		4908247	77196	100.000	100.000

Enantiomerically enriched **7f**

(*R*,*E*)-10,11-dihydro-5*H*-dibenzo[a,d]cyclohepten-5-yl 4-(5-hexyl-4-oxo-2-phenyl-4,5-dihydrothiazol-5-yl)but-2-enoate (7g)

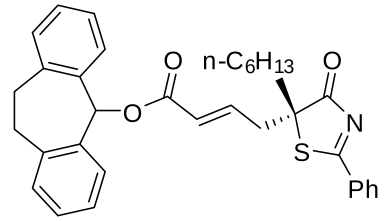

7g

A white solid; $[\alpha]^{25}_{D}$ = +39.9 (*c* 1.00, $CHCl_3$); ^{1}H NMR (500 MHz, $CDCl_3$) δ 8.13 (d, *J* = 7.0 Hz, 2H), 7.69 (t, *J* = 7.6 Hz, 1H), 7.52 (t, *J* = 8.2 Hz, 2H), 7.39-7.37 (m, 2H), 7.21-7.19 (m, 2H), 7.14-7.10 (m, 4H), 6.89 (s, 1H), 6.84-6.78 (m, 1H), 5.97 (d, *J* = 15.8 Hz, 1H), 3.51-3.44 (m, 2H), 2.98-2.90 (m, 2H), 2.88-2.76 (m, 2H), 2.02-1.92 (m, 2H), 1.40-1.34 (m, 1H), 1.30-1.16 (m, 7H), 0.84 (t, *J* = 7.0 Hz, 3H); ^{13}C NMR (125 MHz, $CDCl_3$) δ 195.28, 194.45, 164.36, 141.87, 139.96, 139.94, 136.42, 135.16, 132.03, 130.27, 130.22, 129.57, 129.01, 128.96, 128.69, 128.66, 126.16, 126.05, 126.04, 79.11, 68.62, 41.48, 38.21, 32.27, 32.24, 31.37, 28.93, 24.75, 22.41, 13.92; HRMS (ESI) *m/z* calcd for $C_{34}H_{35}NNaO_3S$ $[M+Na]^+$ = 560.2230, found = 560.2254; The ee value was 94%, t_R (major) = 17.9 min, t_R (minor) = 12.8

min (Chiralcel IC, λ = 254 nm, 40% *i*-PrOH/hexanes, flow rate = 1.0 mL/min).

<Chromatogram>

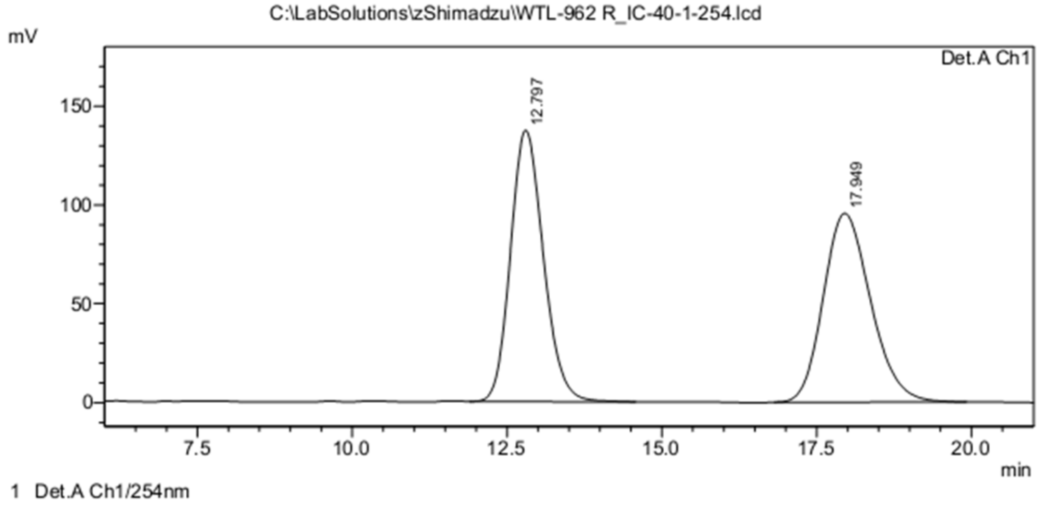

PeakTable

Detector A Ch1 254nm

Peak#	Ret. Time	Area	Height	Area %	Height %
1	12.797	5087406	137351	49.996	58.966
2	17.949	5088291	95581	50.004	41.034
Total		10175697	232932	100.000	100.000

Racemic **7g**

<Chromatogram>

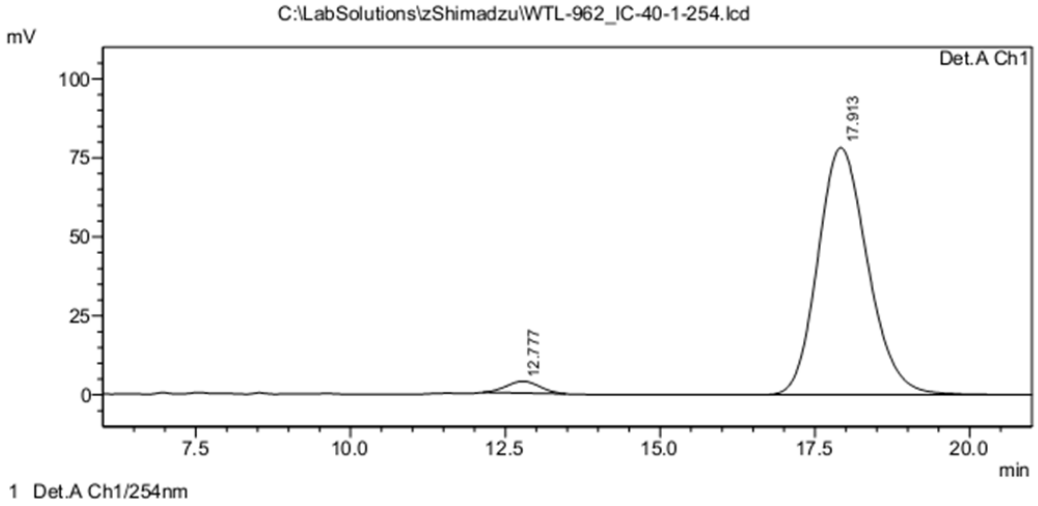

PeakTable

Detector A Ch1 254nm

Peak#	Ret. Time	Area	Height	Area %	Height %
1	12.777	138646	3701	3.072	4.517
2	17.913	4374850	78222	96.928	95.483
Total		4513496	81923	100.000	100.000

Enantiomerically enriched **7g**

(*R*,*E*)-10,11-dihydro-5*H*-dibenzo[a,d]cyclohepten-5-yl

4-(5-cyclohexyl-4-oxo-2-phenyl-4,5-dihydrothiazol-5-yl)but-2-enoate (7h)

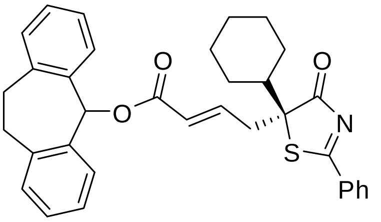

7h

A white solid; $[\alpha]^{25}_{D}$ = +28.0 (*c* 1.30, $CHCl_3$); ^{1}H NMR (500 MHz, $CDCl_3$) δ 8.12 (d, *J* = 7.6 Hz, 2H), 7.69 (t, *J* = 7.6 Hz, 1H), 7.52 (t, *J* = 8.2 Hz, 2H), 7.33 (t, *J* = 7.0 Hz, 2H), 7.20-7.16 (m, 2H), 7.10-7.05 (m, 4H), 6.85 (s, 1H), 6.77-6.71 (m, 1H), 5.97 (d, *J* = 15.8 Hz, 1H), 3.44-3.36 (m, 2H), 3.01-2.97 (m, 1H), 2.93-2.81 (m, 3H), 2.05-1.97 (m, 2H), 1.82-1.80 (m, 1H), 1.68-1.66 (m, 2H), 1.62-1.50 (m, 1H), 1.30-1.14 (m, 2H), 1.12-1.03 (m, 2H), 0.92-0.84 (m, 1H); ^{13}C NMR (125 MHz, $CDCl_3$) δ 195.68, 194.64, 164.26, 141.57, 139.82, 139.80, 136.52, 136.50, 135.08, 132.05, 130.24, 130.17, 129.36, 128.97, 128.94, 128.59, 128.55, 126.13, 126.01, 125.99, 78.83, 73.86, 45.86, 38.85, 32.21, 32.17, 29.42, 27.49, 26.24, 25.88, 25.59; HRMS (ESI) *m/z* calcd for $C_{34}H_{33}NNaO_3S$ $[M+Na]^+$ = 558.2073, found = 558.2079; The ee value was 93%, t_R (major) = 19.6 min, t_R (minor) = 15.3 min (Chiralcel IC, λ = 254 nm, 40% *i*-PrOH/hexanes, flow rate = 1.0 mL/min).

<Chromatogram>

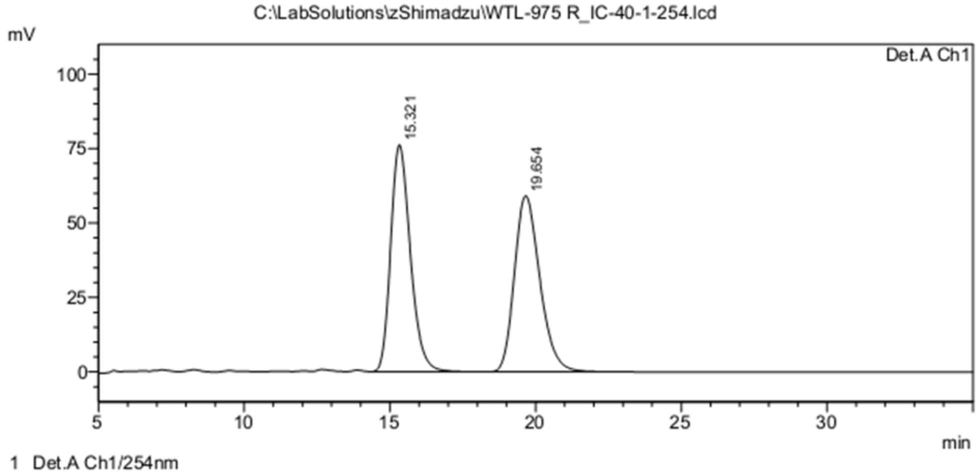

PeakTable

Detector A Ch1 254nm

Peak#	Ret. Time	Area	Height	Area %	Height %
1	15.321	3586135	76282	49.948	56.335
2	19.654	3593624	59126	50.052	43.665
Total		7179759	135409	100.000	100.000

Racemic **7h**

<Chromatogram>

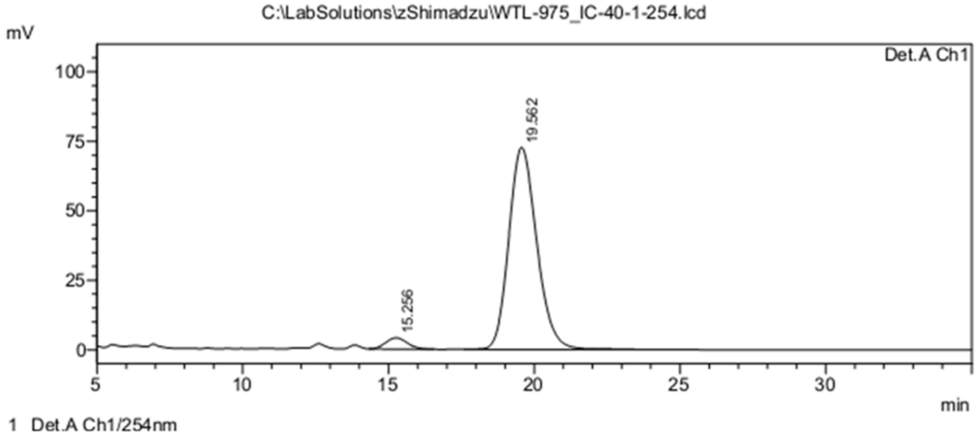

PeakTable

Detector A Ch1 254nm

Peak#	Ret. Time	Area	Height	Area %	Height %
1	15.256	188487	3857	3.735	5.037
2	19.562	4857478	72709	96.265	94.963
Total		5045965	76565	100.000	100.000

Enantiomerically enriched **7h**

(*R*,*E*)-10,11-dihydro-5*H*-dibenzo[a,d]cyclohepten-5-yl 4-(5-(2-(methylthio)ethyl)-4-oxo-2-phenyl-4,5-dihydrothiazol-5-yl)but-2-enoate (7i)

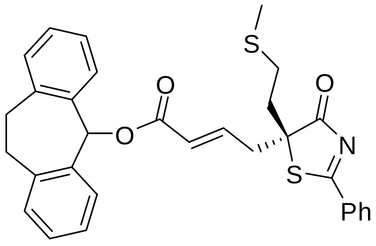

7i

A white solid; $[\alpha]^{25}_{D}$ = -37.6 (*c* 1.40, $CHCl_3$); ^{1}H NMR (500 MHz, $CDCl_3$) δ 8.12 (d, *J* = 7.6 Hz, 2H), 7.70 (t, *J* = 7.6 Hz, 1H), 7.53 (t, *J* = 7.6 Hz, 2H), 7.38-7.36 (m, 2H), 7.23-7.19 (m, 2H), 7.14-7.10 (m, 4H), 6.89 (s, 1H), 6.83-6.77 (m, 1H), 6.00 (d, *J* = 15.8 Hz, 1H), 3.50-3.45 (m, 2H), 2.97-2.93 (m, 2H), 2.91-2.76 (m, 2H), 2.56-2.49 (m, 1H), 2.39-2.33 (m, 2H), 2.27-2.23 (m, 1H), 2.06 (s, 3H); ^{13}C NMR (125 MHz, $CDCl_3$) δ 195.02, 193.79, 164.22, 141.18, 139.96, 136.36, 136.34, 135.35, 131.85, 130.27, 130.23, 129.62, 129.06, 128.99, 128.71, 128.68, 126.56, 126.05, 126.04, 79.23, 67.53, 41.52, 37.26, 32.26, 32.24, 29.44, 15.46; HRMS (ESI) *m/z* calcd for $C_{31}H_{29}NNaO_3S$ $[M+Na]^+$ = 550.1481, found = 550.1491; The ee value was 90%, t_R (major) = 37.6 min, t_R (minor) = 21.8 min (Chiralcel IC, λ = 254 nm, 40% *i*-PrOH/hexanes, flow rate = 1.0 mL/min).

<Chromatogram>

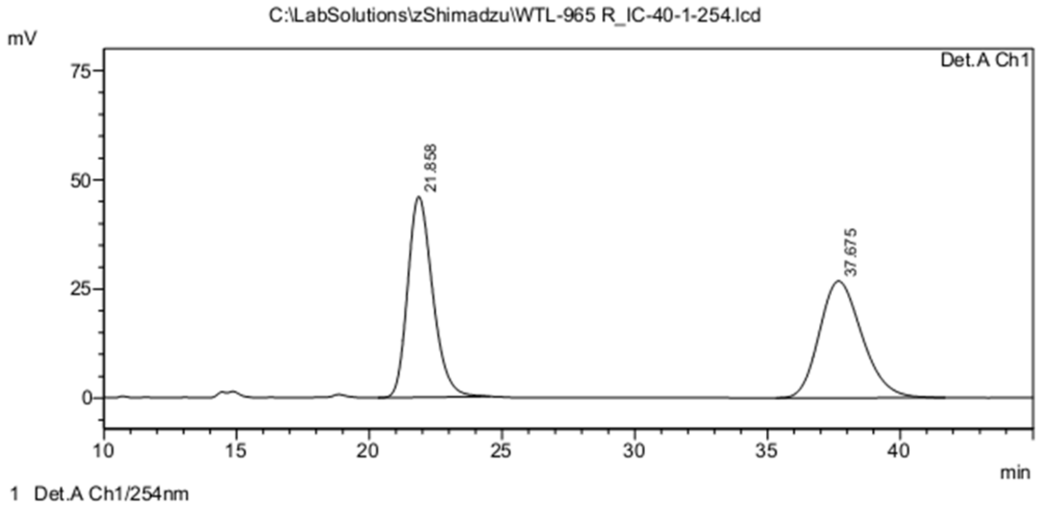

PeakTable

Detector A Ch1 254nm

Peak#	Ret. Time	Area	Height	Area %	Height %
1	21.858	3012687	45978	50.129	63.214
2	37.675	2997143	26756	49.871	36.786
Total		6009830	72734	100.000	100.000

Racemic **7i**

<Chromatogram>

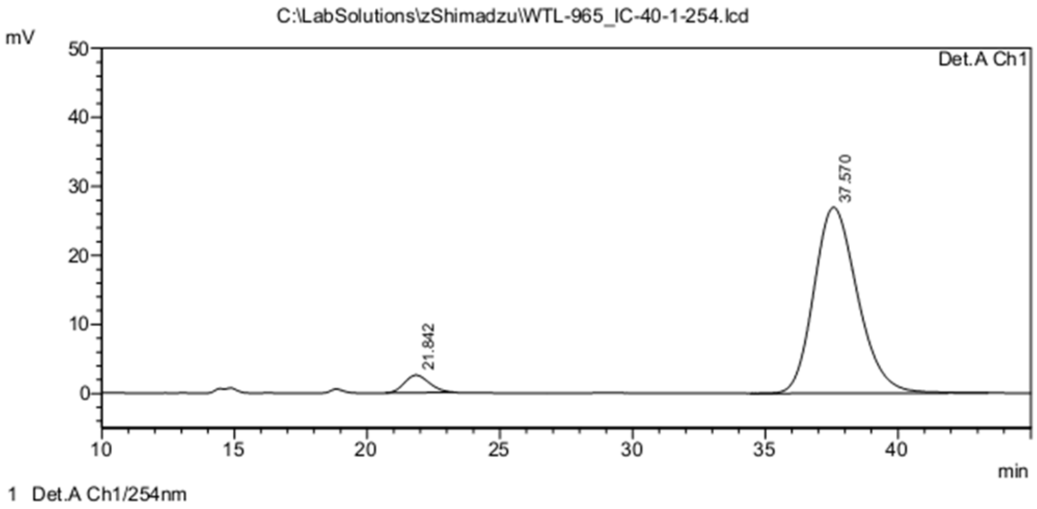

PeakTable

Detector A Ch1 254nm

Peak#	Ret. Time	Area	Height	Area %	Height %
1	21.842	165150	2531	5.089	8.590
2	37.570	3080129	26928	94.911	91.410
Total		3245279	29458	100.000	100.000

Enantiomerically enriched **7i**

(*R*,*E*)-10,11-dihydro-5*H*-dibenzo[a,d]cyclohepten-5-yl 4-(5-decyl-4-oxo-2-phenyl-4,5-dihydrothiazol-5-yl)but-2-enoate (7j)

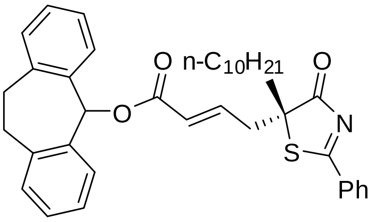

7j

A white oil-solid; $[\alpha]^{25}_{D}$ = +18.7 (*c* 0.70, $CHCl_3$); ^{1}H NMR (500 MHz, $CDCl_3$) δ 8.13 (d, *J* = 7.6 Hz, 2H), 7.69 (t, *J* = 7.6 Hz, 1H), 7.53 (t, *J* = 8.2 Hz, 2H), 7.47-7.37 (m, 2H), 7.23-7.16 (m, 2H), 7.14-7.10 (m, 4H), 6.90 (s, 1H), 6.84-6.78 (m, 1H), 5.98 (d, *J* = 15.2 Hz, 1H), 3.51-3.40 (m, 3H), 3.16-3.10 (m, 1H), 2.98-2.91 (m, 2H), 2.86-2.76 (m, 2H), 2.02-1.91 (m, 2H), 1.29-1.22 (m, 14H), 0.87 (t, *J* = 7.0 Hz, 3H); ^{13}C NMR (125 MHz, $CDCl_3$) δ 195.29, 194.47, 164.38, 141.89, 140.48, 139.95, 138.82, 136.41, 135.17, 132.02, 130.23, 129.59, 129.00, 128.67, 127.88, 126.98, 126.11, 79.12, 68.62, 41.49, 38.22, 32.34, 32.27, 32.24, 31.82, 29.45, 29.29, 29.21, 24.82, 22.61, 14.07; HRMS (ESI) *m/z* calcd for $C_{38}H_{43}NNaO_3S$ $[M+Na]^+$ = 616.2856, found = 616.2868; The ee value was 93%, t_R (major) = 15.7 min, t_R (minor) = 10.8 min (Chiralcel IC, λ = 254 nm, 40% *i*-PrOH/hexanes, flow rate = 1.0 mL/min).

<Chromatogram>

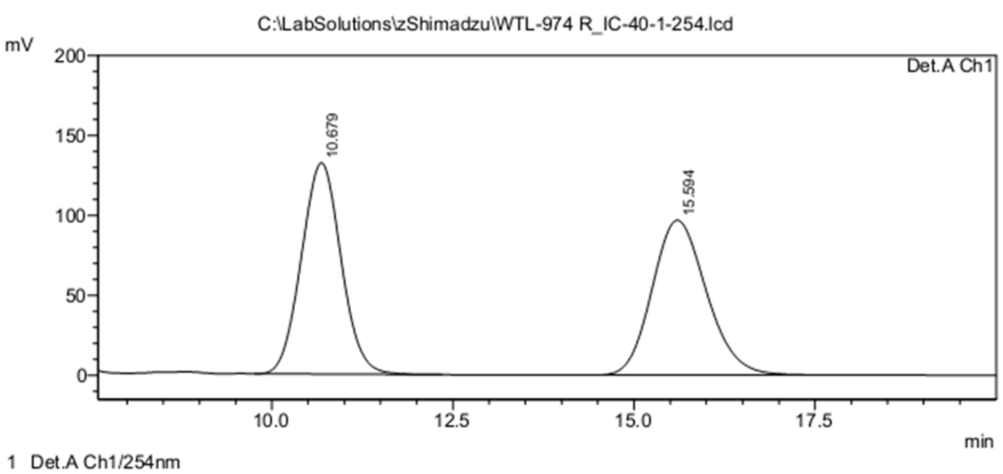

1 Det.A Ch1/254nm

PeakTable

Detector A Ch1 254nm

Peak#	Ret. Time	Area	Height	Area %	Height %
1	10.679	5051131	132167	49.942	57.718
2	15.594	5062866	96819	50.058	42.282
Total		10113997	228987	100.000	100.000

Racemic **7j**

<Chromatogram>

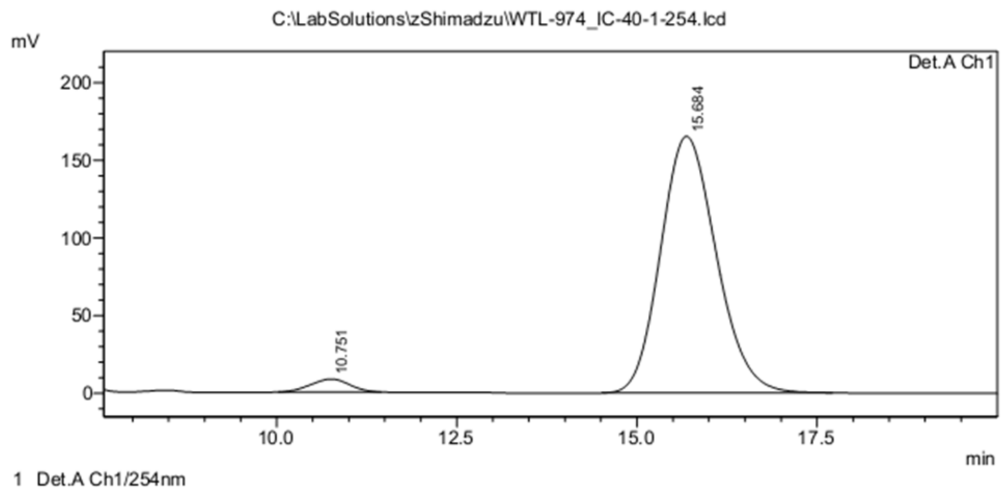

PeakTable

Detector A Ch1 254nm

Peak#	Ret. Time	Area	Height	Area %	Height %
1	10.751	313757	8333	3.408	4.804
2	15.684	8891783	165124	96.592	95.196
Total		9205541	173457	100.000	100.000

Enantiomerically enriched **7j**

(*S*,*E*)-10,11-dihydro-5*H*-dibenzo[a,d]cyclohepten-5-yl 4-(5-benzyl-4-oxo-2-phenyl-4,5-dihydrothiazol-5-yl)but-2-enoate (7k)

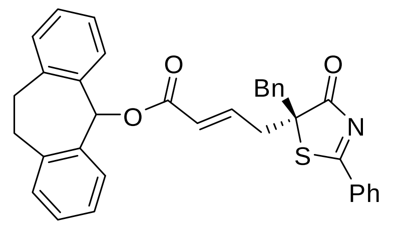

7k

A white solid; $[\alpha]^{25}_{D}$ = -25.3 (*c* 1.10, $CHCl_3$); ^{1}H NMR (500 MHz, $CDCl_3$) δ 8.00 (d, *J* = 7.6 Hz, 2H), 7.64 (t, *J* = 7.6 Hz, 1H), 7.46 (t, *J* = 7.6 Hz, 2H), 7.36-7.34 (m, 2H), 7.24-7.18 (m, 7H), 7.12-7.09 (m, 4H), 6.87 (s, 1H), 6.81-6.75 (m, 1H), 5.98 (d, *J* = 15.8 Hz, 1H), 3.48-3.42 (m, 2H), 3.32 (d, *J* = 13.3 Hz, 1H), 3.22 (d, *J* = 13.3 Hz, 1H), 2.96-2.89 (m, 3H), 2.87-2.83 (m, 1H); ^{13}C NMR (125 MHz, $CDCl_3$) δ 195.03, 194.03, 164.26, 141.39, 139.90, 136.43, 135.11, 134.71, 131.87, 130.31, 130.26, 130.21, 129.48, 128.88, 128.66, 128.63, 128.30, 127.53, 126.44, 126.04, 79.04, 69.31, 44.55, 40.67, 32.23, 32.22; HRMS (ESI) *m/z* calcd for $C_{35}H_{29}NNaO_3S$ $[M+Na]^+$ = 566.1760, found = 566.1751; The ee value was 92%, t_R (major) = 63.1 min, t_R (minor) = 53.6 min (Chiralcel IC, λ = 254 nm, 20% *i*-PrOH/hexanes,

flow rate = 1.0 mL/min).

<Chromatogram>

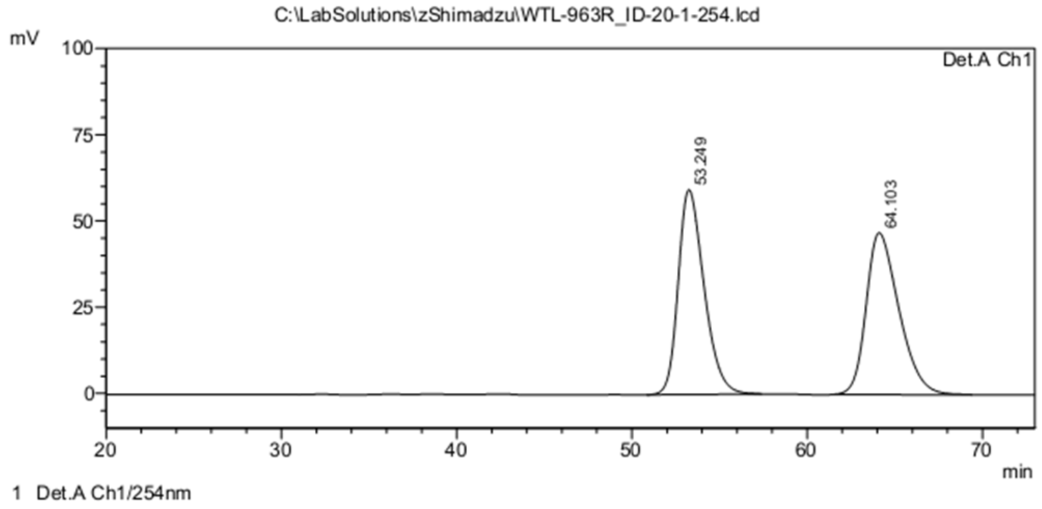

PeakTable

Detector A Ch1 254nm

Peak#	Ret. Time	Area	Height	Area %	Height %
1	53.249	6101299	59369	49.953	55.852
2	64.103	6112855	46927	50.047	44.148
Total		12214154	106296	100.000	100.000

Racemic **7k**

<Chromatogram>

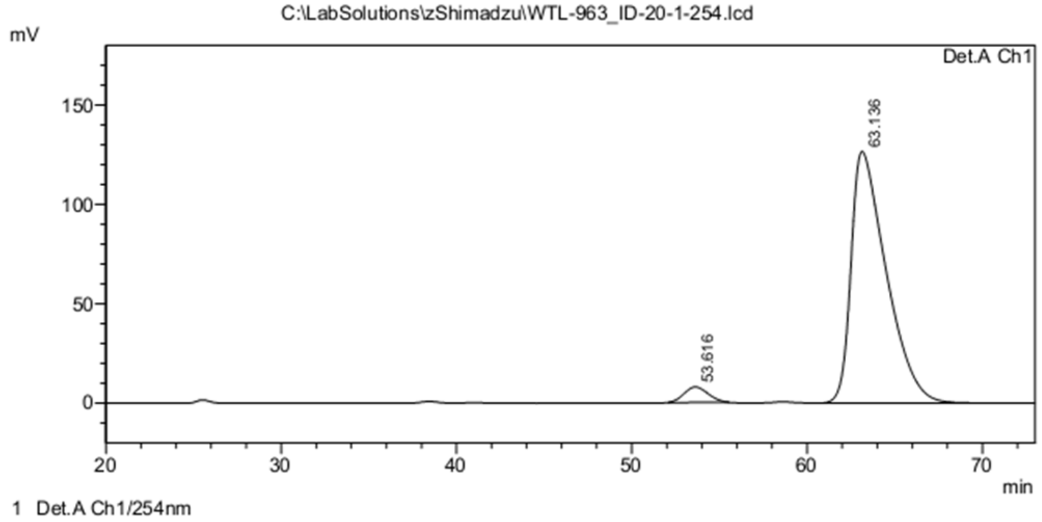

PeakTable

Detector A Ch1 254nm

Peak#	Ret. Time	Area	Height	Area %	Height %
1	53.616	729880	7711	3.955	5.735
2	63.136	17725667	126750	96.045	94.265
Total		18455547	134461	100.000	100.000

Enantiomerically enriched **7k**

(*R*,*E*)-10,11-dihydro-5*H*-dibenzo[a,d]cyclohepten-5-yl 4-(5-methyl-2-(naphthalen-2-yl)-4-oxo-4,5-dihydrothiazol-5-yl)but-2-enoate (7l)

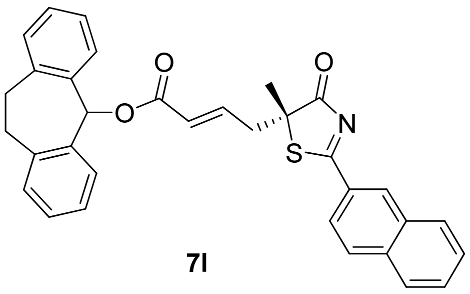

7l

A white solid; $[\alpha]^{25}_{D}$ = +12.0 (*c* 1.30, $CHCl_3$); ^1H NMR (500 MHz, $CDCl_3$) δ 8.68 (s, 1H), 8.12 (dd, $J_{1,2}$ = 1.9 Hz, $J_{1,3}$ = 8.9 Hz, 1H), 7.99-7.91 (m, 3H), 7.67 (t, *J* = 7.0 Hz, 1H), 7.61 (t, *J* = 7.0 Hz, 1H), 7.39-7.37 (m, 1H), 7.21-7.15 (m, 2H), 7.13-7.10 (m, 3H), 7.05 (d, *J* = 7.6 Hz, 1H), 6.89 (s, 1H), 6.88-6.83 (m, 1H), 6.00 (d, *J* = 15.1 Hz, 1H), 3.51-3.44 (m, 2H), 2.96-2.89 (m, 2H), 2.87-2.78 (m, 2H), 1.74 (s, 3H); ^{13}C NMR (125 MHz, $CDCl_3$) δ 194.71, 194.53, 164.39, 141.88, 140.03, 136.70, 136.39, 132.47, 130.97, 130.28, 130.23, 129.85, 129.72, 129.49, 129.33, 128.91, 128.73, 128.69, 127.96, 127.34, 126.39, 126.08, 126.06, 124.18, 79.29, 63.33, 42.06, 32.30, 32.28, 25.46; HRMS (ESI) *m/z* calcd for $C_{33}H_{27}NNaO_3S$ $[M+Na]^+$ = 540.1604, found = 540.1582; The ee value was 89%, t_R (major) = 64.2 min, t_R (minor) = 43.1 min (Chiralcel IC, λ = 254 nm, 30% *i*-PrOH/hexanes, flow rate = 1.0 mL/min).

<Chromatogram>

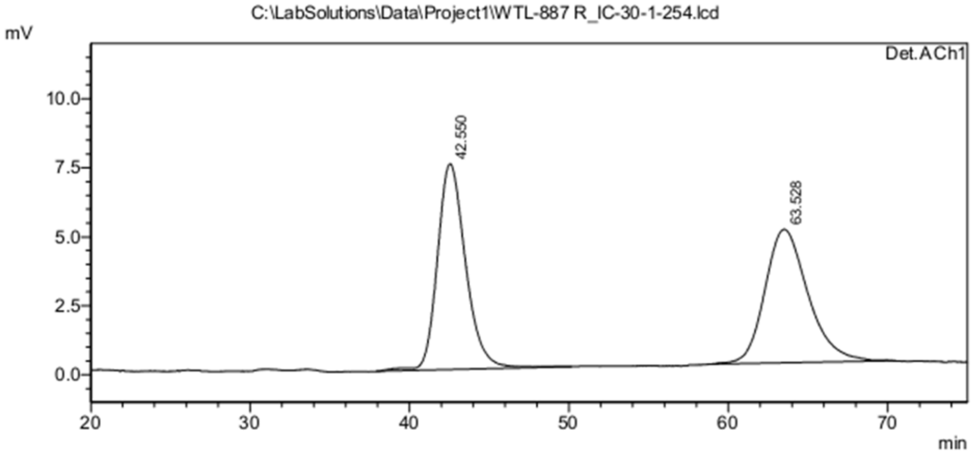

1 Det.A Ch1/254nm

PeakTable

Detector A Ch1 254nm

Peak#	Ret. Time	Area	Height	Area %	Height %
1	42.550	920609	7464	50.168	60.647
2	63.528	914451	4843	49.832	39.353
Total		1835060	12307	100.000	100.000

Racemic **7l**

<Chromatogram>

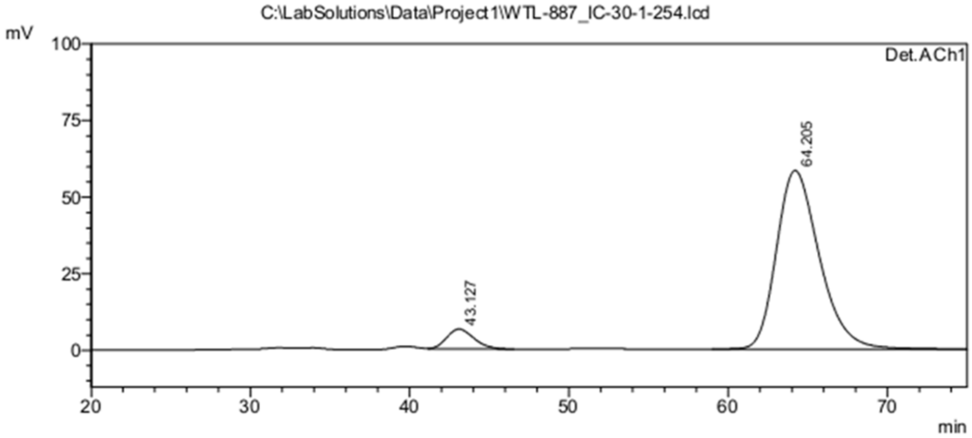

1 Det.A Ch1/254nm

PeakTable

Detector A Ch1 254nm

Peak#	Ret. Time	Area	Height	Area %	Height %
1	43.127	638389	5930	5.510	9.230
2	64.205	10947660	58316	94.490	90.770
Total		11586050	64246	100.000	100.000

Enantiomerically enriched **7l**

9. Representative Procedure for the γ-Addition of 5*H*-Oxazol-4-ones

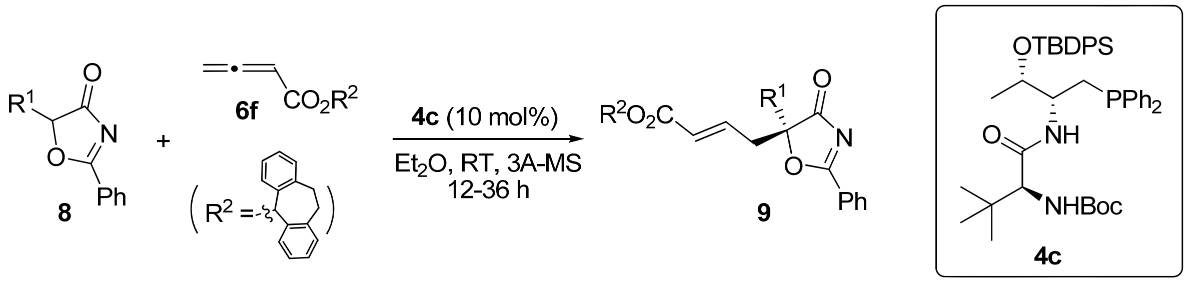

To a flame-dried round bottle flask with a magnetic stirring bar were added 5*H*-oxazol-4-one **8a** (17.5 mg, 0.10 mmol), allenoate **6f** (33.1 mg, 0.12 mmol), activated 3Å-MS (15 mg) and catalyst **4c** (7.2 mg, 0.01 mmol), followed by addition of dry Et_2O (1.0 mL). The flask was sealed, and the reaction mixture was stirred at room temperature for overnight. The molecular sieve was removed by filtration and the solvent was removed under reduced pressure. The crude addition product was directly purified by column chromatography on silica gel (hexane/ethyl acetate = 15:1 to 10:1) to afford **9a** (43.7 mg, 97% yield) as a white solid.

(*S*,*E*)-10,11-dihydro-5*H*-dibenzo[a,d]cyclohepten-5-yl 4-(5-methyl-4-oxo-2-phenyl-4,5-dihydrooxazol-5-yl)but-2-enoate (9a)

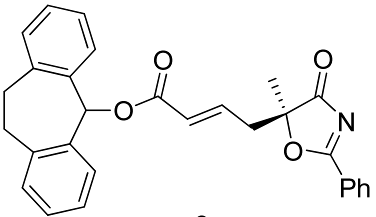

9a

A white solid; $[\alpha]^{25}_{D}$ = +29.8 (*c* 1.10, $CHCl_3$); ^{1}H NMR (500 MHz, $CDCl_3$) δ 8.19 (d, *J* = 7.6 Hz, 2H), 7.71 (t, *J* = 6.9 Hz, 1H), 7.53 (t, *J* = 7.6 Hz, 2H), 7.37 (d, *J* = 7.6 Hz, 2H), 7.22 (t, *J* = 7.4 Hz, 2H) 7.15-7.12 (m, 4H), 6.90 (s, 1H), 6.87-6.81 (m, 1H), 6.01 (d, *J* = 15.8 Hz, 1H), 3.52-3.45 (m, 2H), 2.99-2.94 (m, 2H), 2.80-2.69 (m, 2H), 1.59 (s, 3H); ^{13}C NMR (125 MHz, $CDCl_3$) δ 192.81, 185.20, 164.23, 139.95, 139.66, 136.40, 135.33, 130.27, 130.16, 129.57, 128.96, 128.77, 126.77, 126.08, 125.58, 86.31, 79.19, 38.99, 32.28, 21.52; HRMS (ESI) *m/z* calcd for $C_{29}H_{25}NNaO_4$ $[M+Na]^+$ = 474.1676, found = 474.1677; The ee value was 92%, t_R (major) = 33.3 min, t_R (minor) = 24.6 min (Chiralcel IC, λ = 254 nm, 40% *i*-PrOH/hexanes, flow rate = 1.0 mL/min).

<Chromatogram>

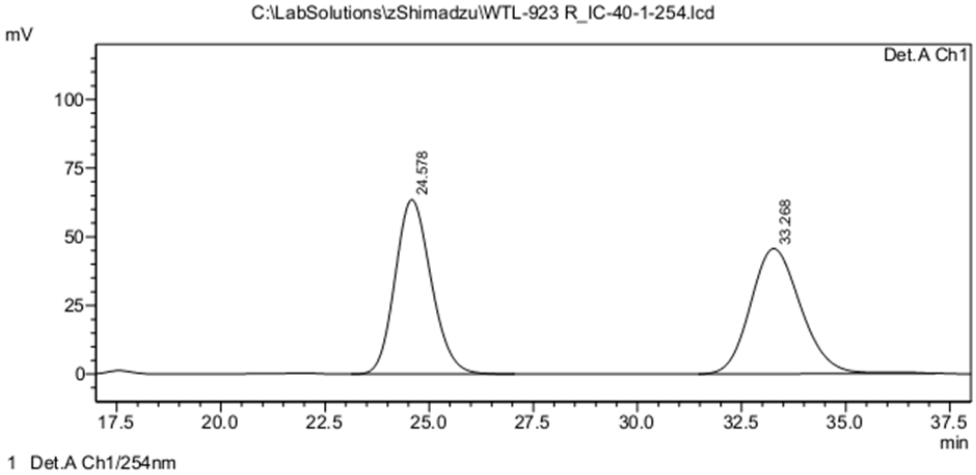

PeakTable

Detector A Ch1 254nm

Peak#	Ret. Time	Area	Height	Area %	Height %
1	24.578	3825971	63451	49.948	58.200
2	33.268	3834006	45571	50.052	41.800
Total		7659977	109022	100.000	100.000

Racemic **9a**

<Chromatogram>

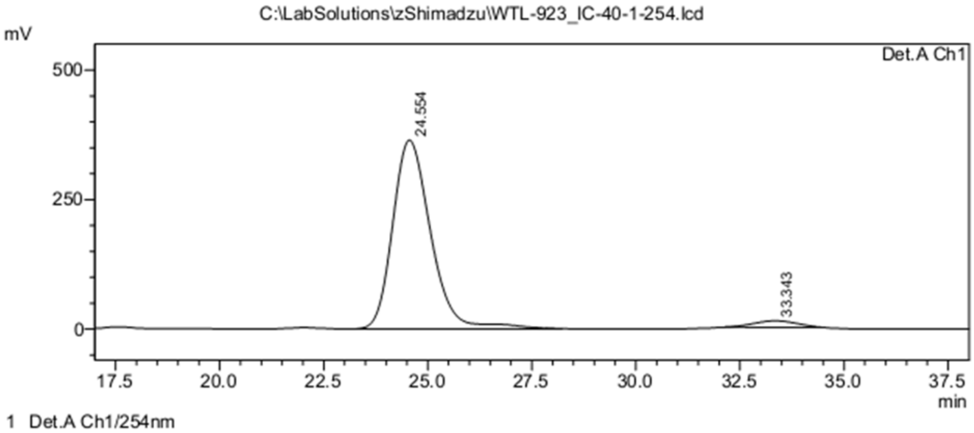

PeakTable

Detector A Ch1 254nm

Peak#	Ret. Time	Area	Height	Area %	Height %
1	24.554	23213336	364374	96.189	96.677
2	33.343	919623	12524	3.811	3.323
Total		24132959	376899	100.000	100.000

Enantiomerically enriched **9a**

(*S*,*E*)-10,11-dihydro-5*H*-dibenzo[a,d]cyclohepten-5-yl 4-(5-ethyl-4-oxo-2-phenyl-4,5-dihydrooxazol-5-yl)but-2-enoate (9b)

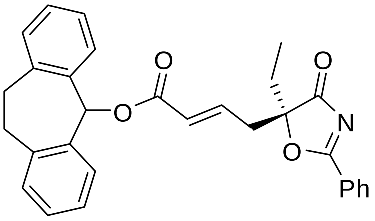

9b

A white solid; $[\alpha]^{25}_{D}$ = +36.6 (*c* 1.60, $CHCl_3$); ^{1}H NMR (500 MHz, $CDCl_3$) δ 8.20 (d, *J* = 7.6 Hz, 2H), 7.71 (t, *J* = 7.6 Hz, 1H), 7.54 (t, *J* = 7.6 Hz, 2H), 7.36 (d, *J* = 8.2 Hz, 2H), 7.22 (t, *J* = 7.6 Hz, 2H) 7.14-7.12 (m, 4H), 6.89 (s, 1H), 6.84-6.78 (m, 1H), 6.00 (d, *J* = 15.8 Hz, 1H), 3.50-3.44 (m, 2H), 2.98-2.94 (m, 2H), 2.82-2.70 (m, 2H), 2.03-1.94 (m, 2H), 0.86 (t, *J* = 7.6 Hz, 3H); ^{13}C NMR (125 MHz, $CDCl_3$) δ 192.44, 185.70, 164.23, 139.93, 139.69, 136.44, 136.42, 135.33, 130.26, 130.14, 129.54, 128.99, 128.70, 126.63, 126.08, 125.45, 89.83, 79.13, 38.10, 32.27, 28.62, 7.17; HRMS (ESI) *m/z* calcd for $C_{30}H_{27}NNaO_4$ $[M+Na]^+$ = 488.1832, found = 488.1837; The ee value was 93%, t_R (major) = 31.2 min, t_R (minor) = 36.3 min (Chiralcel IB, λ = 254 nm, 5% *i*-PrOH/hexanes, flow rate = 1.0 mL/min).

<Chromatogram>

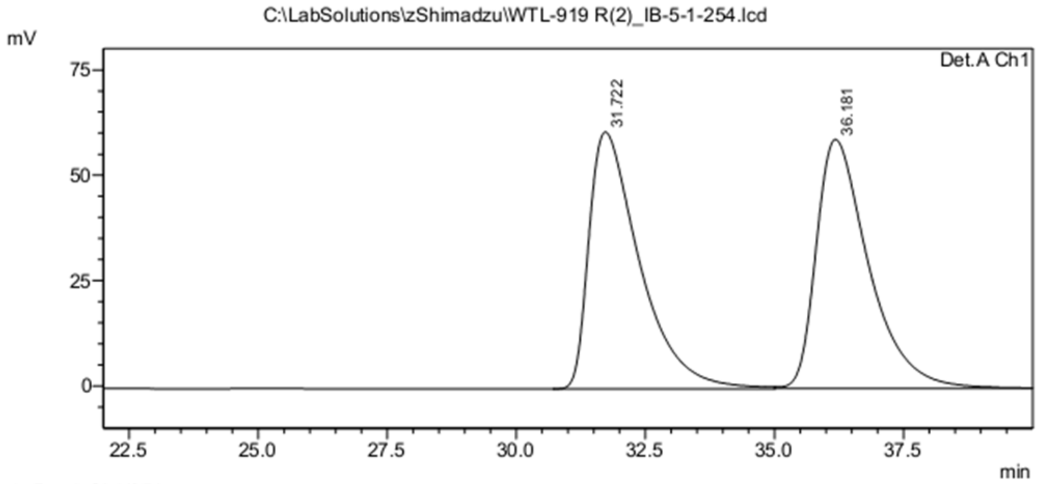

1 Det.A Ch1/254nm

PeakTable

Detector A Ch1 254nm

Peak#	Ret. Time	Area	Height	Area %	Height %
1	31.722	4161985	60916	49.821	50.741
2	36.181	4191933	59136	50.179	49.259
Total		8353918	120051	100.000	100.000

Racemic **9b**

<Chromatogram>

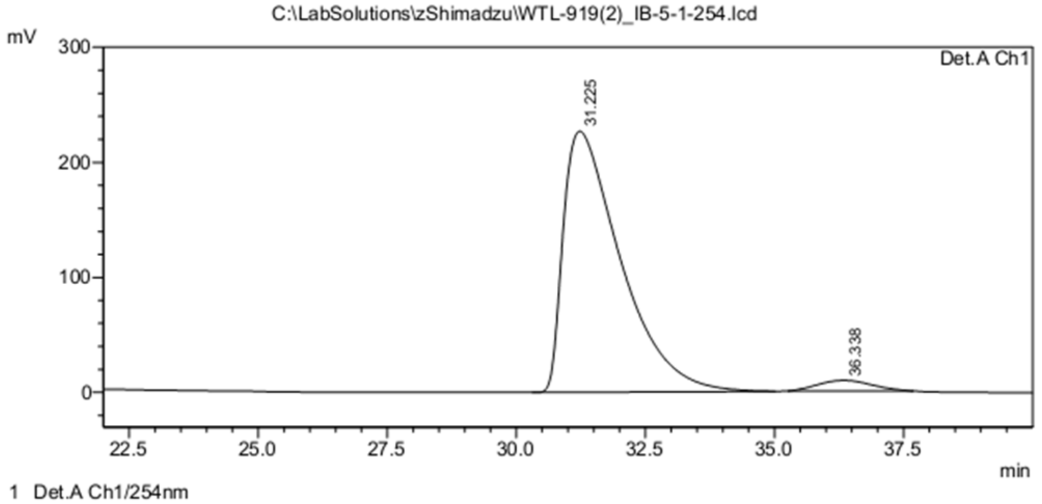

PeakTable

Detector A Ch1 254nm

Peak#	Ret. Time	Area	Height	Area %	Height %
1	31.225	17579531	226923	96.339	96.097
2	36.338	668100	9218	3.661	3.903
Total		18247631	236140	100.000	100.000

Enantiomerically enriched **9b**

(*S*,*E*)-10,11-dihydro-5*H*-dibenzo[a,d]cyclohepten-5-yl 4-(4-oxo-2-phenyl-5-propyl-4,5-dihydrooxazol-5-yl)but-2-enoate (9c)

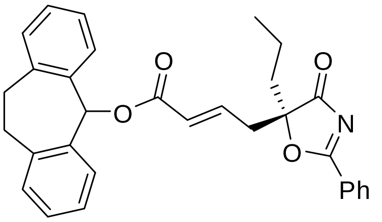

9c

A white solid; $[\alpha]^{25}_{D}$ = +47.3 (*c* 1.40, $CHCl_3$); ^1H NMR (500 MHz, $CDCl_3$) δ 8.20-8.18 (m, 2H), 7.71 (t, *J* = 7.0 Hz, 1H), 7.69-7.52 (m, 2H), 7.36 (d, *J* = 8.2 Hz, 2H), 7.23-7.20 (m, 2H) 7.14-7.11 (m, 4H), 6.89 (s, 1H), 6.84-6.78 (m, 1H), 6.00 (d, *J* = 15.8 Hz, 1H), 3.50-3.44 (m, 2H), 2.99-2.93 (m, 2H), 2.82-2.68 (m, 2H), 1.94-1.89 (m, 2H), 1.31-1.23 (m, 2H), 0.89 (t, *J* = 6.9 Hz, 3H); ^{13}C NMR (125 MHz, $CDCl_3$) δ 192.52, 185.60, 164.24, 139.94, 139.70, 136.45, 135.30, 130.27, 130.14, 129.54, 128.99, 128.70, 126.65, 126.08, 125.50, 89.48, 79.13, 38.41, 37.41, 32.29, 16.25, 13.74; HRMS (ESI) *m/z* calcd for $C_{31}H_{29}NNaO_4$ $[M+Na]^+$ = 502.1989, found = 502.2012; The ee value was 92%, t_R (major) = 44.2 min, t_R (minor) = 50.4 min (Chiralcel ID, λ = 254 nm, 20% *i*-PrOH/hexanes, flow rate = 1.0 mL/min).

<Chromatogram>

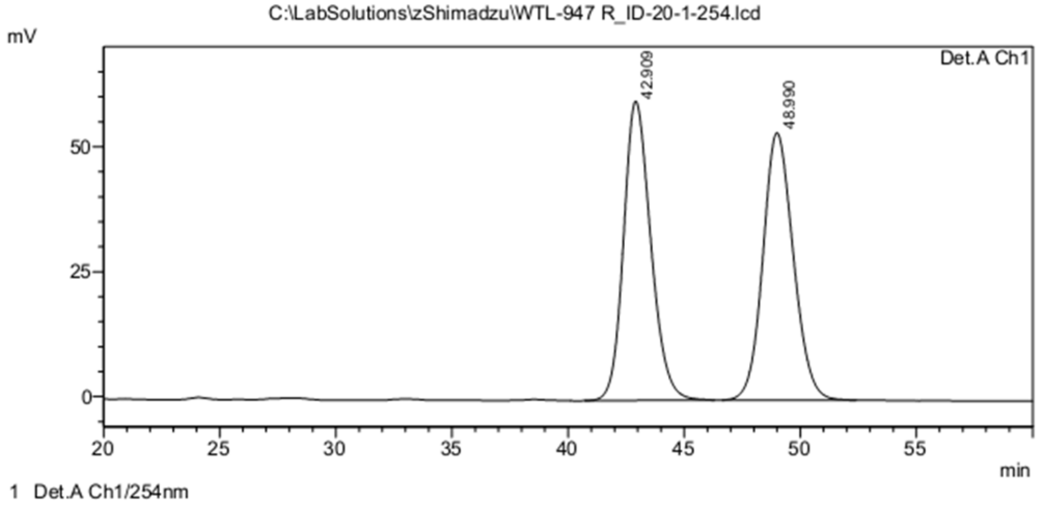

PeakTable

Detector A Ch1 254nm

Peak#	Ret. Time	Area	Height	Area %	Height %
1	42.909	4891841	59882	50.073	52.818
2	48.990	4877490	53493	49.927	47.182
Total		9769331	113375	100.000	100.000

Racemic **9c**

<Chromatogram>

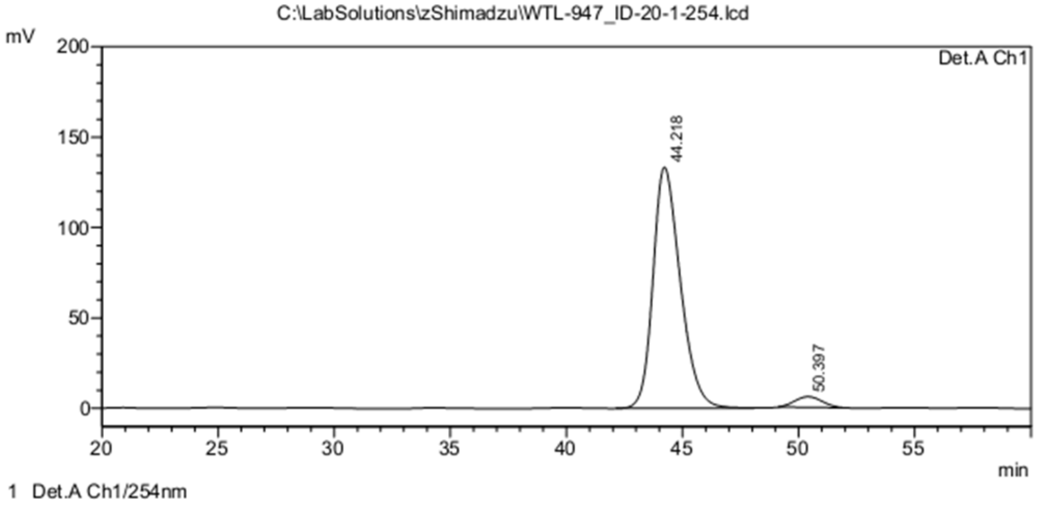

PeakTable

Detector A Ch1 254nm

Peak#	Ret. Time	Area	Height	Area %	Height %
1	44.218	10918912	133391	95.765	95.763
2	50.397	482894	5902	4.235	4.237
Total		11401806	139292	100.000	100.000

Enantiomerically enriched **9c**

(*S*,*E*)-10,11-dihydro-5*H*-dibenzo[a,d]cyclohepten-5-yl

4-(5-isopropyl-4-oxo-2-phenyl-4,5-dihydrooxazol-5-yl)but-2-enoate (9d)

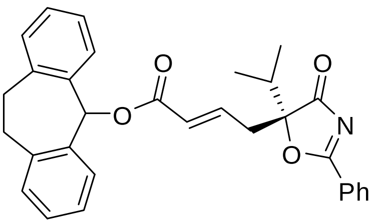

9d

A white solid; $[\alpha]^{25}_{D}$ = +39.4 (*c* 1.40, $CHCl_3$); ^{1}H NMR (500 MHz, $CDCl_3$) δ 8.18 (d, *J* = 7.6 Hz, 2H), 7.71 (t, *J* = 7.6 Hz, 1H), 7.53 (t, *J* = 8.2 Hz, 2H), 7.32 (d, *J* = 7.6 Hz, 2H), 7.22-7.19 (m, 2H), 7.12-7.10 (m, 4H), 6.86 (s, 1H), 6.79-6.73 (m, 1H), 5.98 (d, *J* = 15.8 Hz, 1H), 3.46-3.39 (m, 2H), 2.95-2.87 (m, 3H), 2.76-2.72 (m, 1H), 2.27-2.19 (m, 1H), 1.04 (d, *J* = 7.0 Hz, 3H), 0.98 (d, *J* = 6.9 Hz, 3H); ^{13}C NMR (125 MHz, $CDCl_3$) δ 192.23, 185.65, 164.16, 139.88, 139.70, 136.48, 136.46, 135.27, 130.25, 130.08, 129.45, 128.99, 128.66, 126.62, 126.07, 125.42, 91.87, 79.01, 36.22, 33.54, 32.25, 16.34, 16.14; HRMS (ESI) *m/z* calcd for $C_{31}H_{29}NNaO_4$ $[M+Na]^+$ = 502.1989, found = 502.1996; The ee value was 93%, t_R (major) = 41.2 min, t_R (minor) = 46.3 min (Chiralcel ID, λ = 254 nm, 20% *i*-PrOH/hexanes, flow rate = 1.0 mL/min).

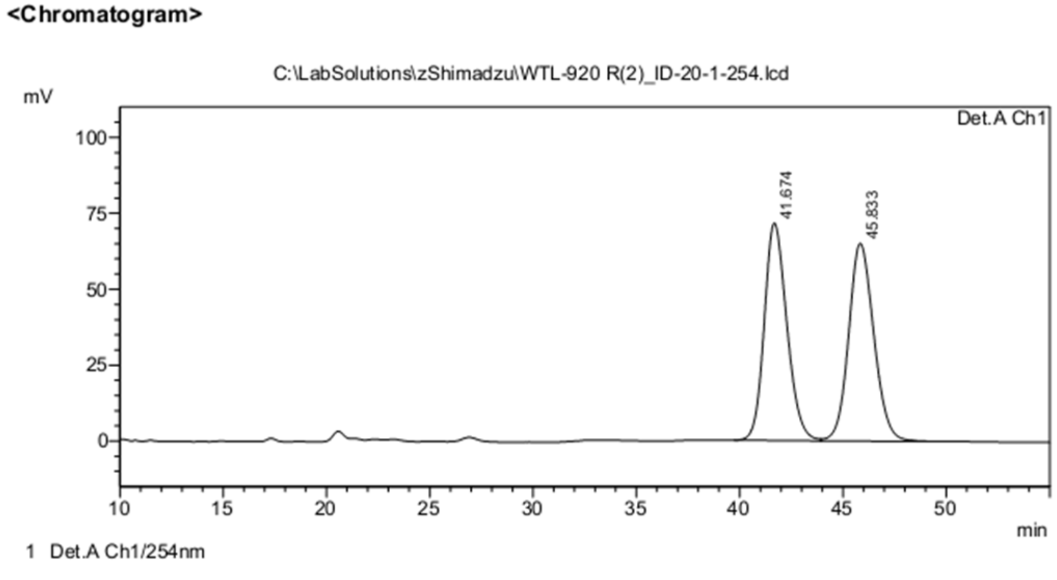

PeakTable

Detector A Ch1 254nm

Peak#	Ret. Time	Area	Height	Area %	Height %
1	41.674	5454330	71604	49.686	52.369
2	45.833	5523217	65126	50.314	47.631
Total		10977547	136730	100.000	100.000

Racemic **9d**

<Chromatogram>

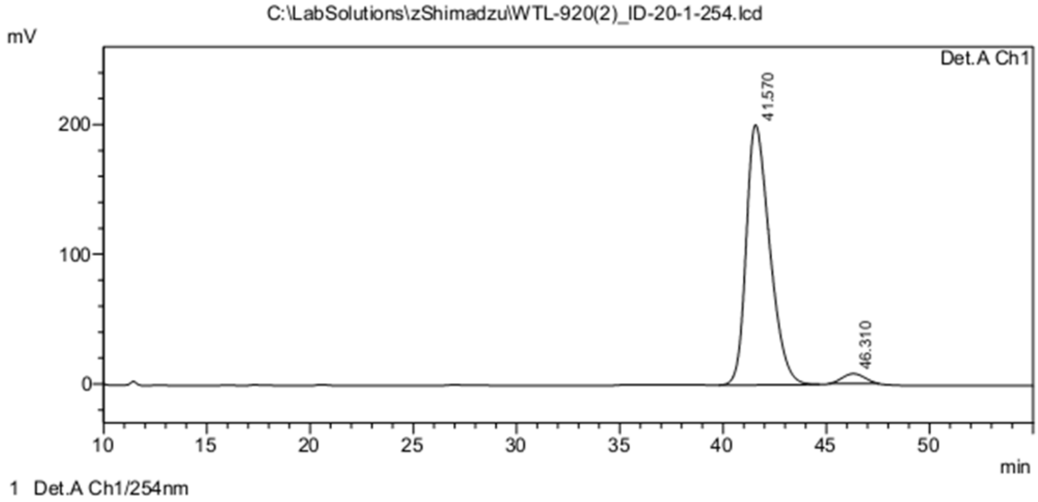

PeakTable

Detector A Ch1 254nm

Peak#	Ret. Time	Area	Height	Area %	Height %
1	41.570	15916989	200630	96.511	96.319
2	46.310	575379	7667	3.489	3.681
Total		16492368	208297	100.000	100.000

Enantiomerically enriched **9d**

(*S*,*E*)-10,11-dihydro-5*H*-dibenzo[a,d]cyclohepten-5-yl 4-(5-butyl-4-oxo-2-phenyl-4,5-dihydrooxazol-5-yl)but-2-enoate (9e)

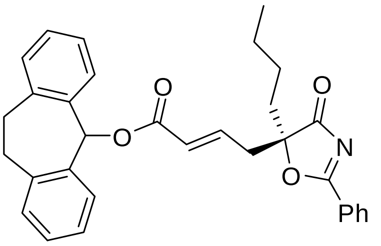

9e

A white solid; $[\alpha]^{25}_{D}$ = +39.7 (*c* 1.00, $CHCl_3$); ^1H NMR (500 MHz, $CDCl_3$) δ 8.20 (d, *J* = 8.0 Hz, 2H), 7.71 (t, *J* = 7.6 Hz, 1H), 7.54 (t, *J* = 7.6 Hz, 2H), 7.35 (d, *J* = 8.2 Hz, 2H), 7.21 (t, *J* = 7.6 Hz, 2H), 7.14-7.11 (m, 4H), 6.89 (s, 1H), 6.84-6.78 (m, 1H), 5.98 (d, *J* = 15.2 Hz, 1H), 3.49-3.43 (m, 2H), 2.99-2.77 (m, 2H), 2.73-2.69 (m, 2H), 1.96-1.91 (m, 2H), 1.31-1.17 (m, 4H), 0.84 (t, *J* = 7.0 Hz, 3H); ^{13}C NMR (125 MHz, $CDCl_3$) δ 192.54, 185.61, 164.24, 139.93, 139.70, 136.46, 135.31, 130.27, 130.16, 129.52, 128.99, 128.70, 126.65, 126.09, 125.52, 89.49, 79.12, 38.43, 35.14, 32.29, 24.80, 22.40, 13.69; HRMS (ESI) *m/z* calcd for $C_{32}H_{31}NNaO_4$ $[M+Na]^+$ = 516.2145, found = 516.2156; The ee value was 91%, t_R (major) = 18.0 min, t_R (minor) = 20.3 min (Chiralcel IC, λ = 254 nm, 40% *i*-PrOH/hexanes, flow rate = 1.0 mL/min).

<Chromatogram>

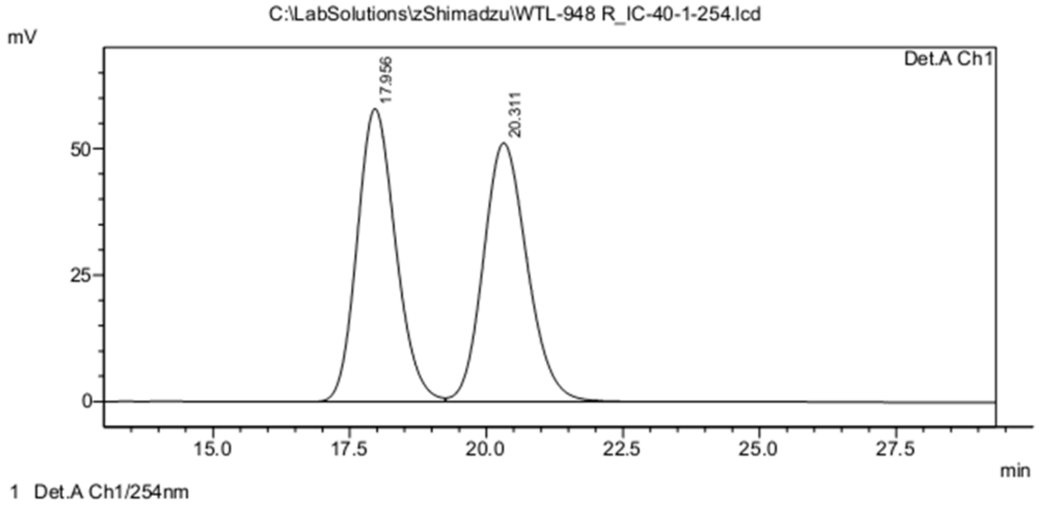

PeakTable

Detector A Ch1 254nm

Peak#	Ret. Time	Area	Height	Area %	Height %
1	17.956	2841202	57974	49.829	53.108
2	20.311	2860706	51188	50.171	46.892
Total		5701908	109163	100.000	100.000

Racemic **9e**

<Chromatogram>

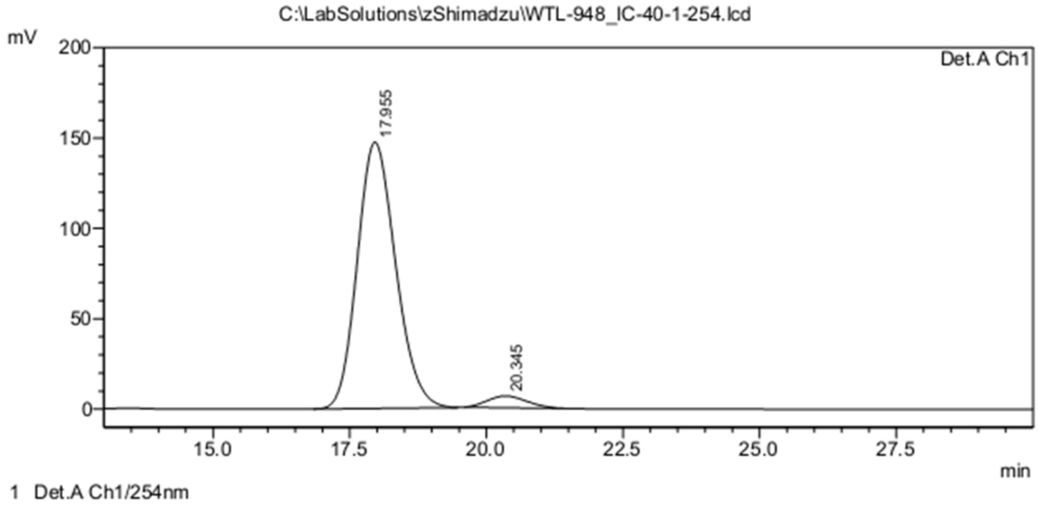

PeakTable

Detector A Ch1 254nm

Peak#	Ret. Time	Area	Height	Area %	Height %
1	17.955	7205814	147367	95.661	95.820
2	20.345	326828	6429	4.339	4.180
Total		7532642	153796	100.000	100.000

Enantiomerically enriched **9e**

(*S*,*E*)-10,11-dihydro-5*H*-dibenzo[a,d]cyclohepten-5-yl 4-(5-isobutyl-4-oxo-2-phenyl-4,5-dihydrooxazol-5-yl)but-2-enoate (9f)

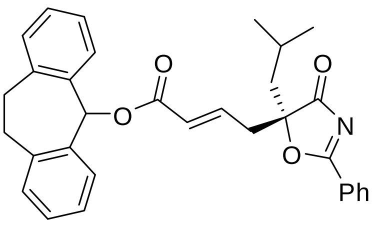

9f

A white solid; $[\alpha]^{25}_{D} = +47.9$ (*c* 0.90, $CHCl_3$); ^{1}H NMR (500 MHz, $CDCl_3$) δ 8.19 (dd, $J_{1,2}$ = 1.3 Hz, $J_{1,3}$ = 8.2 Hz, 2H), 7.71 (t, *J* = 7.6 Hz, 1H), 7.53 (t, *J* = 7.6 Hz, 2H), 7.35 (d, *J* = 8.2 Hz, 2H), 7.23-7.19 (m, 2H), 7.14-7.11 (m, 4H), 6.88 (s, 1H), 6.81-6.75 (m, 1H), 5.98 (d, *J* = 15.8 Hz, 1H), 3.47-3.42 (m, 2H), 2.98-2.92 (m, 2H), 2.81-2.76 (m, 1H), 2.71-2.67 (m, 1H), 1.98-1.94 (m, 1H), 1.84-1.80 (m, 1H), 1.72-1.66 (m, 1H), 0.91 (d, *J* = 6.9 Hz, 3H), 0.87 (d, *J* = 6.9 Hz, 3H); ^{13}C NMR (125 MHz, $CDCl_3$) δ 192.79, 185.54, 164.22, 139.94, 139.60, 136.49, 135.34, 130.30, 130.29, 130.16, 129.51, 129.06, 128.71, 126.75, 126.11, 125.61, 89.47, 79.12, 43.93, 39.09, 32.29, 24.23, 23.87, 23.56; HRMS (ESI) *m/z* calcd for $C_{32}H_{31}NNaO_4$ $[M+Na]^+$ = 516.2145, found = 516.2157; The ee value was 93%, t_R (major) = 16.9 min, t_R (minor) = 19.8 min (Chiralcel IC, λ = 254 nm, 40% *i*-PrOH/hexanes, flow rate = 1.0 mL/min).

<Chromatogram>

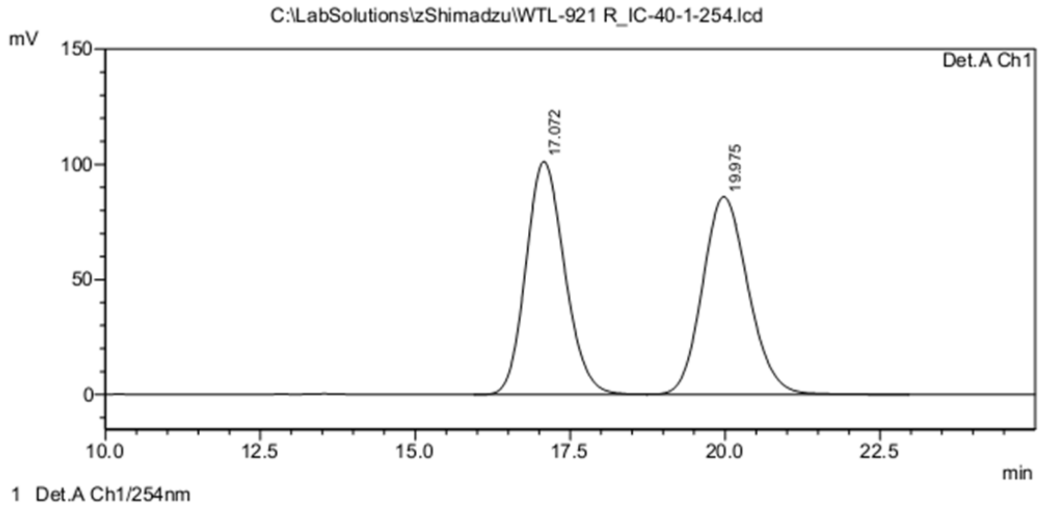

PeakTable

Detector A Ch1 254nm

Peak#	Ret. Time	Area	Height	Area %	Height %
1	17.072	4406811	101226	49.906	54.091
2	19.975	4423334	85914	50.094	45.909
Total		8830144	187140	100.000	100.000

Racemic **9f**

<Chromatogram>

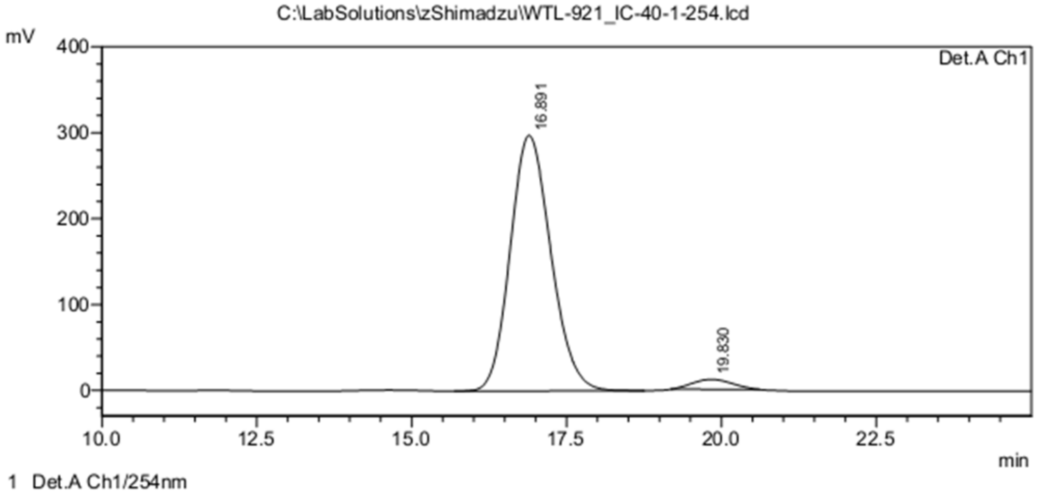

1 Det.A Ch1/254nm

PeakTable

Detector A Ch1 254nm

Peak#	Ret. Time	Area	Height	Area %	Height %
1	16.891	13602086	297586	96.348	96.302
2	19.830	515581	11427	3.652	3.698
Total		14117667	309013	100.000	100.000

Enantiomerically enriched **9f**

(*S*,*E*)-10,11-dihydro-5*H*-dibenzo[a,d]cyclohepten-5-yl 4-(5-*tert*-butyl-4-oxo-2-phenyl-4,5-dihydrooxazol-5-yl)but-2-enoate (9g)

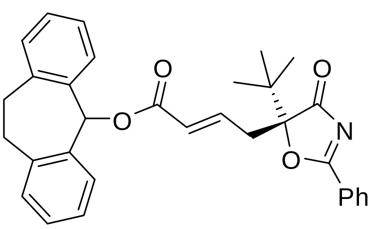

9g

A white solid; $[\alpha]^{25}_{D}$ =+61.4 (*c* 0.80, $CHCl_3$); ^{1}H NMR (500 MHz, $CDCl_3$) δ 8.16 (d, *J* = 8.2 Hz, 2H), 7.71 (t, *J* = 7.0 Hz, 1H), 7.52 (t, *J* = 7.6 Hz, 2H), 7.21-7.16 (m, 4H), 7.08-7.05 (m, 4H), 6.80 (s, 1H), 6.62-6.56 (m, 1H), 5.94 (d, *J* = 15.8 Hz, 1H), 3.36-3.29 (m, 2H), 2.90-2.84 (m, 4H), 1.09 (s, 9H); ^{13}C NMR (125 MHz, $CDCl_3$) δ 191.90, 185.34, 163.94, 139.72, 139.70, 136.46, 135.21, 130.20, 130.18, 130.00, 129.19, 128.99, 128.55, 127.95, 127.05, 126.82, 126.01, 114.05, 93.75, 78.74, 37.29, 33.89, 32.16, 24.52, 14.11; HRMS (ESI) *m/z* calcd for $C_{32}H_{31}NNaO_4$ $[M+Na]^+$ = 516.2145, found = 516.2158; The ee value was 97%, t_R (major) = 33.4 min, t_R (minor) = 37.3 min (Chiralcel ID, λ = 254 nm, 20%

i-PrOH/hexanes, flow rate = 1.0 mL/min).

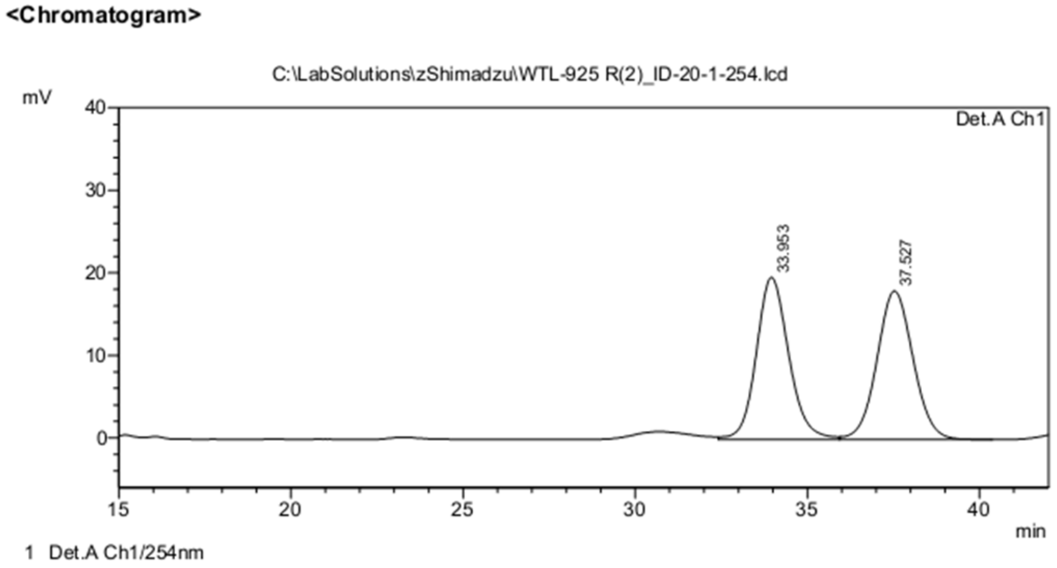

PeakTable

Detector A Ch1 254nm

Peak#	Ret. Time	Area	Height	Area %	Height %
1	33.953	1295943	19661	49.547	52.179
2	37.527	1319626	18019	50.453	47.821
Total		2615570	37679	100.000	100.000

Racemic **9g**

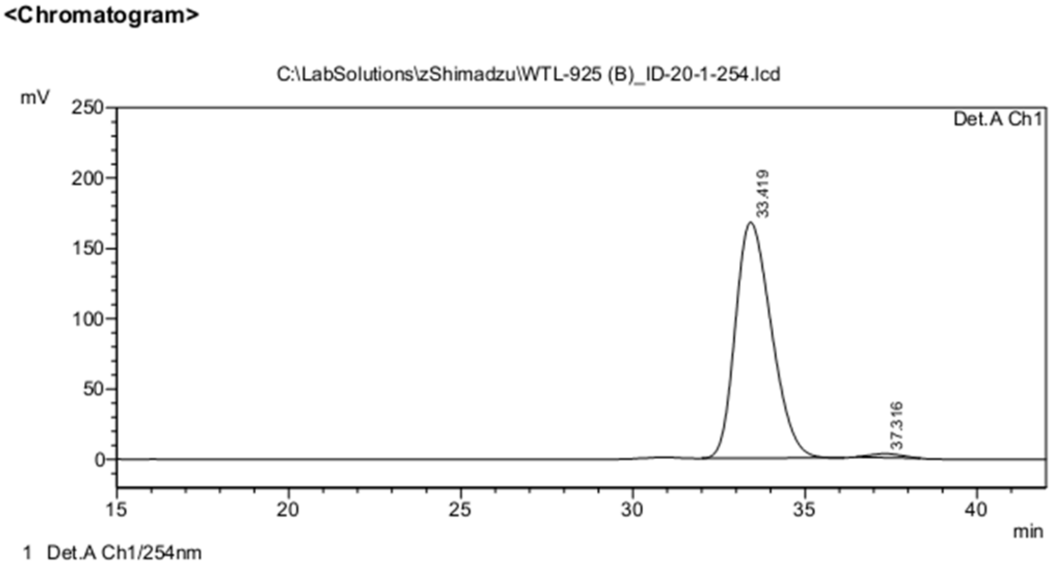

PeakTable

Detector A Ch1 254nm

Peak#	Ret. Time	Area	Height	Area %	Height %
1	33.419	12226918	167718	98.636	98.436
2	37.316	169072	2664	1.364	1.564
Total		12395990	170382	100.000	100.000

Enantiomerically enriched **9g**

(*S*,*E*)-10,11-dihydro-5*H*-dibenzo[a,d]cyclohepten-5-yl 4-(5-hexyl-4-oxo-2-phenyl-4,5-dihydrooxazol-5-yl)but-2-enoate (9h)

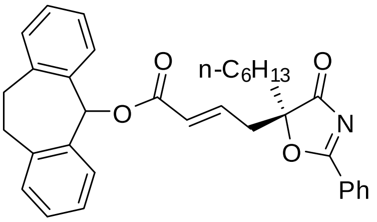

9h

A white foam; $[\alpha]^{25}_{D}$ = +19.9 (*c* 0.90, $CHCl_3$); ^{1}H NMR (500 MHz, $CDCl_3$) δ 8.20 (d, *J* = 7.6 Hz, 2H), 7.71 (t, *J* = 7.6 Hz, 1H), 7.54 (t, *J* = 8.2 Hz, 2H), 7.36 (d, *J* = 7.6 Hz, 2H), 7.22 (t, *J* = 7.6 Hz, 2H), 7.14-7.12 (m, 4H), 6.89 (s, 1H), 6.84-6.78 (m, 1H), 6.00 (d, *J* = 15.2 Hz, 1H), 3.50-3.44 (m, 2H), 2.98-2.93 (m, 2H), 2.82-2.68 (m, 2H), 1.98-1.89 (m, 2H), 1.26-1.20 (m, 8H), 0.83 (t, *J* = 6.3 Hz, 3H); ^{13}C NMR (125 MHz, $CDCl_3$) δ 192.55, 185.59, 164.24, 139.93, 139.72, 136.43, 136.41, 135.31, 130.26, 130.14, 129.53, 128.98, 128.69, 126.61, 126.07, 125.47, 89.50, 79.12, 38.41, 35.36, 32.27, 31.34, 29.67, 28.89, 22.66, 22.39, 13.90; HRMS (ESI) *m/z* calcd for $C_{34}H_{35}NNaO_4$ $[M+Na]^+$ = 544.2458, found = 544.2474; The ee value was 93%, t_R (major) = 16.2 min, t_R (minor) = 18.9 min (Chiralcel IC, λ = 254 nm, 40% *i*-PrOH/hexanes, flow rate = 1.0 mL/min).

<Chromatogram>

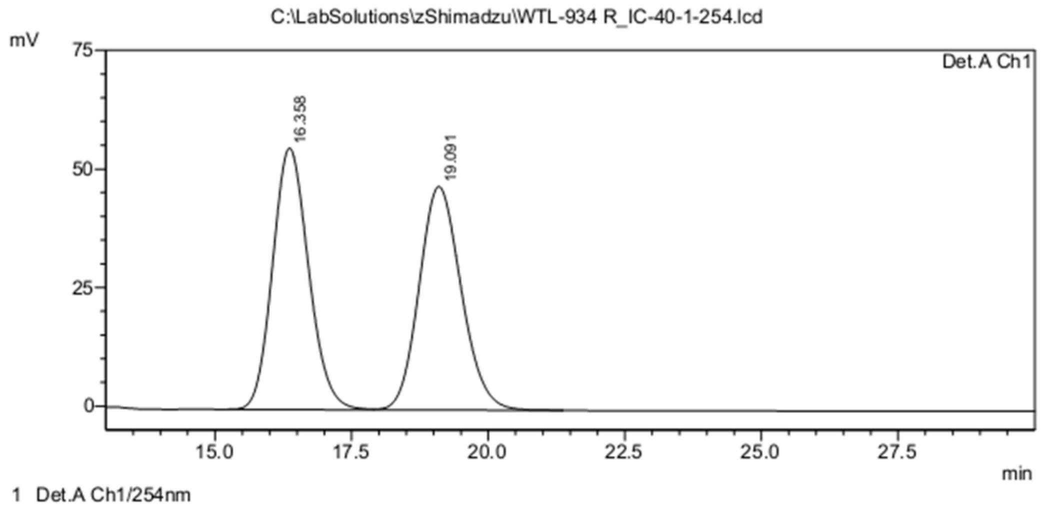

PeakTable

Detector A Ch1 254nm

Peak#	Ret. Time	Area	Height	Area %	Height %
1	16.358	2535259	55149	50.066	53.921
2	19.091	2528597	47128	49.934	46.079
Total		5063856	102278	100.000	100.000

Racemic **9h**

<Chromatogram>

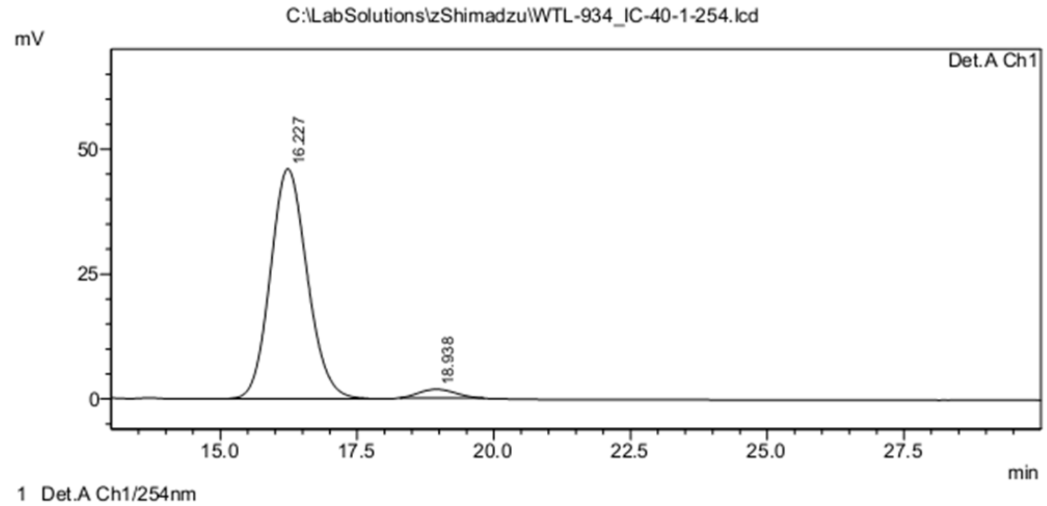

PeakTable

Detector A Ch1 254nm

Peak#	Ret. Time	Area	Height	Area %	Height %
1	16.227	2164825	46055	96.403	96.348
2	18.938	80779	1745	3.597	3.652
Total		2245604	47800	100.000	100.000

Enantiomerically enriched **9h**

(*S*,*E*)-10,11-dihydro-5*H*-dibenzo[a,d]cyclohepten-5-yl 4-(5-(2-(methylthio)ethyl)-4-oxo-2-phenyl-4,5-dihydrooxazol-5-yl)but-2-enoate (9i)

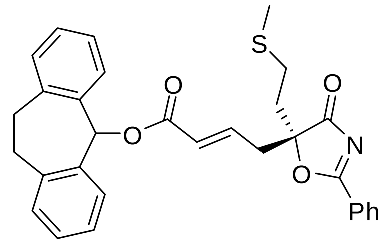

9i

A white solid; $[\alpha]^{25}_{D}$ = -41.7 (*c* 1.10, $CHCl_3$); ^{1}H NMR (500 MHz, $CDCl_3$) δ 8.19 (d, *J* = 7.6 Hz, 2H), 7.72 (t, *J* = 7.6 Hz, 1H), 7.54 (t, *J* = 7.6 Hz, 2H), 7.35 (d, *J* = 7.6 Hz, 2H), 7.23-7.20 (m, 2H), 7.14-7.12 (m, 4H), 6.88 (s, 1H), 6.82-6.76 (m, 1H), 6.00 (d, *J* = 15.8 Hz, 1H), 3.50-3.44 (m, 2H), 2.97-2.93 (m, 2H), 2.84-2.80 (m, 1H), 2.79-2.70 (m, 1H), 2.42-2.36 (m, 2H), 2.25 (t, *J* = 8.2 Hz, 2H), 2.04 (s, 3H); ^{13}C NMR (125 MHz, $CDCl_3$) δ 191.75, 185.70, 164.10, 139.94, 139.03, 136.34, 135.54, 130.27, 130.22, 129.59, 129.05, 128.72, 127.01, 126.08, 125.24, 88.44, 79.26, 38.41, 34.96, 32.27, 27.96; HRMS (ESI) *m/z* calcd for $C_{31}H_{29}NNaO_4$ $[M+Na]^+$ = 534.1710, found = 534.1716; The ee value was 94%, t_R (major) = 44.7 min, t_R (minor) = 65.7 min (Chiralcel IB, λ = 254 nm, 5% *i*-PrOH/hexanes,

flow rate = 1.0 mL/min).

<Chromatogram>

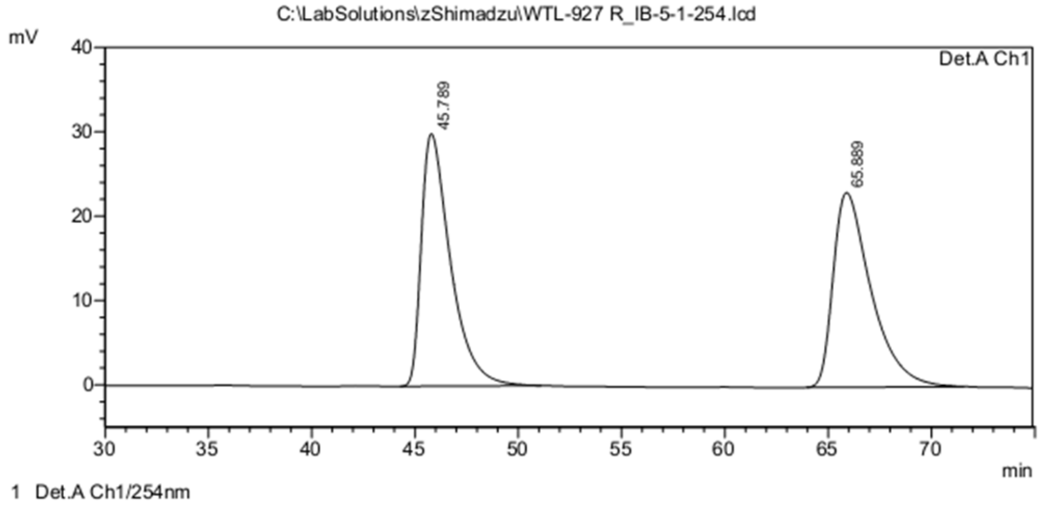

PeakTable

Detector A Ch1 254nm

Peak#	Ret. Time	Area	Height	Area %	Height %
1	45.789	2939944	29937	49.901	56.458
2	65.889	2951579	23089	50.099	43.542
Total		5891523	53026	100.000	100.000

Racemic **9i**

<Chromatogram>

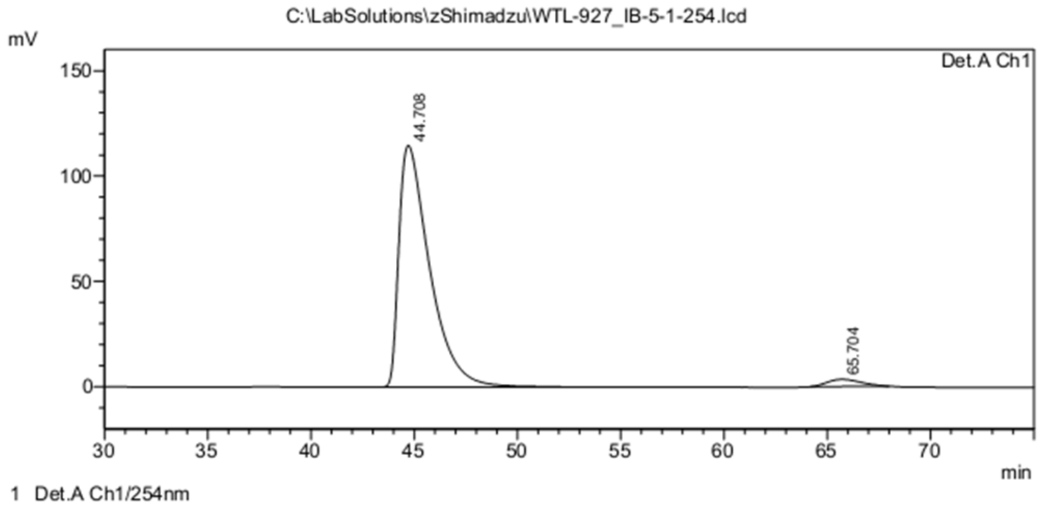

PeakTable

Detector A Ch1 254nm

Peak#	Ret. Time	Area	Height	Area %	Height %
1	44.708	11848726	114713	96.850	97.104
2	65.704	385332	3421	3.150	2.896
Total		12234058	118135	100.000	100.000

Enantiomerically enriched **9i**

(*S*,*E*)-10,11-dihydro-5*H*-dibenzo[a,d]cyclohepten-5-yl 4-(5-decyl-4-oxo-2-phenyl-4,5-dihydrooxazol-5-yl)but-2-enoate (9j)

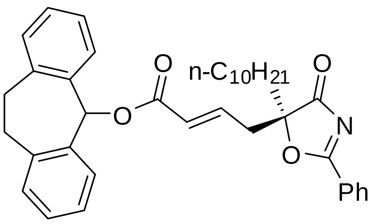

9j

A white foam; $[\alpha]^{25}_{D}$ = +20.3 (*c* 0.70, $CHCl_3$); ^{1}H NMR (500 MHz, $CDCl_3$) δ 8.22 (d, *J* = 7.0 Hz, 2H), 7.74 (t, *J* = 7.6 Hz, 1H), 7.56 (t, *J* = 8.2 Hz, 2H), 7.38 (d, *J* = 8.2 Hz, 2H), 7.24 (t, *J* = 7.6 Hz, 2H), 7.17-7.15 (m, 4H), 6.91 (s, 1H), 6.87-6.80 (m, 1H), 6.00 (d, *J* = 15.2 Hz, 1H), 3.52-3.46 (m, 2H), 3.00-2.95 (m, 2H), 2.84-2.71 (m, 2H), 2.00-1.94 (m, 2H), 1.34-1.22 (m, 16H), 0.89 (t, *J* = 6.9 Hz, 3H); ^{13}C NMR (125 MHz, $CDCl_3$) δ 192.56, 185.59, 164.25, 139.93, 139.73, 136.44, 136.42, 135.31, 130.27, 130.15, 129.54, 128.99, 128.70, 126.62, 126.08, 125.48, 89.52, 79.12, 38.42, 35.38, 32.27, 31.82, 29.43, 29.38, 29.26, 29.20, 29.17, 22.72, 22.62, 14.07; HRMS (ESI) *m/z* calcd for $C_{38}H_{43}NNaO_4$ $[M+Na]^+$ = 600.3084, found = 600.3088; The ee value was 92%, t_R (major) = 13.8 min, t_R (minor) = 16.9 min (Chiralcel IC, λ = 254 nm, 40% *i*-PrOH/hexanes, flow rate = 1.0 mL/min).

<Chromatogram>

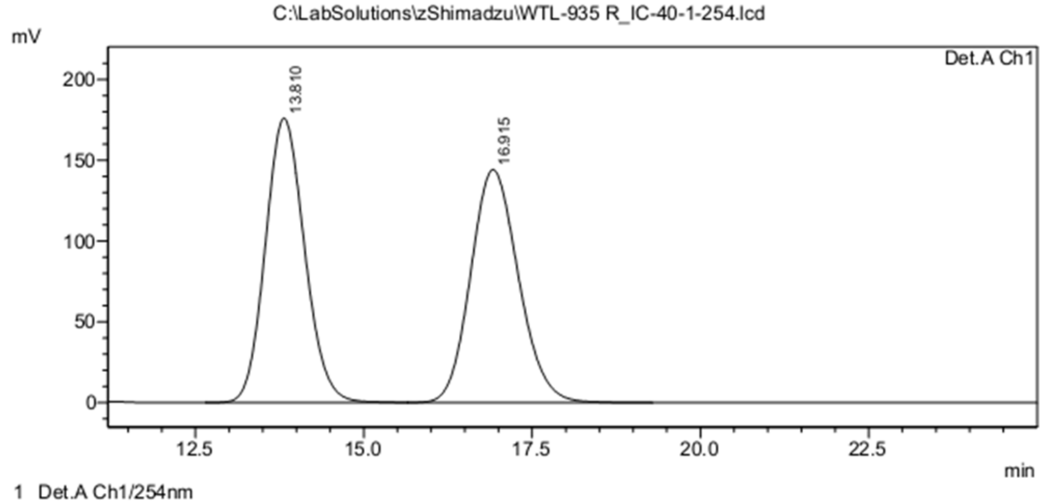

PeakTable

Detector A Ch1 254nm

Peak#	Ret. Time	Area	Height	Area %	Height %
1	13.810	7150257	175858	50.063	54.965
2	16.915	7132366	144085	49.937	45.035
Total		14282623	319943	100.000	100.000

Racemic **9j**

<Chromatogram>

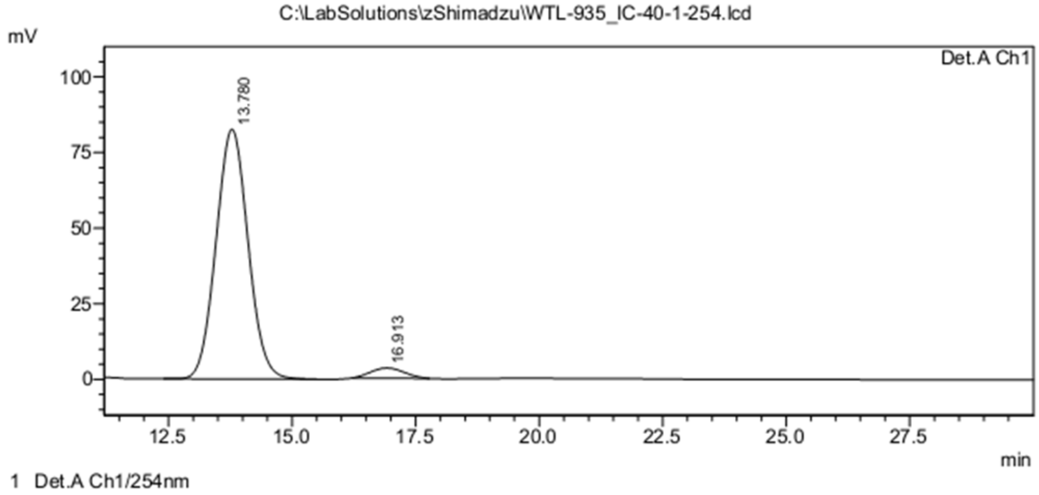

PeakTable

Detector A Ch1 254nm

Peak#	Ret. Time	Area	Height	Area %	Height %
1	13.780	3721578	82571	95.955	96.105
2	16.913	156879	3347	4.045	3.895
Total		3878457	85918	100.000	100.000

Enantiomerically enriched **9j**

(*R*,*E*)-10,11-dihydro-5*H*-dibenzo[a,d]cyclohepten-5-yl 4-(5-benzyl-4-oxo-2-phenyl-4,5-dihydrooxazol-5-yl)but-2-enoate (9k)

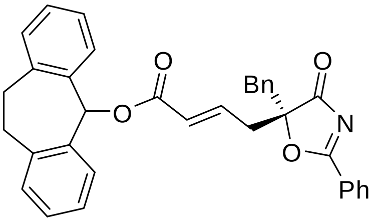

9k

A white solid; $[\alpha]^{25}_{D}$ = -41.3 (*c* 1.00, $CHCl_3$); ^{1}H NMR (500 MHz, $CDCl_3$) δ 8.05 (d, *J* = 8.2 Hz, 2H), 7.66 (t, *J* = 7.6 Hz, 1H), 7.48 (t, *J* = 8.2 Hz, 2H), 7.35 (d, *J* = 7.6 Hz, 2H), 7.22-7.10 (m, 11H), 6.87 (s, 1H), 6.84-6.78 (m, 1H), 6.00 (d, *J* = 15.8 Hz, 1H), 3.47-3.43 (m, 2H), 3.24-3.17 (m, 2H), 2.98-2.91 (m, 2H), 2.84-2.82 (m, 2H); ^{13}C NMR (125 MHz, $CDCl_3$) δ 191.85, 185.32, 164.17, 139.92, 139.39, 136.41, 135.21, 132.82, 130.27, 130.00, 129.98, 129.53, 128.90, 128.70, 128.44, 127.57, 126.87, 126.08, 125.33, 89.16, 79.17, 41.62, 37.96, 32.27, 29.68; HRMS (ESI) *m/z* calcd for $C_{35}H_{29}NNaO_4$ $[M+Na]^+$ = 550.1989, found = 550.1972; The ee value was 81%, t_R (major) = 37.1 min, t_R (minor) = 40.9 min (Chiralcel ID, λ = 254 nm, 20% *i*-PrOH/hexanes, flow rate = 1.0 mL/min).

<Chromatogram>

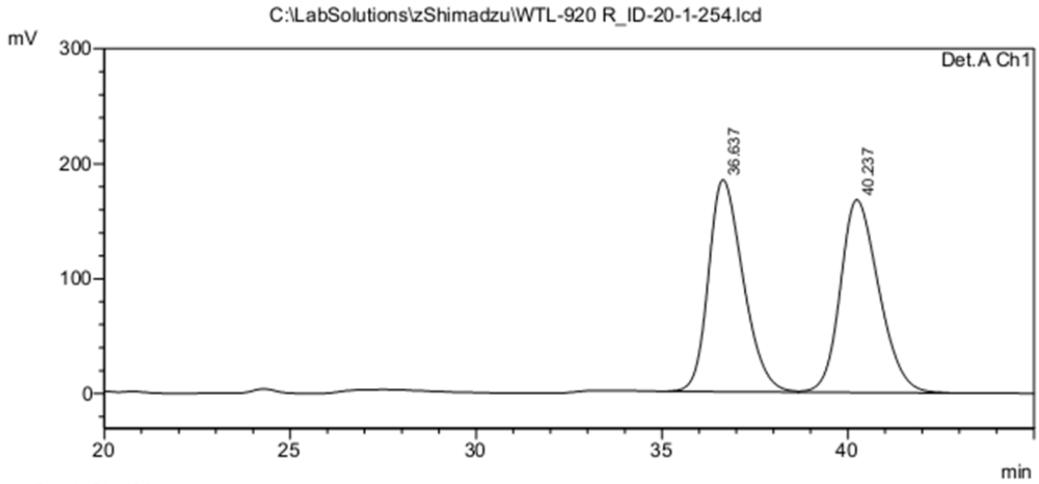

1 Det.A Ch1/254nm

PeakTable

Detector A Ch1 254nm

Peak#	Ret. Time	Area	Height	Area %	Height %
1	36.637	11952850	184246	49.692	52.381
2	40.237	12101257	167496	50.308	47.619
Total		24054107	351742	100.000	100.000

Racemic **9k**

<Chromatogram>

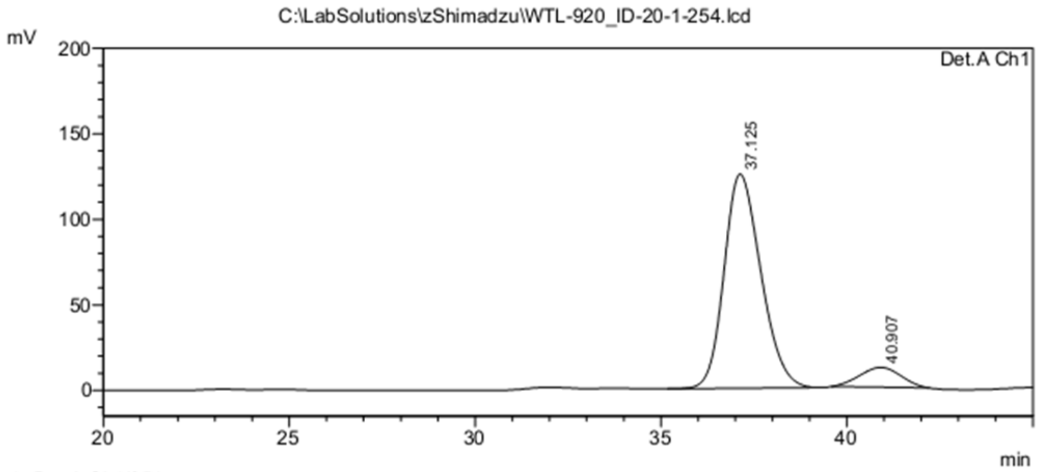

1 Det.A Ch1/254nm

PeakTable

Detector A Ch1 254nm

Peak#	Ret. Time	Area	Height	Area %	Height %
1	37.125	8634285	125268	90.599	91.544
2	40.907	895986	11571	9.401	8.456
Total		9530271	136839	100.000	100.000

Enantiomerically enriched **9k**

(*E*)-benzyl 3-(2,5-diphenylthiazol-4-yloxy)but-2-enoate (11)

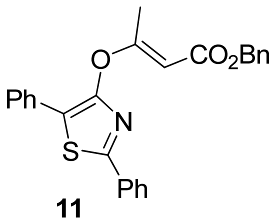

11

A white solid; 93% yield; ^{1}H NMR (500 MHz, $CDCl_3$) δ 7.93-7.91 (m, 2H), 7.58 (d, J = 7.6 Hz, 2H), 7.44 (t, J = 8.2 Hz, 3H), 7.41 (t, J = 7.6 Hz, 2H), 7.35-7.28 (m, 6H), 5.27 (s, 1H), 5.08 (s, 2H), 2.56 (s, 3H); ^{13}C NMR (125 MHz, $CDCl_3$) δ 170.76, 166.97, 162.98, 151.53, 136.19, 132.95, 130.55, 129.57, 129.02, 129.00, 128.49, 128.31, 128.24, 128.09, 127.37, 125.93, 122.23, 97.99, 65.74, 18.14; HRMS (ESI) *m/z* calcd for $C_{26}H_{22}NO_3S$ $[M+H]^+$ = 428.1320, found = 428.1317.

(*E*)-benzyl 4-(2,5-diphenyloxazol-4-yloxy)but-2-enoate (13)

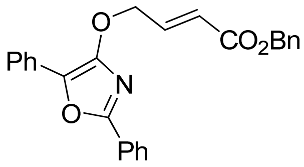

13

A white solid; 95% yield; ^{1}H NMR (500 MHz, $CDCl_3$) δ 7.49-7.47 (m, 4H), 7.41-7.33 (m, 11H), 7.00-6.95 (m, 1H), 5.18 (s, 2H), 4.41 (dd, $J_{1,2}$ = 1.3 Hz, $J_{2,3}$ = 5.1 Hz, 2H); ^{13}C NMR (125 MHz, $CDCl_3$) δ 169.93, 165.44, 141.49, 136.37, 135.67, 129.99, 129.86, 128.59, 128.58, 129.36, 129.31, 126.96, 122.95, 66.49, 38.60; HRMS (ESI) *m/z* calcd for $C_{26}H_{21}NnaO_4$ $[M+Na]^+$ = 434.1368, found = 434.1361.

10. Asymmetric Synthesis of Chiral Tertiary Alcohols and Thioethers

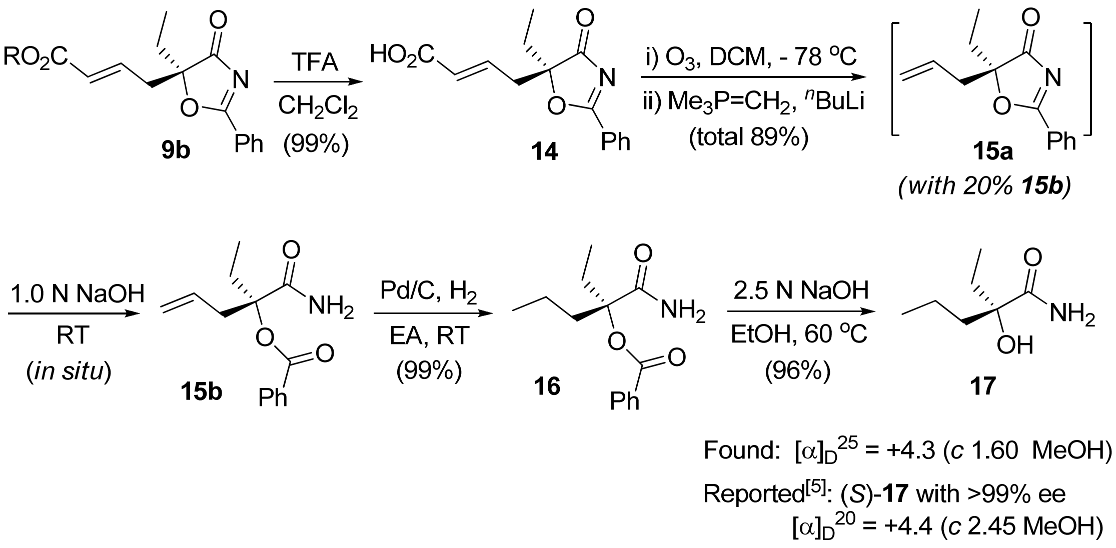

Scheme S3: Elaboration of γ-addition adducts into enantioenriched tertiary alcohol **17**.

(*S,E*)-4-(5-ethyl-4-oxo-2-phenyl-4,5-dihydrooxazol-5-yl)but-2-enoic acid (14)

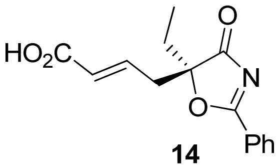

Addition product **9b** (186 mg, 0.40 mmol) was dissolved in a solution of TFA (62 μL, 0.80 mmol) in CH_2Cl_2 (5 mL), and the reaction mixture was stirred at room temperature for 1 h. Saturated aqueous $NaHCO_3$ was added to quench the reaction, and the resulting mixture was extracted with CH_2Cl_2 several times (3 × 5 mL). The combined organic extracts were dried over Na_2SO_4, filtered and concentrated. The residue was purified by flash column chromatography (hexane/ethyl acetate = 3:1) to afford the intermediate **14** as a white solid (108 mg, 99% yield). ^{1}H NMR (500 MHz, $CDCl_3$) δ 8.23 (d, *J* = 7.6 Hz, 2H), 7.72 (t, *J* = 7.6 Hz, 1H), 7.55 (t, *J* = 7.6 Hz, 2H), 6.93-6.87 (m, 1H), 5.96 (d, *J* = 15.8 Hz, 1H), 2.87-2.75 (m, 2H), 2.04-1.99 (m, 2H), 0.89 (t, *J* = 7.6 Hz, 3H); ^{13}C NMR (125 MHz, $CDCl_3$) δ 192.32, 185.78, 169.42, 141.95, 135.43, 130.17, 129.05, 125.67, 125.40, 89.68, 38.12, 7.18; HRMS (ESI) *m/z* calcd for $C_{15}H_{14}NO_4$ $[M-H]^-$ = 272.0923, found = 272.0926.

(*S*)-5-allyl-5-ethyl-2-phenyloxazol-4(5H)-one (15a)

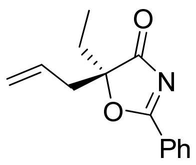

15a

To a solution of **14** (100 mg, 0.37 mmol) in CH_2Cl_2 (3 mL) at -78 ^oC, O_3 was bubbled until the reaction was complete (monitored by TLC). Ph_3P was then added to the reaction system at -78 ^oC, then kept stirring for another 10 min. The reaction mixture was passed through a short pad of silica gel, and eluted with EtOAc. The filtrate was concentrated and the residue was purified by flash column chromatography on silical gel (hexane/ethyl acetate = 10:1 to 3:1) to afford the corresponding aldehyde as a white foam, which was used directly in the next step.

To a solution of methyltrimethylphosphonium iodide (121 mg, 0.56 mmol) in dry THF (3.0 mL) at -78 ^oC, *n*-BuLi (290 µL, 2.0 M in hexane, 0.58 mmol) was slowly added and the mixture was kept stirring at -78 ^oC for 1 h. The mixture was warmed to 0 ^oC and stirred for another 1 h. The crude aldehyde in dry THF (2.0 mL) was introduced dropwise, and the resulting mixture was stirred at 0 ^oC until the reaction was complete (monitored by TLC). The reaction mixture was filtrated by a short pad of silica gel, and eluted with EtOAc. The filtrate was concentrated and the residue was purified by flash column chromatography (hexane/ethyl acetate = 10:1 to 2:1) to afford pure compound **15a** as a white foam (58.3 mg, 69% yield) and **15b** as a white solid (18.3 mg, 20% yield). Then, to the solution of **15a** in THF was added 1 M aqueous NaOH (0.25 mL, 0.25 mmol), and the mixture was stirred 0.5 h at room temperature, and extracted using CH_2Cl_2 (3 × 3 mL). The combined organic phases were washed with brine, dried with Na_2SO_4, and concentrated under reduced pressure. Chromatographic purification (silica gel, hexane/ethyl acetate = 3:1) of the residual material yielded **15b** as a white solid (totally: 81.3 mg, 89% yield, 69% from **15a**). The compound **15a**: ^{1}H NMR (500 MHz, $CDCl_3$) δ 8.26 (d, J = 7.0 Hz, 2H), 7.22 (t, J = 7.6 Hz, 1H), 7.57 (t, J = 8.2 Hz, 2H), 5.73-5.64 (m, 1H), 5.20 (dd, $J_{1,2}$ = 1.3 Hz, $J_{1,3}$ = 17.0 Hz, 1H), 5.13 (d, J = 10.0 Hz, 1H), 2.72-2.65 (m, 2H), 2.07-1.99 (m, 2H), 0.91 (t, J = 7.0 Hz, 3H); ^{13}C NMR (125 MHz, $CDCl_3$) δ 193.14, 185.62, 135.05, 130.01, 129.57, 128.92, 125.84, 120.76, 91.05, 39.96, 28.71, 7.27; HRMS (ESI) *m/z* calcd for $C_{14}H_{16}NO_2$ $[M+H]^+$ = 230.1181, found = 230.1183.

(*S*)-3-carbamoylhex-5-en-3-yl benzoate (15b)

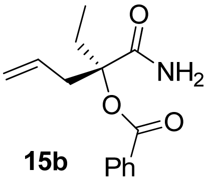

15b

^{1}H NMR (500 MHz, $CDCl_3$) δ 7.95 (d, J = 7.6 Hz, 2H), 7.58 (t, J = 6.9 Hz, 1H), 7.46 (t, J = 7.6 Hz, 2H), 6.41 (br, 2H), 5.76-5.68 (m, 1H), 5.05 (dd, $J_{1,2}$ = 17.1 Hz, $J_{1,3}$ = 35.3 Hz, 2H), 3.19-3.15 (m, 1H), 2.94-2.89 (m, 1H), 2.48-2.41 (m, 1H), 2.21-2.14 (m, 1H), 0.91 (t, J = 7.0 Hz, 3H); ^{13}C NMR (125 MHz, $CDCl_3$) δ 174.33, 164.43, 133.22, 131.46, 130.39, 129.28, 128.58, 119.36, 88.58, 39.10, 28.12, 7.84; HRMS (ESI) *m/z* calcd for $C_{14}H_{16}NO_3$ $[M-H]^-$ = 246.1130, found = 246.1130.

(*S*)-3-carbamoylhexan-3-yl benzoate (16)

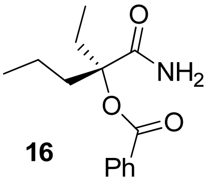

16

To a stirred solution of (*S*)-3-carbamoylhex-5-en-3-yl benzoate (**15b**) (80 mg, 0.32 mmol) in EtOAc (3 ml) was added 10% Pd/C (30 mg). The reaction vessel was evacuated and back-filled with hydrogen for 3 times and afterwards the reaction mixture was stirred under hydrogen atmosphere (1 atm) at room temperature for 2 h. Then, the reaction mixture was filtered over celite, concentrated, and passed through a short pad of silica gel (eluting with EtOAc) to give the title compound **16** as a white solid (79.7 mg, 99% yield). ^{1}H NMR (300 MHz, $CDCl_3$) δ 7.96 (d, J = 7.1 Hz, 2H), 7.60 (t, J = 7.2 Hz, 1H), 7.47 (t, J = 7.7 Hz, 2H), 6.48 (br, 2H), 2.53-2.22 (m, 2H), 2.19-2.04 (m, 2H), 1.41-1.25 (m, 2H), 0.92-0.85 (m, 6H); ^{13}C NMR (75 MHz, $CDCl_3$) δ 175.01, 164.34, 133.25, 130.45, 129.27, 128.63, 89.92, 37.10, 28.26, 17.00, 13.96, 7.89; HRMS (ESI) *m/z* calcd for $C_{14}H_{19}NNaO_3$ $[M+Na]^+$ = 272.1263, found = 272.1270.

(*S*)-2-ethyl-2-hydroxypentanamide (17)

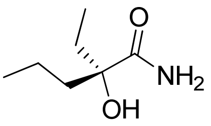

17

To a stirred solution of (*S*)-3-carbamoylhexan-3-yl benzoate (**16**) (50 mg, 0.2 mmol) in ethanol (1 ml) was added 2.5 N NaOH aqueous solution (0.4 ml) at room temperature. The mixture was then heated to 60 °C and stirred for 2 h at this high temperature. After cooling to RT, the reaction mixture was diluted with water, extracted with EtOAc (3 × 2 mL). The combined organic phase was washed with brine, dried with $MgSO_4$ and concentrated. The obtained crude product was purified by flash column chromatography on silical gel (pure ethyl acetate) to afford the title compound **17** as white solid (28.0 mg, 96% yield). ^{1}H NMR (500 MHz, $CDCl_3$) δ 6.51 (br, 1H), 5.75 (br, 1H), 2.15 (br, 1H), 1.87-1.75 (m, 2H), 1.64-1.57 (m, 2H), 1.55-1.41 (m, 1H), 1.32-1.25 (m, 1H), 0.93-0.90 (m, 6H); ^{13}C NMR (125 MHz, $CDCl_3$) δ 177.91, 78.72, 41.86, 32.67, 16.71, 14.23, 7.61.

(*S*)-**17** is a known compound, and its analytical data were reported in literature[5] : >99% ee, $[\alpha]^{20}_D$ = +4.4 (*c* 2.45, MeOH). Derivative **17** obtained in this report was found to have $[\alpha]^{25}_D$ = +4.3 (*c* 1.60, MeOH). Therefore, the absolute configuration of γ-addition product **9b** was deduced to be *S*.

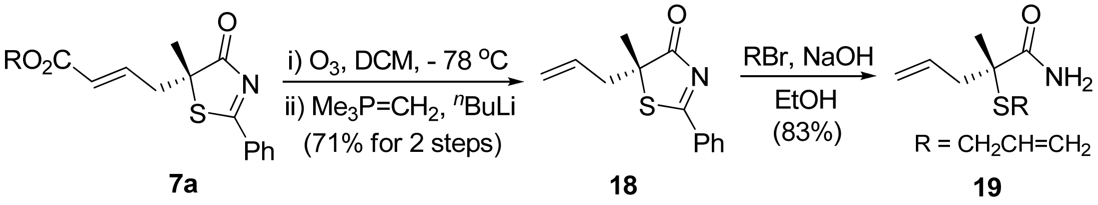

Scheme S4: Asymmetric synthesis of chiral tertiary thioethers.

<u>(*R*)-5-allyl-5-methyl-2-phenylthiazol-4(5H)-one (18)</u>

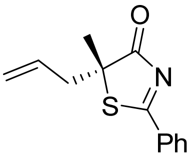

18

The compound **18** was synthesized from **7a**, following the above same procedure described for the synthesis of **15a**.

A white foam; 71% yield for two steps; ^{1}H NMR (500 MHz, $CDCl_3$) δ 8.13 (d, *J* = 7.6 Hz, 2H), 7.67 (t,

J = 6.9 Hz, 1H), 7.52 (t, J = 7.6 Hz, 2H), 5.77-5.69 (m, 1H), 5.22-5.14 (m, 2H), 2.67 (d, J = 7.6 Hz, 2H), 1.70 (s, 3H); ^{13}C NMR (125 MHz, $CDCl_3$) δ 195.40, 194.81, 134.98, 132.27, 131.83, 128.99, 128.83, 120.52, 64.20, 43.86, 25.22; HRMS (ESI) *m/z* calcd for $C_{13}H_{14}NOS$ $[M+H]^+$ = 232.0796, found = 232.0799.

(*R*)-2-(allylthio)-2-methylpent-4-enamide (19)

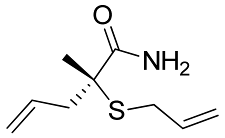

19

To a solution of (*R*)-5-allyl-5-methyl-2-phenylthiazol-4(5*H*)-one (**18**) (46 mg, 0.2 mmol) and allyl bromide (48 mg, 0.4 mmol) in EtOH (2 mL) was 2.5 N NaOH (0.4 mL, 1.0 mmol), and the reaction was complete after stirring for 2 h at 40 °C. Then, the reaction was quenched with 1N aq. $KHSO_4$ at room temperature, followed by extraction with CH_2Cl_2. The combined organic layers were then dried by $NaSO_4$. After removing solvents, the residue was purified by flash column chromatography on silical gel (hexane/ethyl acetate = 2:1) to afford the title compound **19** as a yellow oil (30.5 mg, 83% yield). $[\alpha]^{25}_D$ = +9.1 (*c* 1.20, $CHCl_3$); ^{1}H NMR (500 MHz, $CDCl_3$) δ 6.76 (br, 1H), 5.88-5.78 (m, 2H), 5.64 (br, 1H), 5.22-5.09 (m, 4H), 3.23 (d, J = 7.0 Hz, 2H), 2.61-2.49 (m, 2H), 1.48 (s, 3H); ^{13}C NMR (125 MHz, $CDCl_3$) δ 176.31, 133.24, 132.68, 119.03, 118.08, 52.93, 43.22, 32.92, 23.58; HRMS (ESI) *m/z* calcd for $C_9H_{15}NNaOS$ $[M+Na]^+$ = 208.0772, found = 208.0772.

11. DFT Studies on Mechanism

A. Complete reference for Gaussian 09

Frisch, M. J.; Trucks, G. W.; Schlegel, H. B.; Scuseria, G. E.; Robb, M. A.; Cheeseman, J. R.; Scalmani, G.; Barone, V.; Mennucci, B.; Petersson, G. A.; Nakatsuji, H.; Caricato, M.; Li, X.; Hratchian, H. P.; Izmaylov, A. F.; Bloino, J.; Zheng, G.; Sonnenberg, J. L.; Hada, M.; Ehara, M.; Toyota, K.; Fukuda, R.; Hasegawa, J.; Ishida, M.; Nakajima, T.; Honda, Y.; Kitao, O.; Nakai, H.; Vreven, T.; Montgomery, J. A. Jr.; Peralta, J. E.; Ogliaro, F.; Bearpark, M.; Heyd, J. J.; Brothers, E.; Kudin, K. N.; Staroverov, V. N.; Keith, T.; Kobayashi, R.; Normand, J.; Raghavachari, K.; Rendell, A.; Burant, J. C.; Iyengar, S. S.; Tomasi, J.; Cossi, M.; Rega, N.; Millam, J. M.; Klene, M.;

Knox, J. E.; Cross, J. B.; Bakken, V.; Adamo, C.; Jaramillo, J.; Gomperts, R.; Stratmann, R. E.; Yazyev, O.; Austin, A. J.; Cammi, R.; Pomelli, C.; Ochterski, J. W.; Martin, R. L.; Morokuma, K.; Zakrzewski, V. G.; Voth, G. A.; Salvador, P.; Dannenberg, J. J.; Dapprich, S.; Daniels, A. D.; Farkas, O.; Foresman, J. B.; Ortiz, J. V.; Cioslowski, J.; and Fox, D. J. Gaussian 09, revision D.01; Gaussian, Inc.: Wallingford, CT, **2013**.

B. B3LYP, B3LYP-D3 and M11 absolute calculation energies, enthalpies, and free energies.

Geometry	$E_{B3LYP(elec)}$	$E_{B3LYP\text{-}D3(toluene)}$	$E_{M11(toluene)}$	Correction of $H_{(gas\ phase)}$	Correction of $G_{(gas\ phase)}$	IF*
**2c**	-2822.075341	-2822.744192	-2821.493600	0.791897	0.655959	-
**6c**	-575.575922	-575.759359	-575.412988	0.194183	0.139518	-
**A**	-3397.661430	-3398.520352	-3396.920483	0.988217	0.817636	-
**Ts1**	-3397.639423	-3398.499102	-3396.897344	0.987172	0.822622	-179.3
**B**	-3397.653892	-3398.518565	-3396.920346	0.988900	0.825462	-
**5a**	-914.646207	-914.837481	-914.495147	0.181450	0.130806	-
**C**	-4312.309060	-4313.379685	-4311.436325	1.173129	0.980026	-
**Ts2**	-4312.283606	-4313.353388	-4311.405462	1.167758	0.976554	-1234.0
**D**	-4312.313235	-4313.387337	-4311.448216	1.172730	0.978447	-
**Ts4-*re***	-4312.299426	-4313.375809	-4311.435569	1.172139	0.979317	-259.2
***(R)*-E**	-4312.311239	-4313.382827	-4311.446735	1.174308	0.981624	-
**Ts4-*si***	-4312.298085	-4313.376709	-4311.434779	1.172352	0.983241	-244.2
***(S)*-E**	-4312.318477	-4313.390823	-4311.454855	1.174896	0.986201	-
***(R)*-7a**	-1490.271434	-1490.648619	-1489.964739	0.380207	0.296375	-
***(S)*-7a**	-1490.271434	-1490.648619	-1489.964739	0.380209	0.296379	-
**8a**	-591.682944	-591.870631	-591.533392	0.184348	0.135798	-
**F**	-3989.347169	-3990.410634	-3988.472797	1.175989	0.983922	-
**Ts3**	-3989.319620	-3990.382240	-3988.436851	1.170596	0.982474	-1209.3
**G**	-3989.344417	-3990.409216	-3988.475334	1.175345	0.981949	-
**Ts5-*re***	-3989.334776	-3990.400899	-3988.462835	1.175083	0.983596	-201.6
***(R)*-H**	-3989.361485	-3990.418645	-3988.489186	1.177685	0.986427	-
**Ts5-*si***	-3989.332424	-3990.401829	-3988.463724	1.174839	0.985598	-154.4
***(S)*-H**	-3989.362003	-3990.427619	-3988.497346	1.177752	0.989186	-
***(R)*-9a**	-1167.313434	-1167.684378	-1167.005264	0.382948	0.300932	-
***(S)*-9a**	-1167.313434	-1167.684378	-1167.005264	0.382948	0.300947	-

C. B3LYP geometries for all the optimized compounds and transition states.

2c

C	-0.73064600	-1.30569200	-2.12239100
H	-0.85648300	-2.19934900	-2.74184400
H	-1.70659500	-1.02712800	-1.71315200
H	-0.37801500	-0.49483400	-2.76858400
C	0.25666800	-1.58564100	-0.99188300
C	0.42693600	-0.42092900	0.01794300
C	0.90886400	0.87308400	-0.65704600
H	0.12111000	1.27104100	-1.30821500
H	1.77181800	0.61866000	-1.27927900
P	1.32549100	2.22891700	0.58060500
C	2.94235200	1.61544000	1.24548200
C	2.92376100	0.96608700	2.49079100
C	4.16931200	1.75618200	0.57676000
C	4.09884100	0.45378900	3.04623500
H	1.98568800	0.86296100	3.03089200
C	5.34466200	1.25569800	1.13772700
H	4.20883400	2.26741200	-0.38099000
C	5.31180200	0.60098900	2.37205100
H	4.06470000	-0.05096800	4.00795400
H	6.28762000	1.37672200	0.61056300
H	6.22873400	0.21235300	2.80727200
C	1.82511800	3.62214300	-0.52639400
C	1.93026400	4.89211900	0.06849300
C	2.06926400	3.51081800	-1.90475600
C	2.28670200	6.01064900	-0.68376600
H	1.72535300	5.00327200	1.13078900
C	2.41365200	4.63356500	-2.66206300
H	1.99285600	2.54748600	-2.39972100
C	2.52706700	5.88441400	-2.05408600
H	2.36646500	6.98197900	-0.20276000
H	2.59613200	4.52740500	-3.72843100
H	2.79572900	6.75630100	-2.64455500
O	1.56188700	-1.86664900	-1.50220400
Si	2.31145600	-3.33472200	-1.82179700
C	4.15856800	-3.07305500	-1.35775300
C	4.97781900	-4.34029100	-1.68480700
H	4.61529800	-5.21771300	-1.13443800
H	4.95819500	-4.57720000	-2.75464800
H	6.03022800	-4.19026400	-1.40376700
C	4.31755600	-2.75004700	0.14281200
H	5.37119400	-2.53112800	0.36843600
H	3.73365500	-1.87237300	0.44141900
H	4.01906700	-3.59346700	0.77495600
C	4.71113400	-1.88753700	-2.18101100
H	4.18452600	-0.95569200	-1.94622600
H	5.77537000	-1.73348000	-1.95010700
H	4.62864600	-2.06228900	-3.26014900
N	-0.85139500	-0.14249100	0.71529900
H	-0.89038700	0.81552500	1.06774400
H	1.18795700	-0.75046000	0.73558500
H	-0.10175500	-2.45327100	-0.42559700
C	2.17059200	-3.67788200	-3.68049000
C	2.58427300	-4.89023900	-4.26947700

C	1.69021800	-2.67502000	-4.54374900
C	2.50700300	-5.09670600	-5.64833000
H	2.97315200	-5.69265700	-3.64738400
C	1.61214300	-2.87376100	-5.92406900
H	1.37680700	-1.72249700	-4.12737800
C	2.01768900	-4.08777300	-6.48030000
H	2.83004800	-6.04422200	-6.07244100
H	1.23519400	-2.08019600	-6.56471400
H	1.95651100	-4.24620500	-7.55398300
C	1.47987100	-4.70829400	-0.81331500
C	1.38017600	-4.60439400	0.59067800
C	0.93119800	-5.85814800	-1.41354700
C	0.78688100	-5.61011000	1.35690200
H	1.74614400	-3.71958700	1.10270000
C	0.33831200	-6.86859400	-0.65245200
H	0.95668600	-5.96764800	-2.49360400
C	0.27008000	-6.75026100	0.73656100
H	0.72595600	-5.49689700	2.43614200
H	-0.07164200	-7.74638400	-1.14603800
H	-0.18642700	-7.53732700	1.33168200
S	-1.32511100	-1.13068900	2.01378000
O	-1.92145700	-0.21179300	2.99065300
O	-0.22266200	-2.03021600	2.38383500
C	-2.64276100	-2.13631200	1.33071500
C	-3.87763500	-1.54247400	1.05239600
C	-2.43527200	-3.49635700	1.11048900
C	-4.90387800	-2.32561200	0.53375200
H	-4.03085700	-0.48603300	1.24698200
C	-3.47701100	-4.26614300	0.58967200
H	-1.47560400	-3.94469500	1.34161900
C	-4.72191500	-3.69812600	0.29443100
H	-5.86487900	-1.86703300	0.31301700
H	-3.31218500	-5.32555500	0.41023700
C	-5.85401900	-4.54058500	-0.24396500
H	-6.56202000	-4.80432500	0.55288900
H	-6.42038700	-4.00587500	-1.01453900
H	-5.48616700	-5.47512200	-0.67897700

6c

C	3.15460300	-2.78129300	-1.98829000
C	2.54617100	-1.65265100	-1.75981200
C	3.76915400	-3.92129100	-2.21041500
H	1.46165300	-1.59646100	-1.69152900
H	3.09984300	-0.72328300	-1.64264800
H	4.08382500	-4.55838500	-1.38719800
C	4.06378300	-4.39775400	-3.58779700
O	3.78920100	-3.82536900	-4.62189400
O	4.69934800	-5.59372100	-3.53017200
C	5.04824400	-6.16907000	-4.81538500
H	5.64774700	-5.43907200	-5.36808400
H	4.12888500	-6.34103500	-5.38335100
C	5.80175000	-7.44780200	-4.56756900
C	5.15767300	-8.68658000	-4.66272200
C	7.16046000	-7.41430000	-4.22650700
C	5.85589000	-9.87166500	-4.42325500
H	4.10340200	-8.72214500	-4.92734400

C	7.86055900	-8.59536500	-3.98307600
H	7.66899100	-6.45597500	-4.15087300
C	7.20871700	-9.82749600	-4.08226900
H	5.34438600	-10.82704200	-4.50411600
H	8.91443200	-8.55612000	-3.72076200
H	7.75457600	-10.74885700	-3.89740500

A

C	0.59507300	-0.33458500	-1.70656300
H	0.18646400	-0.86191100	-2.57482000
H	-0.06262800	0.50957900	-1.47209200
H	1.57943800	0.05785200	-1.98254900
C	0.69266500	-1.27894900	-0.50994800
C	1.09000000	-0.58306700	0.81896800
C	2.39916600	0.21267500	0.70799200
H	2.24321000	1.08770300	0.06598100
H	3.14030400	-0.43657800	0.22845200
P	3.04076200	0.86866000	2.35044100
C	3.52077400	-0.70932600	3.20073000
C	2.68886200	-1.16127600	4.23861500
C	4.65341300	-1.46924600	2.86261000
C	2.97731300	-2.35114400	4.91312100
H	1.81001700	-0.58353700	4.51351900
C	4.94631000	-2.65033600	3.54465900
H	5.31540100	-1.12916800	2.07073100
C	4.10704800	-3.09477600	4.57067000
H	2.31963300	-2.69102200	5.70876900
H	5.82872200	-3.22533100	3.27488500
H	4.33582800	-4.01594200	5.10055000
C	4.69584800	1.53714600	1.84982700
C	5.53422900	2.00863700	2.87857300
C	5.12953400	1.69689800	0.52422500
C	6.76174900	2.60417300	2.59479100
H	5.21965200	1.90226700	3.91451600
C	6.35661100	2.30441300	0.23643200
H	4.51547300	1.34754100	-0.29941800
C	7.17751600	2.75913500	1.26851200
H	7.39381700	2.95150200	3.40827000
H	6.67180900	2.41247800	-0.79865100
H	8.13244300	3.22715700	1.04442400
O	1.64698400	-2.31370600	-0.75600800
Si	1.40743500	-3.89655200	-1.26333700
C	2.61581600	-4.96472700	-0.21940900
C	2.55401600	-6.44222000	-0.66254000
H	1.54641600	-6.86262900	-0.55258400
H	2.86529300	-6.57171000	-1.70533600
H	3.23077300	-7.04843800	-0.04297800
C	2.26529200	-4.87934100	1.28102300
H	2.99916300	-5.44812700	1.87025200
H	2.28379800	-3.84815700	1.65002700
H	1.27767600	-5.30308100	1.49521000
C	4.05194900	-4.43343500	-0.42625700
H	4.14962000	-3.39768300	-0.08285900
H	4.76498100	-5.04219900	0.14894000
H	4.35717300	-4.47286800	-1.47862000
N	-0.03790100	0.27601700	1.24746800

H	0.13914500	1.28169200	1.21814700
H	1.21950100	-1.38661000	1.54912000
H	-0.29291500	-1.72186400	-0.33222700
C	1.88952500	-3.99039600	-3.09523200
C	1.72923400	-5.15450900	-3.87428500
C	2.47934200	-2.87541800	-3.71998700
C	2.12513100	-5.19896300	-5.21264700
H	1.28706900	-6.04603200	-3.43626100
C	2.87973300	-2.91333600	-5.05765200
H	2.63041700	-1.96611300	-3.14593400
C	2.70102000	-4.07559000	-5.80923700
H	1.98522100	-6.11067800	-5.78827700
H	3.33219400	-2.03527100	-5.51239600
H	3.01044000	-4.10771800	-6.85089300
C	-0.41081800	-4.37396500	-1.01817600
C	-1.20624100	-4.88306900	-2.06273800
C	-1.03090300	-4.19613700	0.23758500
C	-2.54658400	-5.22175400	-1.86193900
H	-0.78095500	-5.00914400	-3.05400800
C	-2.37343900	-4.52172600	0.44055500
H	-0.47801000	-3.77427900	1.07145300
C	-3.13347100	-5.04399000	-0.60824600
H	-3.13301800	-5.61756200	-2.68770300
H	-2.81999000	-4.36045600	1.41804900
H	-4.17759900	-5.30362200	-0.45121100
S	-0.95710600	-0.17395600	2.57438300
O	-0.52155800	0.48886600	3.81397000
O	-1.04146000	-1.64143200	2.53146900
C	-2.55333600	0.52415600	2.14652400
C	-3.03586700	1.62345800	2.85642900
C	-3.30799700	-0.05802200	1.12408700
C	-4.28206700	2.15619900	2.52020800
H	-2.44393700	2.04063500	3.66410300
C	-4.54931600	0.48486000	0.80488700
H	-2.93128500	-0.92939700	0.59792300
C	-5.05338200	1.60322000	1.48948800
H	-4.66284700	3.01230600	3.07163600
H	-5.14179200	0.03211900	0.01341800
C	-6.38353500	2.20643700	1.10591900
H	-6.82665400	2.76146900	1.93899600
H	-6.26681400	2.90746500	0.26909600
H	-7.09637900	1.43632200	0.79168400
C	2.38984200	5.19683900	0.99463300
C	3.58006500	4.96998700	1.46814000
C	1.18296900	5.42696900	0.52313100
H	3.76792300	4.96107900	2.53950900
H	4.42231900	4.74251800	0.81845900
H	0.85166400	6.43436600	0.28322800
C	0.21058900	4.33120000	0.31054700
O	0.41706000	3.14153800	0.48238600
O	-0.96899300	4.82976900	-0.11355600
C	-2.02439700	3.86439800	-0.34370200
H	-1.72286200	3.19283100	-1.15344700
H	-2.14168100	3.26024100	0.56173900
C	-3.28931700	4.60909300	-0.68243700
C	-4.04628600	4.24287100	-1.80016300

C	-3.74876900	5.64852900	0.13846500
C	-5.24771200	4.89499800	-2.09094000
H	-3.69456700	3.44372000	-2.44856900
C	-4.94138600	6.30761500	-0.15548000
H	-3.16071600	5.94690200	1.00194800
C	-5.69698000	5.92995900	-1.26961400
H	-5.82389700	4.60101000	-2.96419300
H	-5.28369100	7.11588700	0.48530600
H	-6.62718900	6.44337800	-1.49753600

Ts1

C	-0.78194200	-0.06663900	-1.03146600
H	-1.27245400	-0.66671500	-1.80486900
H	-1.52224100	0.61041000	-0.59208800
H	-0.00591800	0.53494400	-1.51709400
C	-0.18534800	-0.97418900	0.04232800
C	0.33764100	-0.22293800	1.29665800
C	1.36166300	0.86336000	0.92370400
H	0.86293300	1.68466700	0.40058100
H	2.09212700	0.41952400	0.23895200
P	2.27723700	1.63667800	2.34540400
C	3.10722100	0.22178200	3.19788700
C	2.48375400	-0.34060200	4.32462500
C	4.32433000	-0.32080300	2.74700500
C	3.06541000	-1.42792500	4.98134900
H	1.53529900	0.05109800	4.68124200
C	4.90279200	-1.40165800	3.41181800
H	4.82261000	0.10250900	1.88049000
C	4.27488200	-1.95675100	4.53026700
H	2.56693600	-1.85838400	5.84514800
H	5.84250100	-1.81302000	3.05276600
H	4.72741400	-2.79987700	5.04533800
C	3.64587100	2.56183400	1.55282500
C	4.73593400	2.96583000	2.34584800
C	3.58835600	2.99351200	0.21861600
C	5.74906400	3.75854900	1.81134700
H	4.79475600	2.65195400	3.38449200
C	4.60287500	3.79446700	-0.31312300
H	2.75736900	2.70798400	-0.41698200
C	5.68496700	4.17602600	0.47861100
H	6.58720400	4.05440600	2.43649000
H	4.54249900	4.11779600	-1.34869900
H	6.47366800	4.79721300	0.06302100
O	0.90296400	-1.74049600	-0.48048100
Si	0.92915700	-3.33003300	-1.02563600
C	2.54239500	-4.09540300	-0.31206700
C	2.72844700	-5.54058400	-0.82186600
H	1.89257800	-6.18908500	-0.53065300
H	2.82833800	-5.58216200	-1.91234400
H	3.64370000	-5.97546800	-0.39448100
C	2.50792800	-4.11347500	1.23051800
H	3.46321000	-4.49231500	1.62234600
H	2.35831800	-3.11294400	1.65135900
H	1.71717100	-4.76718300	1.61440600
C	3.74286600	-3.23781800	-0.77167400
H	3.66707400	-2.20948800	-0.40042100

H	4.68035800	-3.66332800	-0.38418700
H	3.82505000	-3.19749700	-1.86406000
N	-0.80336200	0.32822000	2.04209700
H	-1.00301800	1.33533900	1.94204700
H	0.82536600	-0.98161800	1.91367700
H	-0.96823500	-1.64560000	0.40873600
C	1.02816200	-3.27915000	-2.91722900
C	0.97304500	-4.43616600	-3.72113100
C	1.21304500	-2.04793600	-3.57407200
C	1.08553100	-4.36646700	-5.11122000
H	0.83702200	-5.41243700	-3.26201200
C	1.32827100	-1.97111200	-4.96395100
H	1.27168200	-1.13715300	-2.98563200
C	1.26253000	-3.13103000	-5.73716800
H	1.03544600	-5.27624600	-5.70438900
H	1.46962800	-1.00513600	-5.44276300
H	1.35029900	-3.07396100	-6.81918200
C	-0.63672200	-4.22755600	-0.44864100
C	-1.50396000	-4.88031400	-1.34561900
C	-0.99109200	-4.24182200	0.91802100
C	-2.65405900	-5.53810800	-0.90238400
H	-1.28837100	-4.86883400	-2.40990400
C	-2.14400000	-4.89048500	1.36515000
H	-0.38321700	-3.72178100	1.65207900
C	-2.97540900	-5.54791900	0.45549400
H	-3.30132100	-6.03655900	-1.61987500
H	-2.38841200	-4.87655800	2.42410900
H	-3.87075400	-6.05811400	0.80215600
S	-1.30882100	-0.36408500	3.46663100
O	-0.85777700	0.40070100	4.64406200
O	-0.96636400	-1.79325900	3.37829400
C	-3.08722200	-0.16958700	3.37529400
C	-3.69685600	0.89915400	4.03080800
C	-3.84217600	-1.09784900	2.65325500
C	-5.08187200	1.04480200	3.94668600
H	-3.09407900	1.59495900	4.60397400
C	-5.22313700	-0.93862400	2.58553800
H	-3.35507500	-1.93764300	2.16803100
C	-5.86412100	0.13590100	3.22326200
H	-5.56150200	1.87647800	4.45683700
H	-5.81601100	-1.66261000	2.03148200
C	-7.36044700	0.31153200	3.11486500
H	-7.74518100	0.96971300	3.90022300
H	-7.63568700	0.75566200	2.14904500
H	-7.88320700	-0.64856200	3.18898500
C	0.96096600	3.35714200	3.02851900
C	0.78183200	3.25648300	4.33598300
C	0.66674200	4.15861700	1.94299100
H	0.18995600	4.01709400	4.84368100
H	1.16698300	2.44294400	4.93833800
H	1.34277600	4.95594900	1.64973200
C	-0.49505100	3.93561700	1.13511600
O	-1.31424500	3.00788400	1.22090800
O	-0.64654900	4.90768900	0.18445700
C	-1.80579800	4.79387200	-0.65073700
H	-1.74695900	3.87417800	-1.24476300

H	-2.69497900	4.70057100	-0.01490700
C	-1.89355800	6.00971000	-1.54250200
C	-1.45915200	7.26780700	-1.10699300
C	-2.46722000	5.89480800	-2.81437500
C	-1.59812700	8.38703100	-1.92864600
H	-1.00042200	7.36002300	-0.12780600
C	-2.61495200	7.01518500	-3.63388900
H	-2.79916600	4.92064700	-3.16779000
C	-2.17928100	8.26601100	-3.19302400
H	-1.25144400	9.35656100	-1.58000900
H	-3.06148000	6.90830900	-4.61913600
H	-2.28723400	9.13886100	-3.83152800

B

C	-0.58331200	-0.83576900	-1.56961000
H	-0.87309400	-1.58683700	-2.31153400
H	-1.46003500	-0.22332100	-1.33702800
H	0.18404600	-0.19845000	-2.02303900
C	-0.07752200	-1.52577600	-0.30596800
C	0.23395900	-0.56526600	0.87408900
C	1.28914900	0.47584800	0.46085300
H	0.86219100	1.20873800	-0.23103100
H	2.07568300	-0.06443100	-0.07536100
P	2.13017600	1.44990000	1.78891600
C	3.03874000	0.30991400	2.87758100
C	2.32822400	-0.49422900	3.78902200
C	4.43601100	0.19895500	2.79051000
C	3.02152400	-1.40180500	4.59092200
H	1.25006400	-0.40779300	3.89031700
C	5.11621500	-0.71258100	3.59815200
H	4.99472300	0.82563700	2.10378600
C	4.41013700	-1.51455800	4.49638100
H	2.46783400	-2.01764700	5.29342800
H	6.19737300	-0.78980400	3.52724600
H	4.94202100	-2.22303800	5.12551000
C	3.32324500	2.51450700	0.91004600
C	3.47176900	3.84677900	1.32594500
C	4.08986000	2.03298800	-0.16470500
C	4.37805600	4.68300800	0.67402100
H	2.87426800	4.22142800	2.15107400
C	4.99467400	2.87553300	-0.81109200
H	3.99098600	1.00514300	-0.50141100
C	5.13843700	4.20032200	-0.39306700
H	4.48460300	5.71404400	0.99836700
H	5.58285800	2.49672300	-1.64199200
H	5.84073900	4.85573900	-0.90057400
O	1.12861900	-2.25502900	-0.55391100
Si	1.34620300	-3.84712700	-1.05351000
C	2.70582800	-4.56021700	0.10577600
C	3.07022100	-6.00356300	-0.30350200
H	2.20162800	-6.67284900	-0.26831200
H	3.49400300	-6.04845000	-1.31304000
H	3.82531700	-6.41089400	0.38439200
C	2.22753300	-4.56616200	1.57316100
H	3.03861700	-4.91203800	2.23062000
H	1.93515400	-3.56618400	1.91410200

H	1.37737900	-5.24025600	1.72377500
C	3.96605400	-3.67239700	0.00010200
H	3.76089200	-2.64436200	0.32058200
H	4.76188900	-4.06791100	0.64812700
H	4.35908500	-3.63746800	-1.02265300
N	-1.01472100	0.07967200	1.33680200
H	-0.90397400	1.08985000	1.53994500
H	0.66559700	-1.19372700	1.66326000
H	-0.85729300	-2.20013400	0.05747900
C	1.99127700	-3.78786200	-2.83404000
C	2.23162200	-4.94913100	-3.59657500
C	2.29433200	-2.55110800	-3.43367800
C	2.74210600	-4.87861300	-4.89423600
H	2.01398700	-5.92902400	-3.17889000
C	2.80619900	-2.47285200	-4.73082200
H	2.12609400	-1.63562100	-2.87393800
C	3.03082900	-3.63781800	-5.46573300
H	2.91346800	-5.79187700	-5.45858300
H	3.02765100	-1.50231900	-5.16835700
H	3.42733700	-3.58030500	-6.47620800
C	-0.29083000	-4.79035200	-0.95994100
C	-0.83883200	-5.43788200	-2.08464000
C	-1.02764600	-4.85085800	0.24273300
C	-2.04855100	-6.13156500	-2.00996700
H	-0.32164100	-5.39478200	-3.03852800
C	-2.24178500	-5.53471100	0.31962500
H	-0.67370700	-4.34000900	1.13338100
C	-2.75186100	-6.18435400	-0.80586900
H	-2.44403700	-6.62397400	-2.89494000
H	-2.78972800	-5.54991600	1.25775900
H	-3.69614400	-6.71974500	-0.74700400
S	-1.75593500	-0.69308400	2.65433600
O	-0.92928500	-0.56608400	3.87027800
O	-2.11884400	-2.04691100	2.20683000
C	-3.22119400	0.30243300	2.85336100
C	-3.12775500	1.54689800	3.48189300
C	-4.44044400	-0.19066900	2.38807000
C	-4.28334300	2.31098500	3.62498600
H	-2.18087500	1.93172800	3.84505800
C	-5.58684200	0.58409000	2.55767900
H	-4.48270800	-1.16513900	1.91352600
C	-5.52618200	1.84395300	3.17038600
H	-4.20471700	3.28601700	4.09793900
H	-6.54337400	0.20434800	2.20711700
C	-6.76619900	2.69348800	3.32000500
H	-6.79924500	3.18333800	4.29939700
H	-6.79227500	3.48677100	2.56127000
H	-7.67779500	2.09858200	3.20507600
C	0.94862200	2.49520600	2.67273000
C	1.14308700	2.63198300	4.01754700
C	-0.02455800	3.08744700	1.79668100
H	0.49318500	3.29111500	4.58001000
H	1.90207400	2.07502200	4.55198400
H	0.12776800	3.06378700	0.72284100
C	-1.00860700	4.00101500	2.26665600
O	-1.30795200	4.26146400	3.43781800

O	-1.68109300	4.60083300	1.20604500
C	-2.72866400	5.50115700	1.55221700
H	-3.66541300	4.94202900	1.68937600
H	-2.49393100	5.96753100	2.51626800
C	-2.89612400	6.54075900	0.46696200
C	-1.82193000	6.93837200	-0.33775000
C	-4.13902000	7.16020000	0.28485100
C	-1.98756100	7.93670900	-1.29915900
H	-0.86211000	6.44843500	-0.21098700
C	-4.30535200	8.16329800	-0.67106000
H	-4.98565400	6.85259400	0.89587400
C	-3.22803200	8.55541400	-1.46828600
H	-1.14457200	8.23137300	-1.91963600
H	-5.27801100	8.63195500	-0.79873500
H	-3.35593300	9.33196000	-2.21803200

5a

C	0.05204000	0.78693800	0.54545400
C	1.51086400	1.26105200	0.30119600
C	0.50961300	3.27097000	0.17598400
S	-0.97026200	2.30352600	0.46576800
N	1.64077300	2.64540700	0.12200100
C	0.41122000	4.72778000	0.00306800
C	-0.81873600	5.40279700	0.06901300
C	1.58758600	5.46170700	-0.23718500
C	-0.87246200	6.78384100	-0.10335100
H	-1.73607700	4.85173600	0.25571100
C	1.52867500	6.84101700	-0.40871200
H	2.53018600	4.92796200	-0.28516600
C	0.29988000	7.50510200	-0.34265300
H	-1.82822800	7.29707800	-0.05083300
H	2.44110100	7.40053400	-0.59413800
H	0.25598600	8.58249400	-0.47705500
C	-0.36013700	-0.30285100	-0.44800600
H	0.37077200	-1.11565700	-0.38837800
H	-1.35236600	-0.70106100	-0.21632600
H	-0.37037300	0.08258100	-1.47220600
H	-0.00004800	0.39916000	1.56841100
O	2.43143900	0.47448200	0.26664400

C

C	-1.48487100	-0.86564300	0.00661000
H	-2.16106100	-1.53188600	-0.53815800
H	-2.04874200	-0.38433900	0.81200100
H	-1.14274100	-0.09068200	-0.68743700
C	-0.31336800	-1.65488500	0.58783000
C	0.60332100	-0.82438500	1.53049400
C	1.13896300	0.42619900	0.80667700
H	0.36889000	1.20202300	0.78328900
H	1.35845100	0.15009100	-0.22819400
P	2.66894000	1.26382500	1.46197800
C	3.23382200	0.49241500	3.00891100
C	2.55925300	0.77635900	4.20925700
C	4.33315100	-0.38224200	3.01427800
C	2.98054200	0.17624900	5.39522700
H	1.71623400	1.45772900	4.22203100

C	4.74791500	-0.97236500	4.20746900
H	4.87017200	-0.59559100	2.09628700
C	4.07082500	-0.69497500	5.39645500
H	2.43810900	0.37985300	6.31228400
H	5.59929700	-1.64709400	4.20563800
H	4.39149300	-1.16044800	6.32446000
C	3.98048800	1.03133900	0.21619100
C	4.08874900	-0.16079200	-0.51611200
C	4.91200900	2.06184800	0.01415900
C	5.12203000	-0.32052300	-1.44106400
H	3.37099700	-0.96355800	-0.37407100
C	5.94057300	1.89436400	-0.91261100
H	4.82454900	2.98420700	0.58121000
C	6.04739900	0.70597900	-1.63929200
H	5.19688900	-1.24441300	-2.00691200
H	6.65613100	2.69642100	-1.06948100
H	6.84942600	0.58093500	-2.36168900
O	0.51987300	-2.20791100	-0.44264900
Si	0.33568500	-3.70356400	-1.20139100
C	2.11487400	-4.28189400	-1.64899200
C	2.06859800	-5.68835600	-2.28581500
H	1.64161800	-6.43341300	-1.60382700
H	1.48390000	-5.69936200	-3.21276700
H	3.08563200	-6.01993500	-2.54024000
C	3.01758800	-4.33583600	-0.39858800
H	4.04662600	-4.59512900	-0.68729800
H	3.05237400	-3.37515900	0.12769300
H	2.68120800	-5.09654800	0.31399300
C	2.72343300	-3.29446900	-2.66977800
H	2.77224300	-2.27323900	-2.27497400
H	3.74746800	-3.60350200	-2.92760400
H	2.14595800	-3.26040200	-3.60012400
N	-0.11972100	-0.41829700	2.73567200
H	-0.30750800	0.57296000	2.93065300
H	1.43089700	-1.48285300	1.81502600
H	-0.70995400	-2.47101300	1.19962800
C	-0.64803900	-3.46128900	-2.80195400
C	-1.10434100	-4.53864600	-3.58902800
C	-0.88013300	-2.16271600	-3.29370500
C	-1.77407000	-4.32930000	-4.79633600
H	-0.93858900	-5.56179600	-3.26097800
C	-1.54752900	-1.94598700	-4.50112100
H	-0.52778600	-1.30832500	-2.72395600
C	-2.00000800	-3.03005500	-5.25475700
H	-2.11748900	-5.18056700	-5.37880000
H	-1.71182500	-0.93073600	-4.85369700
H	-2.52076400	-2.86475600	-6.19443600
C	-0.54122700	-4.90122900	-0.02367700
C	-1.72700700	-5.57621200	-0.37262900
C	-0.01447600	-5.14600500	1.26266600
C	-2.34130600	-6.47450900	0.50330300
H	-2.18844500	-5.39491800	-1.33860300
C	-0.62576000	-6.04012100	2.14388300
H	0.86859600	-4.61435200	1.60414200
C	-1.78806900	-6.71449800	1.76205200
H	-3.25341200	-6.98384300	0.20213100

H	-0.19659600	-6.20028800	3.12925100
H	-2.26238700	-7.41557300	2.44419100
S	-0.42422300	-1.43855200	4.01162300
O	-0.20674100	-0.68485300	5.24982500
O	0.28766800	-2.69483700	3.73604900
C	-2.18896600	-1.77301800	3.93595100
C	-3.08658500	-0.77353600	4.32635000
C	-2.64729400	-3.01232600	3.49432500
C	-4.45324600	-1.02471500	4.25640200
H	-2.71586400	0.18261900	4.68216200
C	-4.02268000	-3.24698600	3.43403900
H	-1.94001700	-3.78312600	3.20878800
C	-4.94437500	-2.26358200	3.81118600
H	-5.15323500	-0.24817600	4.55627600
H	-4.37927100	-4.21371900	3.08702500
C	-6.43125900	-2.52639800	3.76361600
H	-6.97011200	-1.69459200	3.29517400
H	-6.65949700	-3.43721000	3.20130100
H	-6.84401200	-2.64860100	4.77357000
C	2.36110700	3.04465400	1.64594600
C	1.54441300	3.57586800	0.67845700
C	3.09795300	3.67397400	2.68961600
H	1.41354400	4.64947800	0.62594400
H	1.07176600	2.97248500	-0.08796300
H	3.74754300	3.09445200	3.33059000
C	2.96602200	5.05381400	2.97808800
O	2.19187100	5.87424600	2.46062500
O	3.83603100	5.45236400	3.99474900
C	-0.77756400	4.72838400	3.20971700
C	-0.97279500	3.25615000	2.82822400
C	-2.31138600	4.13363900	1.26388300
S	-1.88973600	5.64808900	2.07455500
N	-1.78536400	3.03192700	1.72156500
C	-3.23507700	4.13152400	0.11922600
C	-3.70073700	5.32487300	-0.45798500
C	-3.66619800	2.90045800	-0.40819400
C	-4.58040700	5.28816500	-1.53704300
H	-3.36807900	6.28353200	-0.07075700
C	-4.54682600	2.86858800	-1.48503000
H	-3.30179800	1.98537100	0.04485200
C	-5.00637100	4.06118200	-2.05194400
H	-4.93170700	6.21674600	-1.97753700
H	-4.87703800	1.91327200	-1.88334500
H	-5.69372900	4.03461900	-2.89318600
C	-1.02974100	5.00246800	4.69373300
H	-2.06961500	4.80046200	4.97149100
H	-0.37534000	4.34773500	5.27628800
H	-0.78632400	6.03708800	4.94797000
O	-0.45949000	2.35009700	3.47625500
C	3.65756300	6.78389900	4.47089000
H	3.38042800	7.44316000	3.64398300
H	4.64369500	7.08193300	4.84765400
C	2.62810000	6.87974400	5.58163900
C	2.47743200	5.84242900	6.51093900
C	1.84384400	8.03034400	5.72328600
C	1.56584700	5.95645500	7.56165600

H	3.07324400	4.94198400	6.39451000
C	0.93465200	8.15035500	6.77712400
H	1.94182800	8.83649500	4.99926500
C	0.79321400	7.11251800	7.70099700
H	1.45939700	5.14234900	8.27455400
H	0.33289100	9.05102800	6.87196100
H	0.08464600	7.20211900	8.52048700
H	0.24803200	5.01637200	2.93940600

Ts2

C	-1.85403500	-0.85955800	0.00199900
H	-2.62090800	-1.52907900	-0.39973900
H	-2.31863500	-0.20620800	0.74756700
H	-1.49391200	-0.23129200	-0.81998000
C	-0.71024000	-1.67076100	0.60955300
C	0.29562800	-0.82684300	1.44756200
C	0.91088600	0.27610600	0.57191800
H	0.14251800	1.00571100	0.29456700
H	1.27457600	-0.19132500	-0.34601000
P	2.27332100	1.33002400	1.26698800
C	3.20054500	0.55428700	2.63153200
C	2.59826100	0.34531900	3.88524000
C	4.54303300	0.18068300	2.44162500
C	3.32495600	-0.24082500	4.92027700
H	1.56579900	0.61679300	4.05841000
C	5.26135900	-0.40350800	3.48453000
H	5.03177300	0.34608100	1.48878700
C	4.65432000	-0.61689800	4.72305400
H	2.83720800	-0.40536900	5.87583300
H	6.29726500	-0.68900600	3.32535900
H	5.21668000	-1.07392900	5.53261000
C	3.46180200	1.57882600	-0.10220900
C	3.82677100	0.50726800	-0.93488200
C	4.06152400	2.83584500	-0.28679600
C	4.76932600	0.69376600	-1.94600600
H	3.39028300	-0.47806900	-0.79868800
C	5.00675000	3.01158300	-1.29868600
H	3.77844500	3.67650700	0.33941300
C	5.35892100	1.94645300	-2.12947000
H	5.03779900	-0.13893600	-2.58958000
H	5.46161100	3.98779200	-1.43878500
H	6.08992600	2.09145300	-2.92005100
O	0.05248000	-2.32307800	-0.41320700
Si	0.07128000	-3.93101300	-0.90562400
C	1.90511600	-4.26152900	-1.38942100
C	2.08636100	-5.72042000	-1.86143500
H	1.80640600	-6.44008100	-1.08243100
H	1.49228400	-5.93846900	-2.75598500
H	3.13943200	-5.90662000	-2.11766600
C	2.84727400	-4.00933400	-0.19364200
H	3.89511600	-4.12752300	-0.50670900
H	2.73475500	-2.99650800	0.21173800
H	2.67209300	-4.71892500	0.62216500
C	2.29465500	-3.30853800	-2.54144200
H	2.19477600	-2.25618000	-2.24868900
H	3.34115400	-3.47716100	-2.83585000

H	1.67176800	-3.46397600	-3.42941100
N	-0.27406200	-0.22860600	2.65237100
H	-0.83625100	0.64303200	2.57332500
H	1.08376200	-1.51409300	1.76843100
H	-1.12341500	-2.42284100	1.29079400
C	-1.01956400	-4.10389500	-2.44338100
C	-1.27969400	-5.34625400	-3.05764100
C	-1.55928300	-2.95740100	-3.05629000
C	-2.04985700	-5.43990900	-4.21848200
H	-0.88056000	-6.26135800	-2.62714100
C	-2.33051500	-3.04394900	-4.21756300
H	-1.36943500	-1.98197800	-2.61931000
C	-2.57967900	-4.28687000	-4.80107500
H	-2.23580000	-6.41251500	-4.66718600
H	-2.73603900	-2.14041400	-4.66606000
H	-3.18049200	-4.35736700	-5.70415400
C	-0.53746700	-5.05234800	0.49268100
C	-1.59838900	-5.96223000	0.31452600
C	0.06502600	-5.00618300	1.76746000
C	-2.01904300	-6.80474400	1.34584900
H	-2.11408300	-6.01137600	-0.63975900
C	-0.35352300	-5.84350700	2.80343800
H	0.85588200	-4.29285200	1.97733300
C	-1.39278600	-6.75220600	2.59220700
H	-2.83841200	-7.49873900	1.17553400
H	0.12917800	-5.77625800	3.77467100
H	-1.71663400	-7.40903600	3.39571700
S	-0.66407900	-1.14944000	3.98758500
O	-0.54633900	-0.24737300	5.14132800
O	0.14680200	-2.37452100	3.91533400
C	-2.39556900	-1.60818900	3.84937500
C	-3.36845700	-0.60473500	3.76287900
C	-2.74825700	-2.95632300	3.86402400
C	-4.70745600	-0.97502500	3.68645700
H	-3.08233400	0.44251100	3.72456600
C	-4.09883300	-3.30370100	3.79088400
H	-1.97899200	-3.71797200	3.92225800
C	-5.09571000	-2.32532600	3.70442100
H	-5.46654000	-0.20023200	3.60873400
H	-4.37669400	-4.35473100	3.79665800
C	-6.55699100	-2.70192600	3.63648900
H	-7.05796700	-2.20679200	2.79618300
H	-6.68798300	-3.78210100	3.51909400
H	-7.08623900	-2.39914400	4.54910600
C	1.59220400	2.96793000	1.68344800
C	0.99688600	3.60008900	0.64606200
C	1.70652600	3.54430800	3.02673100
H	0.58530300	4.59388500	0.77606800
H	0.94321400	3.18546600	-0.35691700
H	2.02771600	2.87852700	3.82318100
C	2.35646000	4.86179100	3.11763800
O	2.60906000	5.63431300	2.20335600
O	2.63640700	5.16458000	4.42250700
H	0.33716600	3.78902600	3.33260500
C	-1.02044200	3.92421700	3.64415800
C	-1.72564100	3.26268200	2.53938900

C	-2.14372800	5.38999500	1.94375100
S	-1.37318000	5.67975300	3.50909400
N	-2.24133800	4.14367700	1.57985300
C	-2.61972100	6.50083200	1.10413100
C	-2.39630200	7.84516900	1.44506800
C	-3.32415900	6.20710300	-0.07799900
C	-2.86648700	8.86992800	0.62655100
H	-1.84407200	8.09131700	2.34773400
C	-3.79413500	7.23400300	-0.89150800
H	-3.48938100	5.16598400	-0.33300500
C	-3.56748200	8.56911200	-0.54376200
H	-2.68286200	9.90491800	0.90189800
H	-4.33891600	6.99367800	-1.80082600
H	-3.93380700	9.36969800	-1.18096600
C	-1.06835300	3.31210700	5.03403500
H	-2.04539300	3.45180400	5.51380100
H	-0.87936000	2.23696200	4.96266100
H	-0.30176200	3.74401300	5.68796600
O	-1.84548100	2.02082100	2.41024500
C	3.21735200	6.46173600	4.64404500
H	2.60638900	7.21177400	4.12845000
H	4.21637100	6.49881700	4.19516100
C	3.27939900	6.71956600	6.12910200
C	2.15858000	6.50180000	6.94230600
C	4.44851000	7.22063800	6.71123600
C	2.20908400	6.78017600	8.30734700
H	1.25013600	6.10335600	6.49971600
C	4.49929300	7.50889100	8.07727300
H	5.32725000	7.38536100	6.09138400
C	3.37937900	7.28783300	8.87921200
H	1.33365200	6.60245000	8.92664700
H	5.41567700	7.89792500	8.51369400
H	3.41713700	7.50606600	9.94329400

D

C	-0.73732300	0.39683100	0.87446600
H	-1.40432000	0.13073000	0.04935700
H	-1.33898400	0.74618200	1.72056200
H	-0.10360600	1.22439300	0.53830200
C	0.11408600	-0.80754500	1.26932000
C	0.89969400	-0.62388700	2.59847600
C	1.68577000	0.69746000	2.56240000
H	1.00222800	1.51352900	2.83218500
H	2.09416000	0.89053500	1.56655200
P	3.10506500	0.92778700	3.69236200
C	2.82797400	0.48869000	5.43488400
C	2.60494000	-0.86163400	5.75352300
C	2.85730800	1.44961500	6.45516800
C	2.39501300	-1.23713800	7.07765400
H	2.55897200	-1.62064900	4.97964100
C	2.66408700	1.06063200	7.78091700
H	3.02241800	2.49428700	6.22135200
C	2.42900800	-0.27862400	8.09235900
H	2.18398000	-2.27625700	7.30745100
H	2.68467700	1.81047300	8.56638700
H	2.26139400	-0.57500800	9.12393400

C	4.52476500	-0.08230500	3.15555800
C	5.59026700	-0.29297700	4.04907500
C	4.58050100	-0.63900100	1.86857100
C	6.70137900	-1.03536400	3.65267100
H	5.54473600	0.10739800	5.05760900
C	5.69198800	-1.39088100	1.48337400
H	3.76563000	-0.50016100	1.16795400
C	6.75232100	-1.58580100	2.36955100
H	7.51948300	-1.19343500	4.34943800
H	5.71965200	-1.82671700	0.48940300
H	7.61407700	-2.17322100	2.06493500
O	1.08902500	-1.10539400	0.25889000
Si	0.96194600	-2.09193800	-1.09580800
C	1.61500800	-3.85316200	-0.65771900
C	1.46697700	-4.80690900	-1.86370900
H	0.42518400	-4.89516200	-2.19368700
H	2.06871500	-4.48322600	-2.72061600
H	1.81313600	-5.81431000	-1.58986200
C	0.85197900	-4.45870900	0.54252200
H	1.29400900	-5.43040500	0.80770200
H	0.89782100	-3.83144200	1.43996700
H	-0.20171300	-4.64426300	0.30728800
C	3.10978300	-3.75400400	-0.28046700
H	3.26244900	-3.11697800	0.59814600
H	3.50342500	-4.75114500	-0.03470800
H	3.71372200	-3.35181800	-1.10264600
N	0.02918500	-0.59216700	3.76778200
H	-0.19518100	0.34798700	4.18903300
H	1.56952400	-1.48714900	2.67444400
H	-0.53770300	-1.67536000	1.42227400
C	2.10520400	-1.25078500	-2.34988600
C	2.31807600	-1.76111700	-3.64654000
C	2.79487700	-0.07438100	-1.99996400
C	3.18266800	-1.13388000	-4.54615600
H	1.79296800	-2.65553700	-3.97278800
C	3.66314200	0.55605000	-2.89402600
H	2.64314600	0.36295700	-1.01825600
C	3.86187600	0.02683300	-4.16996600
H	3.32331000	-1.55001600	-5.54070200
H	4.17700600	1.46476200	-2.59002200
H	4.53508600	0.51677800	-4.86909400
C	-0.81348100	-2.13264100	-1.76027500
C	-1.12339800	-1.56353800	-3.01115800
C	-1.87956900	-2.69933400	-1.03021400
C	-2.42599800	-1.56959800	-3.51497900
H	-0.33718200	-1.10233300	-3.60180400
C	-3.18449600	-2.70123100	-1.52512900
H	-1.70570200	-3.13625200	-0.05116700
C	-3.46097600	-2.13985500	-2.77325700
H	-2.63175400	-1.12325700	-4.48476200
H	-3.98304600	-3.13827100	-0.93130900
H	-4.47625800	-2.14233000	-3.16179900
S	-0.53099000	-1.95685600	4.49170900
O	-0.80815300	-1.62661900	5.89232100
O	0.40071500	-3.05365100	4.15383400
C	-2.10286500	-2.35991900	3.71462300

C	-3.07984500	-1.36939400	3.56635100
C	-2.35175200	-3.67651500	3.32726000
C	-4.31307300	-1.71458000	3.01943700
H	-2.87679000	-0.33861500	3.84682000
C	-3.59618300	-4.00335200	2.78323200
H	-1.57877700	-4.42763100	3.44972300
C	-4.59404500	-3.03337900	2.62600500
H	-5.06626700	-0.94125900	2.89067900
H	-3.79315900	-5.02969600	2.48166400
C	-5.95008700	-3.39241700	2.06378300
H	-6.71355800	-3.40412400	2.85262700
H	-6.27855100	-2.66381200	1.31349500
H	-5.94345200	-4.38320400	1.59767600
C	3.51558200	2.70834300	3.55870500
C	2.54923000	3.58038600	3.88046400
C	4.86578700	3.13641100	3.03045400
H	2.73458100	4.65076200	3.79315000
H	1.55527100	3.26257400	4.21456700
H	5.03699500	4.19378900	3.25977800
C	4.99301300	2.94793100	1.52055400
O	4.21202000	2.33014800	0.82402500
O	6.10021900	3.55027700	1.06331400
H	5.69288200	2.58565800	3.49635200
C	-1.58462200	3.60908100	5.52900000
C	-1.26983300	2.59622300	4.62011700
C	-3.44421100	2.88278600	4.08189800
S	-3.25008100	4.08896400	5.34891800
N	-2.35417000	2.20850900	3.82243500
C	-4.71797100	2.70405200	3.37357700
C	-5.93094600	3.20319000	3.88158300
C	-4.74169100	2.01367000	2.14620300
C	-7.12451600	3.02407000	3.18547700
H	-5.94028500	3.72232400	4.83630200
C	-5.93824300	1.83222600	1.45569700
H	-3.80731900	1.63708000	1.74447300
C	-7.13684800	2.33653200	1.96921700
H	-8.05006200	3.41672100	3.59893600
H	-5.93309800	1.30345700	0.50541400
H	-8.06869100	2.19685000	1.42754300
C	-0.66809900	4.19956400	6.55139400
H	-1.11419100	4.22265600	7.55505900
H	0.22874900	3.57258100	6.59764400
H	-0.35293700	5.22829300	6.31605000
O	-0.10762600	2.02864900	4.48694300
C	6.35119200	3.42153400	-0.36945700
H	6.43465200	2.35685300	-0.60503200
H	5.48313000	3.82066500	-0.90165400
C	7.61022000	4.17547200	-0.69311200
C	7.56143300	5.54539000	-0.98130700
C	8.84937200	3.52350100	-0.69697900
C	8.72988400	6.25202600	-1.26565100
H	6.60270000	6.05879800	-0.98419900
C	10.02013700	4.22706500	-0.98230100
H	8.89572100	2.45924500	-0.47766300
C	9.96161300	5.59306000	-1.26646700
H	8.67934300	7.31374700	-1.49078800

H	10.97556200	3.70964700	-0.98610500
H	10.87203400	6.14156200	-1.49221000

Ts4-*re*

C	0.77830100	-2.05096800	-1.55824400
H	0.78934500	-2.80132200	-2.35556000
H	0.35763600	-2.50153400	-0.65480600
H	0.11756200	-1.23589200	-1.87149100
C	2.19459700	-1.55549800	-1.28303400
C	2.32060700	-0.61903800	-0.04890400
C	1.38173800	0.59624700	-0.19730700
H	0.35849800	0.29358200	0.03320700
H	1.41900400	0.90359100	-1.24459600
P	1.77833200	2.09213100	0.82247100
C	3.03415400	1.71568200	2.09694400
C	4.39145100	1.58212700	1.75327600
C	2.63575200	1.51685900	3.42689300
C	5.33325800	1.26691900	2.73126500
H	4.71904200	1.72762900	0.72843600
C	3.58667800	1.21429800	4.40085900
H	1.58184500	1.54171900	3.67728300
C	4.93265500	1.09140500	4.05717600
H	6.37736800	1.15351600	2.45451400
H	3.26822200	1.04949400	5.42580500
H	5.66775600	0.84167900	4.81711300
C	2.60258900	3.30720200	-0.30329300
C	3.53462500	4.23378400	0.19315800
C	2.15783900	3.44869400	-1.62925300
C	4.03291500	5.25028000	-0.62467500
H	3.87965300	4.16649400	1.21899900
C	2.66208900	4.45873600	-2.44681300
H	1.40181300	2.78348900	-2.03392000
C	3.60510200	5.36044500	-1.94787700
H	4.75311200	5.95594500	-0.22013600
H	2.31330900	4.54250500	-3.47202300
H	3.99832900	6.14673400	-2.58654800
O	2.72729600	-0.83878100	-2.40482200
Si	3.65323400	-1.41237100	-3.68579700
C	4.96651400	-0.04753800	-4.02584500
C	5.91208600	-0.47964800	-5.16739800
H	6.44277500	-1.41012700	-4.93153200
H	5.37519300	-0.62347100	-6.11206500
H	6.67120100	0.29679700	-5.34162000
C	5.80860900	0.23126300	-2.76291300
H	6.50784800	1.05864200	-2.95332600
H	5.18115400	0.51717900	-1.91101900
H	6.40650800	-0.63809500	-2.46856200
C	4.24278000	1.25402500	-4.43551200
H	3.57995300	1.61376400	-3.64070500
H	4.97765700	2.04769600	-4.63555700
H	3.64333700	1.12106600	-5.34344500
N	2.00601600	-1.33479800	1.18740700
H	1.27271400	-0.94929500	1.81209900
H	3.36350400	-0.28552200	-0.03441600
H	2.83366100	-2.41809000	-1.07078800
C	2.52063200	-1.59284500	-5.19406400

C	2.96412200	-2.12158900	-6.42368000
C	1.18939300	-1.14007000	-5.13161300
C	2.11816800	-2.20342400	-7.53154000
H	3.98479500	-2.48238400	-6.52470000
C	0.33791000	-1.21793700	-6.23601800
H	0.81511300	-0.71956900	-4.20316200
C	0.80022700	-1.75159300	-7.43983000
H	2.48835300	-2.61947400	-8.46530200
H	-0.68642800	-0.86245000	-6.15535200
H	0.13954200	-1.81454800	-8.30076100
C	4.44187600	-3.07494800	-3.23180800
C	4.26824200	-4.22654500	-4.02368400
C	5.23790500	-3.20227500	-2.07316600
C	4.87942500	-5.43884600	-3.69483400
H	3.63916900	-4.18334600	-4.90770000
C	5.84830400	-4.41273100	-1.73775800
H	5.36753200	-2.36097500	-1.39923100
C	5.67604600	-5.53383800	-2.55294100
H	4.72841800	-6.30921000	-4.32870600
H	6.45297500	-4.47653000	-0.83684600
H	6.15444600	-6.47567000	-2.29588000
S	3.11603900	-2.30986100	1.94766100
O	3.12752500	-1.98095000	3.37616900
O	4.35225700	-2.27851700	1.14792600
C	2.42148100	-3.96273500	1.81868600
C	1.33463100	-4.30597400	2.62958600
C	2.96497300	-4.88370700	0.92627500
C	0.78882300	-5.58157900	2.52891100
H	0.92880800	-3.58267700	3.32971100
C	2.40590500	-6.16105000	0.84128200
H	3.81523400	-4.60788100	0.31214400
C	1.31443900	-6.53009100	1.63536900
H	-0.05795700	-5.84908500	3.15698900
H	2.83030300	-6.88002600	0.14462000
C	0.71922800	-7.91623200	1.55159400
H	-0.36737100	-7.87682400	1.40925200
H	1.14763500	-8.48654100	0.72146600
H	0.90156800	-8.48318500	2.47367000
C	0.32863400	2.85602700	1.46980800
C	0.55865100	4.12300200	2.28037400
H	1.51374900	4.12308600	2.81324800
C	0.46081800	5.40997200	1.45890800
O	-0.34135100	5.62425300	0.57683900
O	1.37678400	6.31692200	1.87251800
C	-1.99773000	1.20002400	2.48805000
C	-1.11439500	0.08687500	2.23107700
C	-2.82897100	-0.54237300	0.91487500
S	-3.56240200	0.85365000	1.72564100
N	-1.59725700	-0.79837300	1.25267400
C	-3.58566500	-1.34376700	-0.05793200
C	-4.80923600	-0.90607100	-0.59268800
C	-3.07441400	-2.59091000	-0.46207000
C	-5.50394500	-1.69481300	-1.50720000
H	-5.21249600	0.06030100	-0.30263900
C	-3.77244200	-3.37566300	-1.37562600
H	-2.13339400	-2.92615300	-0.04033800

C	-4.98913600	-2.93192100	-1.90232200
H	-6.44728100	-1.34118900	-1.91436000
H	-3.36766300	-4.33825600	-1.67664800
H	-5.53232900	-3.54607600	-2.61552800
C	-1.97016500	1.95188200	3.79174400
H	-0.92688800	2.10924300	4.07980300
H	-2.46508900	2.92780600	3.71970100
H	-2.45583400	1.38993100	4.60041100
O	0.04315100	-0.00641600	2.70690900
H	-0.21438200	4.20293600	3.05584400
C	-0.96906800	2.49401100	1.10153800
H	-1.14981400	1.83578900	0.25740900
H	-1.73497800	3.25633500	1.21261700
C	1.34672400	7.59114800	1.18387500
H	0.35033200	8.02804700	1.31657500
H	1.49011900	7.41814100	0.11366100
C	2.42933700	8.46827300	1.75595000
C	2.48055600	8.73482100	3.13141100
C	3.38600200	9.05062200	0.91774700
C	3.46983200	9.56516700	3.65529000
H	1.74688900	8.27907000	3.79097100
C	4.37340900	9.89067800	1.43892900
H	3.35624400	8.84713500	-0.15024200
C	4.41805100	10.14809500	2.80920500
H	3.50064900	9.76116600	4.72380100
H	5.10796900	10.33867000	0.77495800
H	5.18683300	10.79838400	3.21786300

***(R)*-E**

C	0.73249700	-2.04032300	-1.46148000
H	0.70782900	-2.77889100	-2.26935300
H	0.27674100	-2.48194900	-0.56996400
H	0.12532100	-1.18347800	-1.77187100
C	2.17311800	-1.62804400	-1.16862300
C	2.34231300	-0.72970700	0.08958300
C	1.48638600	0.54447100	-0.03559300
H	0.43730800	0.29416800	0.13121300
H	1.58195500	0.88473300	-1.06960900
P	1.88892300	2.00732400	1.04135600
C	3.15668700	1.52974000	2.29017400
C	4.48784300	1.24543300	1.93825400
C	2.75951200	1.39064900	3.62657100
C	5.40056100	0.83453500	2.90898400
H	4.81798300	1.34780300	0.90862800
C	3.67837300	0.99061900	4.59702800
H	1.72160700	1.56832900	3.88675500
C	4.99769000	0.71211100	4.24056800
H	6.42292000	0.60375900	2.62332900
H	3.35699600	0.87851600	5.62857400
H	5.70868100	0.38634400	4.99482300
C	2.80908300	3.18819100	-0.07084200
C	3.89262600	3.96010800	0.37698200
C	2.27029600	3.47285300	-1.33882000
C	4.44887500	4.95277900	-0.43542300
H	4.31488000	3.78624000	1.36084800
C	2.82661900	4.45869800	-2.15119300

H	1.39331200	2.93850600	-1.69356800
C	3.92393100	5.20023200	-1.70361800
H	5.29869100	5.52638400	-0.07395400
H	2.39909600	4.65273000	-3.13097700
H	4.35902100	5.96864100	-2.33686100
O	2.74411100	-0.91201400	-2.26913800
Si	3.65897600	-1.48271000	-3.55825500
C	5.00513500	-0.14317000	-3.86404200
C	5.92765800	-0.56205400	-5.02913900
H	6.43702100	-1.51280000	-4.82877600
H	5.37746800	-0.66215500	-5.97179200
H	6.70459300	0.20009100	-5.18668600
C	5.86600000	0.07123400	-2.60145300
H	6.58388100	0.88701300	-2.77092400
H	5.25469300	0.34477900	-1.73393900
H	6.44486900	-0.82227200	-2.34357200
C	4.31070000	1.18955900	-4.22249100
H	3.66994200	1.54411300	-3.40760200
H	5.06405000	1.96848400	-4.41061700
H	3.69463200	1.10013600	-5.12466200
N	1.97677700	-1.47094500	1.29971800
H	1.28387000	-1.07588400	1.94391200
H	3.40261600	-0.46282600	0.12589600
H	2.76416200	-2.52980800	-0.97879900
C	2.52976600	-1.60276700	-5.07559100
C	2.96238800	-2.13039100	-6.30950100
C	1.21686800	-1.09895600	-5.01668500
C	2.12305900	-2.16305000	-7.42496000
H	3.96966400	-2.52774600	-6.40818000
C	0.37226500	-1.12710500	-6.12874800
H	0.85234400	-0.67594900	-4.08547300
C	0.82314600	-1.66109900	-7.33673900
H	2.48458300	-2.57919600	-8.36206000
H	-0.63755700	-0.73147300	-6.05136500
H	0.16789000	-1.68488100	-8.20378400
C	4.39387200	-3.17777700	-3.13328800
C	4.17506300	-4.31201200	-3.93900200
C	5.18582400	-3.35022100	-1.97780600
C	4.73951900	-5.55093000	-3.62644900
H	3.54701500	-4.23351200	-4.82126400
C	5.75004200	-4.58719900	-1.65861900
H	5.34868300	-2.52220800	-1.29470200
C	5.53370000	-5.69064000	-2.48743600
H	4.55465700	-6.40679200	-4.27105300
H	6.35345400	-4.68549200	-0.75997100
H	5.97638300	-6.65315500	-2.24321700
S	3.01562400	-2.54451000	2.03713400
O	2.99153700	-2.28351000	3.47863500
O	4.27265800	-2.53992200	1.27312600
C	2.23673800	-4.14732500	1.79879500
C	1.07817400	-4.45184900	2.52058800
C	2.78868300	-5.06851200	0.91148400
C	0.47106900	-5.69045900	2.33756500
H	0.66166700	-3.72737900	3.21338500
C	2.16701900	-6.30769800	0.74287000
H	3.69043200	-4.81932900	0.36294000

C	1.00543900	-6.63921900	1.44979000
H	-0.43139000	-5.92871000	2.89602100
H	2.59650200	-7.02585700	0.04854600
C	0.35179800	-7.99144800	1.28518200
H	0.60039900	-8.65552800	2.12367700
H	-0.74054000	-7.90813900	1.25432200
H	0.68080700	-8.48642200	0.36591900
C	0.47067900	2.69416100	1.71686000
C	0.67380700	4.02310800	2.42461900
H	1.66106700	4.10899200	2.89269200
C	0.46959300	5.26782900	1.55372900
O	-0.27752400	5.36114600	0.60172100
O	1.21264100	6.30430200	2.01184900
C	-1.77398600	1.41061600	2.31840300
C	-1.15072100	0.01812400	2.31052300
C	-2.95376600	-0.49077400	1.08511400
S	-3.44679600	1.13830500	1.58682400
N	-1.79244700	-0.91482400	1.49692100
C	-3.83470100	-1.32072200	0.25219200
C	-5.03476300	-0.82318600	-0.28376700
C	-3.46360000	-2.65227900	-0.01410000
C	-5.84627100	-1.63879600	-1.06840800
H	-5.32966500	0.20511200	-0.09539100
C	-4.27809700	-3.46271500	-0.79859900
H	-2.53662900	-3.02639900	0.40605500
C	-5.47066400	-2.95940200	-1.32752900
H	-6.77068500	-1.24349100	-1.47955800
H	-3.98394300	-4.48914200	-0.99897000
H	-6.10523300	-3.59419700	-1.94001800
C	-1.85727600	2.01018800	3.72103000
H	-0.84436600	2.07810200	4.12586600
H	-2.29893200	3.01214900	3.69713400
H	-2.45545900	1.38378100	4.38972800
O	-0.10058500	-0.21405200	2.90170600
H	-0.03535100	4.13635200	3.25903200
C	-0.92471600	2.33401400	1.30333800
H	-0.98321100	1.83287600	0.33103800
H	-1.51325400	3.24995800	1.18364700
C	1.06823900	7.54715600	1.27877900
H	0.02712500	7.87879200	1.34043600
H	1.29133500	7.34851700	0.22563600
C	2.01131600	8.55783300	1.87441100
C	1.52692200	9.64489700	2.60935700
C	3.39540300	8.42039900	1.69907600
C	2.40552300	10.58198000	3.15819900
H	0.45480800	9.75873400	2.75264000
C	4.27547100	9.35027600	2.25012700
H	3.77663100	7.57491500	1.13149600
C	3.78160900	10.43545100	2.98033300
H	2.01484600	11.42305900	3.72480900
H	5.34671000	9.23282100	2.10836000
H	4.46739100	11.16277200	3.40677800

Ts4-*si*

C	0.73445200	-3.60402300	0.18213500
H	0.86031400	-4.65870800	-0.08340000

H	0.28179400	-3.54701200	1.17651500
H	0.03474000	-3.15719300	-0.53203400
C	2.08531600	-2.89289400	0.15346600
C	2.06490100	-1.43045500	0.68629900
C	1.05232200	-0.58255400	-0.11308200
H	0.04299600	-0.86008100	0.20402200
H	1.17805400	-0.82721500	-1.16957600
P	1.19653100	1.25752600	0.05672000
C	2.62522200	1.71472400	1.09543000
C	3.93482700	1.66804600	0.58575400
C	2.41925700	2.03924400	2.44360600
C	5.01724000	1.96103700	1.41388100
H	4.11243300	1.41125200	-0.45421600
C	3.50611100	2.33956400	3.26431900
H	1.41639800	2.02834100	2.85629900
C	4.80276400	2.30340400	2.75108000
H	6.02662300	1.91764000	1.01482300
H	3.33232100	2.57828600	4.30860000
H	5.64846500	2.52787900	3.39516500
C	1.58879500	1.91122800	-1.62421600
C	2.29507200	3.11586400	-1.77798900
C	1.03061100	1.30849400	-2.76459800
C	2.46369800	3.68534400	-3.04174300
H	2.72143600	3.61258400	-0.91317700
C	1.20637600	1.87481600	-4.02620600
H	0.44157700	0.40085300	-2.67822800
C	1.92601200	3.06323800	-4.16917200
H	3.01289800	4.61771200	-3.13870300
H	0.77550000	1.38840900	-4.89662000
H	2.06085100	3.50422600	-5.15285600
O	2.60399500	-2.83136800	-1.18192900
Si	3.74506700	-3.78807000	-1.95975600
C	4.79418100	-2.55983600	-3.00655500
C	5.91526500	-3.30645000	-3.76110500
H	6.58685600	-3.83809000	-3.07580900
H	5.51472100	-4.03281100	-4.47728900
H	6.52616300	-2.59206300	-4.33188800
C	5.43715200	-1.48664900	-2.10302300
H	5.98564400	-0.75732800	-2.71730700
H	4.68414100	-0.93584500	-1.52749500
H	6.15263800	-1.92090600	-1.39629800
C	3.87434800	-1.86017900	-4.03155600
H	3.07451100	-1.29526200	-3.53902400
H	4.45486900	-1.14898600	-4.63757100
H	3.40756500	-2.57608700	-4.71762200
N	1.74071600	-1.30846400	2.10410800
H	0.73505800	-1.37485900	2.35259900
H	3.07672100	-1.04414100	0.54216400
H	2.78821600	-3.43987400	0.79009600
C	2.82958100	-4.98263400	-3.11042200
C	3.49331600	-5.95895000	-3.88141600
C	1.43360000	-4.88392100	-3.26054700
C	2.79701700	-6.79974900	-4.75198800
H	4.57170300	-6.07379900	-3.80394200
C	0.73085400	-5.72129600	-4.12993100
H	0.88998200	-4.13834100	-2.68842700

C	1.41133200	-6.68307000	-4.87791800
H	3.33634900	-7.54508700	-5.33129000
H	-0.34796800	-5.62243100	-4.22241800
H	0.86704400	-7.33677800	-5.55471100
C	4.78321100	-4.73365100	-0.68941800
C	4.94662500	-6.13153600	-0.74567400
C	5.42477800	-4.05071200	0.36565000
C	5.73140900	-6.81322700	0.18724900
H	4.44716600	-6.70317900	-1.52224700
C	6.20574200	-4.72798700	1.30465100
H	5.29809800	-2.97934000	0.48707600
C	6.36726200	-6.11221500	1.21301500
H	5.84050800	-7.89259600	0.11497900
H	6.67914000	-4.17039000	2.10840500
H	6.97810200	-6.64113400	1.94041500
S	2.81013200	-1.73617100	3.30536900
O	2.66014800	-0.78905400	4.41445000
O	4.12197400	-1.92217100	2.66380700
C	2.23406200	-3.32379100	3.91842700
C	1.01052700	-3.38404300	4.59642100
C	3.00177400	-4.46869600	3.71769500
C	0.55927000	-4.61439000	5.06283600
H	0.42092300	-2.48452100	4.74696000
C	2.53330900	-5.69296000	4.20051900
H	3.94851000	-4.40167100	3.19254200
C	1.31062700	-5.78686700	4.87467600
H	-0.39350600	-4.66718900	5.58456900
H	3.13075400	-6.58815200	4.04597500
C	0.80005500	-7.11277300	5.38831700
H	-0.15719100	-7.37728500	4.92188100
H	1.50745500	-7.92189100	5.18177800
H	0.63117100	-7.08230200	6.47184800
C	-0.30459100	2.00057300	0.61943600
C	-0.28084400	3.50881000	0.82538900
H	0.67892500	3.87306600	1.20144800
C	-0.65657400	4.31196900	-0.41888300
O	-1.54363000	4.03596800	-1.19595000
O	0.11718000	5.41918400	-0.53541200
C	-2.51392200	0.52633200	2.35001800
C	-1.41982600	-0.31216900	2.77841200
C	-1.17872600	1.33741900	4.28940100
S	-2.67588400	1.86853100	3.50619100
N	-0.67266900	0.23153700	3.82542900
C	-0.55452900	2.10613700	5.37671200
C	-1.13707300	3.27591300	5.89296200
C	0.66399700	1.65048900	5.91808600
C	-0.51978000	3.97677100	6.92666100
H	-2.07773100	3.63962500	5.48770600
C	1.27414900	2.35636500	6.95292500
H	1.11904400	0.75171000	5.51388200
C	0.68765600	3.51948100	7.46100000
H	-0.98281800	4.87914100	7.31668500
H	2.21129900	1.99281200	7.36628600
H	1.16706600	4.06591600	8.26898500
C	-3.73985200	-0.05095900	1.69549600
H	-3.44421200	-0.96496800	1.16991300

H	-4.51277200	-0.32081100	2.42654500
H	-4.19566700	0.63393500	0.96763400
O	-1.06391900	-1.37489400	2.20452100
H	-1.02409300	3.76508600	1.59193700
C	-1.52971400	1.33720500	0.55874800
H	-1.60419300	0.34715600	0.12194500
H	-2.41080700	1.95436800	0.41120000
C	-0.16105200	6.25631700	-1.67308000
H	-1.18065900	6.64866600	-1.59014900
H	-0.13089200	5.63228700	-2.57422700
C	0.85330700	7.37323900	-1.74236600
C	0.55890700	8.50543100	-2.51363800
C	2.09431100	7.29609300	-1.10022500
C	1.48902000	9.53576200	-2.64989300
H	-0.40643800	8.58205600	-3.01005300
C	3.02357400	8.33094100	-1.23094500
H	2.32214700	6.43285000	-0.48423700
C	2.72672700	9.45149900	-2.00776500
H	1.24386000	10.40761500	-3.25064000
H	3.98047100	8.26063200	-0.72013900
H	3.45041300	10.25591600	-2.10767900

***(S)*-E**

C	0.43220600	-3.47065500	0.74915000
H	0.54149100	-4.55148200	0.61404400
H	0.21990800	-3.27770700	1.80530300
H	-0.43221200	-3.14138900	0.16349700
C	1.70675600	-2.75489100	0.30621400
C	1.72792100	-1.22387900	0.58894500
C	0.54908100	-0.51752800	-0.11457900
H	-0.38848400	-0.76668500	0.39057300
H	0.51724900	-0.92694000	-1.12582200
P	0.63636500	1.33859400	-0.26361100
C	2.40528300	1.81019300	-0.15193300
C	3.25070300	1.80418600	-1.27340100
C	2.95511700	2.06120400	1.11626300
C	4.61349600	2.07191100	-1.13067700
H	2.84947700	1.59337500	-2.25872300
C	4.31738000	2.32743100	1.25322200
H	2.31934300	2.02013800	1.99347100
C	5.14750000	2.33903200	0.13104600
H	5.25548000	2.06773900	-2.00734500
H	4.72806500	2.50462000	2.24264800
H	6.20958800	2.54159200	0.24079600
C	0.18271000	1.62063600	-2.05950200
C	0.52767000	2.83348800	-2.68506300
C	-0.68965800	0.75364300	-2.74134000
C	0.05071700	3.14745400	-3.95789200
H	1.16810300	3.54539800	-2.17543000
C	-1.17255400	1.07015800	-4.01297400
H	-1.00980200	-0.17903100	-2.28706500
C	-0.79873300	2.26452400	-4.62919000
H	0.34143300	4.08715000	-4.41985500
H	-1.83879800	0.37781500	-4.52118900
H	-1.17001400	2.50836100	-5.62103700
O	1.91908200	-2.91511900	-1.10087300

Si	2.89675200	-3.98659600	-1.94682100
C	3.58907900	-2.96816900	-3.42202700
C	4.47494800	-3.85270900	-4.32635900
H	5.31979200	-4.28639700	-3.77663200
H	3.90761700	-4.67200500	-4.78185300
H	4.89225700	-3.25115500	-5.14683000
C	4.43141100	-1.77505000	-2.92266700
H	4.75121900	-1.16067200	-3.77747200
H	3.86663500	-1.12590600	-2.24427300
H	5.33851700	-2.10271200	-2.40278300
C	2.40442800	-2.42516800	-4.25361500
H	1.76689300	-1.75621700	-3.66434600
H	2.78057300	-1.85132300	-5.11324900
H	1.77473100	-3.23224800	-4.64584200
N	1.71950700	-0.88950100	2.01398400
H	0.81375400	-0.93195400	2.48590400
H	2.66924100	-0.85343500	0.17778700
H	2.55709900	-3.17944700	0.85091300
C	1.81019100	-5.39319700	-2.60448300
C	2.33404900	-6.50796600	-3.29066900
C	0.41135200	-5.32286800	-2.46525700
C	1.50385300	-7.50797200	-3.80107400
H	3.40766200	-6.60553300	-3.43222000
C	-0.42519200	-6.31900200	-2.97409300
H	-0.02774700	-4.47196500	-1.95347500
C	0.11965000	-7.41649300	-3.64210500
H	1.93783100	-8.35720300	-4.32308600
H	-1.50221100	-6.23723400	-2.84919700
H	-0.52869400	-8.19385000	-4.03838400
C	4.25419700	-4.65715300	-0.80749300
C	4.46253800	-6.03662600	-0.61498800
C	5.09669400	-3.77258900	-0.10123900
C	5.47820300	-6.51330000	0.21711600
H	3.81766000	-6.75440600	-1.11310000
C	6.11047200	-4.24298500	0.73615300
H	4.95335300	-2.69911100	-0.17743600
C	6.30900500	-5.61698200	0.89128400
H	5.61643100	-7.58459900	0.34136800
H	6.73745800	-3.53318600	1.26924300
H	7.10127400	-5.98542500	1.53831200
S	3.02706700	-1.10963500	3.02093100
O	3.00011000	-0.02867100	4.01429300
O	4.20280700	-1.30436300	2.16211800
C	2.71410500	-2.63119600	3.92549700
C	1.67234800	-2.66744000	4.86075500
C	3.50718900	-3.75196700	3.68912200
C	1.42913300	-3.85038200	5.55163300
H	1.05932100	-1.78801100	5.03826500
C	3.24899700	-4.92759400	4.39781700
H	4.31159500	-3.70425900	2.96306200
C	2.21187200	-4.99723600	5.33471000
H	0.61816000	-3.88538600	6.27551800
H	3.86648600	-5.80334200	4.21424400
C	1.93439700	-6.26865400	6.10201500
H	2.07877500	-6.12194900	7.17984500
H	0.89913900	-6.60201100	5.95962400

H	2.59534500	-7.08082000	5.78419800
C	-0.29183000	2.15439600	0.93901500
C	0.00158100	3.64777800	1.15163100
H	1.04153100	3.88486000	0.91687400
C	-0.89638300	4.57156900	0.34572200
O	-2.08656800	4.74179100	0.53398600
O	-0.22188700	5.19444700	-0.65852500
C	-2.14291600	1.31440800	2.66971700
C	-1.20756700	0.22745100	3.21890000
C	-0.72771400	1.77401200	4.75376900
S	-1.93949200	2.70674000	3.86738200
N	-0.46412400	0.56639700	4.33619900
C	-0.03008400	2.36765200	5.90118400
C	-0.58378700	3.43983700	6.62188600
C	1.22549600	1.85211600	6.27397400
C	0.10026500	3.98169900	7.70664200
H	-1.55777700	3.83483100	6.34658500
C	1.90784600	2.40888100	7.35295400
H	1.66471800	1.04910600	5.69118900
C	1.34728800	3.46741200	8.07326500
H	-0.33851500	4.80282100	8.26626000
H	2.88290200	2.01738600	7.62855900
H	1.88248800	3.89469200	8.91716400
C	-3.59719500	0.81166100	2.67006400
H	-3.65037900	-0.10971300	2.08048400
H	-3.95119300	0.59537200	3.68266800
H	-4.26184300	1.55596600	2.21998600
O	-1.11667700	-0.86381400	2.66294600
H	-0.16310500	3.91437500	2.20215200
C	-1.71568900	1.69086200	1.20005400
H	-1.94423300	0.77828300	0.63500700
H	-2.44607800	2.43867400	0.86298000
C	-1.00969000	6.05731900	-1.50435600
H	-1.58814200	6.73338800	-0.86401100
H	-1.72698400	5.45195800	-2.06784600
C	-0.08741300	6.81454500	-2.42706200
C	1.12769900	7.33977300	-1.96701400
C	-0.46344200	7.04470100	-3.75606900
C	1.94741900	8.07858200	-2.82020600
H	1.43299100	7.15506500	-0.94151000
C	0.35206100	7.79215500	-4.60901500
H	-1.40209500	6.63734700	-4.12539900
C	1.56132400	8.31007400	-4.14297300
H	2.88972300	8.47531400	-2.45133000
H	0.04484500	7.96274400	-5.63752200
H	2.20055800	8.88708400	-4.80586800

***(R)*-7a**

C	0.42712300	3.76230800	2.12801200
C	0.80894400	5.04382300	2.05638100
C	2.01930800	5.52187700	2.76641400
O	2.76491900	4.84208100	3.44466700
O	2.19570800	6.85033300	2.55947500
C	-1.81089300	2.49951100	2.31760200
C	-1.22825500	1.16482600	2.87374000
C	-2.87960800	0.25840500	1.64804000

S	-3.24708300	1.96726400	1.28615600
N	-1.87454600	0.00724900	2.42488700
C	-3.70929100	-0.81124400	1.07441600
C	-4.79676300	-0.53689800	0.22871400
C	-3.39986000	-2.14792000	1.38766200
C	-5.56072900	-1.57781100	-0.29329200
H	-5.04830500	0.48936600	-0.02362200
C	-4.16562200	-3.18403300	0.86300200
H	-2.55808700	-2.34519200	2.04203600
C	-5.24698000	-2.90220500	0.02240700
H	-6.39995800	-1.35614100	-0.94609700
H	-3.92073300	-4.21328000	1.10918500
H	-5.84405900	-3.71306400	-0.38588900
C	-2.26542600	3.39602800	3.47827300
H	-1.41439700	3.57765400	4.14153500
H	-2.63332600	4.35960700	3.11027700
H	-3.06144300	2.91761800	4.05617200
O	-0.28447500	1.16062100	3.63654200
H	0.25886900	5.78091900	1.47681800
C	-0.76739900	3.21059500	1.40063800
H	-0.43009900	2.48654900	0.64564900
H	-1.27408300	4.01831100	0.85994900
H	1.01354200	3.07475800	2.73250700
C	3.35504300	7.42883000	3.21099900
H	3.28830400	7.22498100	4.28401000
H	4.25223000	6.92820600	2.83390800
C	3.36934000	8.90469200	2.91782800
C	2.66959100	9.79835100	3.73853700
C	4.06821400	9.40583500	1.81293700
C	2.66617200	11.16541000	3.46056500
H	2.12579400	9.41848900	4.60043900
C	4.06819000	10.77273000	1.53161300
H	4.61624500	8.71991700	1.17104000
C	3.36644100	11.65516300	2.35558400
H	2.12155800	11.84819700	4.10727200
H	4.61755400	11.14868900	0.67262000
H	3.36836400	12.72036300	2.14010200

***(S)*-7a**

C	0.42712300	3.76230800	-2.12801200
C	0.80894400	5.04382300	-2.05638100
C	2.01930800	5.52187700	-2.76641400
O	2.76491900	4.84208100	-3.44466700
O	2.19570800	6.85033300	-2.55947500
C	-1.81089300	2.49951100	-2.31760200
C	-1.22825500	1.16482600	-2.87374000
C	-2.87960800	0.25840500	-1.64804000
S	-3.24708300	1.96726400	-1.28615600
N	-1.87454600	0.00724900	-2.42488700
C	-3.70929100	-0.81124400	-1.07441600
C	-4.79676300	-0.53689800	-0.22871400
C	-3.39986000	-2.14792000	-1.38766200
C	-5.56072900	-1.57781100	0.29329200
H	-5.04830500	0.48936600	0.02362200
C	-4.16562200	-3.18403300	-0.86300200
H	-2.55808700	-2.34519200	-2.04203600

C	-5.24698000	-2.90220500	-0.02240700
H	-6.39995800	-1.35614100	0.94609700
H	-3.92073300	-4.21328000	-1.10918500
H	-5.84405900	-3.71306400	0.38588900
C	-2.26542600	3.39602800	-3.47827300
H	-1.41439700	3.57765400	-4.14153500
H	-2.63332600	4.35960700	-3.11027700
H	-3.06144300	2.91761800	-4.05617200
O	-0.28447500	1.16062100	-3.63654200
H	0.25886900	5.78091900	-1.47681800
C	-0.76739900	3.21059500	-1.40063800
H	-0.43009900	2.48654900	-0.64564900
H	-1.27408300	4.01831100	-0.85994900
H	1.01354200	3.07475800	-2.73250700
C	3.35504300	7.42883000	-3.21099900
H	3.28830400	7.22498100	-4.28401000
H	4.25223000	6.92820600	-2.83390800
C	3.36934000	8.90469200	-2.91782800
C	2.66959100	9.79835100	-3.73853700
C	4.06821400	9.40583500	-1.81293700
C	2.66617200	11.16541000	-3.46056500
H	2.12579400	9.41848900	-4.60043900
C	4.06819000	10.77273000	-1.53161300
H	4.61624500	8.71991700	-1.17104000
C	3.36644100	11.65516300	-2.35558400
H	2.12155800	11.84819700	-4.10727200
H	4.61755400	11.14868900	-0.67262000
H	3.36836400	12.72036300	-2.14010200

8a

C	0.15695200	1.01428700	0.54656500
C	1.67639900	1.18181800	0.35149900
C	0.77181000	3.13600900	0.17901200
N	1.92917100	2.54792600	0.13830400
C	0.54585800	4.57133400	0.00008300
C	-0.74861300	5.11163100	0.05642500
C	1.64822000	5.41054400	-0.23158000
C	-0.93551600	6.48104100	-0.11799000
H	-1.59451200	4.45693500	0.23560000
C	1.45317200	6.77745500	-0.40448600
H	2.63938700	4.97159100	-0.27157400
C	0.16280300	7.31399100	-0.34815600
H	-1.93703100	6.89929000	-0.07433400
H	2.30562300	7.42638100	-0.58341900
H	0.01356200	8.38182700	-0.48360500
C	-0.49314000	0.08517300	-0.46605200
H	-0.03795900	-0.90562300	-0.37107200
H	-1.57030500	0.00265700	-0.29336700
H	-0.32512500	0.44653900	-1.48560600
H	-0.06025500	0.69943800	1.57319200
O	2.48616900	0.28187600	0.36936400
O	-0.32624400	2.37197400	0.40301400

F

C	-1.46940000	-0.93091600	0.07036400
H	-2.15952900	-1.59708900	-0.45677500

H	-2.01396400	-0.44491500	0.88602000
H	-1.14248600	-0.15813400	-0.63339400
C	-0.28434400	-1.72084200	0.62254700
C	0.65001600	-0.89363400	1.55103800
C	1.16120700	0.36508200	0.82392500
H	0.38046500	1.13017800	0.81073900
H	1.37143700	0.09388900	-0.21428500
P	2.68628100	1.22604400	1.46009100
C	3.26123400	0.49927600	3.02449600
C	2.59286200	0.81193600	4.22127800
C	4.36548100	-0.36899900	3.04579500
C	3.02696400	0.24749100	5.41999300
H	1.74366900	1.48595100	4.22080400
C	4.79224400	-0.92381300	4.25156000
H	4.89659800	-0.60501200	2.12991600
C	4.12253800	-0.61687000	5.43739700
H	2.49030400	0.47262300	6.33546200
H	5.64697600	-1.59419600	4.26189100
H	4.45254800	-1.05461000	6.37556700
C	3.99959300	0.97567400	0.21954000
C	4.10895000	-0.22513200	-0.49810700
C	4.93163000	2.00364000	0.00664500
C	5.14416200	-0.39626700	-1.41896000
H	3.39034700	-1.02574300	-0.34871900
C	5.96231200	1.82460700	-0.91553700
H	4.84230900	2.93356600	0.56107200
C	6.07038000	0.62735600	-1.62742000
H	5.21952100	-1.32675300	-1.97381400
H	6.67830100	2.62459000	-1.08086400
H	6.87388600	0.49338700	-2.34656200
O	0.52920400	-2.26447000	-0.42907400
Si	0.33627200	-3.75407400	-1.19620900
C	2.10909800	-4.31529600	-1.68977600
C	2.05820700	-5.71158200	-2.34814900
H	1.64440700	-6.46908400	-1.67170500
H	1.45922600	-5.71011100	-3.26595000
H	3.07240400	-6.03512000	-2.62341400
C	3.03681300	-4.38391000	-0.45857000
H	4.06240900	-4.62810100	-0.77189200
H	3.07337300	-3.43292900	0.08504400
H	2.72153600	-5.16082700	0.24609900
C	2.69092100	-3.30746000	-2.70599200
H	2.74442200	-2.29316500	-2.29425100
H	3.71011000	-3.60741900	-2.99218600
H	2.09241900	-3.25986800	-3.62235100
N	-0.04254700	-0.50089300	2.77738100
H	-0.26194400	0.48449400	2.97069700
H	1.48746800	-1.54988600	1.81077400
H	-0.66511100	-2.54238100	1.23733300
C	-0.68541800	-3.50647800	-2.77230200
C	-1.15014500	-4.58188700	-3.55719300
C	-0.94012900	-2.20640500	-3.24859400
C	-1.84931400	-4.36952000	-4.74714400
H	-0.96755900	-5.60594400	-3.24117200
C	-1.63712800	-1.98666100	-4.43862500
H	-0.58262000	-1.35310800	-2.68050200

C	-2.09735400	-3.06895300	-5.19004400
H	-2.19856500	-5.21943700	-5.32811700
H	-1.81863000	-0.97030000	-4.77939100
H	-2.64128200	-2.90123600	-6.11606800
C	-0.50407900	-4.96927000	-0.00972800
C	-1.69150100	-5.65202500	-0.33721800
C	0.05479600	-5.22099900	1.26164100
C	-2.27662200	-6.56442900	0.54413500
H	-2.17733200	-5.46602800	-1.29026000
C	-0.52700700	-6.12947900	2.14810800
H	0.94041100	-4.68387200	1.58757400
C	-1.69155500	-6.81138400	1.78703200
H	-3.19089200	-7.07939600	0.25953100
H	-0.07317300	-6.29512400	3.12146100
H	-2.14299100	-7.52363200	2.47312400
S	-0.32448500	-1.53342500	4.04706800
O	-0.10119500	-0.78684900	5.28840700
O	0.39758500	-2.77989000	3.75366200
C	-2.08657400	-1.88639700	3.99048800
C	-2.99013100	-0.89442500	4.38650700
C	-2.53704200	-3.13233700	3.55986800
C	-4.35471800	-1.16033000	4.33395500
H	-2.62542300	0.06770900	4.73240200
C	-3.91050400	-3.38181200	3.51696800
H	-1.82501400	-3.89654100	3.26865200
C	-4.83797000	-2.40653000	3.90066300
H	-5.05935500	-0.38961200	4.63800600
H	-4.26082800	-4.35368600	3.17805800
C	-6.32245200	-2.68515400	3.87202100
H	-6.87539300	-1.86214300	3.40441500
H	-6.54752300	-3.60192300	3.31817800
H	-6.72237700	-2.80510900	4.88741500
C	2.35978200	3.00856800	1.59749700
C	1.54595200	3.50498300	0.60981900
C	3.07252300	3.67464800	2.63590100
H	1.39942300	4.57510200	0.53226800
H	1.08662300	2.87679800	-0.14484800
H	3.73169000	3.12321200	3.29165200
C	2.88973200	5.05049700	2.90837300
O	2.09575900	5.84078200	2.37266300
O	3.72826900	5.48772800	3.93707300
C	-0.85269100	4.63248500	3.05092300
C	-0.98177600	3.13149500	2.81661600
C	-2.19267700	4.10502400	1.33295300
N	-1.81455900	2.91438700	1.72381600
C	-3.08839400	4.37229600	0.20605100
C	-3.40172400	5.69039300	-0.16234200
C	-3.63512400	3.29397600	-0.50848400
C	-4.25563300	5.92370800	-1.23772200
H	-2.97124800	6.51663200	0.39283200
C	-4.48855500	3.53533000	-1.58103200
H	-3.38062200	2.28338700	-0.20753800
C	-4.79946900	4.84896400	-1.94659800
H	-4.49644600	6.94330400	-1.52439600
H	-4.91244400	2.70134400	-2.13311800
H	-5.46548200	5.03487700	-2.78496100

C	-1.28869500	5.10588000	4.42765200
H	-2.32691000	4.82579300	4.63601700
H	-0.63926200	4.64025800	5.17432600
H	-1.18200200	6.19019400	4.51291700
O	-0.45135200	2.25852800	3.49449800
C	3.47962800	6.80607400	4.41481000
H	3.18953800	7.45701100	3.58548800
H	4.44260900	7.14794600	4.81378800
C	2.42303900	6.84972800	5.50408400
C	2.25128000	5.77233300	6.38245000
C	1.63201800	7.99188900	5.67628400
C	1.31073700	5.83799800	7.41232500
H	2.85370900	4.87979200	6.24308500
C	0.69465400	8.06324500	6.70917500
H	1.74706300	8.82998400	4.99193200
C	0.53077200	6.98496100	7.58163900
H	1.18897000	4.99322700	8.08611100
H	0.08817000	8.95791400	6.82781900
H	-0.19986800	7.03685400	8.38484200
H	0.15984000	4.97850700	2.80516100
O	-1.73472000	5.16226200	2.01257600

Ts3

C	-1.84346600	-0.97009600	-0.00068900
H	-2.61114800	-1.65457300	-0.37494400
H	-2.29819900	-0.31183500	0.74636100
H	-1.51066500	-0.34920700	-0.83956400
C	-0.67715100	-1.75969800	0.59135900
C	0.34898700	-0.89305900	1.38005000
C	0.92826700	0.19734100	0.46549200
H	0.15670900	0.93788000	0.22972200
H	1.23433100	-0.27883600	-0.46906900
P	2.35191500	1.22718200	1.07360700
C	3.30455000	0.46134900	2.42653300
C	2.73554400	0.29406700	3.70214000
C	4.63165700	0.05201200	2.20486000
C	3.47978300	-0.28616300	4.72804900
H	1.71474000	0.59270900	3.89910900
C	5.36775400	-0.52552300	3.23902800
H	5.09404200	0.18328400	1.23369000
C	4.79373300	-0.69727200	4.49963800
H	3.01698100	-0.41965400	5.70067600
H	6.39143700	-0.83917100	3.05561900
H	5.36965200	-1.15009200	5.30199000
C	3.49182000	1.39079600	-0.34793500
C	3.79822400	0.27559700	-1.14640300
C	4.11487900	2.62184500	-0.60979000
C	4.70703700	0.39390900	-2.19773300
H	3.34186200	-0.69079700	-0.95181400
C	5.02534600	2.73047000	-1.66202100
H	3.87456300	3.49405500	-0.01035700
C	5.32033700	1.62168200	-2.45707600
H	4.93046100	-0.47248000	-2.81340600
H	5.49816500	3.68762500	-1.86153000
H	6.02483700	1.71339400	-3.27902100
O	0.06001700	-2.43198900	-0.43792900

Si	0.05196100	-4.04619300	-0.90940900
C	1.87574300	-4.41061400	-1.40839900
C	2.03290400	-5.88161200	-1.85054600
H	1.75484700	-6.58047900	-1.05232400
H	1.42411000	-6.11170300	-2.73211000
H	3.08002300	-6.08643200	-2.11682100
C	2.83636300	-4.14401400	-0.23053000
H	3.87865100	-4.28106800	-0.55441300
H	2.74056700	-3.12188100	0.15522900
H	2.66405200	-4.83408600	0.60244400
C	2.26216900	-3.48867800	-2.58647200
H	2.17496700	-2.42879900	-2.31786100
H	3.30394400	-3.67430000	-2.88758200
H	1.62845600	-3.65869200	-3.46398600
N	-0.18531900	-0.27199100	2.58973100
H	-0.75897500	0.59803400	2.50666400
H	1.15058400	-1.57075100	1.68788200
H	-1.06518900	-2.49827400	1.30150800
C	-1.05581000	-4.22233900	-2.43456500
C	-1.33943300	-5.46814000	-3.03114100
C	-1.58463200	-3.07558900	-3.05642700
C	-2.12139100	-5.56461000	-4.18384900
H	-0.94973700	-6.38364000	-2.59286300
C	-2.36753400	-3.16494100	-4.20963100
H	-1.37707700	-2.09795700	-2.63258700
C	-2.63979900	-4.41109800	-4.77570300
H	-2.32551600	-6.53975800	-4.61892600
H	-2.76432900	-2.26113500	-4.66532300
H	-3.24980100	-4.48374300	-5.67243100
C	-0.55733700	-5.14296600	0.50797900
C	-1.63429400	-6.03777100	0.35132300
C	0.06315400	-5.09606700	1.77412600
C	-2.05341700	-6.86606700	1.39470800
H	-2.16337300	-6.08662500	-0.59564200
C	-0.35327800	-5.91992900	2.82164900
H	0.86645600	-4.39217800	1.96828400
C	-1.40881300	-6.81442400	2.63161800
H	-2.88568700	-7.54847100	1.24098000
H	0.14475900	-5.85414700	3.78529700
H	-1.73095300	-7.46080800	3.44424100
S	-0.53494700	-1.17291000	3.94785800
O	-0.38138400	-0.25609200	5.08631100
O	0.27209300	-2.40089900	3.87178000
C	-2.27034900	-1.63218100	3.86814100
C	-3.24419800	-0.63330900	3.74690000
C	-2.62372300	-2.97693400	3.96206000
C	-4.58473200	-1.00541000	3.71694200
H	-2.95902900	0.41000100	3.64240500
C	-3.97539400	-3.32599000	3.93339300
H	-1.85352500	-3.73518600	4.04450800
C	-4.97335100	-2.35209200	3.81451600
H	-5.34461800	-0.23469300	3.61147300
H	-4.25339000	-4.37495400	3.99882900
C	-6.43582600	-2.72997900	3.79639300
H	-6.95918000	-2.26075800	2.95497900
H	-6.56930100	-3.81326900	3.71611500

H	-6.94085800	-2.39935000	4.71316100
C	1.74052000	2.89646800	1.46816700
C	1.11343300	3.51276900	0.43951600
C	1.89237500	3.47718000	2.80997100
H	0.71879400	4.51441000	0.55809500
H	1.01196400	3.06813800	-0.54677900
H	2.33619600	2.83784300	3.56843000
C	2.42795700	4.85029000	2.87895200
O	2.52237400	5.65378100	1.96390400
O	2.77490100	5.14788900	4.17027600
H	0.58553900	3.60502100	3.21587300
C	-0.79457700	3.75188800	3.62143800
C	-1.57904400	3.16641100	2.54617500
C	-1.73877400	5.31052400	2.36521100
N	-2.08219000	4.20931400	1.75385900
C	-2.03818200	6.67260300	1.92195400
C	-1.55367600	7.79202100	2.61809600
C	-2.83218100	6.85969700	0.77754600
C	-1.86235600	9.07652500	2.17474500
H	-0.93571400	7.65107300	3.49841600
C	-3.13832500	8.14580300	0.34226900
H	-3.19655700	5.98429400	0.25019300
C	-2.65474000	9.25810800	1.03842200
H	-1.48164400	9.93789200	2.71692800
H	-3.75436200	8.28305400	-0.54250800
H	-2.89339100	10.26156800	0.69577900
C	-0.79828900	3.25738500	5.04409100
H	-1.73932200	3.49866700	5.55723200
H	-0.67839800	2.16987200	5.04954500
H	0.02638900	3.69443500	5.61922900
O	-1.76159500	1.94894500	2.29053100
C	3.22259100	6.48962400	4.43506900
H	2.95844600	7.11377500	3.57583400
H	4.31595500	6.47552400	4.51830600
C	2.59977300	6.99931800	5.71256800
C	1.22178200	6.87116200	5.93945700
C	3.38600300	7.64689300	6.67116000
C	0.64783300	7.38389900	7.10265800
H	0.60225100	6.35831300	5.20921100
C	2.81079200	8.16903400	7.83233000
H	4.45783600	7.74214500	6.50987500
C	1.43938100	8.03742400	8.05165900
H	-0.42063200	7.27270100	7.26861600
H	3.43640100	8.66881300	8.56750500
H	0.98967200	8.43630900	8.95719000
O	-1.02712600	5.15751900	3.50078400

G

C	-0.72000600	0.33058900	0.83636200
H	-1.37064600	0.06072900	-0.00058100
H	-1.34040700	0.66499900	1.67493100
H	-0.09425800	1.17016800	0.51492000
C	0.14347900	-0.86375200	1.23321500
C	0.92697400	-0.67453200	2.56284300
C	1.70223500	0.65358700	2.53136700
H	1.01329600	1.46283600	2.80823900

H	2.11045100	0.85547900	1.53737300
P	3.11416200	0.88090000	3.67125000
C	2.82850700	0.38998500	5.39898300
C	2.69187100	-0.97971700	5.68352500
C	2.75126400	1.32811600	6.43705800
C	2.46582600	-1.39807900	6.99174900
H	2.72693200	-1.72219300	4.89308300
C	2.54265300	0.89743400	7.74732100
H	2.84909300	2.38623200	6.22872100
C	2.39609500	-0.46150900	8.02498200
H	2.32373700	-2.45439800	7.19479200
H	2.48128600	1.62962800	8.54717500
H	2.21621200	-0.79135800	9.04425300
C	4.54961000	-0.10006800	3.12195000
C	5.62277900	-0.29613500	4.00970200
C	4.61000600	-0.64855200	1.83188600
C	6.74539200	-1.01621100	3.60470400
H	5.57420400	0.09754600	5.02079500
C	5.73313600	-1.37844000	1.43787800
H	3.78980500	-0.52047900	1.13539000
C	6.80061200	-1.55894900	2.31839500
H	7.56935700	-1.16294300	4.29704300
H	5.76425600	-1.80822500	0.44137000
H	7.67151800	-2.12902500	2.00691900
O	1.12385700	-1.15207800	0.22430700
Si	1.01175700	-2.13708400	-1.13199600
C	1.68138800	-3.89232400	-0.69476100
C	1.55283600	-4.84360700	-1.90489100
H	0.51481300	-4.94011900	-2.24437300
H	2.15912900	-4.51167500	-2.75541500
H	1.90571100	-5.84873000	-1.63122100
C	0.91598800	-4.51040000	0.49765100
H	1.36650600	-5.47829900	0.76250700
H	0.94822600	-3.88607000	1.39776000
H	-0.13385000	-4.70647300	0.25364400
C	3.17201700	-3.77771600	-0.30592100
H	3.31111100	-3.14026100	0.57465200
H	3.57472100	-4.77084700	-0.05859300
H	3.77776200	-3.36780800	-1.12298500
N	0.05703300	-0.65373100	3.73280400
H	-0.15989400	0.28151900	4.16932500
H	1.60474000	-1.53183400	2.63586500
H	-0.49887600	-1.73888500	1.38492100
C	2.15203100	-1.28235100	-2.38008300
C	2.37744600	-1.78848400	-3.67625900
C	2.82712800	-0.09902000	-2.02499000
C	3.24025500	-1.15081400	-4.57026200
H	1.86370400	-2.68797500	-4.00654700
C	3.69372700	0.54181700	-2.91327900
H	2.66451700	0.33526300	-1.04362500
C	3.90517100	0.01639300	-4.18876400
H	3.39074000	-1.56402500	-5.56459000
H	4.19640700	1.45548700	-2.60535300
H	4.57712000	0.51433400	-4.88344400
C	-0.75997200	-2.19736600	-1.80504200
C	-1.06921700	-1.63808000	-3.06048200

C	-1.82405900	-2.77101900	-1.07747500
C	-2.36905300	-1.66015500	-3.57096500
H	-0.28483400	-1.17164800	-3.64947900
C	-3.12624200	-2.78904700	-1.57916300
H	-1.65084500	-3.20030600	-0.09491700
C	-3.40197400	-2.23713600	-2.83164900
H	-2.57436100	-1.22082200	-4.54404300
H	-3.92349300	-3.23096600	-0.98717500
H	-4.41517800	-2.25184900	-3.22528600
S	-0.48858400	-2.02571200	4.45356900
O	-0.75491300	-1.70644100	5.85887600
O	0.44205500	-3.11764200	4.09821900
C	-2.06884000	-2.42989000	3.69308500
C	-3.05853900	-1.44682000	3.58410700
C	-2.31316900	-3.74116000	3.28603000
C	-4.30040400	-1.79526700	3.05948600
H	-2.85810100	-0.41873400	3.87568600
C	-3.56567400	-4.07058500	2.76185100
H	-1.53108200	-4.48699600	3.37972400
C	-4.57704700	-3.10904400	2.64629200
H	-5.06672000	-1.02906900	2.96846800
H	-3.75881900	-5.09303000	2.44496800
C	-5.94147100	-3.47156100	2.10711700
H	-6.69252000	-3.48136000	2.90774800
H	-6.28272500	-2.74649500	1.35890300
H	-5.94068200	-4.46369600	1.64397400
C	3.50855100	2.66812100	3.58048000
C	2.53639000	3.52714800	3.91982200
C	4.85606600	3.11849700	3.06314600
H	2.71756300	4.59995300	3.85507800
H	1.54289700	3.19532000	4.24439400
H	5.01483200	4.17392400	3.30965400
C	4.98967000	2.95497000	1.55103200
O	4.21936800	2.33678000	0.84308100
O	6.08946800	3.57973400	1.10570000
H	5.68804400	2.56988900	3.52278200
C	-1.72143300	3.55263100	5.31092600
C	-1.25850700	2.50532100	4.53104800
C	-3.33763900	2.85777700	4.02734200
N	-2.33078500	2.08119800	3.72173000
C	-4.68334000	2.85000600	3.46577700
C	-5.69543000	3.67895500	3.98200400
C	-4.98550600	2.00410800	2.38217400
C	-6.97519100	3.65557800	3.43142100
H	-5.46852300	4.33538800	4.81569900
C	-6.26743300	1.98523700	1.83833800
H	-4.19893000	1.37779200	1.97417600
C	-7.27030500	2.80916200	2.35939500
H	-7.74716600	4.30122400	3.84282100
H	-6.48372700	1.33093000	0.99712700
H	-8.26950000	2.79400900	1.93207600
C	-1.10125500	4.38483600	6.36916500
H	-1.66020000	4.34554100	7.31541600
H	-0.09532400	3.99603900	6.55876900
H	-1.01028700	5.44686600	6.09259600
O	-0.07959500	1.96045200	4.49136100

C	6.34581100	3.47520000	-0.32796700
H	6.44065600	2.41503800	-0.57923600
H	5.47546500	3.87343000	-0.85709400
C	7.59813800	4.24661200	-0.63612900
C	7.53499400	5.61708100	-0.91850400
C	8.84549900	3.61034900	-0.63053100
C	8.69723700	6.33973700	-1.18801700
H	6.56994400	6.11843700	-0.92867200
C	10.01003400	4.32992200	-0.90067600
H	8.90311400	2.54572500	-0.41568400
C	9.93711300	5.69643200	-1.17931000
H	8.63547500	7.40177900	-1.40882800
H	10.97193800	3.82462600	-0.89717700
H	10.84276600	6.25739500	-1.39335900
O	-3.05065400	3.77655400	4.98127100

Ts5-*re*

C	0.74829800	-2.15390800	-1.56395700
H	0.78256200	-2.92537700	-2.34035700
H	0.32200500	-2.58499900	-0.65342300
H	0.07907300	-1.35809800	-1.90776000
C	2.15288500	-1.62588600	-1.28731900
C	2.25190800	-0.65984500	-0.07302000
C	1.27561900	0.52508300	-0.25053800
H	0.26219100	0.20202300	-0.00174000
H	1.29630900	0.79362700	-1.30791500
P	1.64887400	2.06000500	0.72059000
C	2.99943300	1.76996200	1.91272100
C	4.34602200	1.89020400	1.52628900
C	2.68417900	1.35224400	3.21576300
C	5.36140000	1.62091200	2.44289600
H	4.60587300	2.19589100	0.51811100
C	3.70803100	1.08979400	4.12530700
H	1.64831800	1.18068300	3.48979600
C	5.04285700	1.22933900	3.74471100
H	6.39954700	1.71106700	2.13623300
H	3.45804600	0.74699400	5.12430600
H	5.83598600	1.01367500	4.45526000
C	2.30269800	3.31234000	-0.47138400
C	3.05533700	4.40009900	0.00654700
C	1.91744400	3.30820800	-1.82247800
C	3.43595700	5.43461400	-0.84838800
H	3.34828200	4.44687700	1.04932600
C	2.30232900	4.34201400	-2.67690700
H	1.30881300	2.50716600	-2.22723600
C	3.06635700	5.40523400	-2.19411700
H	4.01553300	6.26524800	-0.45647800
H	2.00338800	4.31136200	-3.72078000
H	3.36727200	6.20797000	-2.86172400
O	2.68090100	-0.92247200	-2.42057400
Si	3.62849300	-1.50395700	-3.68197300
C	4.93581500	-0.13347000	-4.02430900
C	5.89237200	-0.56956700	-5.15517900
H	6.42437300	-1.49652900	-4.90877600
H	5.36398700	-0.72146500	-6.10335500
H	6.65020100	0.20845400	-5.32811300

C	5.76731300	0.16075700	-2.75771300
H	6.46028600	0.99324700	-2.94925900
H	5.13300200	0.44538200	-1.91045500
H	6.37130000	-0.70125300	-2.45458300
C	4.20867800	1.16072800	-4.45029600
H	3.54158500	1.52573300	-3.66153900
H	4.94104900	1.95535300	-4.65580700
H	3.61309600	1.01624900	-5.35912000
N	1.95742000	-1.34704500	1.18123800
H	1.19387100	-0.98881800	1.79234700
H	3.28497000	-0.29440800	-0.06626000
H	2.80693400	-2.47108200	-1.05268900
C	2.51309700	-1.71147300	-5.19993200
C	2.97414700	-2.24739200	-6.41992400
C	1.17564900	-1.27526400	-5.15422800
C	2.13935100	-2.35154300	-7.53436400
H	3.99991900	-2.59684800	-6.50789700
C	0.33522000	-1.37528500	-6.26530400
H	0.78760100	-0.85088200	-4.23316600
C	0.81507600	-1.91545700	-7.45932000
H	2.52313000	-2.77289300	-8.46022900
H	-0.69431400	-1.03220000	-6.19750800
H	0.16305600	-1.99587700	-8.32539500
C	4.42926700	-3.15266100	-3.20150400
C	4.28049800	-4.31351200	-3.98499500
C	5.21468800	-3.25837600	-2.03360400
C	4.90580200	-5.51380800	-3.63924500
H	3.66035600	-4.28743100	-4.87603200
C	5.83963600	-4.45661200	-1.68188000
H	5.32461100	-2.41014800	-1.36499200
C	5.69240400	-5.58705800	-2.48877300
H	4.77370300	-6.39183500	-4.26675400
H	6.43649200	-4.50326000	-0.77469100
H	6.18208000	-6.51947200	-2.21886300
S	3.03803700	-2.35529800	1.92765900
O	3.02578300	-2.06208900	3.36419200
O	4.29130400	-2.31992300	1.15478800
C	2.32717800	-3.99677900	1.74666800
C	1.13502700	-4.29500100	2.41573300
C	2.96496200	-4.95425800	0.96238900
C	0.58371400	-5.56501000	2.28427400
H	0.64698600	-3.53922300	3.02350000
C	2.39797400	-6.22587800	0.84472700
H	3.88780300	-4.70608800	0.44989500
C	1.20603200	-6.55184100	1.50059700
H	-0.34593400	-5.79762300	2.79869100
H	2.89352200	-6.97301500	0.22942900
C	0.60281500	-7.93243000	1.38663400
H	-0.46737200	-7.88370100	1.15331900
H	1.09270100	-8.52054100	0.60419500
H	0.70116400	-8.48717300	2.32894100
C	0.20466300	2.72559300	1.50197200
C	0.41506700	3.82000200	2.54771200
H	1.44011100	3.84342400	2.92198400
C	0.03592400	5.20869200	2.04663700
O	-1.06952600	5.53834200	1.67173500

O	1.09334000	6.05291000	2.07763400
C	-2.16621100	0.91896700	2.40791000
C	-1.17497900	-0.08070300	2.18331000
C	-2.83593300	-0.47274100	0.85279000
N	-1.62906300	-0.89113500	1.12166700
C	-3.75421000	-0.99830000	-0.15425600
C	-4.99871700	-0.38973000	-0.38942900
C	-3.38948300	-2.13425000	-0.89751000
C	-5.85993200	-0.90990400	-1.35311100
H	-5.28042900	0.48666700	0.18484300
C	-4.25576100	-2.64825000	-1.85830400
H	-2.42845100	-2.59884100	-0.70376700
C	-5.49279300	-2.03882200	-2.09046500
H	-6.82050200	-0.43316100	-1.52914400
H	-3.96694000	-3.52779600	-2.42751500
H	-6.16716900	-2.44247600	-2.84098800
C	-2.48275200	1.65152400	3.66880600
H	-1.55954300	1.75934000	4.24592200
H	-2.90327900	2.64796900	3.48580000
H	-3.20124800	1.09624700	4.28796800
O	-0.02870700	-0.15190000	2.69958600
H	-0.23049000	3.61551900	3.41064600
C	-1.08176100	2.39863800	1.10387100
H	-1.26616300	1.80048900	0.21632900
H	-1.87275600	3.10485300	1.33709000
C	0.82896300	7.40417000	1.62469800
H	0.00601800	7.80853100	2.22451100
H	0.49156700	7.37182300	0.58472800
C	2.09045000	8.21329500	1.77571700
C	2.74441400	8.29383700	3.01323800
C	2.61283000	8.91989400	0.68683000
C	3.89780900	9.06350000	3.15561600
H	2.35063800	7.74148400	3.86217800
C	3.76386400	9.69946200	0.82915100
H	2.11430300	8.86152100	-0.27796500
C	4.40962900	9.77099100	2.06379600
H	4.39699300	9.11499600	4.11952500
H	4.15537100	10.24624600	-0.02458600
H	5.30658600	10.37392000	2.17672000
O	-3.26080600	0.57425700	1.60394600

***(R)*-H**

C	-2.78695900	0.02183800	1.01747300
H	-3.74365100	-0.50938400	1.00246100
H	-2.46635600	0.12530700	2.05831500
H	-2.94790200	1.02606000	0.61142900
C	-1.74686600	-0.73716600	0.19674000
C	-0.29387300	-0.19349300	0.33420400
C	-0.20557600	1.28243300	-0.09764900
H	-0.70614600	1.91995300	0.64029200
H	-0.75999300	1.37215400	-1.03367300
P	1.49275600	2.03327500	-0.33320600
C	2.45142100	0.87148000	-1.37726900
C	2.83071200	1.22242400	-2.68272300
C	2.82015900	-0.39149600	-0.87780100

C	3.55847200	0.32973500	-3.47191800
H	2.56465500	2.19395300	-3.08396600
C	3.54635600	-1.27974800	-1.67196500
H	2.54579700	-0.69020800	0.12708900
C	3.91600300	-0.92239600	-2.97025100
H	3.84686100	0.61779900	-4.47920100
H	3.81667400	-2.25108800	-1.26804800
H	4.48102900	-1.61608500	-3.58723300
C	1.08280400	3.47792000	-1.44396800
C	0.05192500	3.49078000	-2.40039800
C	1.89532100	4.61617600	-1.31301300
C	-0.16759400	4.61690100	-3.19477000
H	-0.58066900	2.62222000	-2.55206900
C	1.67243800	5.74200600	-2.10952500
H	2.71386700	4.60697700	-0.59745900
C	0.63884700	5.74786900	-3.04665800
H	-0.96859400	4.60902200	-3.92943200
H	2.31086600	6.61409900	-1.99509900
H	0.46365300	6.62563300	-3.66344800
O	-2.07452800	-0.69394000	-1.19888000
Si	-2.69155800	-1.88559000	-2.20670900
C	-1.97149700	-1.47821800	-3.94216300
C	-2.49469500	-2.48164300	-4.99277200
H	-2.21127500	-3.51370600	-4.75108000
H	-3.58544100	-2.44223100	-5.09038200
H	-2.07090700	-2.24847000	-5.98030200
C	-0.42882100	-1.52644900	-3.93534600
H	-0.03978400	-1.21010800	-4.91417300
H	0.00232000	-0.85927000	-3.18070300
H	-0.05100700	-2.53821300	-3.75104800
C	-2.41921500	-0.05362300	-4.34101900
H	-2.05303800	0.69672000	-3.63035600
H	-2.01939800	0.20277100	-5.33286800
H	-3.51045400	0.03582300	-4.38967900
N	0.25620800	-0.32737100	1.68195700
H	0.07164800	0.43689000	2.34129800
H	0.31461600	-0.80351000	-0.33769700
H	-1.72187500	-1.78148500	0.52969700
C	-4.57928100	-1.72524000	-2.26594800
C	-5.40649000	-2.65678500	-2.92655100
C	-5.20609500	-0.60238700	-1.69324400
C	-6.79018500	-2.48342600	-2.99872800
H	-4.97116500	-3.53633100	-3.39465000
C	-6.58950700	-0.42201000	-1.76263400
H	-4.60060400	0.14432600	-1.18914200
C	-7.38642300	-1.36457900	-2.41371700
H	-7.40155400	-3.22129100	-3.51235200
H	-7.04385400	0.45528700	-1.30870200
H	-8.46343200	-1.22773400	-2.46812400
C	-2.17693200	-3.59362700	-1.56590200
C	-3.11229900	-4.60449800	-1.27157200
C	-0.81507500	-3.89392600	-1.35131500
C	-2.71013500	-5.86242600	-0.81600300
H	-4.17353900	-4.40800200	-1.39010700
C	-0.40633700	-5.14733500	-0.89197700
H	-0.05449700	-3.13803600	-1.52286700

C	-1.35447600	-6.13950400	-0.63089900
H	-3.45653800	-6.62422400	-0.60415000
H	0.65017500	-5.34140100	-0.72856700
H	-1.03857600	-7.11815000	-0.27821900
S	0.80071800	-1.72084700	2.37666400
O	1.69237300	-1.29676700	3.46375100
O	1.27221400	-2.59111000	1.28854300
C	-0.59215000	-2.54186100	3.16580900
C	-1.14179600	-1.98290500	4.32449500
C	-1.11413700	-3.71410800	2.62194800
C	-2.22977200	-2.60561900	4.92855100
H	-0.72181300	-1.07469300	4.74522900
C	-2.20487100	-4.32570300	3.24304900
H	-0.67337100	-4.13857300	1.72702000
C	-2.77796100	-3.78633400	4.40087300
H	-2.66112500	-2.16947800	5.82656400
H	-2.61487900	-5.23600900	2.81279300
C	-3.94204100	-4.46477700	5.08409500
H	-4.68099100	-3.73566100	5.43490900
H	-4.44824600	-5.16515300	4.41227100
H	-3.60757200	-5.03348300	5.96192600
C	2.34998700	2.45486200	1.08445500
C	3.79807900	2.05910900	1.26543400
H	4.11020800	1.32124900	0.51720500
C	4.81295000	3.19801400	1.13941800
O	4.66731600	4.24663500	0.54110900
O	5.96445700	2.88647000	1.78438800
C	1.36186700	3.25647900	3.44066400
C	-0.12767200	2.92219700	3.55798200
C	0.12865800	4.85963900	4.44631100
N	-0.79819600	3.98846500	4.14569800
C	-0.11181300	6.14550700	5.10284100
C	0.95010100	7.02050800	5.38258200
C	-1.42484800	6.49840700	5.45574400
C	0.69697900	8.23764400	6.01051900
H	1.96053000	6.74004000	5.10548700
C	-1.66943100	7.71649600	6.08274100
H	-2.22983400	5.80711900	5.23028900
C	-0.61039100	8.58657000	6.36093600
H	1.51829600	8.91483800	6.22667200
H	-2.68473700	7.98965800	6.35563900
H	-0.80425700	9.53703300	6.85082400
C	2.24595300	2.28341000	4.20785800
H	2.20095100	1.29657100	3.73973000
H	3.28307700	2.63214000	4.21312500
H	1.90759900	2.19131500	5.24549400
O	-0.66133400	1.88088500	3.18821300
H	4.00050200	1.56617500	2.22500800
C	1.82590900	3.56862600	1.96250000
H	0.99600600	4.11105400	1.48931500
H	2.61176100	4.32571100	2.09881200
C	7.02099400	3.86725400	1.69898000
H	6.61804100	4.83456500	2.02143100
H	7.32740600	3.97874600	0.65366800
C	8.17154900	3.42271300	2.56562900
C	7.95400900	2.97596000	3.87604200

C	9.48285800	3.49220200	2.08339700
C	9.02770900	2.60793600	4.68542200
H	6.93776700	2.90364300	4.25270400
C	10.56095700	3.13308100	2.89572700
H	9.66279600	3.82769200	1.06438600
C	10.33554200	2.68898800	4.19891300
H	8.84439300	2.25710000	5.69762900
H	11.57380800	3.19235700	2.50606700
H	11.17195800	2.40314100	4.83115800
O	1.39515300	4.55401000	4.13650900

Ts5-*si*

C	0.78378800	-3.62470600	0.13976200
H	0.94122400	-4.67384500	-0.13134900
H	0.32755900	-3.58312000	1.13288600
H	0.07122900	-3.19430600	-0.57183600
C	2.11330400	-2.87481100	0.11606000
C	2.05326200	-1.41679300	0.65659000
C	1.01681500	-0.59278900	-0.14024500
H	0.01487300	-0.87673300	0.19885900
H	1.13486500	-0.85352800	-1.19331500
P	1.16157300	1.24981300	0.00238100
C	2.64542200	1.72335000	0.94599200
C	3.89708900	1.86223100	0.32060400
C	2.54623500	1.86024200	2.33951400
C	5.02607000	2.16518100	1.08026200
H	3.99346800	1.73843400	-0.75300800
C	3.68064400	2.16658400	3.09065300
H	1.59943800	1.69160600	2.84134700
C	4.91656400	2.32539700	2.46346100
H	5.99003300	2.27085700	0.59085400
H	3.59432100	2.25050100	4.16864500
H	5.79892400	2.55691400	3.05364000
C	1.40216400	1.89724500	-1.70600200
C	1.96561100	3.17095200	-1.90028500
C	0.89243900	1.20860300	-2.81987400
C	2.04405300	3.72422400	-3.17880200
H	2.34084200	3.73924900	-1.05661500
C	0.97404600	1.76447100	-4.09670300
H	0.41833100	0.23971400	-2.70621000
C	1.55442000	3.02028800	-4.28055700
H	2.48380400	4.70915300	-3.30680100
H	0.58271100	1.21222500	-4.94626400
H	1.61955200	3.44996900	-5.27634400
O	2.63275500	-2.78995800	-1.21893200
Si	3.78433700	-3.72466400	-2.00702000
C	4.83102200	-2.47300700	-3.02904800
C	5.95229100	-3.20242200	-3.79987500
H	6.62468000	-3.74793900	-3.12633200
H	5.55230600	-3.91353100	-4.53147300
H	6.56266300	-2.47549800	-4.35527100
C	5.47373800	-1.41965200	-2.10249900
H	6.02697500	-0.68013100	-2.70026700
H	4.72173700	-0.87750000	-1.51764700
H	6.18534500	-1.87043300	-1.40228900
C	3.91021200	-1.75284800	-4.03866800

H	3.11201400	-1.19632400	-3.53391100
H	4.49030600	-1.03122400	-4.63279600
H	3.44104000	-2.45530200	-4.73705400
N	1.71899900	-1.30878000	2.07224500
H	0.71267800	-1.44616300	2.30339200
H	3.05397800	-1.00199200	0.51366900
H	2.83129600	-3.40345900	0.75083000
C	2.88059500	-4.90668300	-3.18032900
C	3.55309600	-5.86310000	-3.96850500
C	1.48342100	-4.82017900	-3.32688400
C	2.86421400	-6.69656900	-4.85193300
H	4.63269200	-5.96802500	-3.89428300
C	0.78800400	-5.65020500	-4.20911900
H	0.93294200	-4.09092800	-2.74050800
C	1.47720500	-6.59226300	-4.97399800
H	3.41030300	-7.42677100	-5.44407000
H	-0.29204400	-5.56156500	-4.29780700
H	0.93864100	-7.24056000	-5.66053200
C	4.82367700	-4.68351500	-0.74798400
C	5.00913300	-6.07711100	-0.83408600
C	5.44212700	-4.01500000	0.33000500
C	5.79261400	-6.76805800	0.09310600
H	4.52766200	-6.63857100	-1.62927500
C	6.22044200	-4.70216600	1.26398000
H	5.29895400	-2.94840200	0.47350600
C	6.40395200	-6.08134800	1.14313600
H	5.91903800	-7.84376300	-0.00210200
H	6.67479000	-4.15603000	2.08636600
H	7.01262800	-6.61782300	1.86682100
S	2.80464900	-1.69930500	3.27476100
O	2.68043500	-0.71908400	4.35886100
O	4.10930100	-1.90680600	2.62219200
C	2.22771100	-3.26355200	3.94109500
C	1.02020100	-3.29247800	4.64906400
C	2.97925800	-4.42069700	3.74963100
C	0.56908400	-4.50633700	5.15712000
H	0.44424800	-2.38211400	4.78798000
C	2.51083500	-5.62733000	4.27485700
H	3.91330300	-4.37641300	3.19961100
C	1.30482000	-5.69063700	4.98187300
H	-0.37232300	-4.53672900	5.70091300
H	3.09456600	-6.53289500	4.12825700
C	0.80074200	-6.99512900	5.55359900
H	-0.21916700	-7.21176200	5.21350300
H	1.43906900	-7.83435400	5.26046900
H	0.77296600	-6.96400300	6.65043500
C	-0.30359800	1.99373100	0.69638600
C	-0.23127400	3.44960300	1.15231700
H	0.77397300	3.73949400	1.46336200
C	-0.73412100	4.43648500	0.10314700
O	-1.83871500	4.42711800	-0.39472500
O	0.21648400	5.35035800	-0.20901300
C	-2.51013200	0.36192200	2.49399700
C	-1.41832100	-0.51286100	2.74589800
C	-1.29224300	1.13595200	4.13991600
N	-0.65964400	0.05339200	3.78246600

C	-0.87231800	2.09359200	5.15988800
C	-1.64178600	3.23074500	5.45814200
C	0.33280800	1.86828500	5.85144000
C	-1.21160300	4.12848000	6.43384300
H	-2.57375100	3.39879300	4.92786900
C	0.75176000	2.77018700	6.82610600
H	0.92284100	0.98947200	5.60762400
C	-0.01602600	3.90193800	7.12064200
H	-1.81254500	5.00522900	6.66068300
H	1.68109500	2.58943500	7.35991200
H	0.31535100	4.60281400	7.88238400
C	-3.85104300	0.04870900	1.92957300
H	-3.75573400	-0.85042800	1.31205900
H	-4.59031300	-0.15960300	2.71566600
H	-4.25688600	0.85596400	1.30314600
O	-1.06040400	-1.54645200	2.11095700
H	-0.88332000	3.56735200	2.02616100
C	-1.53004900	1.37359400	0.59770700
H	-1.65764100	0.41883600	0.10014300
H	-2.41756500	1.99446600	0.65076900
C	-0.16244700	6.33927100	-1.19432500
H	-1.00512200	6.91471100	-0.79254500
H	-0.51614200	5.82957700	-2.09508600
C	1.03084400	7.21453100	-1.48259700
C	1.31427900	7.59945700	-2.79843200
C	1.84812000	7.68698100	-0.44663600
C	2.38967800	8.44675900	-3.07692700
H	0.68784800	7.23633800	-3.61027500
C	2.92749900	8.52527000	-0.72428000
H	1.64184100	7.38443500	0.57574900
C	3.20038700	8.90978500	-2.03987200
H	2.59556400	8.73850900	-4.10335400
H	3.55631700	8.88059100	0.08776000
H	4.04071400	9.56453200	-2.25413500
O	-2.43859100	1.38047300	3.46066100

***(S)*-H**

C	0.61849100	-3.55765700	0.04673100
H	0.80518600	-4.59821200	-0.23759700
H	0.17565700	-3.54959300	1.04749300
H	-0.11523500	-3.14132400	-0.65119400
C	1.92453300	-2.76611600	0.01897600
C	1.81516400	-1.30441400	0.54245800
C	0.79409500	-0.49428400	-0.28610500
H	-0.22176900	-0.81737200	-0.04048400
H	0.99144400	-0.74169600	-1.33072700
P	0.85843300	1.36370300	-0.12990300
C	2.52969300	1.80887600	0.48160700
C	3.62801400	1.94585600	-0.38348500
C	2.73912400	1.89148200	1.86782800
C	4.90317900	2.19143000	0.12863400
H	3.49259600	1.86293400	-1.45641000
C	4.01515400	2.13820500	2.37433600
H	1.90776800	1.73330900	2.54533700
C	5.09735000	2.29484700	1.50707800
H	5.74315800	2.29802500	-0.55237000

H	4.16105500	2.18419600	3.44929700
H	6.09169200	2.48095000	1.90422300
C	0.84704800	1.91920300	-1.91882700
C	1.33379800	3.19212300	-2.26977500
C	0.15599500	1.18577400	-2.90011500
C	1.17081200	3.69308500	-3.56192300
H	1.84134300	3.80389100	-1.53171400
C	-0.01225900	1.68870500	-4.19171500
H	-0.26687000	0.21315200	-2.66713000
C	0.50059100	2.94139800	-4.52969900
H	1.56543700	4.67557100	-3.80676300
H	-0.54290000	1.09679600	-4.93299600
H	0.37438500	3.33110700	-5.53630100
O	2.44750300	-2.68879000	-1.31249100
Si	3.62082000	-3.62186600	-2.07088100
C	4.58709900	-2.39776500	-3.19366300
C	5.70038300	-3.13617500	-3.96782800
H	6.41594600	-3.62146300	-3.29230900
H	5.29599800	-3.90004900	-4.64149000
H	6.26458200	-2.42332100	-4.58641600
C	5.23118800	-1.27304100	-2.35564300
H	5.71282200	-0.54083400	-3.02074000
H	4.49164100	-0.73455700	-1.75271300
H	6.00570500	-1.65486400	-1.68110000
C	3.60458800	-1.76229100	-4.20342000
H	2.81023500	-1.20065200	-3.69872700
H	4.14021000	-1.05994800	-4.85885600
H	3.13010000	-2.51495700	-4.84367100
N	1.47560900	-1.21631500	1.96420500
H	0.50192200	-1.40247700	2.21045000
H	2.80764800	-0.86550000	0.42483900
H	2.65396300	-3.26833400	0.66381700
C	2.75251500	-4.91380100	-3.15220400
C	3.45090400	-5.91647900	-3.85584800
C	1.35676500	-4.86013300	-3.32672700
C	2.78808600	-6.82683700	-4.68158900
H	4.53124200	-5.99648500	-3.76227400
C	0.68737600	-5.76674200	-4.15190500
H	0.78664400	-4.09382000	-2.81059900
C	1.40170300	-6.75496700	-4.83064200
H	3.35444400	-7.59081300	-5.20847300
H	-0.39203900	-5.70048400	-4.26472200
H	0.88328900	-7.46250700	-5.47274400
C	4.71909900	-4.45228100	-0.76869200
C	4.93390300	-5.84383900	-0.73903600
C	5.35101400	-3.68086300	0.22978500
C	5.75924200	-6.43597300	0.21992000
H	4.44313600	-6.48134700	-1.46830800
C	6.17301500	-4.26761600	1.19431500
H	5.18614300	-2.60912400	0.28347400
C	6.38561200	-5.64819200	1.18687400
H	5.90825400	-7.51299500	0.21323100
H	6.63885000	-3.64348700	1.95228400
H	7.02945700	-6.10651600	1.93351800
S	2.56207700	-1.55326900	3.18642800
O	2.29489500	-0.62502900	4.29127700

O	3.89417500	-1.60885000	2.56892900
C	2.12954200	-3.19027400	3.78816500
C	0.93372800	-3.36209600	4.49602500
C	2.98250900	-4.26480900	3.54488200
C	0.59715500	-4.63390300	4.94919700
H	0.27723000	-2.51622900	4.68010700
C	2.62840100	-5.53138200	4.01483400
H	3.90686900	-4.11202700	2.99832300
C	1.43676000	-5.73702300	4.71941800
H	-0.33350300	-4.77443200	5.49418700
H	3.29232600	-6.37134300	3.82611200
C	1.05712500	-7.10680100	5.23110100
H	0.05684500	-7.39739900	4.88799700
H	1.76396000	-7.87084100	4.89325800
H	1.03949100	-7.12997700	6.32818800
C	-0.36196800	1.98205900	0.91502700
C	-0.22861600	3.42807200	1.41095200
H	0.81725700	3.70990100	1.55325800
C	-0.88235400	4.45490100	0.49852400
O	-2.07838000	4.59333600	0.32608200
O	0.03930300	5.21768700	-0.15062100
C	-2.41267500	0.85672600	2.10285400
C	-1.64115700	-0.37171100	2.57606800
C	-1.40859500	1.11995500	4.09682700
N	-1.07525900	-0.11312400	3.81325200
C	-0.98013400	1.85429200	5.28585300
C	-1.59981200	3.06388800	5.64187100
C	0.06999500	1.33947400	6.06554900
C	-1.18137900	3.74511400	6.78183000
H	-2.40567000	3.45387800	5.02907100
C	0.48498900	2.03338100	7.19936100
H	0.56694100	0.42787500	5.75077000
C	-0.14130800	3.22991700	7.56137200
H	-1.66391700	4.67693300	7.06260200
H	1.30335500	1.64366800	7.79780000
H	0.18540700	3.76550100	8.44889600
C	-3.90665900	0.58925800	1.94637500
H	-4.05286700	-0.18165100	1.18334100
H	-4.34587600	0.23392600	2.88412800
H	-4.42642200	1.50062400	1.63502000
O	-1.49424700	-1.40266400	1.92746800
H	-0.73199700	3.52424300	2.38020600
C	-1.78447900	1.46088800	0.80808800
H	-1.88427600	0.65849200	0.06641500
H	-2.46628700	2.25825400	0.48244800
C	-0.49008000	6.19182400	-1.07136600
H	-1.24900800	6.78515900	-0.54754700
H	-0.99574800	5.67438900	-1.89292300
C	0.63869600	7.05447200	-1.57968100
C	0.60837100	7.53247800	-2.89577400
C	1.70020200	7.43257100	-0.74695900
C	1.61246400	8.37887800	-3.37062400
H	-0.20805600	7.24140100	-3.55318500
C	2.70890900	8.27072700	-1.22321400
H	1.73782800	7.05605000	0.27047800
C	2.66730400	8.74949800	-2.53486600

H	1.57319000	8.74167900	-4.39442700
H	3.52933800	8.55095400	-0.56762100
H	3.45326100	9.40317200	-2.90358400
O	-2.20944300	1.76303200	3.23193300

***(R)*-9a**

C	0.58153000	3.51384900	2.35134000
C	0.90054200	4.80250000	2.17884400
C	2.03635000	5.41370700	2.91029000
O	2.77175900	4.84278000	3.69197700
O	2.15371600	6.72552800	2.58982400
C	-1.56232000	2.14441600	2.55614800
C	-0.98542100	0.88836700	3.24948900
C	-2.55277400	0.22379100	1.92297300
N	-1.67997500	-0.23262000	2.77085100
C	-3.50825100	-0.59261500	1.17229300
C	-4.41881300	-0.00208900	0.28175500
C	-3.50607200	-1.98546300	1.35486200
C	-5.31923700	-0.80115000	-0.41894100
H	-4.41425400	1.07406100	0.14695100
C	-4.40864000	-2.77678900	0.65081300
H	-2.79413600	-2.42051400	2.04811900
C	-5.31538600	-2.18665300	-0.23555100
H	-6.02399700	-0.34452500	-1.10787800
H	-4.40715900	-3.85375500	0.79178700
H	-6.01926000	-2.80724500	-0.78351800
C	-2.21383700	3.10045000	3.54884300
H	-1.45993100	3.44781100	4.26078600
H	-2.63927800	3.96679900	3.03159100
H	-3.00992900	2.59522200	4.10430100
O	-0.08593800	0.89339200	4.06276800
H	0.35093300	5.44884400	1.49934900
C	-0.53706300	2.82661000	1.62190500
H	-0.12046600	2.05321700	0.96054600
H	-1.07731600	3.53828400	0.98630800
H	1.16238400	2.91870700	3.05309700
C	3.23678400	7.42958600	3.24997100
H	3.11675300	7.31641600	4.33161800
H	4.18179100	6.95397700	2.96951600
C	3.18480200	8.87208800	2.82492800
C	2.41490800	9.79661600	3.54177600
C	3.89107600	9.30853400	1.69736900
C	2.34968100	11.13100000	3.13960700
H	1.86528100	9.46694900	4.42049700
C	3.82894800	10.64220800	1.29179200
H	4.49339900	8.59797300	1.13600200
C	3.05728800	11.55604700	2.01288600
H	1.75098900	11.83889400	3.70657700
H	4.38448400	10.96855900	0.41665000
H	3.01090900	12.59596000	1.70062200
O	-2.59399600	1.55969900	1.70819700

***(S)*-9a**

C	0.58207500	3.51361200	-2.35060600
C	0.90124000	4.80220500	-2.17792300
C	2.03690700	5.41345600	-2.90953400

O	2.77204400	4.84259100	-3.69153300
O	2.15421900	6.72527800	-2.58918900
C	-1.56197000	2.14455000	-2.55550300
C	-0.98530500	0.88861900	-3.24930700
C	-2.55276900	0.22387900	-1.92295700
N	-1.68009800	-0.23240100	-2.77106200
C	-3.50843200	-0.59261100	-1.17254000
C	-4.41882100	-0.00220100	-0.28173300
C	-3.50661700	-1.98539000	-1.35568100
C	-5.31943800	-0.80131800	0.41864900
H	-4.41397500	1.07389900	-0.14649800
C	-4.40937700	-2.77676100	-0.65194300
H	-2.79481200	-2.42033100	-2.04914600
C	-5.31594900	-2.18674700	0.23468000
H	-6.02406800	-0.34479500	1.10778500
H	-4.40819200	-3.85366600	-0.79336100
H	-6.01998000	-2.80737900	0.78240200
C	-2.21344200	3.10098600	-3.54785100
H	-1.45953800	3.44841700	-4.25976500
H	-2.63868700	3.96725400	-3.03030200
H	-3.00967100	2.59603800	-4.10338100
O	-0.08588800	0.89377700	-4.06263000
H	0.35179800	5.44847600	-1.49823400
C	-0.53650900	2.82633600	-1.62117800
H	-0.11992700	2.05269200	-0.96009500
H	-1.07658900	3.53790600	-0.98531800
H	1.16271500	2.91855800	-3.05261700
C	3.23691300	7.42943300	-3.24983700
H	3.11656900	7.31598700	-4.33142100
H	4.18214400	6.95413400	-2.96960400
C	3.18471700	8.87202500	-2.82514500
C	2.41417300	9.79612000	-3.54183800
C	3.89141700	9.30898100	-1.69805300
C	2.34871300	11.13058400	-3.13998000
H	1.86420300	9.46605500	-4.42018800
C	3.82907500	10.64274900	-1.29278600
H	4.49425600	8.59876100	-1.13680600
C	3.05675300	11.55615700	-2.01372600
H	1.74949900	11.83813400	-3.70682300
H	4.38495500	10.96950200	-0.41801100
H	3.01019500	12.59613400	-1.70170500
O	-2.59366700	1.55973800	-1.70763300

Ts4-na

C	-5.54190508	0.22110182	0.15157467
H	-6.52652719	0.69449149	0.07885993
H	-5.32452171	0.01762649	1.20395827
H	-4.79715106	0.93356320	-0.21827510
C	-5.52372864	-1.07938491	-0.64562623
C	-4.23204411	-1.92547425	-0.46787180
C	-2.98737133	-1.09145710	-0.83613954
H	-2.75271200	-0.40982950	-0.01633363
H	-3.24983081	-0.49426794	-1.71200681
P	-1.44513807	-2.02811348	-1.25933488
C	-1.59656515	-3.79561575	-0.81651468
C	-2.35575399	-4.67437668	-1.60999518
C	-0.98288700	-4.27411416	0.35030042
C	-2.48523605	-6.01307646	-1.24402212
H	-2.84225905	-4.32208223	-2.51442052
C	-1.10739679	-5.61775638	0.70177741
H	-0.46455207	-3.58150934	1.00272712
C	-1.85394532	-6.48750655	-0.09262471
H	-3.08368244	-6.68158544	-1.85604721
H	-0.63964505	-5.97732562	1.61337334
H	-1.95959438	-7.53032933	0.19303195
C	-1.29886683	-2.01782692	-3.10368859
C	-0.65494876	-3.06587836	-3.78241285
C	-1.65290742	-0.86701903	-3.82914632
C	-0.40495637	-2.97867522	-5.15365582
H	-0.34748723	-3.95717489	-3.24670777
C	-1.41012004	-0.78471500	-5.19923304
H	-2.10672537	-0.01613380	-3.33127889
C	-0.78896932	-1.84291471	-5.86682194
H	0.09422673	-3.80102543	-5.65848743
H	-1.70268758	0.10912226	-5.74253725
H	-0.60126548	-1.77805486	-6.93515662
O	-5.67100995	-0.83580215	-2.05091461
Si	-7.07232491	-0.80482927	-2.97968836
C	-6.60977793	-1.65798515	-4.64139875
C	-7.83843525	-1.72709164	-5.57391910
H	-8.66571886	-2.28851697	-5.12285570
H	-8.20797215	-0.72999538	-5.83958130
H	-7.57051111	-2.23358404	-6.51261918
C	-6.09100543	-3.09087934	-4.39738937
H	-5.76824160	-3.54001391	-5.34809961
H	-5.23165564	-3.10166642	-3.71726239
H	-6.86568835	-3.74146688	-3.97725690
C	-5.49703236	-0.83968235	-5.33267440
H	-4.59264765	-0.78828195	-4.71630095
H	-5.21876131	-1.31137527	-6.28663818
H	-5.81790107	0.18534683	-5.55110236
N	-4.10834565	-2.41225167	0.90598817
H	-3.23843206	-2.20251453	1.43119781
H	-4.33295413	-2.77001392	-1.15754337
H	-6.34669467	-1.71365839	-0.30220203
C	-7.52359403	1.00718331	-3.30092672
C	-8.69069866	1.39301089	-3.99173274
C	-6.65044979	2.03228794	-2.89119237
C	-8.97634518	2.73494988	-4.25114502

H	-9.39628225	0.63901204	-4.33201055
C	-6.92927877	3.37655629	-3.14808529
H	-5.73791220	1.77237917	-2.36331886
C	-8.09489352	3.73186287	-3.82837092
H	-9.88634561	3.00129944	-4.78312541
H	-6.23628043	4.14572830	-2.81600986
H	-8.31517506	4.77732321	-4.02876781
C	-8.47759160	-1.69985974	-2.07615614
C	-9.71554403	-1.08002508	-1.81794926
C	-8.31799374	-3.03130392	-1.63557638
C	-10.75427578	-1.76024178	-1.17786937
H	-9.87284102	-0.04652266	-2.11157096
C	-9.35185423	-3.71483841	-0.99199087
H	-7.36941002	-3.54361825	-1.76402943
C	-10.57683841	-3.08226808	-0.76761265
H	-11.69953743	-1.25472539	-0.99603451
H	-9.19431675	-4.73886001	-0.66350732
H	-11.38436536	-3.61453225	-0.27103485
S	-4.93185179	-3.75155970	1.44355372
O	-3.99508596	-4.61968514	2.16312128
O	-5.73089758	-4.26058516	0.31607317
C	-6.07097148	-3.11039762	2.67739136
C	-5.57111136	-2.72600227	3.92598849
C	-7.42961052	-3.00498509	2.38936615
C	-6.44724066	-2.22267706	4.88216375
H	-4.51201076	-2.82515417	4.14162868
C	-8.29493102	-2.50045629	3.36304839
H	-7.80519865	-3.31789293	1.42127376
C	-7.82210609	-2.10215056	4.61837806
H	-6.05961495	-1.92096503	5.85262444
H	-9.35561226	-2.41826185	3.13815401
C	-8.76126005	-1.56890318	5.67493773
H	-8.40546195	-0.61452610	6.08124271
H	-9.76712464	-1.41055422	5.27361561
H	-8.84586910	-2.26531750	6.51918659
C	-0.00226224	-1.26081330	-0.60087732
C	1.31694725	-1.93753633	-0.94275883
H	1.22523789	-3.02129889	-1.05771538
C	1.99738900	-1.37266824	-2.19119487
O	2.04087545	-0.20272695	-2.50229530
O	2.59208503	-2.35805194	-2.90392261
H	2.02702342	-1.78499199	-0.11944526
C	0.00000000	0.00000000	0.00000000
H	-0.86364365	0.65443590	-0.06566894
H	0.94123004	0.54219136	-0.01446046
C	3.30077241	-1.92704341	-4.09168459
H	4.07377107	-1.21185630	-3.78792890
H	2.60318459	-1.39860418	-4.74740921
C	3.88650360	-3.14067798	-4.76449888
C	4.73371033	-4.00844002	-4.06108294
C	3.61278060	-3.40574127	-6.11050769
C	5.29265434	-5.11825993	-4.69232937
H	4.94420691	-3.81516477	-3.01259284
C	4.17892405	-4.51249578	-6.74837399
H	2.95414575	-2.73998485	-6.66355517
C	5.01870696	-5.37184000	-6.03970984
H	5.94513721	-5.78513956	-4.13499757

H	3.95978108	-4.70379961	-7.79551864
H	5.45783161	-6.23524968	-6.53225039

12. Reference

1. (a) Han, X.; Wang, Y.; Zhong, F.; Lu, Y. *J. Am. Chem. Soc.* **2011**, *133*, 1726; (b) Han, X.; Zhong, F.; Wang, Y.; Lu, Y. *Angew. Chem. Int. Ed.* **2012**, *51*, 767; (c) Zhong, F.; Han, X.; Wang, Y.; Lu, Y. *Chem. Sci.* **2012**, *3*, 1231; (d) Zhong, F.; Han, X.; Wang, Y.; Lu, Y. *Angew. Chem. Int. Ed.* **2011**, *50*, 7837; (e) Zhong, F.; Luo, J.; Chen, G.-Y.; Dou, X.; Lu, Y. *J. Am. Chem. Soc.* **2012**, *134*, 10222; (f) Zhong, F.; Dou, X.; Han, X.; Yao, W.; Zhu, Q.; Meng, Y.; Lu, Y. *Angew. Chem. Int. Ed.* **2013**, *52*, 943; (g) Wang, T.; Yao, W.; Zhong, F.; Pang, G. H.; Lu, Y. *Angew. Chem. Int. Ed.* **2014**, *53*, 2964.
2. (a) Kerdesky, F. A. J.; Holms, J. H.; Moore, J. L.; Bell, R. L.; Dyer, R. D.; Carter, G. W.; Brooks, D. W. *J. Med. Chem.* **1991**, *34*, 2158; (b) Diosdado, S.; Etxabe, J.; Izquierdo, J.; Landa, A.; Mielgo, A.; Olaizola, I.; López, R.; Palomo, C. *Angew. Chem. Int. Ed.* **2013**, *52*, 11846; (c) Shaffer, J. E.; Thomson, S. A.; *US Patent* 5.087.631 Feb 11, **1992**; (d) Chen, W.; Hartwig, J. F. *J. Am. Chem. Soc.* **2014**, *136*, 377.
3. Keto-enol-tautomerism has been found by ^{1}H-NMR using $CDCl_3$ as solvent. Using DMSO-d_6 as solvent, only the enol form was observed.
4. (a) Trost, B. M.; Dogra, K.; Franzin, M. *J. Am. Chem. Soc.* **2004**, *126*, 1944; (b) Misaki, T.; Takimotoa, G.; Sugimura, T. *J. Am. Chem. Soc.* **2010**, *132*, 6286, and the references cited therein; (c) Jacobsen, N. W.; Philippides, A. *Aust. J. Chem.*, **1985**, *38*, 1335.
5. J. L. G. Ruano, A. M. M. Castro, J. H. Rodríguez, *J. Org. Chem.* **1992**, *57*, 7235.

13. NMR Spectra of the Products

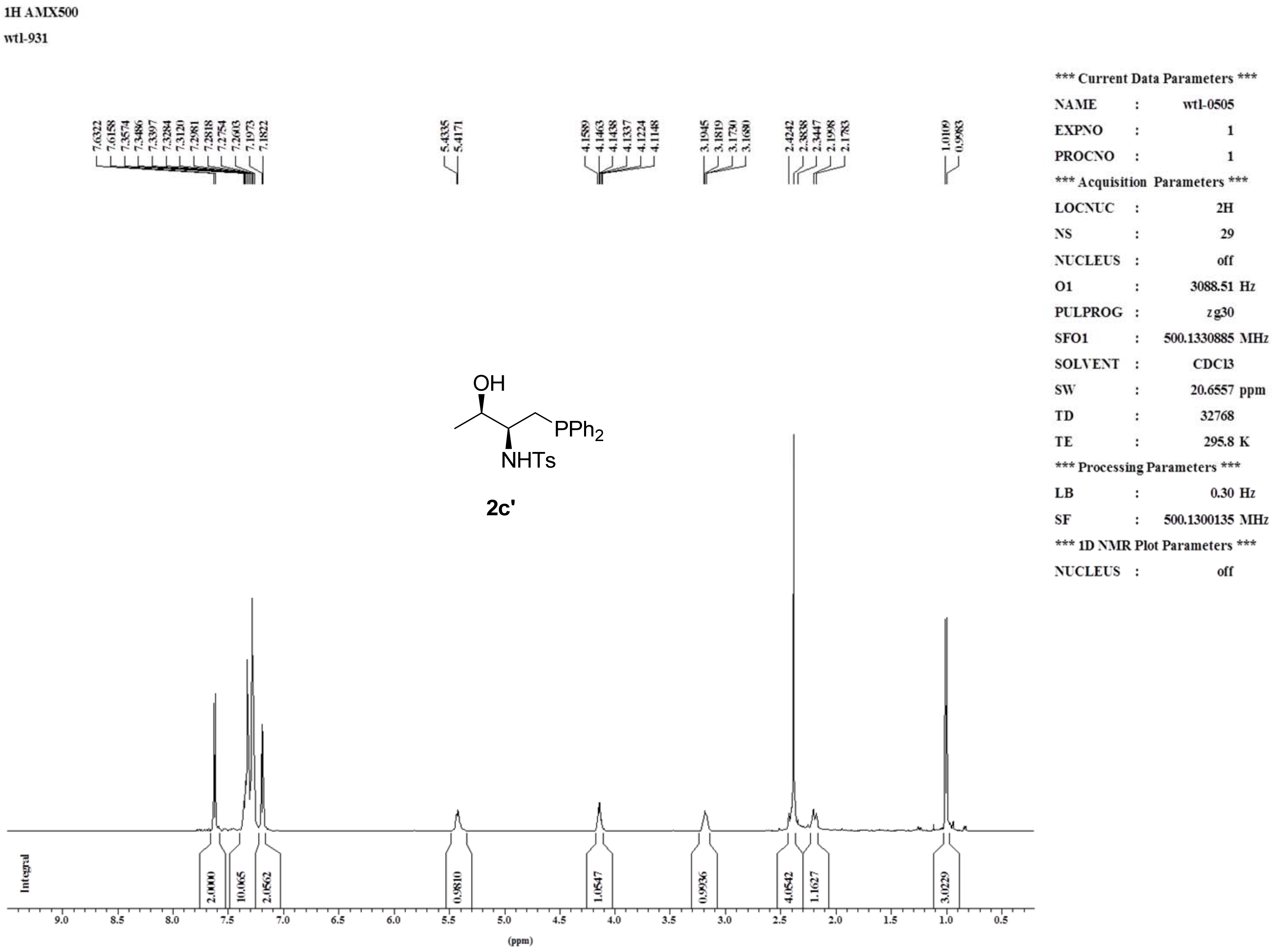

13C AMX500

wtl-931

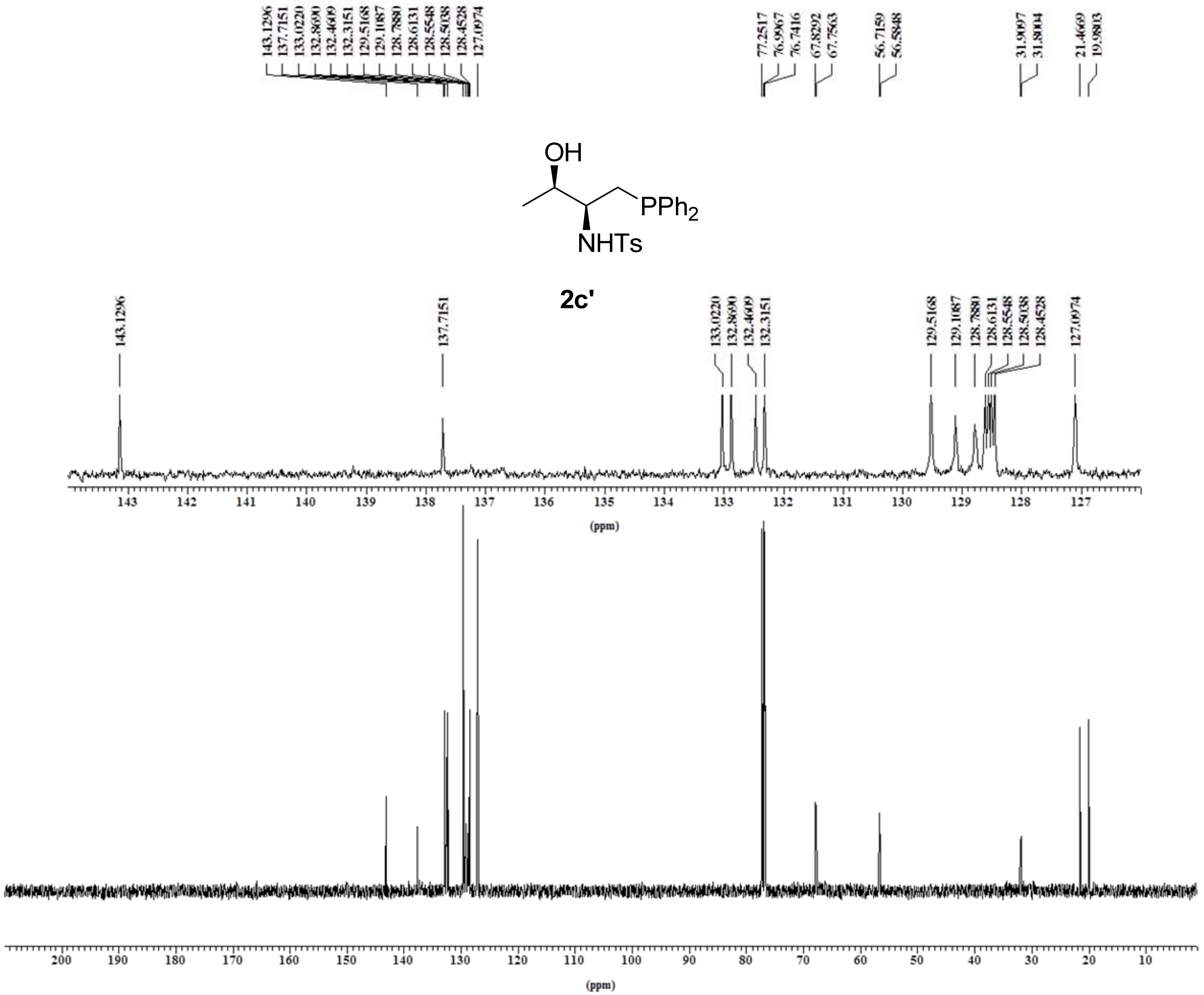

2c'

*** Current Data Parameters ***

NAME : wtl-0505

EXPNO : 2

PROCNO : 1

*** Acquisition Parameters ***

LOCNUC : 2H

NS : 88

NUCLEUS : off

O1 : 13204.57 Hz

PULPROG : zgpg30

SFO1 : 125.7709936 MHz

SOLVENT : CDCl3

SW : 238.7675 ppm

TD : 65536

TE : 295.9 K

*** Processing Parameters ***

LB : 1.00 Hz

SF : 125.7577997 MHz

*** 1D NMR Plot Parameters ***

NUCLEUS : off

31p AMX500

wtl-931

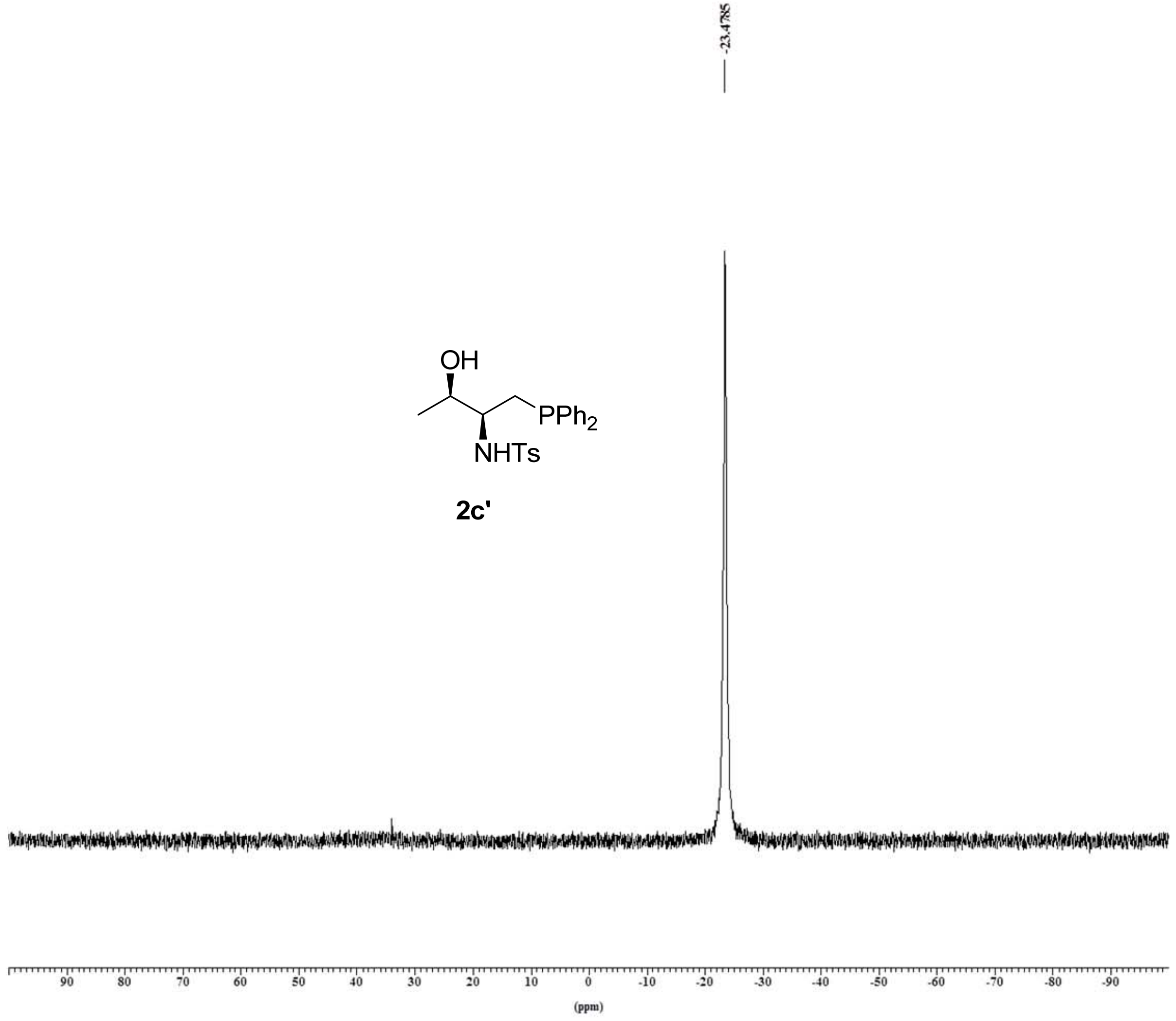

2c'

*** Current Data Parameters ***

NAME	:	wtl-0505
EXPNO	:	3
PROCNO	:	1

*** Acquisition Parameters ***

LOCNUC	:	2H
NS	:	50
NUCLEUS	:	off
O1	:	-10122.85 Hz
PULPROG	:	zgpg
SFO1	:	202.4462121 MHz
SOLVENT	:	CDCl3
SW	:	401.5922 ppm
TD	:	65536
TE	:	296.1 K

*** Processing Parameters ***

LB	:	1.00 Hz
SF	:	202.4562131 MHz

*** 1D NMR Plot Parameters ***

NUCLEUS	:	off

1H AMX500

wtl-937-2

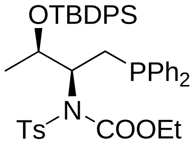

2c''
(dr = 85/15)

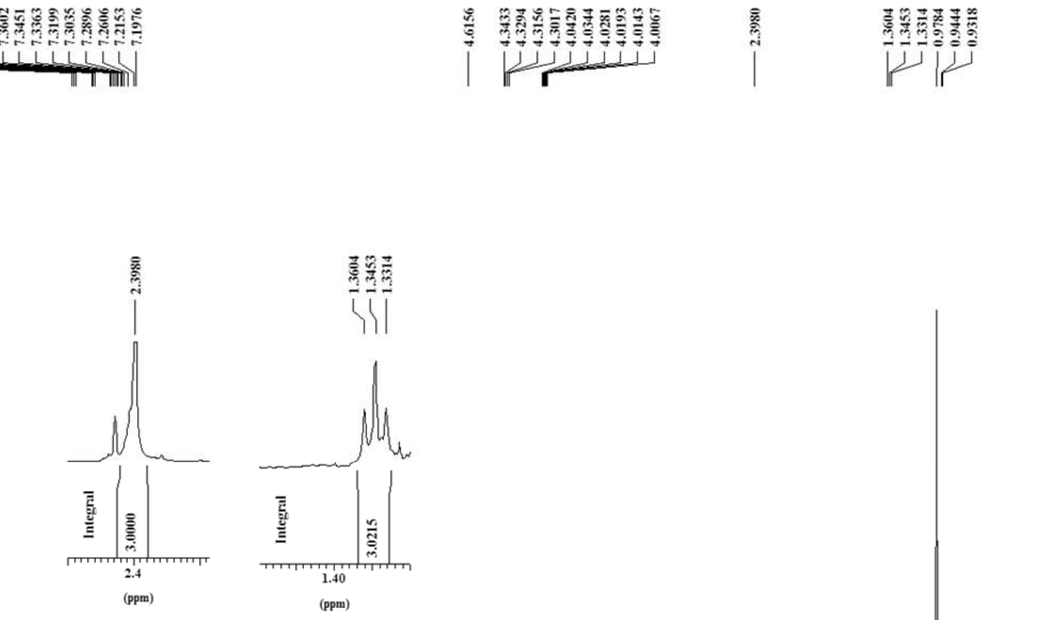

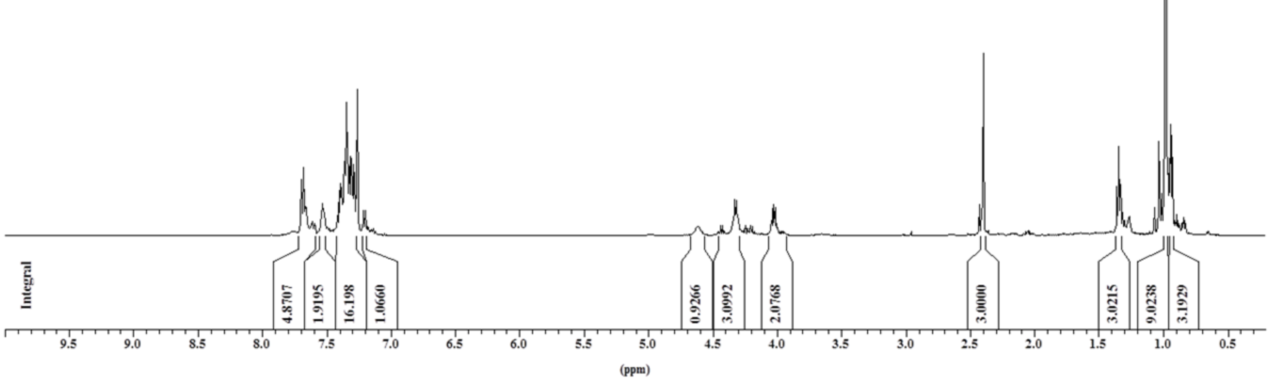

*** Current Data Parameters ***

NAME	:	wtl-0507
EXPNO	:	6
PROCNO	:	1

*** Acquisition Parameters ***

LOCNUC	:	2H
NS	:	60
NUCLEUS	:	off
O1	:	3088.51 Hz
PULPROG	:	zg30
SFO1	:	500.1330885 MHz
SOLVENT	:	CDCl3
SW	:	20.6557 ppm
TD	:	32768
TE	:	296.2 K

*** Processing Parameters ***

LB	:	0.30 Hz
SF	:	500.1300134 MHz

*** 1D NMR Plot Parameters ***

NUCLEUS	:	off

13C AMX500

wtl-937-2

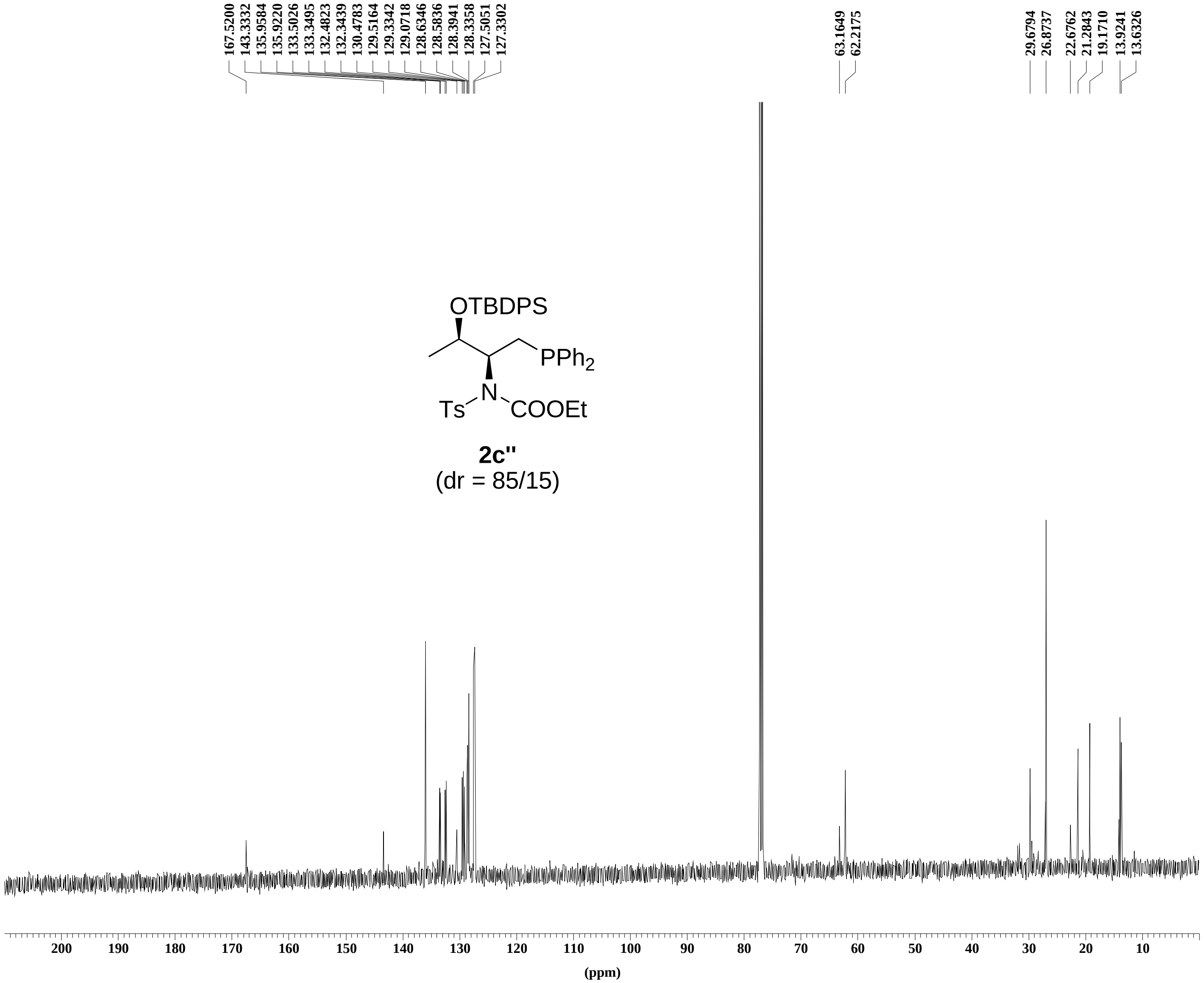

2c''
(dr = 85/15)

*** Current Data Parameters ***		
NAME	:	wtl-0507
EXPNO	:	3
PROCNO	:	1
*** Acquisition Parameters ***		
LOCNUC	:	2H
NS	:	1109
NUCLEUS	:	off
O1	:	13204.57 Hz
PULPROG	:	zgpg30
SFO1	:	125.7709936 MHz
SOLVENT	:	CDCl3
SW	:	238.7675 ppm
TD	:	65536
TE	:	296.6 K
*** Processing Parameters ***		
LB	:	1.00 Hz
SF	:	125.7577925 MHz
*** 1D NMR Plot Parameters ***		
NUCLEUS	:	off

31p

wtl-937-2

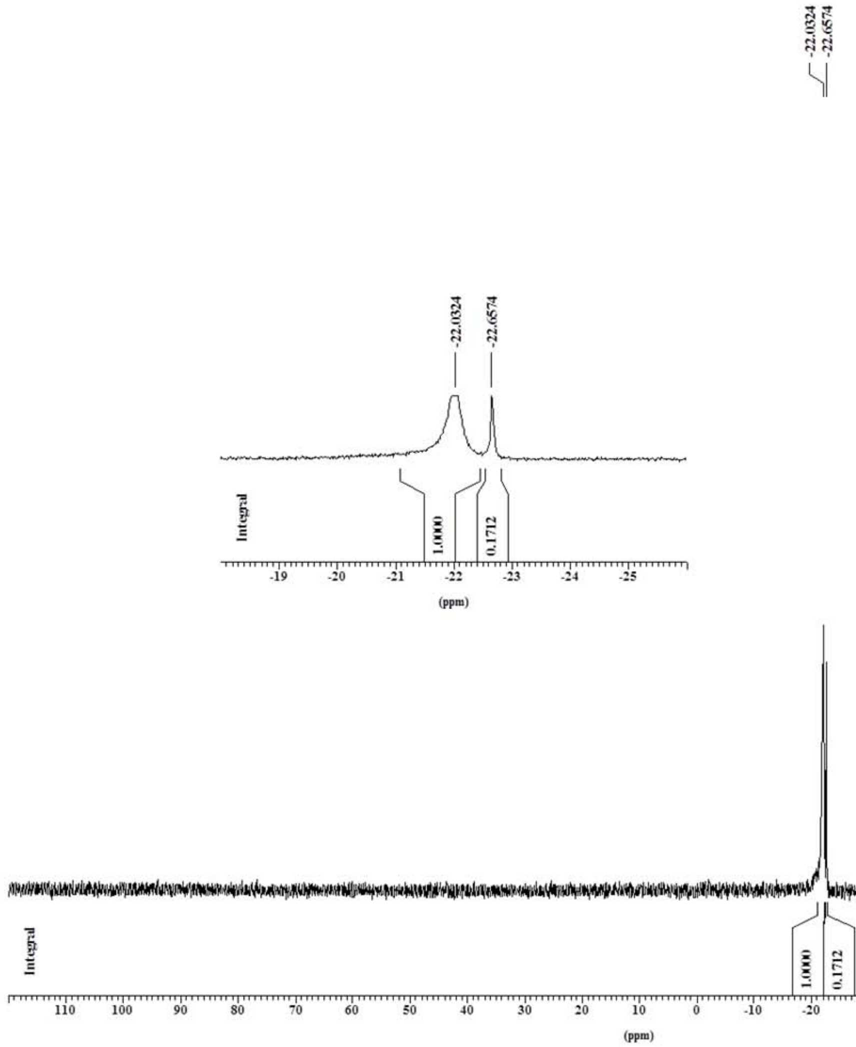

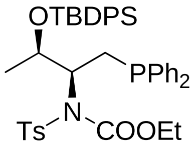

2c''
(dr = 85/15)

*** Current Data Parameters ***		
NAME	:	wtl-0507
EXPNO	:	7
PROCNO	:	1
*** Acquisition Parameters ***		
LOCNUC	:	2H
NS	:	230
NUCLEUS	:	off
O1	:	-10122.85 Hz
PULPROG	:	zgpg
SFO1	:	202.4462121 MHz
SOLVENT	:	CDCl3
SW	:	401.5922 ppm
TD	:	65536
TE	:	296.3 K
*** Processing Parameters ***		
LB	:	1.00 Hz
SF	:	202.4562131 MHz
*** 1D NMR Plot Parameters ***		
NUCLEUS	:	off

1H AMX500

wtl-876

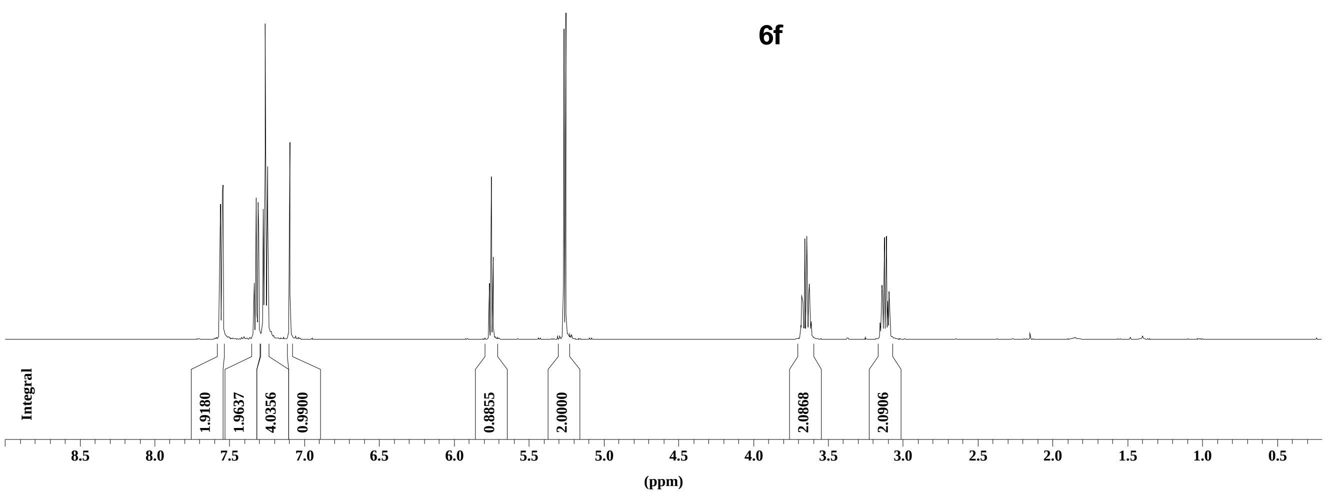

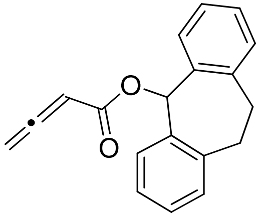

6f

*** Current Data Parameters ***

NAME	:	wtl-0418
EXPNO	:	3
PROCNO	:	1

*** Acquisition Parameters ***

LOCNUC	:	2H
NS	:	15
NUCLEUS	:	off
O1	:	3088.51 Hz
PULPROG	:	zg30
SFO1	:	500.1330885 MHz
SOLVENT	:	CDCl3
SW	:	20.6557 ppm
TD	:	32768
TE	:	294.7 K

*** Processing Parameters ***

LB	:	0.30 Hz
SF	:	500.1300096 MHz

*** 1D NMR Plot Parameters ***

NUCLEUS	:	off

13C AMX500

wtl-876

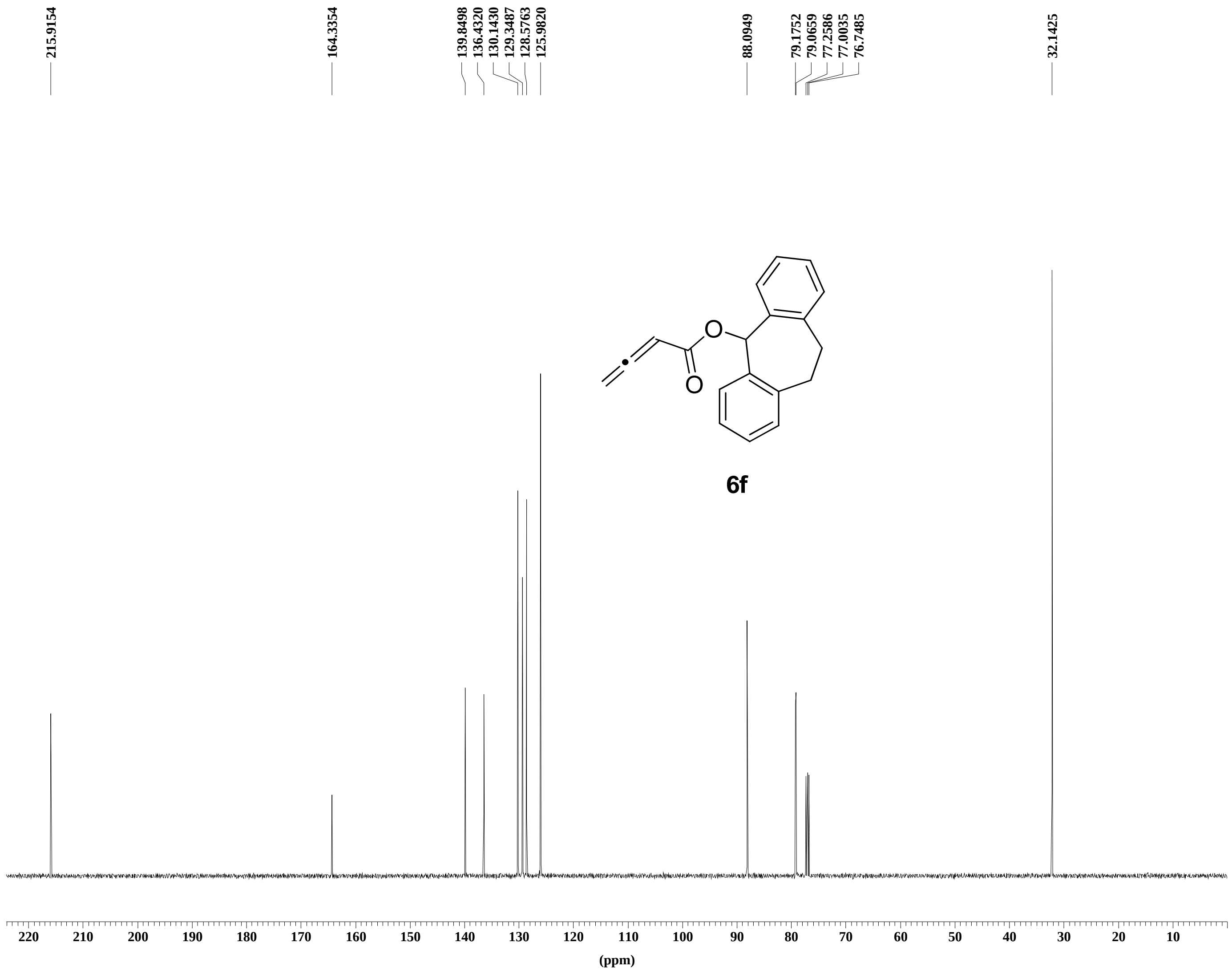

6f

*** Current Data Parameters ***

NAME	:	wtl-0418
EXPNO	:	4
PROCNO	:	1

*** Acquisition Parameters ***

LOCNUC	:	2H
NS	:	43
NUCLEUS	:	off
O1	:	13204.57 Hz
PULPROG	:	zgpg30
SFO1	:	125.7709936 MHz
SOLVENT	:	CDCl3
SW	:	238.7675 ppm
TD	:	65536
TE	:	295.2 K

*** Processing Parameters ***

LB	:	1.00 Hz
SF	:	125.7578264 MHz

*** 1D NMR Plot Parameters ***

NUCLEUS	:	off

1H AMX500

wtl-874

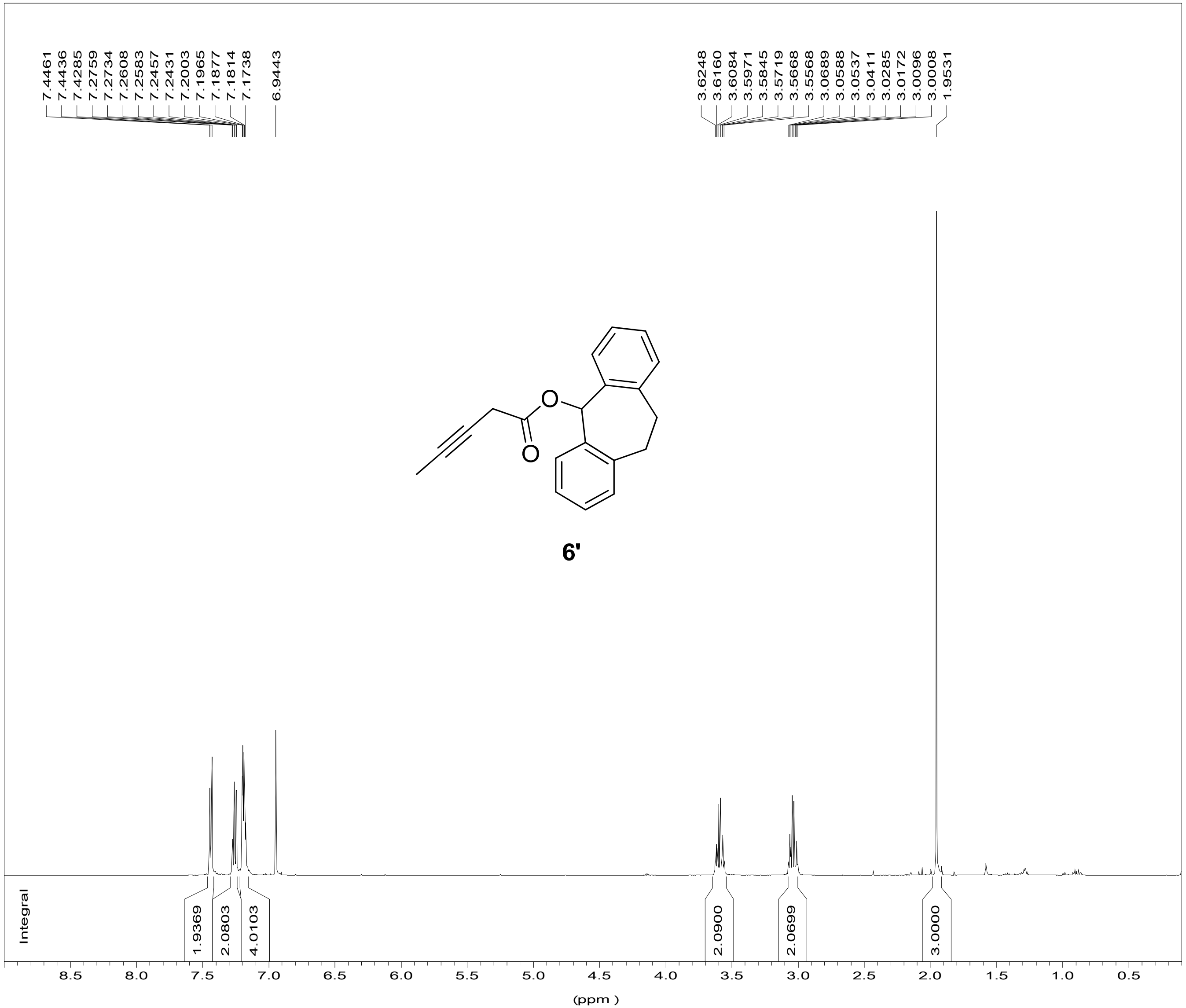

*** Current Data Parameters ***

NAME	:	wtl-0318
EXPNO	:	1
PROCNO	:	1

*** Acquisition Parameters ***

DS	:	0
INSTRUM	:	spect
LOCNUC	:	2H
NS	:	24
NUCLEUS	:	off
O1	:	3088.51 Hz
PULPROG	:	zg30
SFO1	:	500.1330885 MHz
SOLVENT	:	CDCl3
SW	:	20.6557 ppm
TD	:	32768
TE	:	298.6 K

*** Processing Parameters ***

LB	:	0.30 Hz
OFFSET	:	16.479 ppm
SI	:	16384

*** 1D NMR Plot Parameters ***

NUCLEUS	:	off

13C AMX500

wtl-874

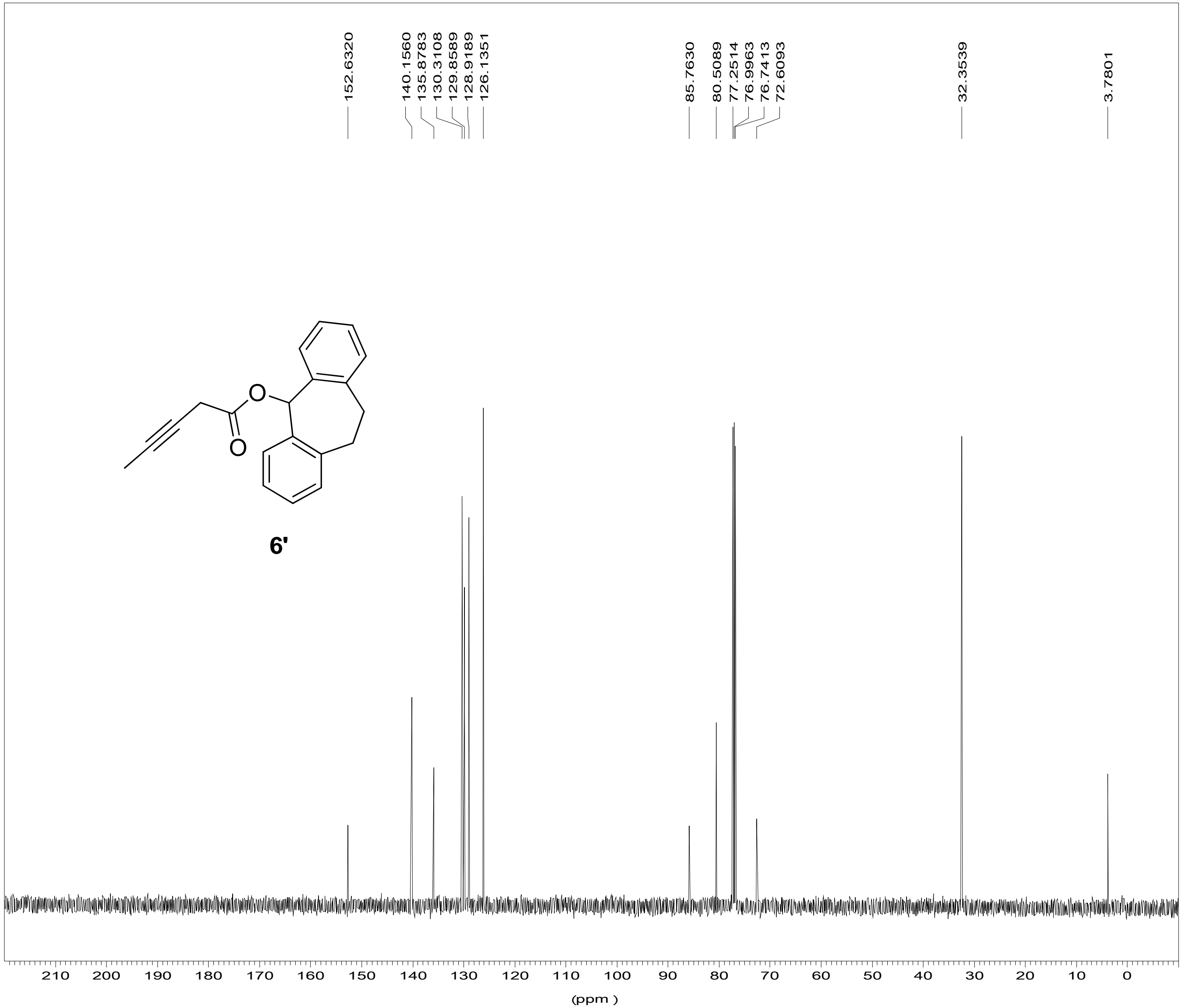

6'

*** Current Data Parameters ***		
NAME	:	wtl-0318
EXPNO	:	2
PROCNO	:	1
*** Acquisition Parameters ***		
DS	:	0
INSTRUM	:	spect
LOCNUC	:	2H
NS	:	70
NUCLEUS	:	off
O1	:	13204.57 Hz
PULPROG	:	zgpg30
SFO1	:	125.7709936 MHz
SOLVENT	:	CDCl3
SW	:	238.7675 ppm
TD	:	65536
TE	:	298.6 K
*** Processing Parameters ***		
LB	:	1.00 Hz
OFFSET	:	224.340 ppm
SI	:	32768
*** 1D NMR Plot Parameters ***		
NUCLEUS	:	off

1H normal range AC300

wtl-959-2

*** Current Data Parameters ***

NAME	:	may24wtl
EXPNO	:	1
PROCNO	:	1

*** Acquisition Parameters ***

LOCNUC	:	2H
NS	:	26
NUCLEUS	:	off
O1	:	1853.43 Hz
PULPROG	:	zg30
SFO1	:	300.1318534 MHz
SOLVENT	:	DMSO
SW	:	17.9519 ppm
TD	:	32768
TE	:	296.7 K

*** Processing Parameters ***

LB	:	0.30 Hz
SF	:	300.1300000 MHz

*** 1D NMR Plot Parameters ***

NUCLEUS	:	off

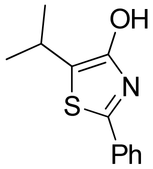

5d

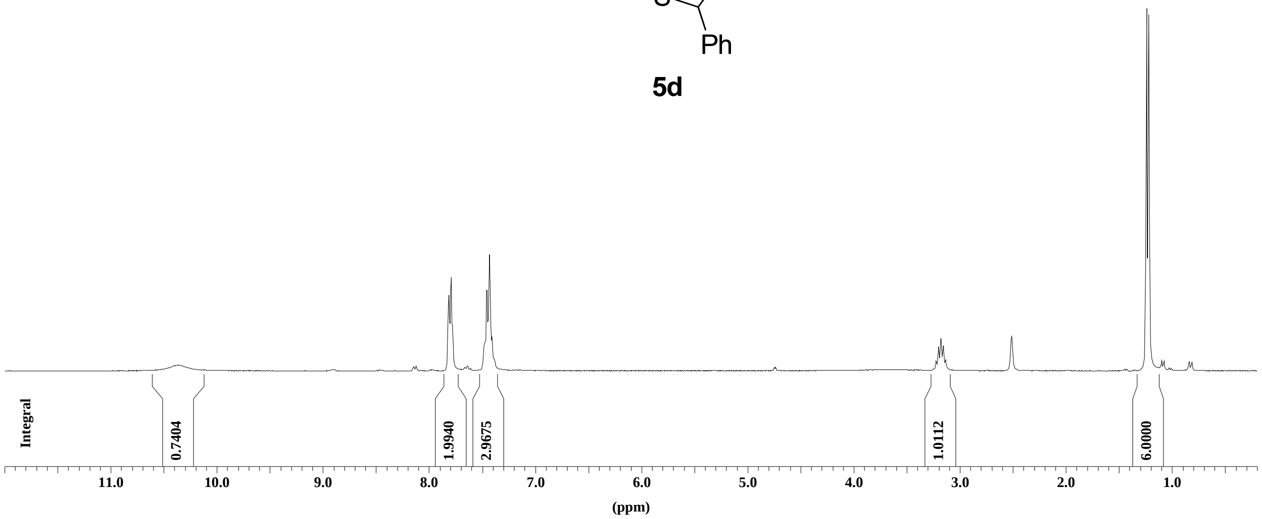

13C Standard AC300

wtl-959-2

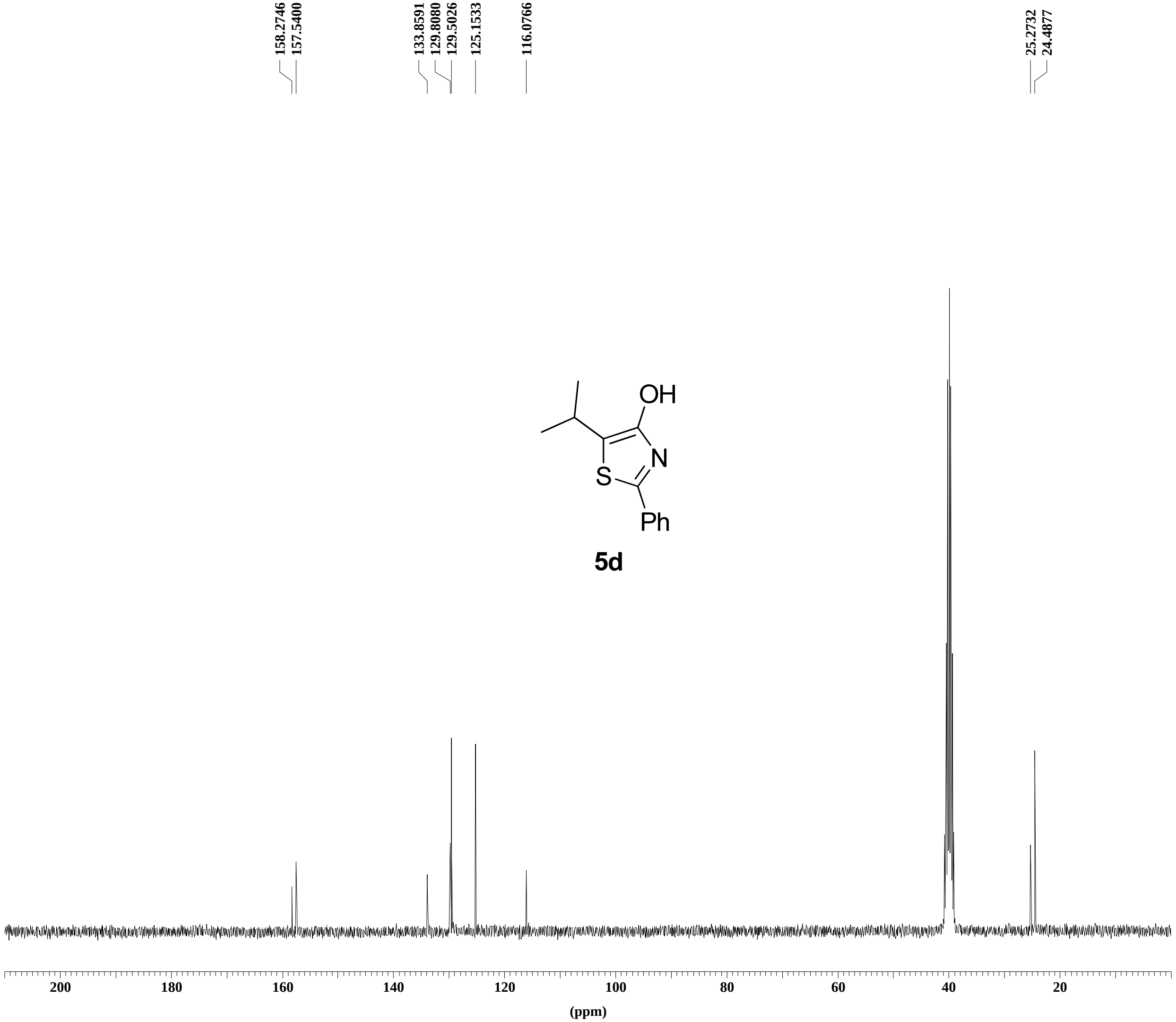

5d

*** Current Data Parameters ***
NAME : may24wtl
EXPNO : 2
PROCNO : 1
*** Acquisition Parameters ***
LOCNUC : 2H
NS : 521
NUCLEUS : off
O1 : 7924.11 Hz
PULPROG : zgpg30
SFO1 : 75.4756731 MHz
SOLVENT : DMSO
SW : 238.2968 ppm
TD : 32768
TE : 296.7 K
*** Processing Parameters ***
LB : 1.00 Hz
SF : 75.4677567 MHz
*** 1D NMR Plot Parameters ***
NUCLEUS : off

1H normal range AC300

wtl-969-2

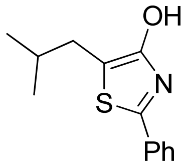

5f

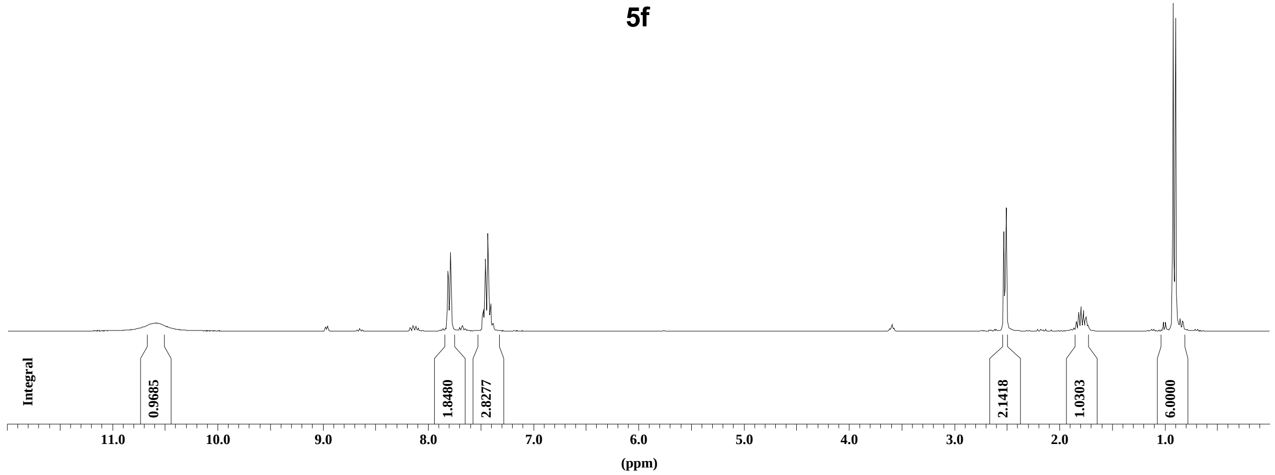

*** Current Data Parameters ***

NAME	:	may29wtl
EXPNO	:	1
PROCNO	:	1

*** Acquisition Parameters ***

LOCNUC	:	2H
NS	:	19
NUCLEUS	:	off
O1	:	1853.43 Hz
PULPROG	:	zg30
SFO1	:	300.1318534 MHz
SOLVENT	:	DMSO
SW	:	17.9519 ppm
TD	:	32768
TE	:	296.7 K

*** Processing Parameters ***

LB	:	0.30 Hz
SF	:	300.1300000 MHz

*** 1D NMR Plot Parameters ***

NUCLEUS	:	off

13C Standard AC300

wtl-969-2

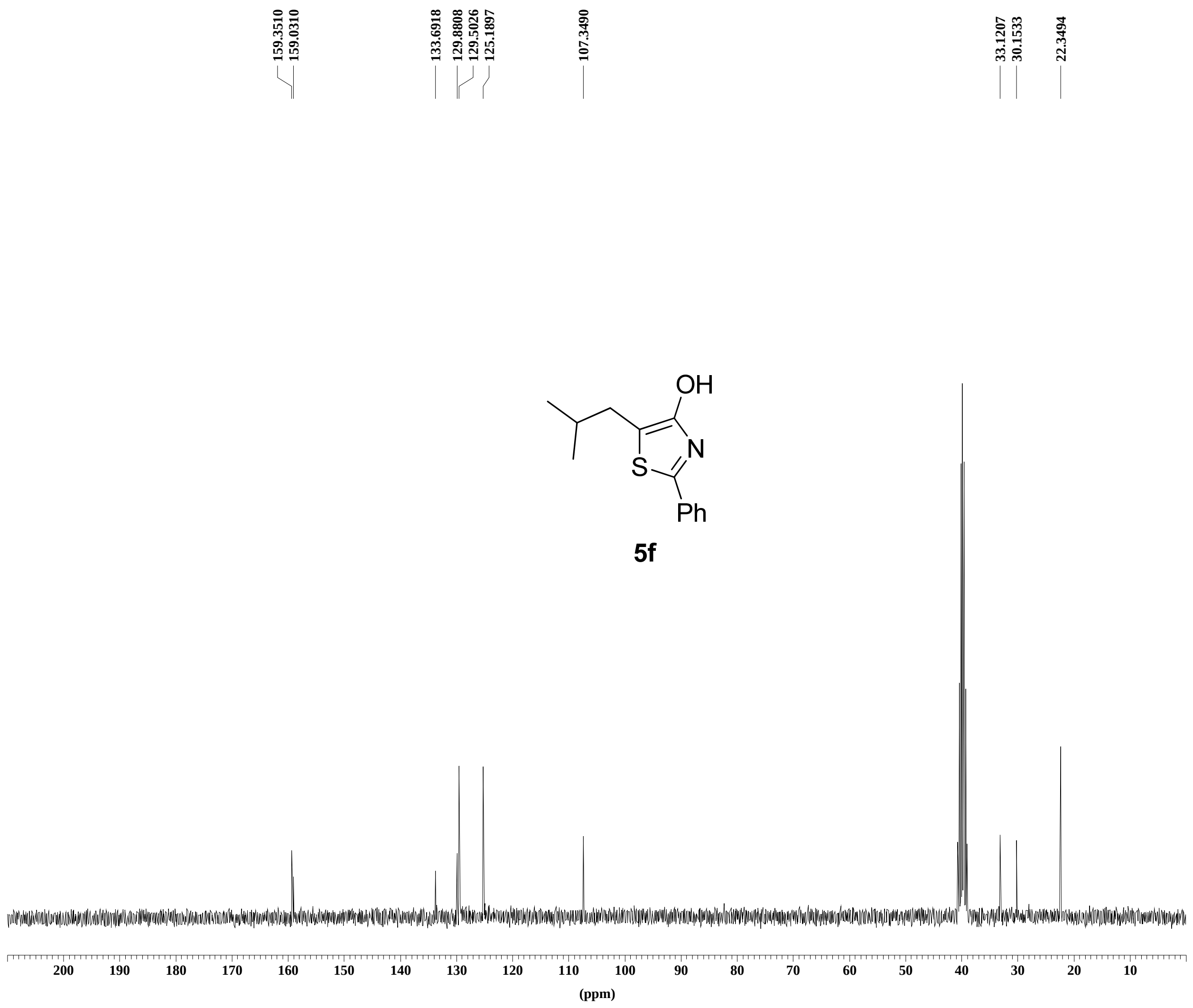

*** Current Data Parameters ***		
NAME	:	may29wtl
EXPNO	:	2
PROCNO	:	1
*** Acquisition Parameters ***		
LOCNUC	:	2H
NS	:	217
NUCLEUS	:	off
O1	:	7924.11 Hz
PULPROG	:	zgpg30
SFO1	:	75.4756731 MHz
SOLVENT	:	DMSO
SW	:	238.2968 ppm
TD	:	32768
TE	:	296.7 K
*** Processing Parameters ***		
LB	:	1.00 Hz
SF	:	75.4677567 MHz
*** 1D NMR Plot Parameters ***		
NUCLEUS	:	off

1H AMX500

wtl-955-2

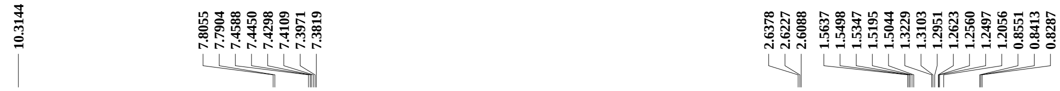

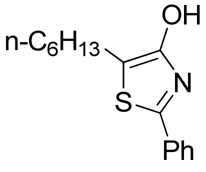

5g

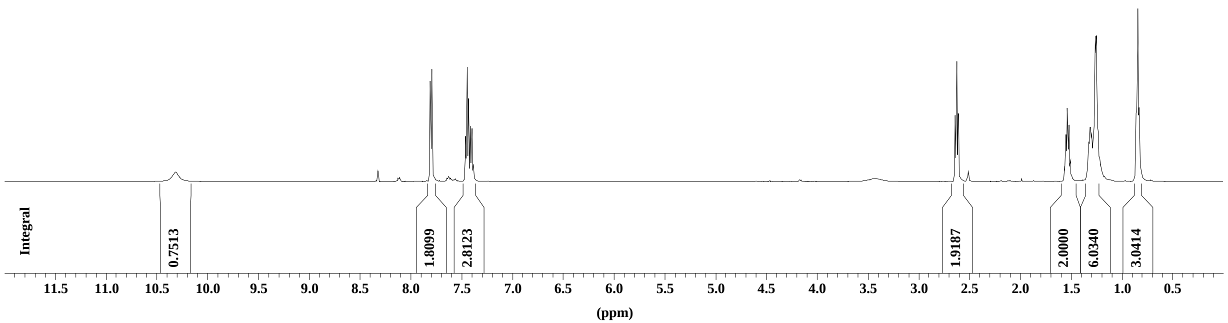

*** Current Data Parameters ***		
NAME	:	wtl-0522
EXPNO	:	3
PROCNO	:	1
*** Acquisition Parameters ***		
LOCNUC	:	2H
NS	:	17
NUCLEUS	:	off
O1	:	3088.51 Hz
PULPROG	:	zg30
SFO1	:	500.1330885 MHz
SOLVENT	:	DMSO
SW	:	20.6557 ppm
TD	:	32768
TE	:	298.0 K
*** Processing Parameters ***		
LB	:	0.30 Hz
SF	:	500.1300000 MHz
*** 1D NMR Plot Parameters ***		
NUCLEUS	:	off

13C AMX500

wtl-955-2

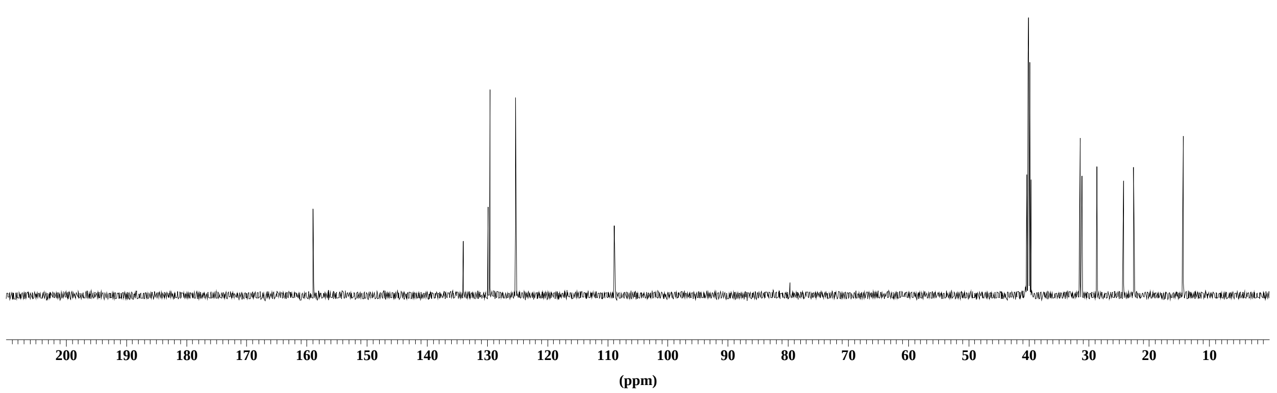

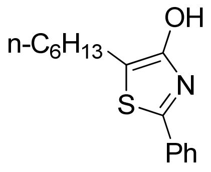

5g

***** Current Data Parameters *****

NAME	:	wtl-0522	
EXPNO	:	4	
PROCNO	:	1	

***** Acquisition Parameters *****

LOCNUC	:	2H	
NS	:	25	
NUCLEUS	:	off	
O1	:	13204.57	Hz
PULPROG	:	zgpg30	
SFO1	:	125.7709936	MHz
SOLVENT	:	DMSO	
SW	:	238.7675	ppm
TD	:	65536	
TE	:	298.1	K

***** Processing Parameters *****

LB	:	1.00	Hz
SF	:	125.7577890	MHz

***** 1D NMR Plot Parameters *****

NUCLEUS	:	off	

1H AMX500

wtl-971-3

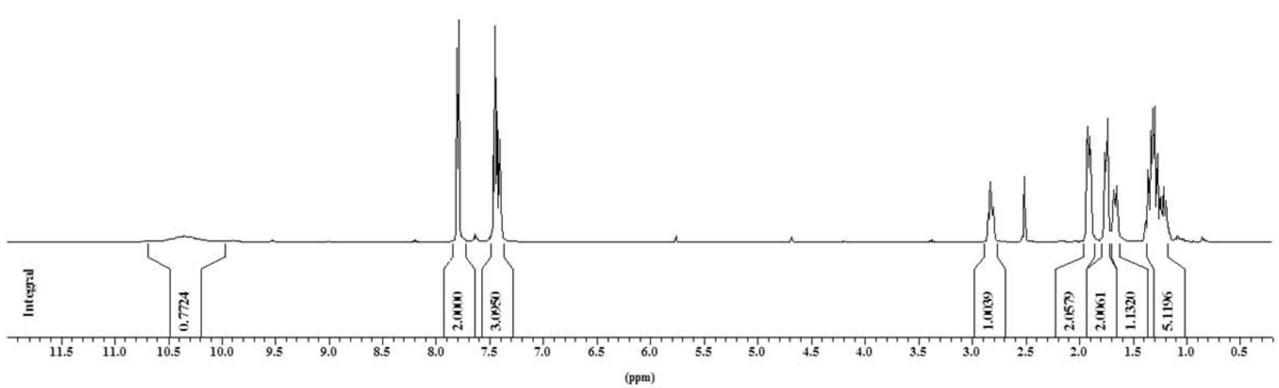

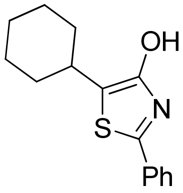

5h

*** Current Data Parameters ***

NAME	:	wtl-0603
EXPNO	:	1
PROCNO	:	1

*** Acquisition Parameters ***

LOCNUC	:	2H
NS	:	29
NUCLEUS	:	off
O1	:	3088.51 Hz
PULPROG	:	zg30
SFO1	:	500.1330885 MHz
SOLVENT	:	DMSO
SW	:	20.6557 ppm
TD	:	32768
TE	:	295.4 K

*** Processing Parameters ***

LB	:	0.30 Hz
SF	:	500.1300000 MHz

*** 1D NMR Plot Parameters ***

NUCLEUS	:	off

13C AMX500

wtl-971-3

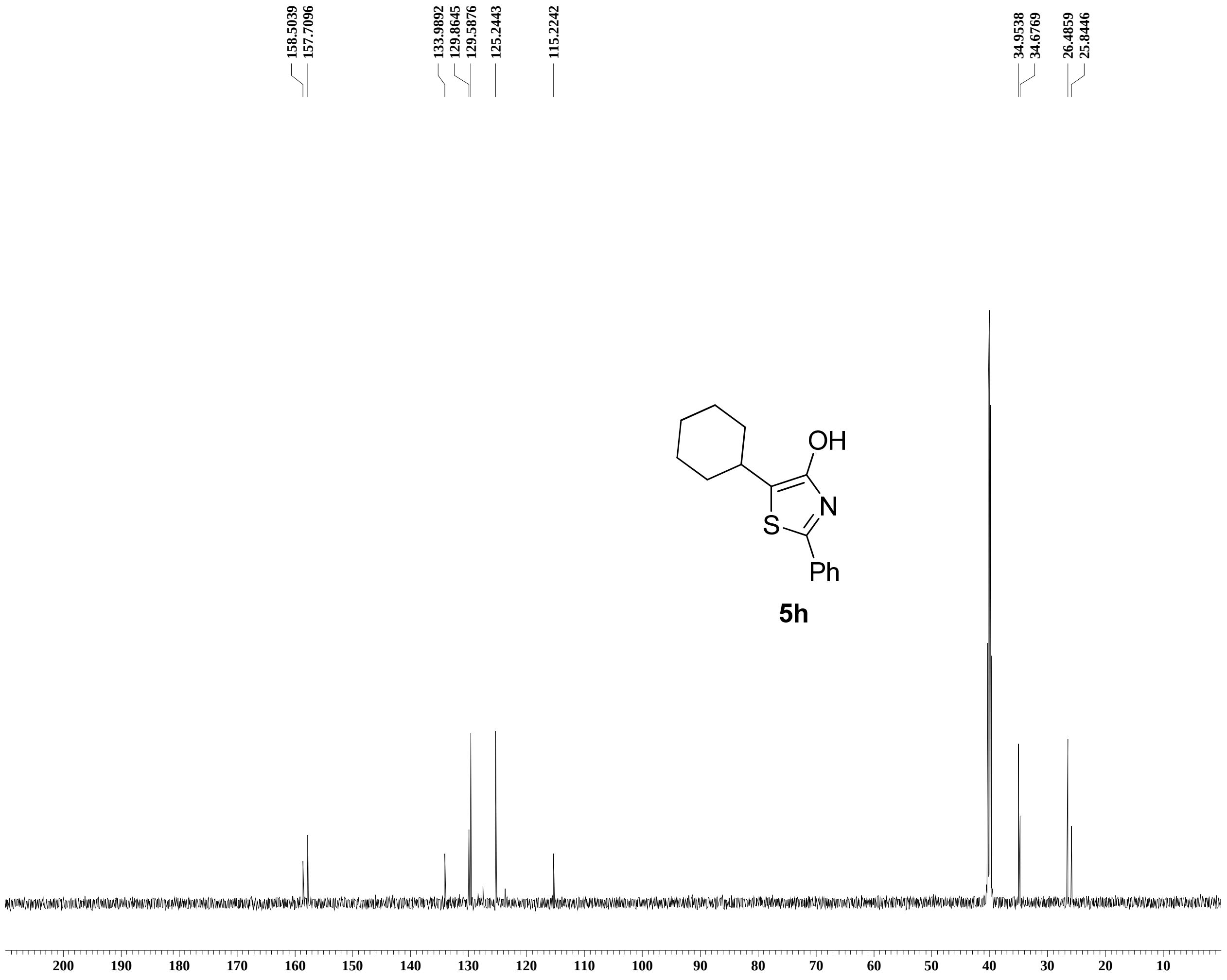

5h

*** Current Data Parameters ***

NAME	:	wtl-0603
EXPNO	:	2
PROCNO	:	1

*** Acquisition Parameters ***

LOCNUC	:	2H
NS	:	30
NUCLEUS	:	off
O1	:	13204.57 Hz
PULPROG	:	zgpg30
SFO1	:	125.7709936 MHz
SOLVENT	:	DMSO
SW	:	238.7675 ppm
TD	:	65536
TE	:	295.5 K

*** Processing Parameters ***

LB	:	1.00 Hz
SF	:	125.7577890 MHz

*** 1D NMR Plot Parameters ***

NUCLEUS	:	off

1H AMX500

wtl-966-2

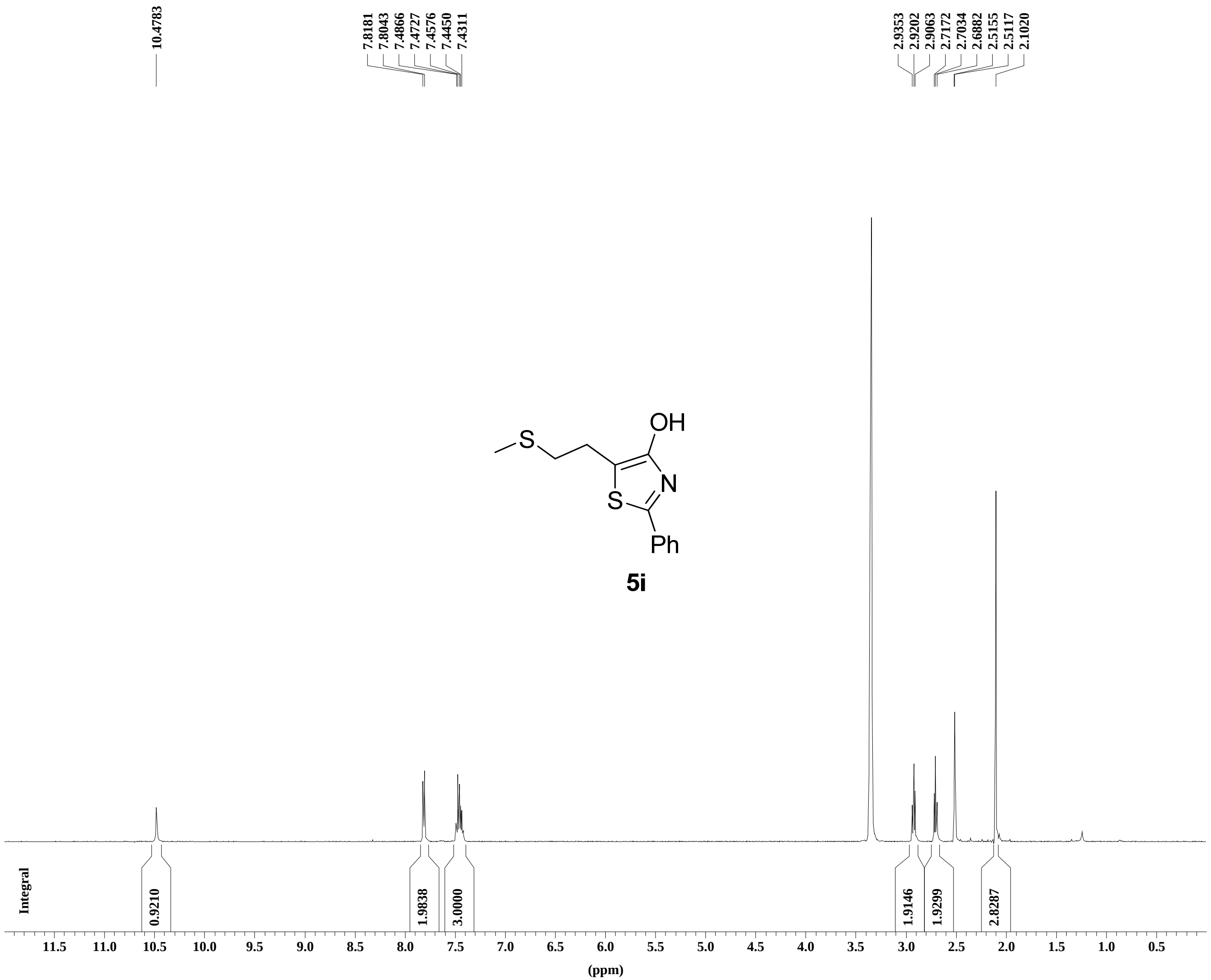

***** Current Data Parameters *****

NAME	:	wtl-0527
EXPNO	:	6
PROCNO	:	1

***** Acquisition Parameters *****

LOCNUC	:	2H
NS	:	1
NUCLEUS	:	off
O1	:	3088.51 Hz
PULPROG	:	zg30
SFO1	:	500.1330885 MHz
SOLVENT	:	DMSO
SW	:	20.6557 ppm
TD	:	32768
TE	:	298.1 K

***** Processing Parameters *****

LB	:	0.30 Hz
SF	:	500.1300000 MHz

***** 1D NMR Plot Parameters *****

NUCLEUS	:	off

13C AMX500

wtl-957-2

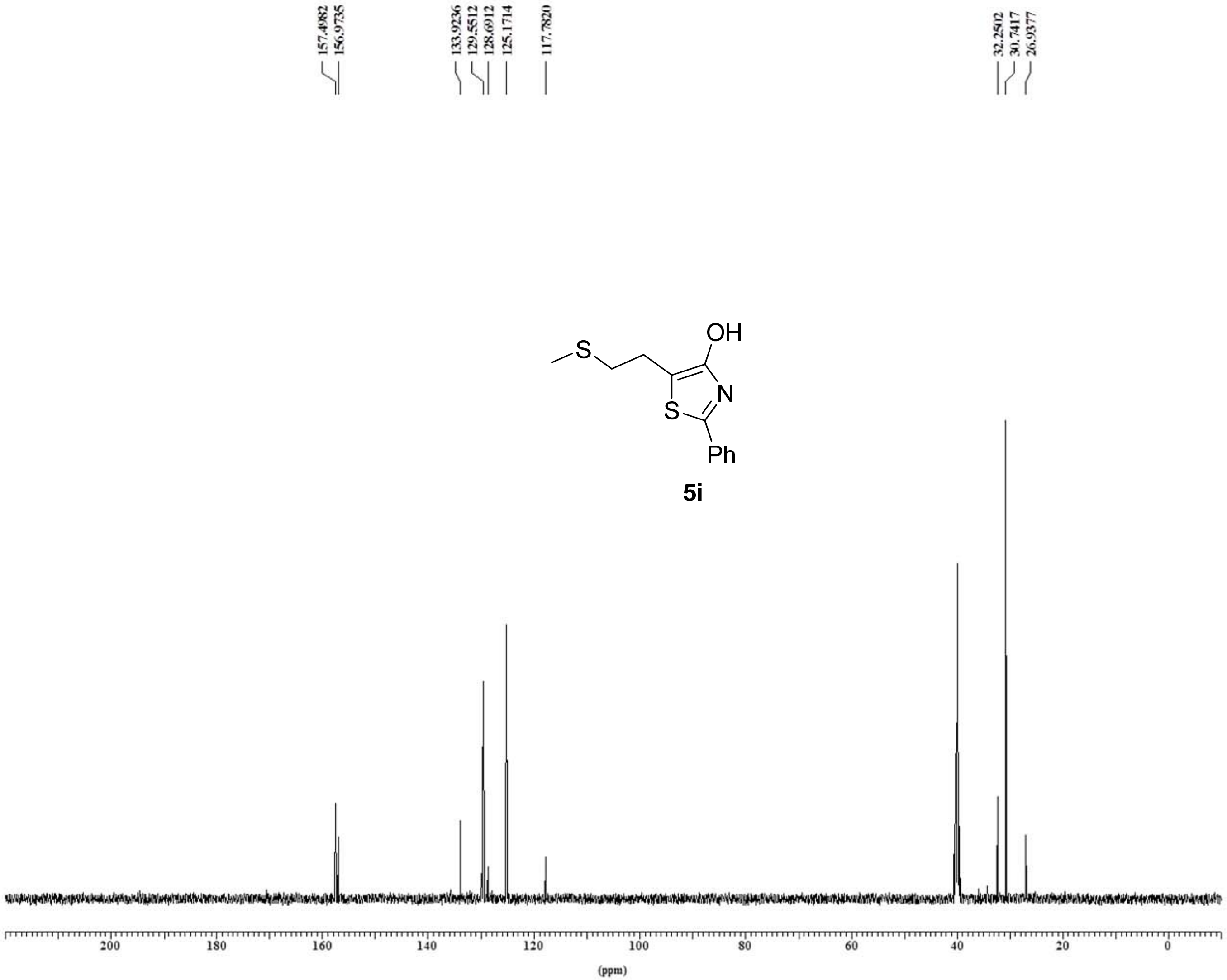

*** Current Data Parameters ***		
NAME	:	wtl-0527
EXPNO	:	11
PROCNO	:	1
*** Acquisition Parameters ***		
LOCNUC	:	2H
NS	:	30
NUCLEUS	:	off
O1	:	13204.57 Hz
PULPROG	:	zgpg30
SFO1	:	125.7709936 MHz
SOLVENT	:	DMSO
SW	:	238.7675 ppm
TD	:	65536
TE	:	298.2 K
*** Processing Parameters ***		
LB	:	1.00 Hz
SF	:	125.7577890 MHz
*** 1D NMR Plot Parameters ***		
NUCLEUS	:	off

1H AMX500

wtl-976-2

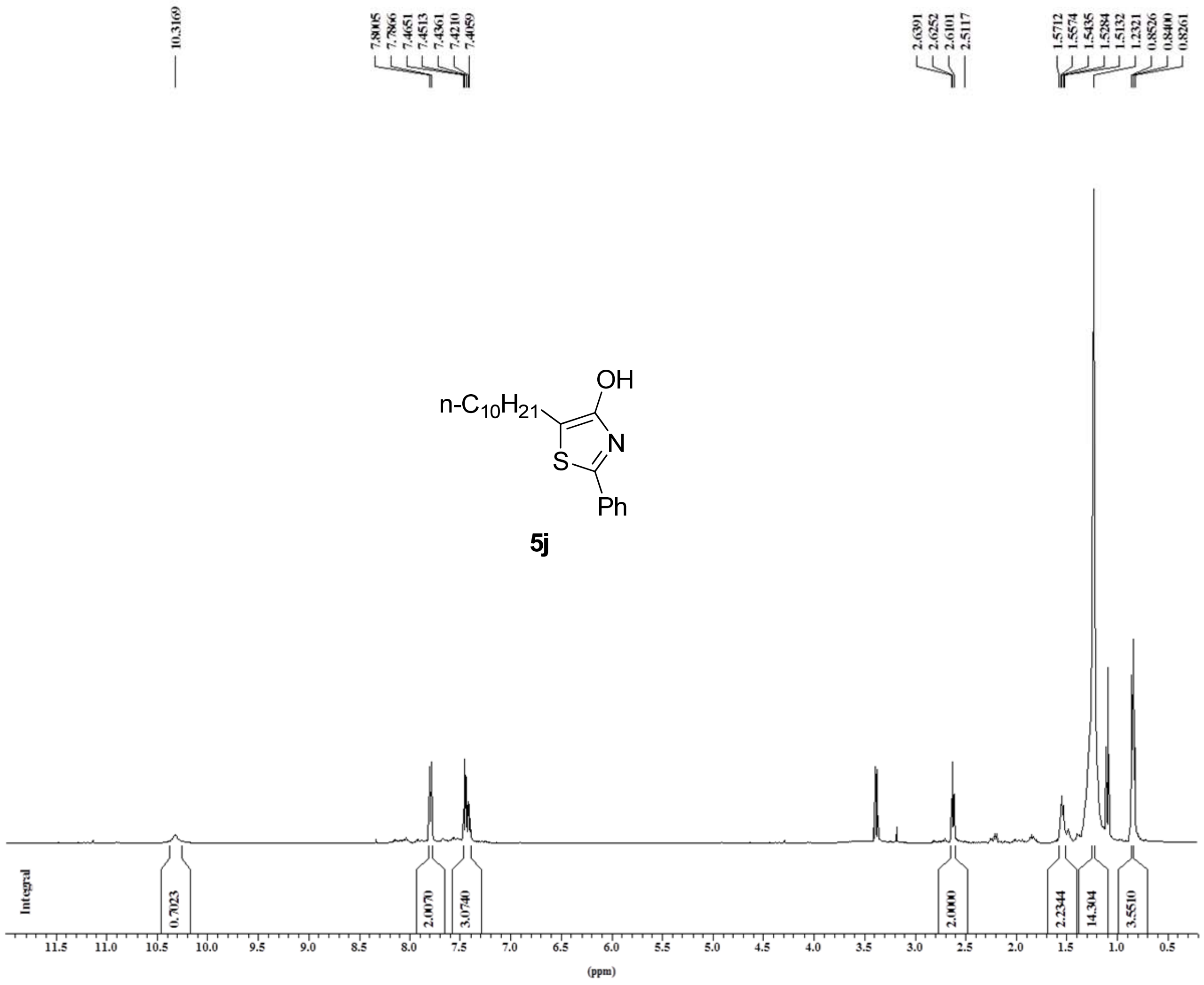

*** Current Data Parameters ***

NAME	:	wtl-0612
EXPNO	:	1
PROCNO	:	1

*** Acquisition Parameters ***

LOCNUC	:	2H
NS	:	31
NUCLEUS	:	off
O1	:	3088.51 Hz
PULPROG	:	zg30
SFO1	:	500.1330885 MHz
SOLVENT	:	DMSO
SW	:	20.6557 ppm
TD	:	32768
TE	:	295.6 K

*** Processing Parameters ***

LB	:	0.30 Hz
SF	:	500.1300000 MHz

*** 1D NMR Plot Parameters ***

NUCLEUS	:	off

13C AMX500

wtl-976-2

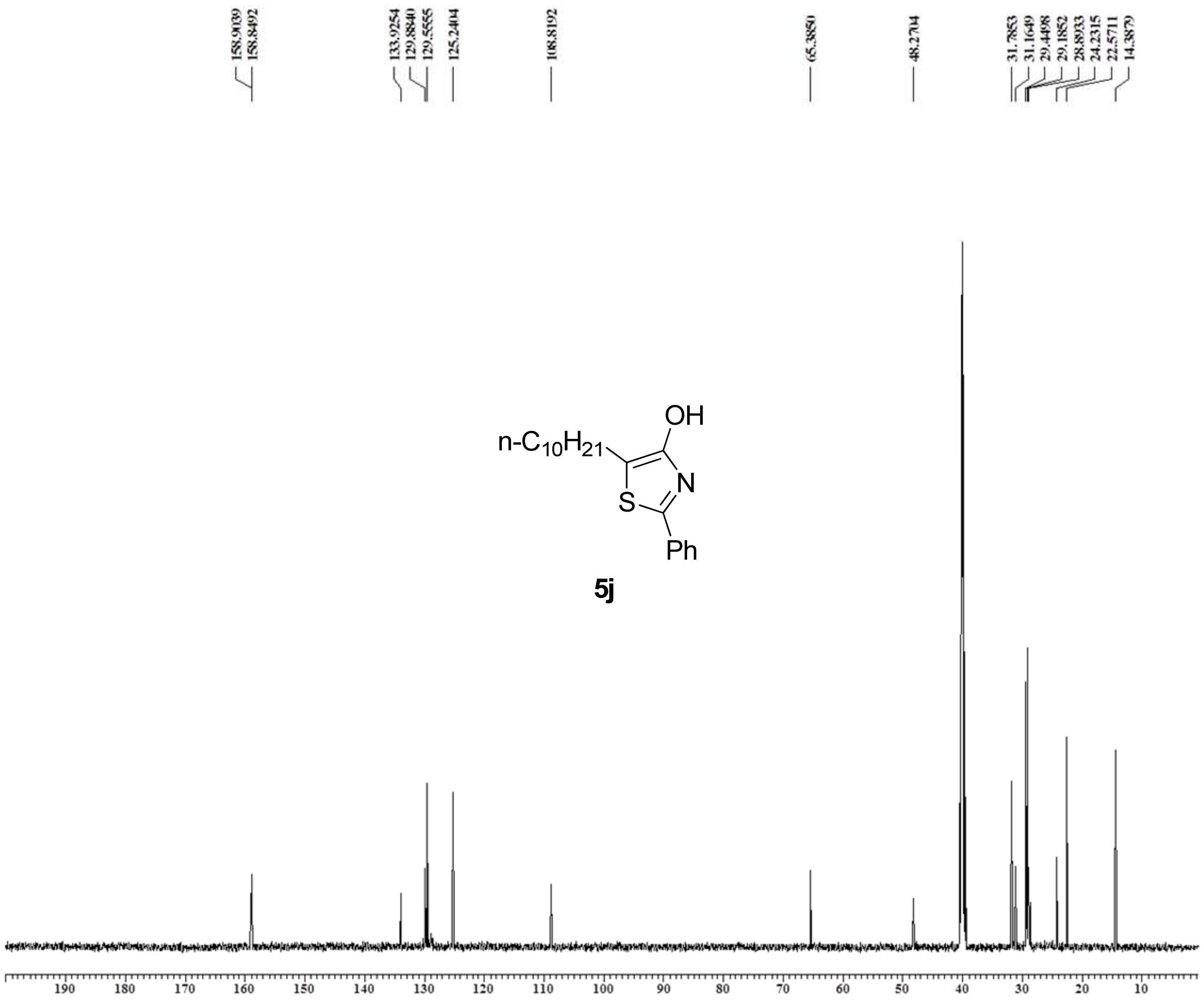

5j

*** Current Data Parameters ***

NAME	:	wtl-0612
EXPNO	:	2
PROCNO	:	1

*** Acquisition Parameters ***

LOCNUC	:	2H
NS	:	267
NUCLEUS	:	off
O1	:	18863.67 Hz
PULPROG	:	zgpg30
SFO1	:	125.7766527 MHz
SOLVENT	:	DMSO
SW	:	298.8948 ppm
TD	:	65536
TE	:	295.7 K

*** Processing Parameters ***

LB	:	1.00 Hz
SF	:	125.7577890 MHz

*** 1D NMR Plot Parameters ***

NUCLEUS	:	off

1H AMX500

wtl-924

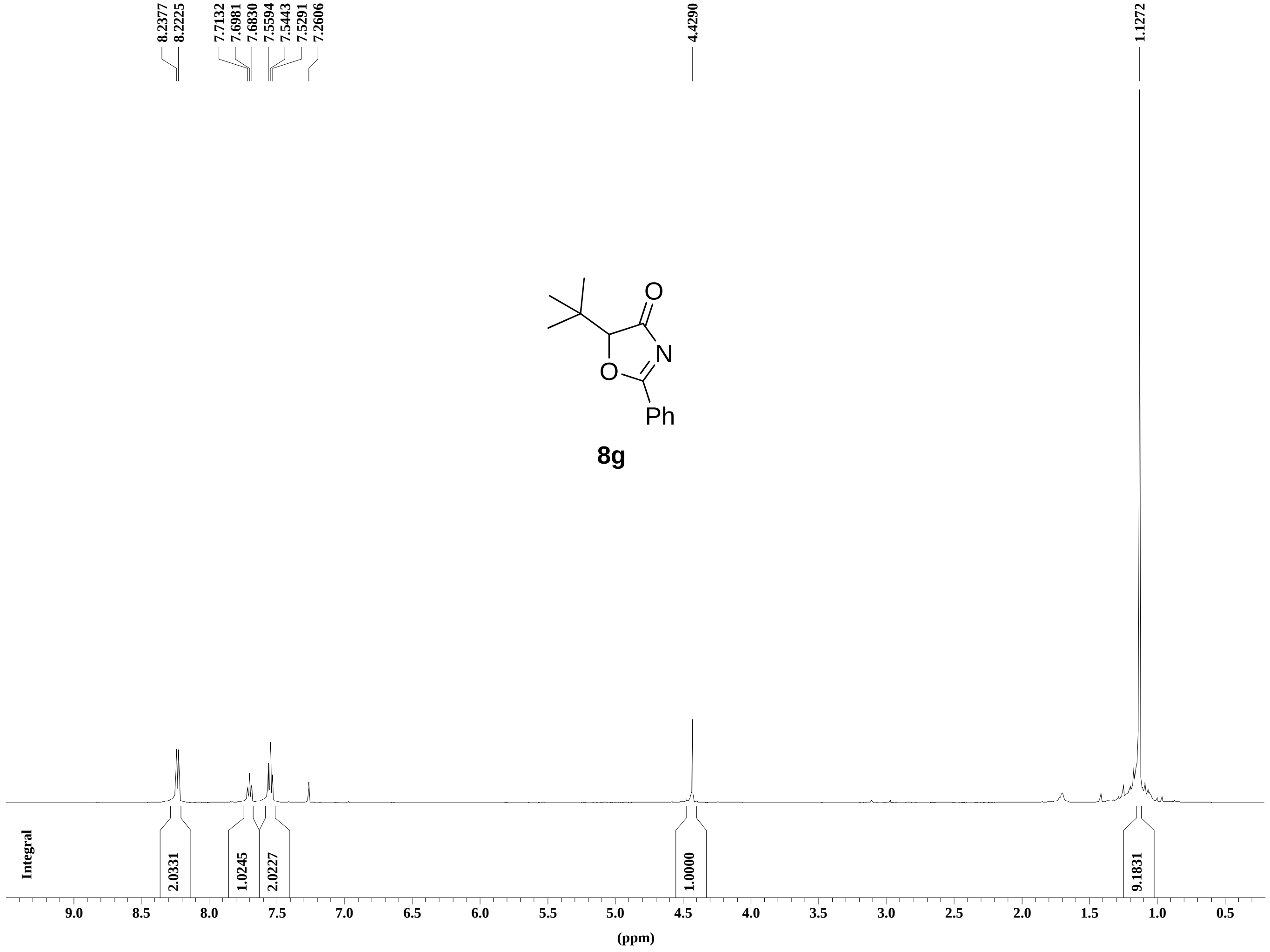

***** Current Data Parameters *****

NAME	:	wtl-0423
EXPNO	:	1
PROCNO	:	1

***** Acquisition Parameters *****

LOCNUC	:	2H
NS	:	34
NUCLEUS	:	off
O1	:	3088.51 Hz
PULPROG	:	zg30
SFO1	:	500.1330885 MHz
SOLVENT	:	CDCl3
SW	:	20.6557 ppm
TD	:	32768
TE	:	297.1 K

***** Processing Parameters *****

LB	:	0.30 Hz
SF	:	500.1300134 MHz

***** 1D NMR Plot Parameters *****

NUCLEUS	:	off

13C AMX500

wtl-924

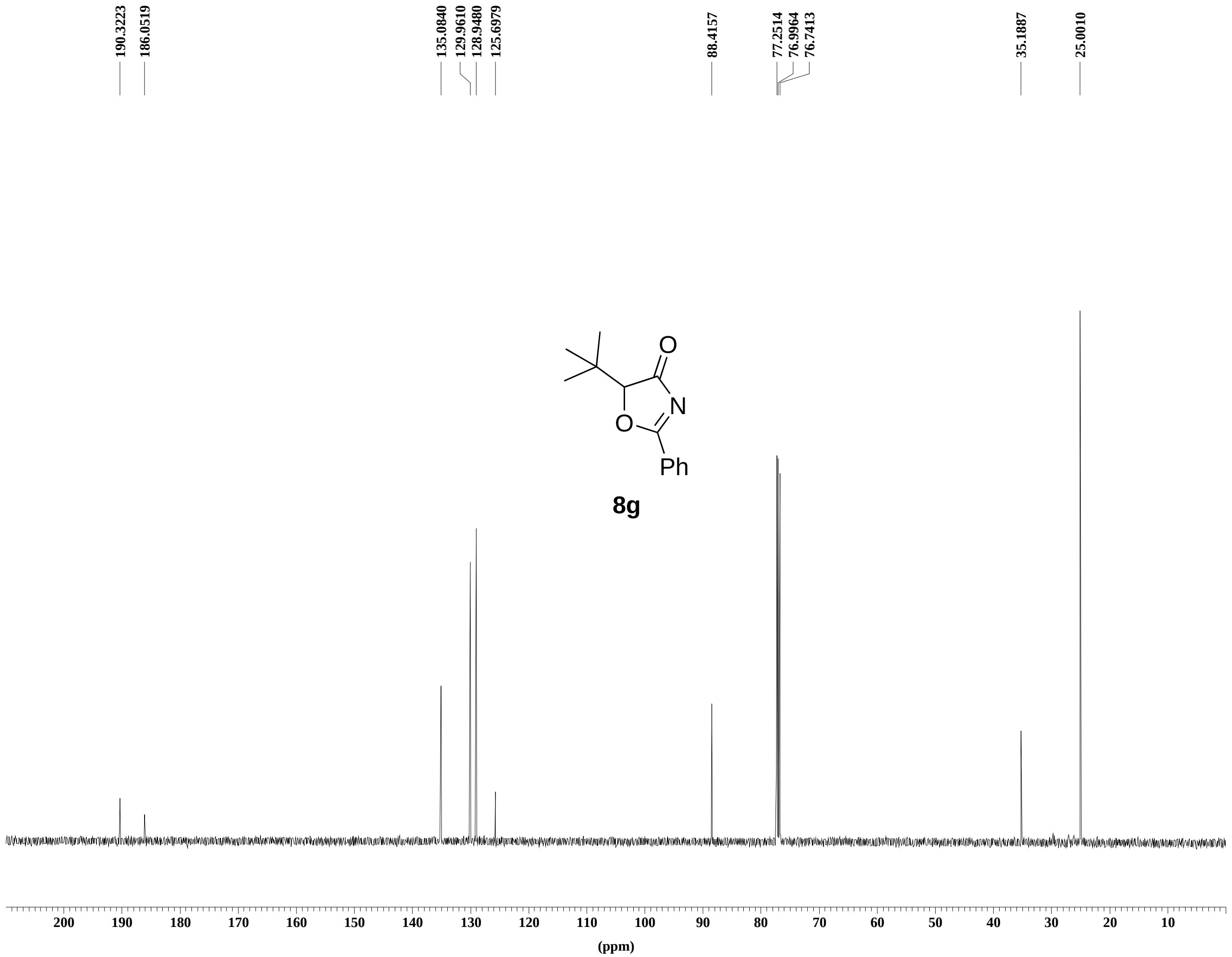

***** Current Data Parameters *****

NAME	:	wtl-0423
EXPNO	:	2
PROCNO	:	1

***** Acquisition Parameters *****

LOCNUC	:	2H
NS	:	357
NUCLEUS	:	off
O1	:	13204.57 Hz
PULPROG	:	zgpg30
SFO1	:	125.7709936 MHz
SOLVENT	:	CDCl3
SW	:	238.7675 ppm
TD	:	65536
TE	:	297.1 K

***** Processing Parameters *****

LB	:	1.00 Hz
SF	:	125.7577943 MHz

***** 1D NMR Plot Parameters *****

NUCLEUS	:	off

1H AMX500

wtl-928-3

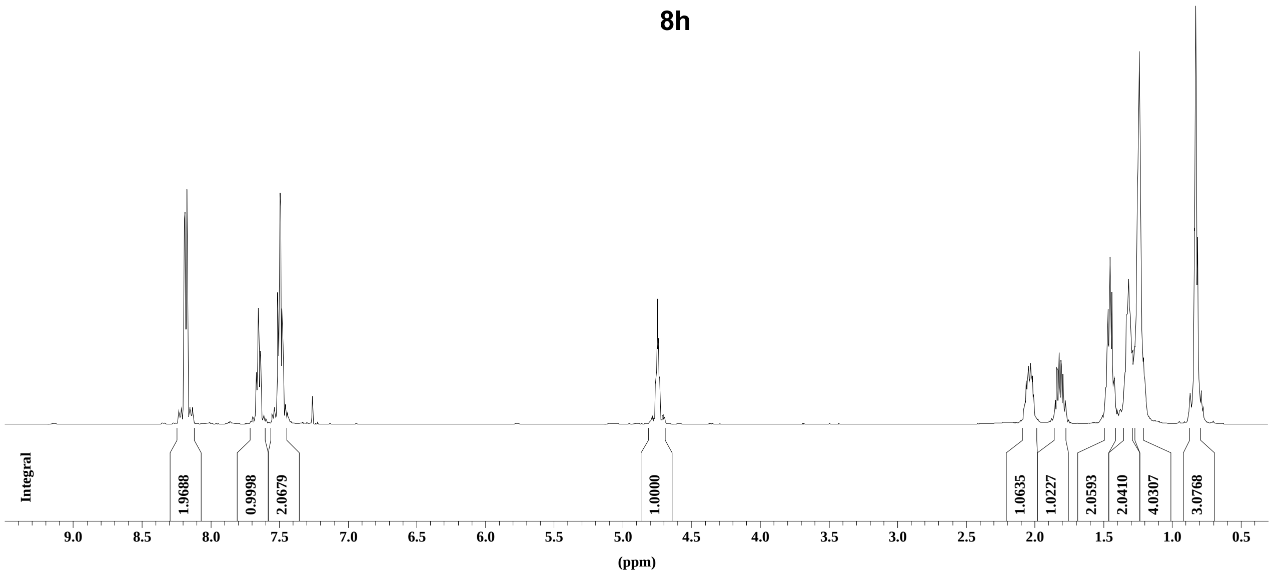

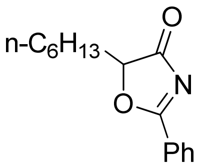

8h

*** Current Data Parameters ***

NAME	:	wtl-0503
EXPNO	:	1
PROCNO	:	1

*** Acquisition Parameters ***

LOCNUC	:	2H
NS	:	24
NUCLEUS	:	off
O1	:	3088.51 Hz
PULPROG	:	zg30
SFO1	:	500.1330885 MHz
SOLVENT	:	CDCl3
SW	:	20.6557 ppm
TD	:	32768
TE	:	295.7 K

*** Processing Parameters ***

LB	:	0.30 Hz
SF	:	500.1300134 MHz

*** 1D NMR Plot Parameters ***

NUCLEUS	:	off

13C AMX500

wtl-928-3

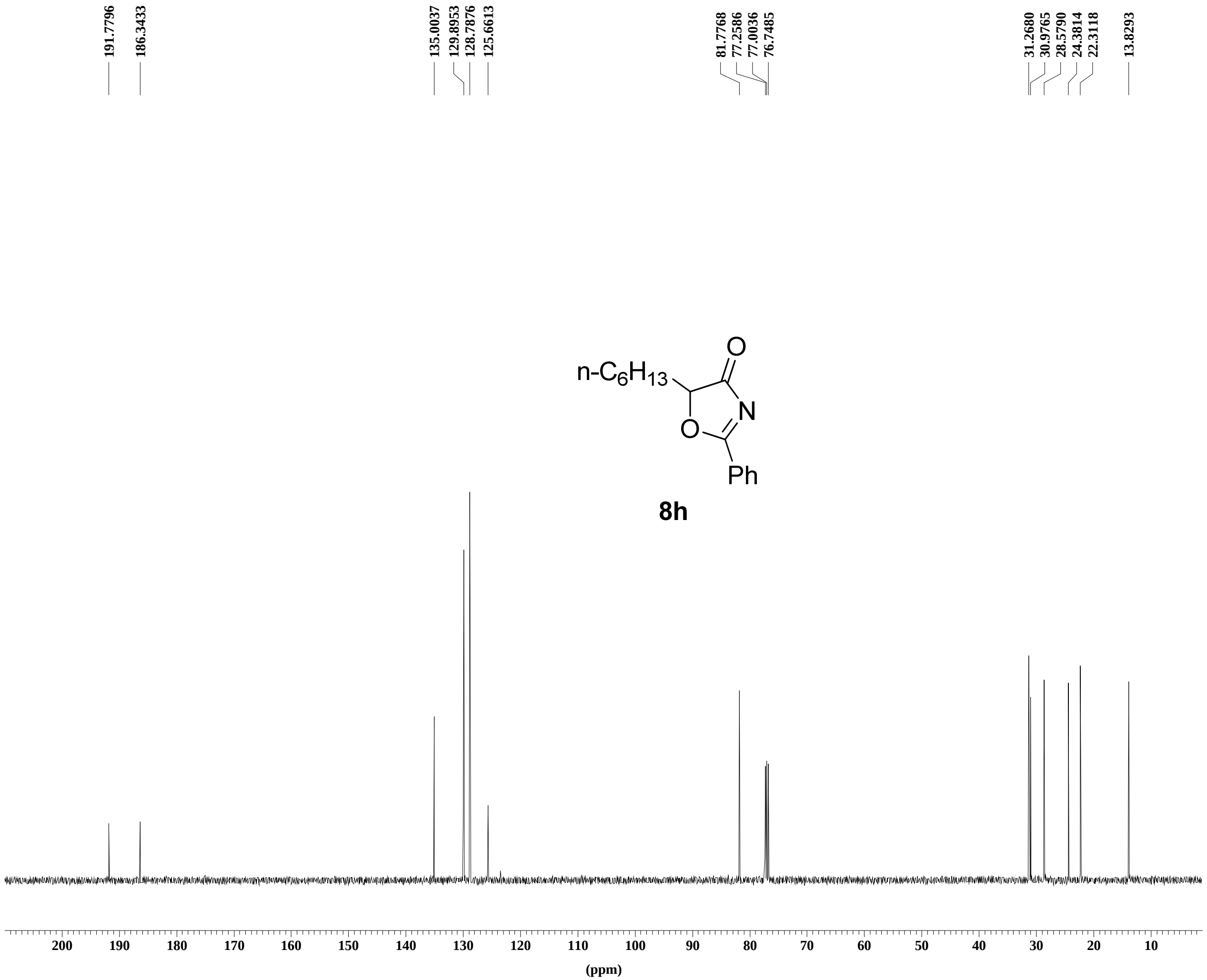

*** Current Data Parameters ***

NAME	:	wtl-0503
EXPNO	:	2
PROCNO	:	1

*** Acquisition Parameters ***

LOCNUC	:	2H
NS	:	19
NUCLEUS	:	off
O1	:	13204.57 Hz
PULPROG	:	zgpg30
SFO1	:	125.7709936 MHz
SOLVENT	:	CDCl3
SW	:	238.7675 ppm
TD	:	65536
TE	:	295.9 K

*** Processing Parameters ***

LB	:	1.00 Hz
SF	:	125.7578090 MHz

*** 1D NMR Plot Parameters ***

NUCLEUS	:	off

1H AMX500

wtl-918-3

*** Current Data Parameters ***

NAME	:	wtl-0428
EXPNO	:	2
PROCNO	:	1

*** Acquisition Parameters ***

LOCNUC	:	2H
NS	:	33
NUCLEUS	:	off
O1	:	3088.51 Hz
PULPROG	:	zg30
SFO1	:	500.1330885 MHz
SOLVENT	:	CDCl3
SW	:	20.6557 ppm
TD	:	32768
TE	:	570.6 K

*** Processing Parameters ***

LB	:	0.30 Hz
SF	:	500.1300134 MHz

*** 1D NMR Plot Parameters ***

NUCLEUS	:	off

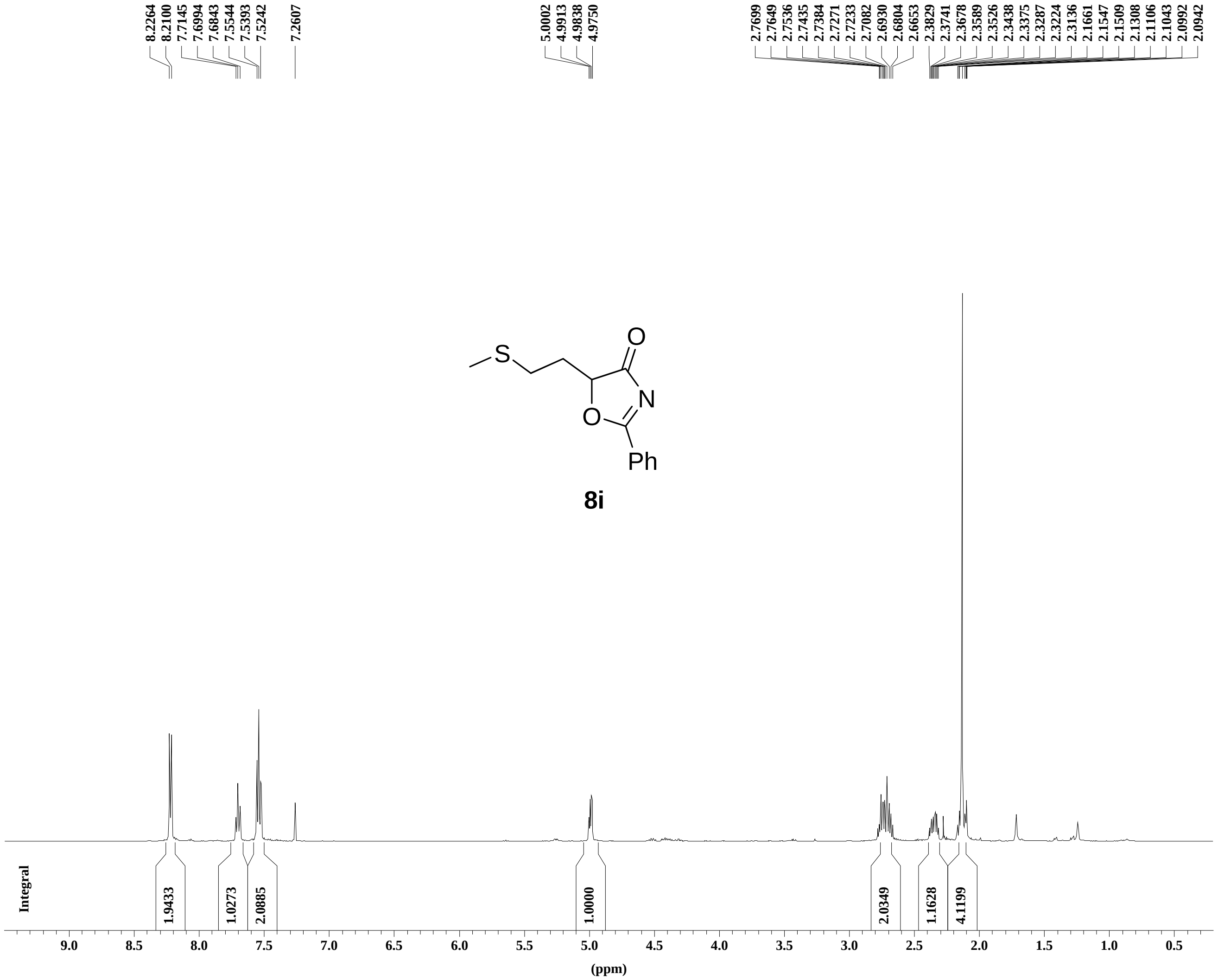

13C AMX500

wtl-918-3

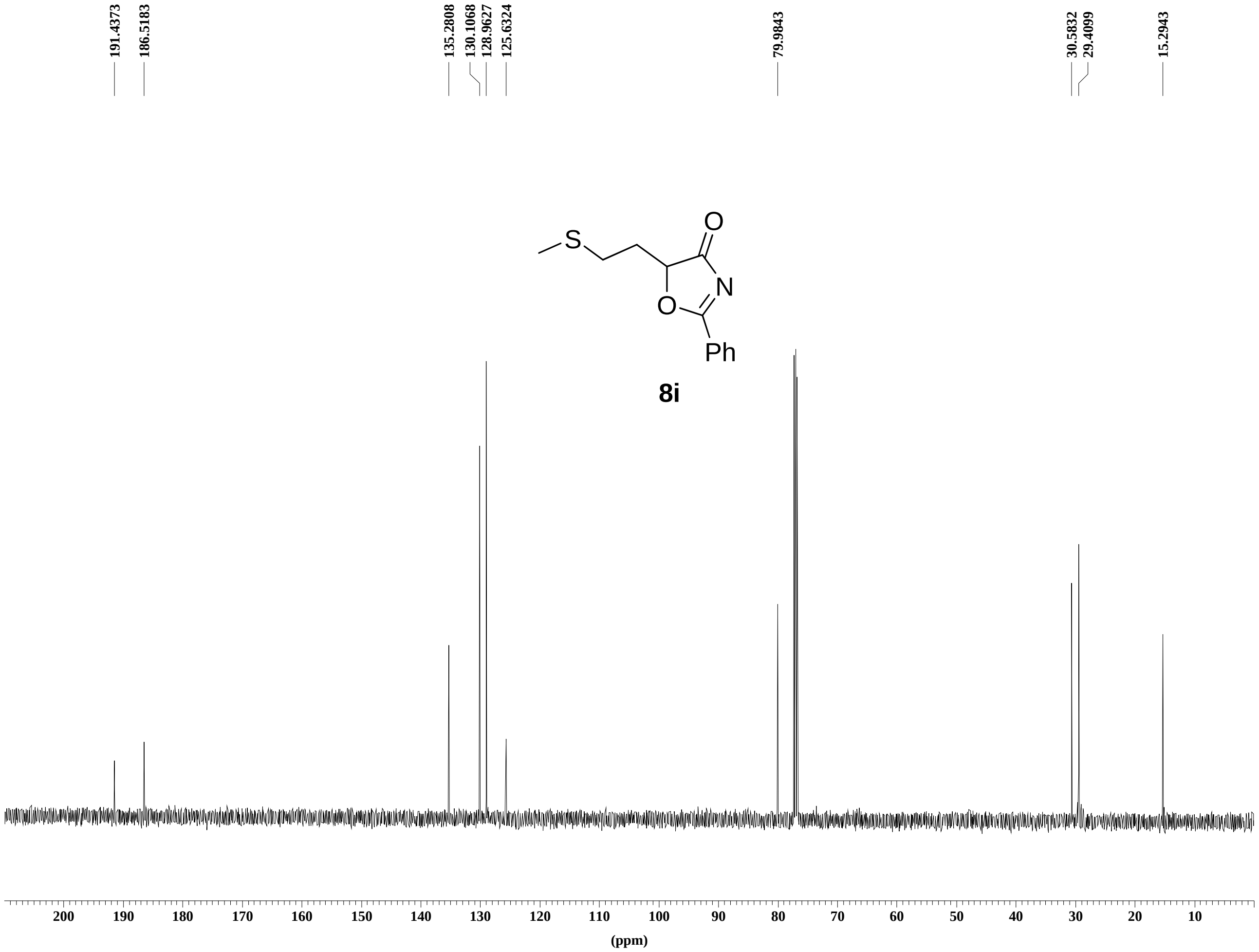

*** Current Data Parameters ***		
NAME	:	wtl-0428
EXPNO	:	1
PROCNO	:	1
*** Acquisition Parameters ***		
LOCNUC	:	2H
NS	:	70
NUCLEUS	:	off
O1	:	13204.57 Hz
PULPROG	:	zgpg30
SFO1	:	125.7709936 MHz
SOLVENT	:	CDCl3
SW	:	238.7675 ppm
TD	:	65536
TE	:	297.6 K
*** Processing Parameters ***		
LB	:	1.00 Hz
SF	:	125.7577961 MHz
*** 1D NMR Plot Parameters ***		
NUCLEUS	:	off

1H AMX500

wtl-929-3

*** Current Data Parameters ***

NAME	:	wtl-0503
EXPNO	:	3
PROCNO	:	1

*** Acquisition Parameters ***

LOCNUC	:	2H
NS	:	33
NUCLEUS	:	off
O1	:	3088.51 Hz
PULPROG	:	zg30
SFO1	:	500.1330885 MHz
SOLVENT	:	CDCl3
SW	:	20.6557 ppm
TD	:	32768
TE	:	295.7 K

*** Processing Parameters ***

LB	:	0.30 Hz
SF	:	500.1300134 MHz

*** 1D NMR Plot Parameters ***

NUCLEUS	:	off

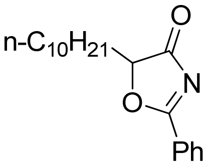

8j

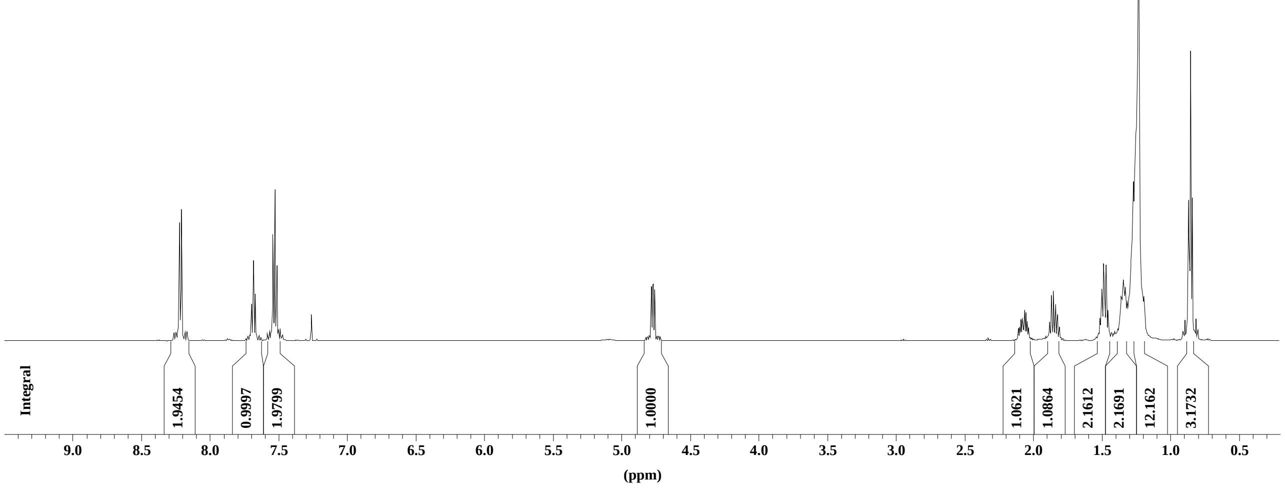

13C AMX500

wtl-929-3

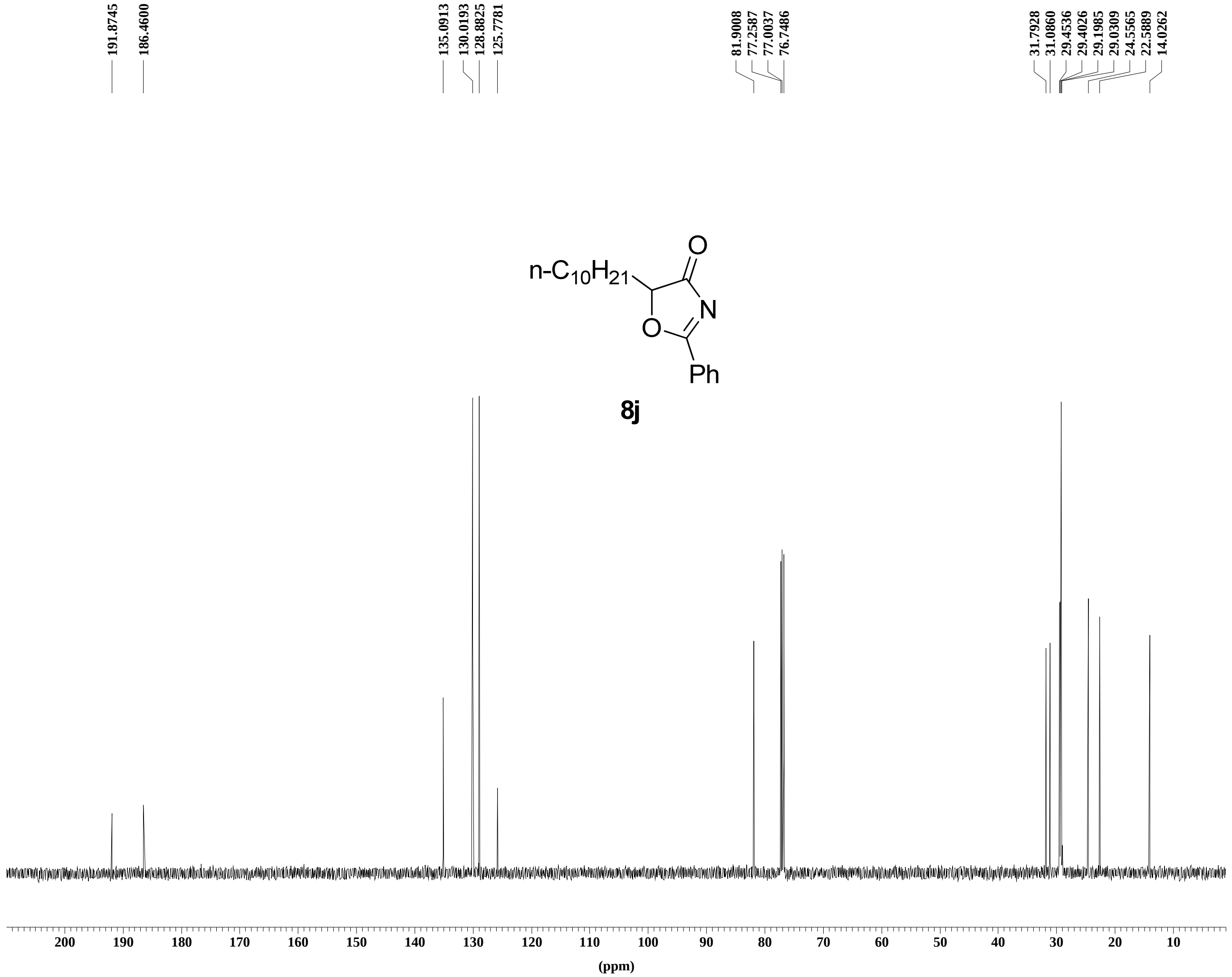

*** Current Data Parameters ***

NAME	:	wtl-0503
EXPNO	:	4
PROCNO	:	1

*** Acquisition Parameters ***

LOCNUC	:	2H
NS	:	38
NUCLEUS	:	off
O1	:	13204.57 Hz
PULPROG	:	zgpg30
SFO1	:	125.7709936 MHz
SOLVENT	:	CDCl3
SW	:	238.7675 ppm
TD	:	65536
TE	:	295.8 K

*** Processing Parameters ***

LB	:	1.00 Hz
SF	:	125.7577989 MHz

*** 1D NMR Plot Parameters ***

NUCLEUS	:	off

1H AMX500

wtl-877-3

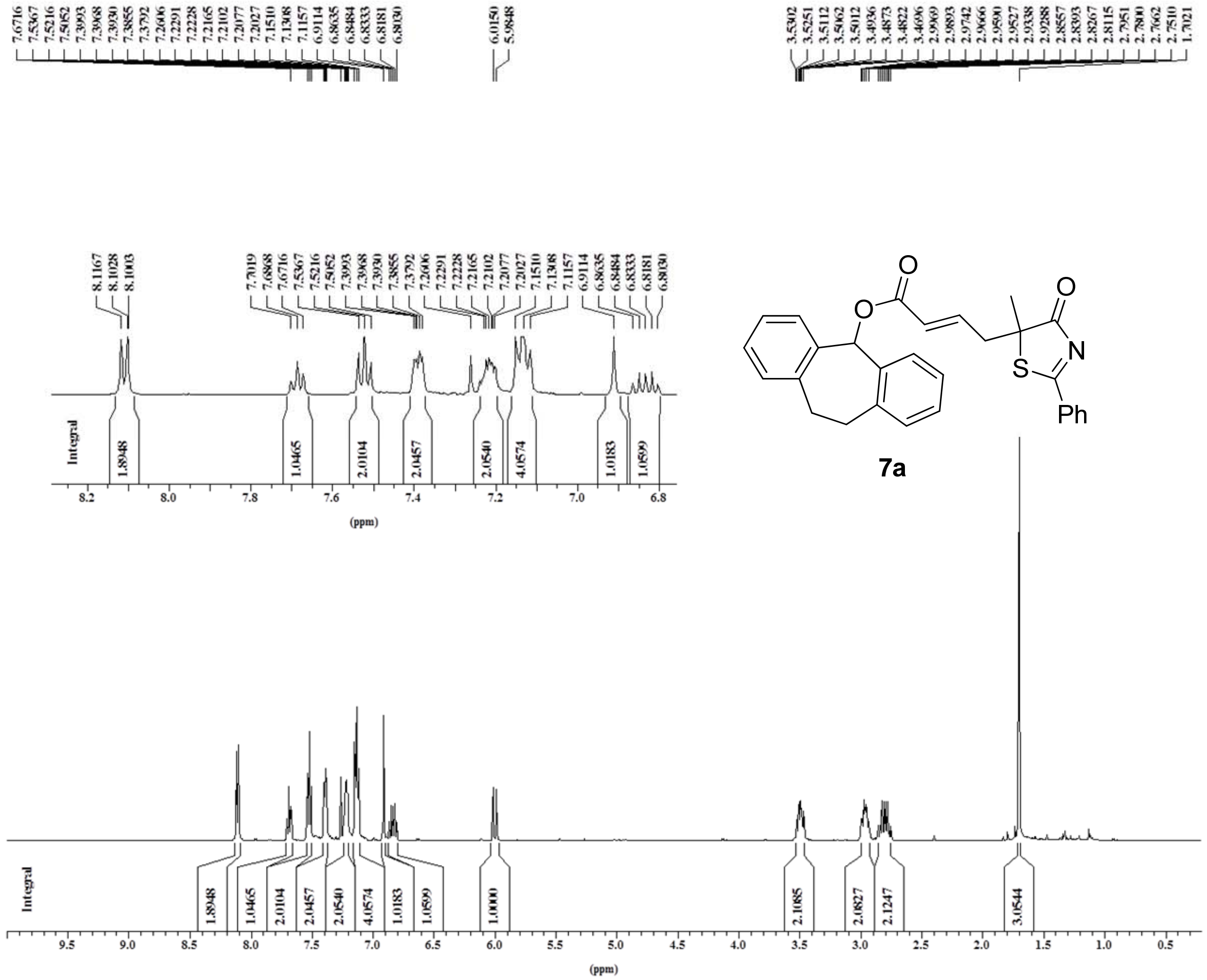

7a

*** Current Data Parameters ***		
NAME	:	wtl-0607
EXPNO	:	1
PROCNO	:	1
*** Acquisition Parameters ***		
LOCNUC	:	2H
NS	:	27
NUCLEUS	:	off
O1	:	3088.51 Hz
PULPROG	:	zg30
SFO1	:	500.1330885 MHz
SOLVENT	:	CDCl3
SW	:	20.6557 ppm
TD	:	32768
TE	:	296.0 K
*** Processing Parameters ***		
LB	:	0.30 Hz
SF	:	500.1300134 MHz
*** 1D NMR Plot Parameters ***		
NUCLEUS	:	off

13C AMX500

wtl-877-3

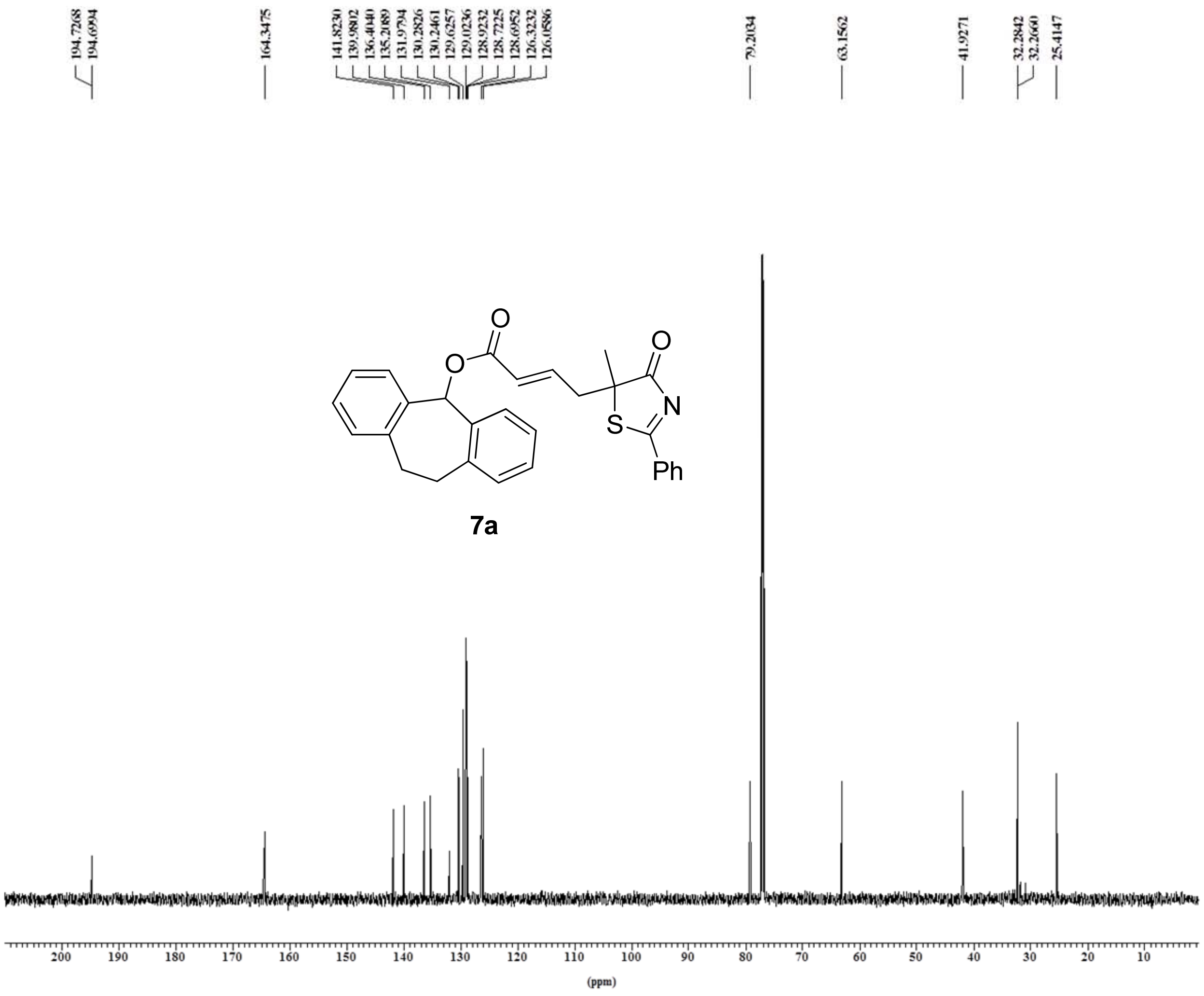

*** Current Data Parameters ***		
NAME	:	wtl-0607
EXPNO	:	2
PROCNO	:	1
*** Acquisition Parameters ***		
LOCNUC	:	2H
NS	:	158
NUCLEUS	:	off
O1	:	18863.67 Hz
PULPROG	:	zgpg30
SFO1	:	125.7766527 MHz
SOLVENT	:	CDCl3
SW	:	298.8948 ppm
TD	:	65536
TE	:	296.3 K
*** Processing Parameters ***		
LB	:	1.00 Hz
SF	:	125.7577974 MHz
*** 1D NMR Plot Parameters ***		
NUCLEUS	:	off

1H AMX500

wtl-890

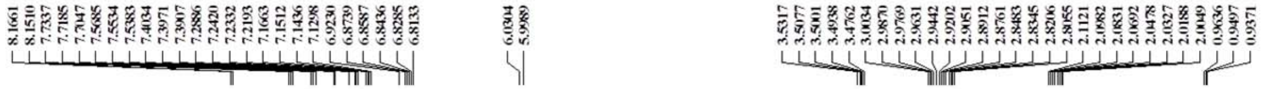

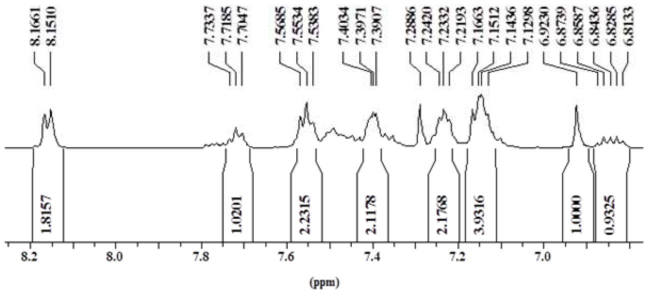

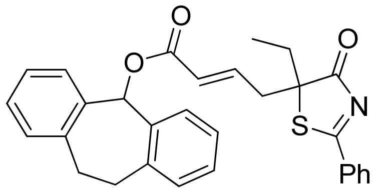

7b

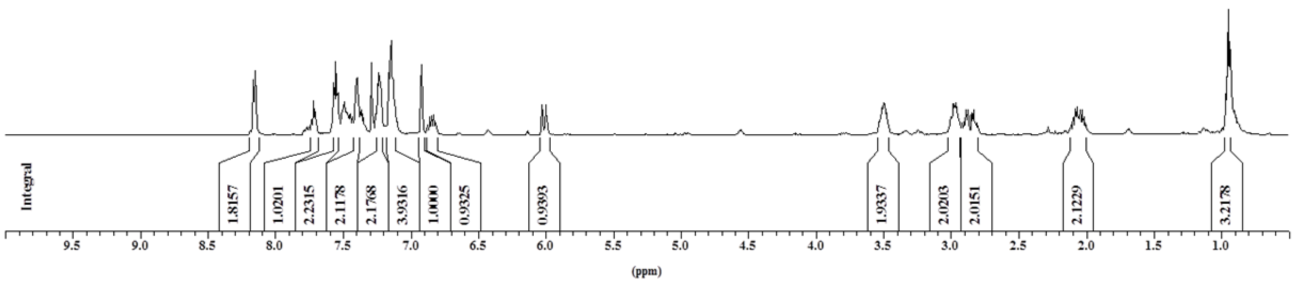

*** Current Data Parameters ***

NAME : wtl-0607

EXPNO : 5

PROCNO : 1

*** Acquisition Parameters ***

LOCNUC : 2H

NS : 11

NUCLEUS : off

O1 : 3088.51 Hz

PULPROG : zg30

SFO1 : 500.1330885 MHz

SOLVENT : CDCl3

SW : 20.6557 ppm

TD : 32768

TE : 296.6 K

*** Processing Parameters ***

LB : 0.30 Hz

SF : 500.1300000 MHz

*** 1D NMR Plot Parameters ***

NUCLEUS : off

13C AMX500

wtl-890

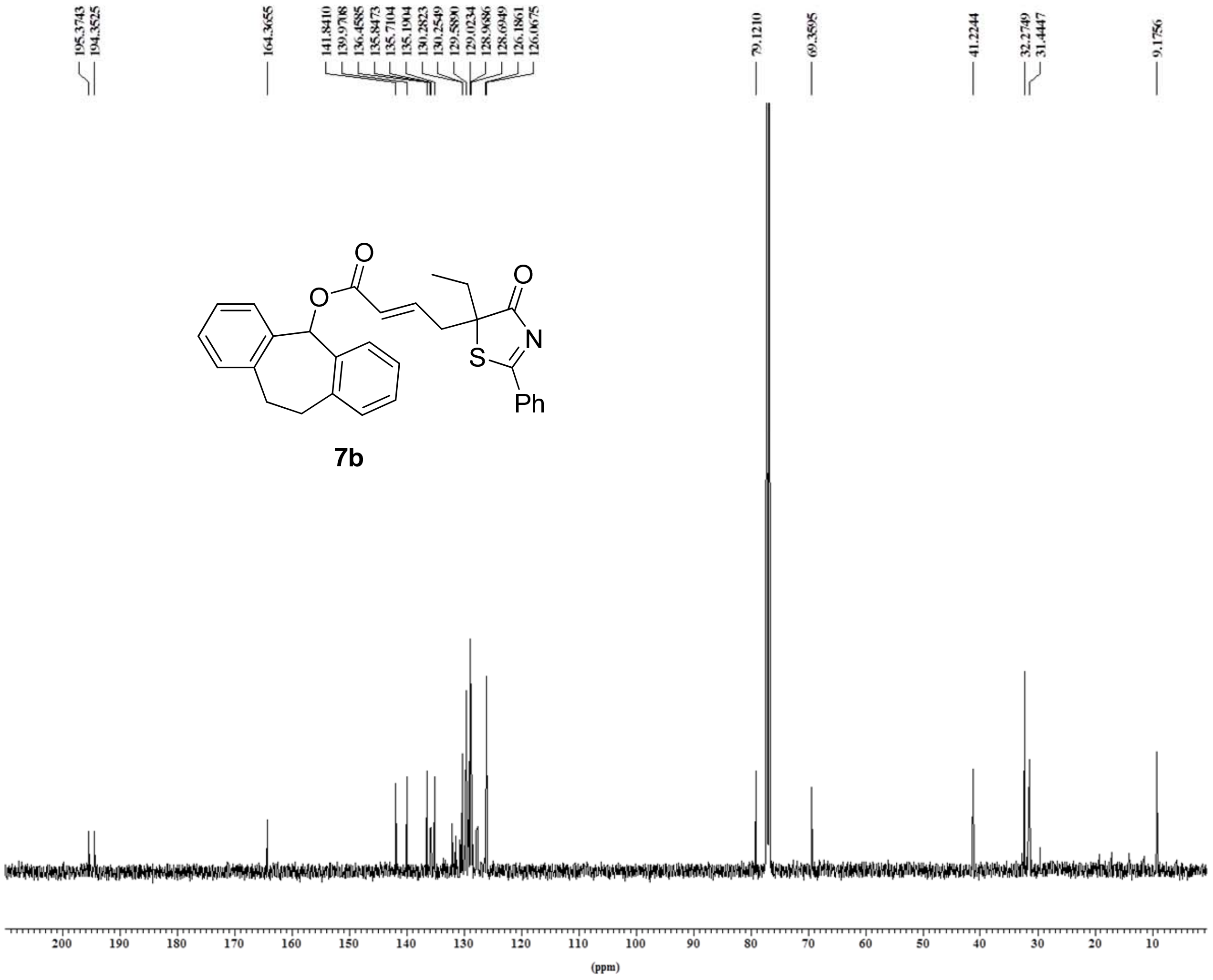

7b

*** Current Data Parameters ***

NAME	:	wtl-0607
EXPNO	:	6
PROCNO	:	1

*** Acquisition Parameters ***

LOCNUC	:	2H
NS	:	1002
NUCLEUS	:	off
O1	:	18863.67 Hz
PULPROG	:	zgpg30
SFO1	:	125.7766527 MHz
SOLVENT	:	CDCl3
SW	:	298.8948 ppm
TD	:	65536
TE	:	296.3 K

*** Processing Parameters ***

LB	:	1.00 Hz
SF	:	125.7577951 MHz

*** 1D NMR Plot Parameters ***

NUCLEUS	:	off

1H AMX500

wtl-960R

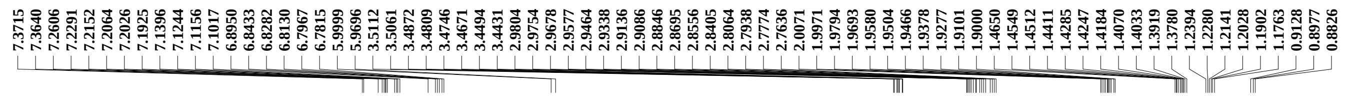

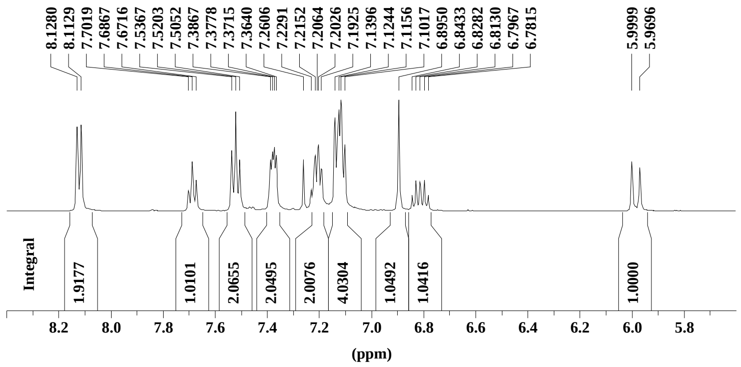

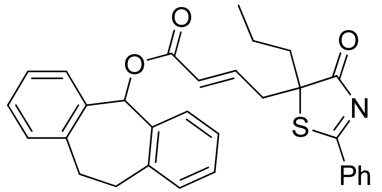

7c

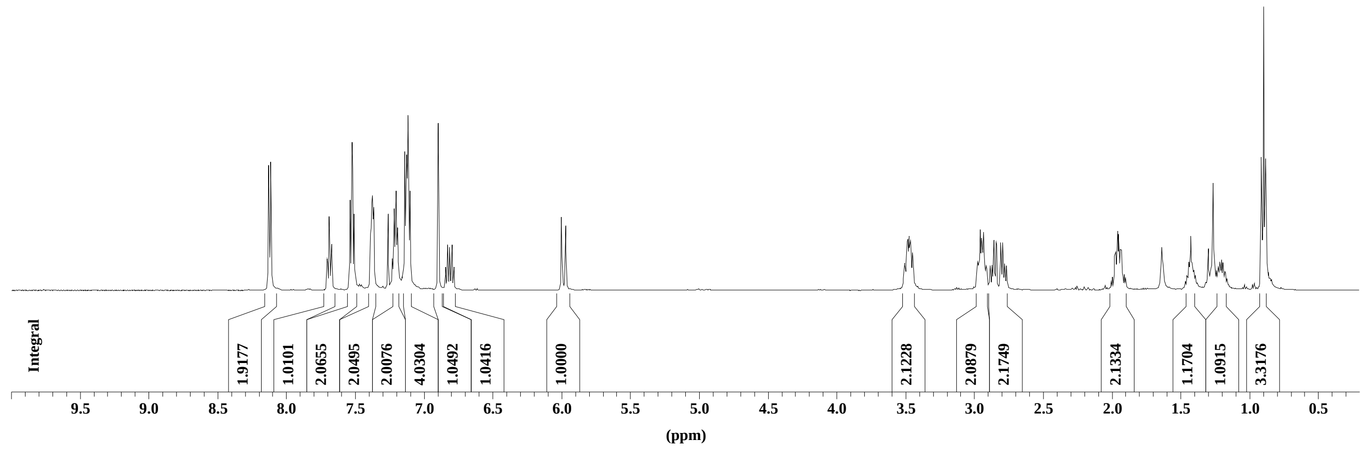

***** Current Data Parameters *****		
**NAME**	**:**	**wtl-0602**
**EXPNO**	**:**	**1**
**PROCNO**	**:**	**1**
***** Acquisition Parameters *****		
**LOCNUC**	**:**	**2H**
**NS**	**:**	**31**
**NUCLEUS**	**:**	**off**
**O1**	**:**	**3088.51 Hz**
**PULPROG**	**:**	**zg30**
**SFO1**	**:**	**500.1330885 MHz**
**SOLVENT**	**:**	**CDCl3**
**SW**	**:**	**20.6557 ppm**
**TD**	**:**	**32768**
**TE**	**:**	**295.2 K**
***** Processing Parameters *****		
**LB**	**:**	**0.30 Hz**
**SF**	**:**	**500.1300140 MHz**
***** 1D NMR Plot Parameters *****		
**NUCLEUS**	**:**	**off**

13C AMX500

wtl-960R

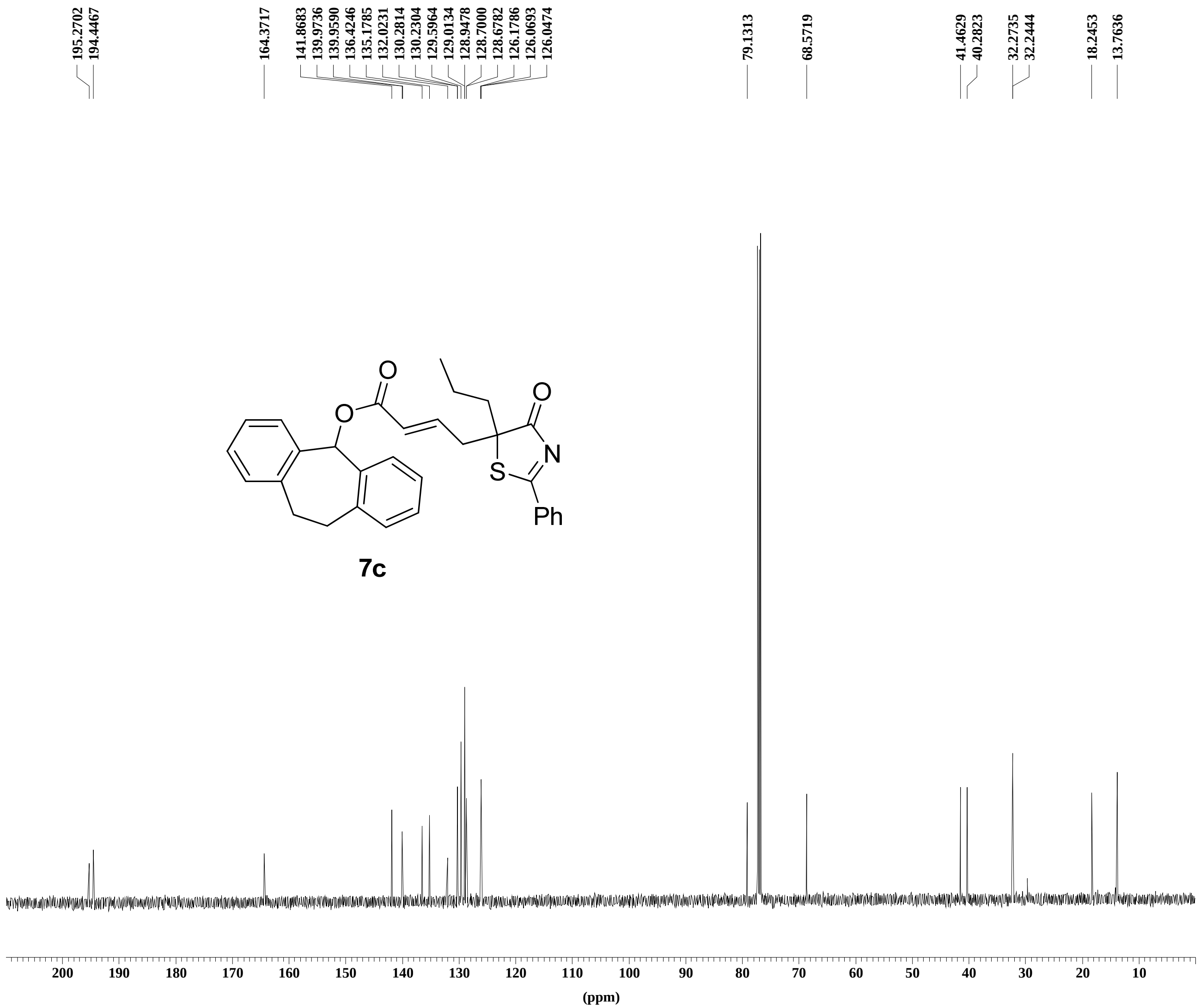

7c

*** Current Data Parameters ***

NAME	:	wtl-0602
EXPNO	:	2
PROCNO	:	1

*** Acquisition Parameters ***

LOCNUC	:	2H
NS	:	204
NUCLEUS	:	off
O1	:	13204.57 Hz
PULPROG	:	zgpg30
SFO1	:	125.7709936 MHz
SOLVENT	:	CDCl3
SW	:	238.7675 ppm
TD	:	65536
TE	:	295.4 K

*** Processing Parameters ***

LB	:	1.00 Hz
SF	:	125.7577961 MHz

*** 1D NMR Plot Parameters ***

NUCLEUS	:	off

1H AMX500

wtl-964 R

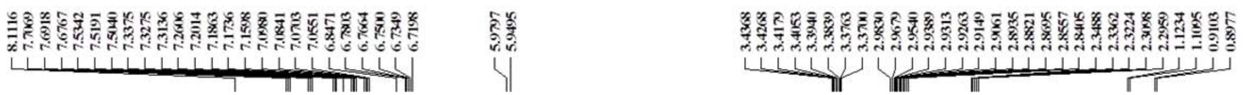

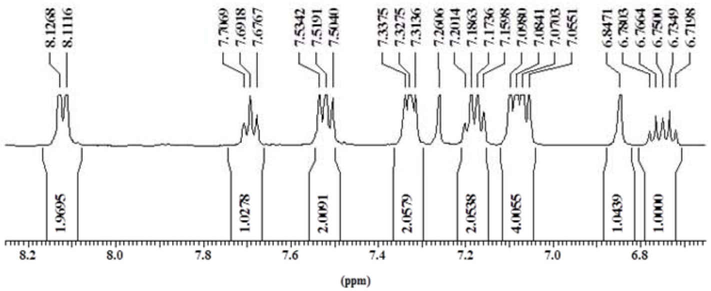

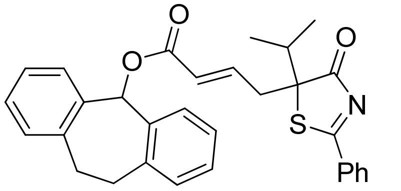

7d

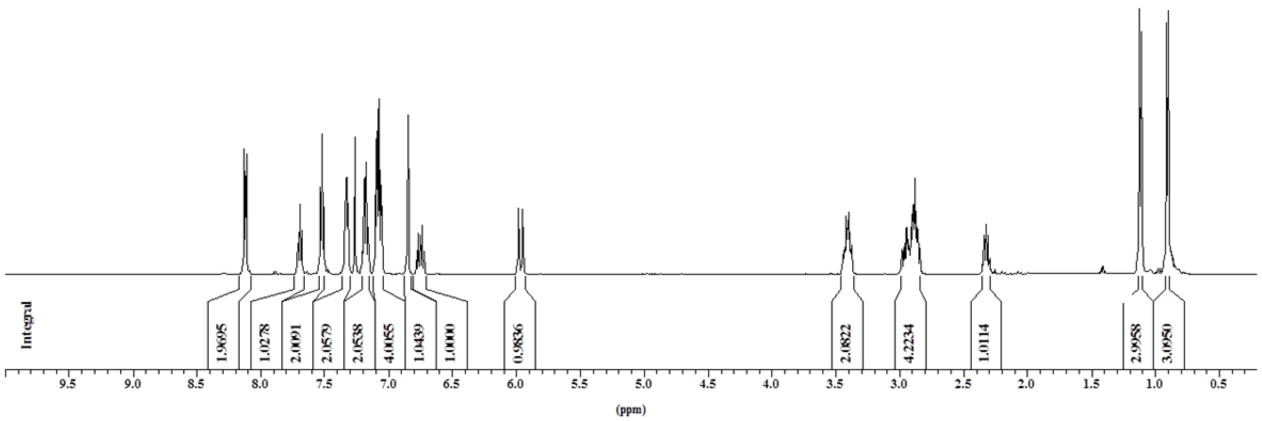

*** Current Data Parameters ***		
NAME	:	wtl-0529
EXPNO	:	1
PROCNO	:	1
*** Acquisition Parameters ***		
LOCNUC	:	2H
NS	:	45
NUCLEUS	:	off
O1	:	3088.51 Hz
PULPROG	:	zg30
SFO1	:	500.1330885 MHz
SOLVENT	:	CDCl3
SW	:	20.6557 ppm
TD	:	32768
TE	:	295.5 K
*** Processing Parameters ***		
LB	:	0.30 Hz
SF	:	500.1300134 MHz
*** 1D NMR Plot Parameters ***		
NUCLEUS	:	off

13C AMX500

wtl-964 R

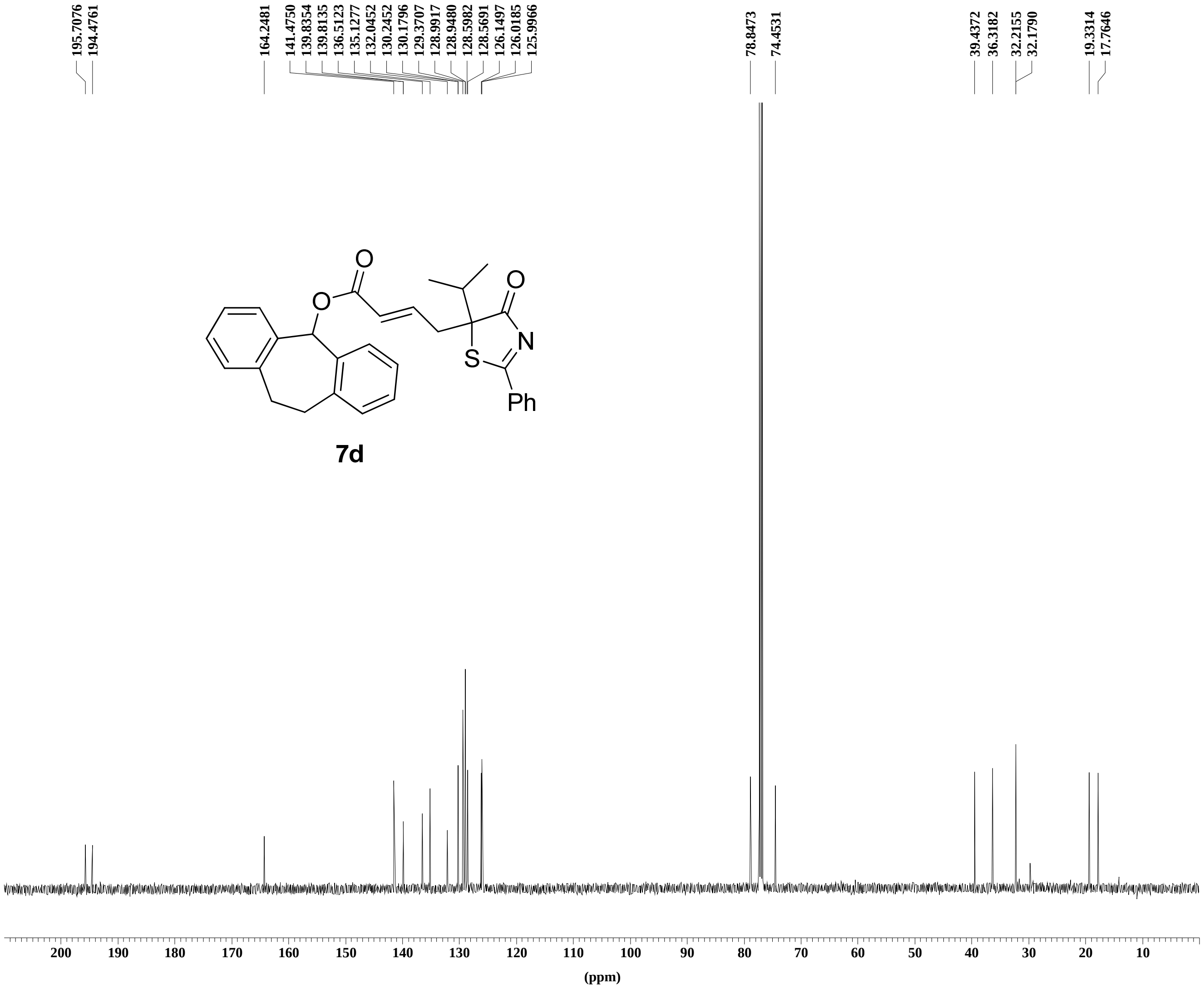

7d

*** Current Data Parameters ***

NAME	:	wtl-0529
EXPNO	:	2
PROCNO	:	1

*** Acquisition Parameters ***

LOCNUC	:	2H
NS	:	627
NUCLEUS	:	off
O1	:	13204.57 Hz
PULPROG	:	zgpg30
SFO1	:	125.7709936 MHz
SOLVENT	:	CDCl3
SW	:	238.7675 ppm
TD	:	65536
TE	:	295.8 K

*** Processing Parameters ***

LB	:	1.00 Hz
SF	:	125.7577943 MHz

*** 1D NMR Plot Parameters ***

NUCLEUS	:	off

1H AMX500

wtl-961R

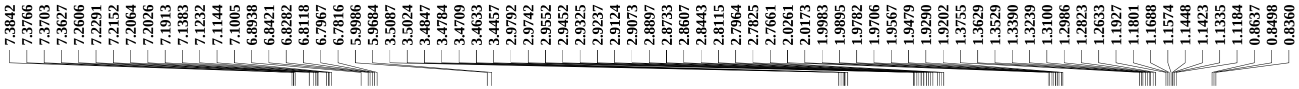

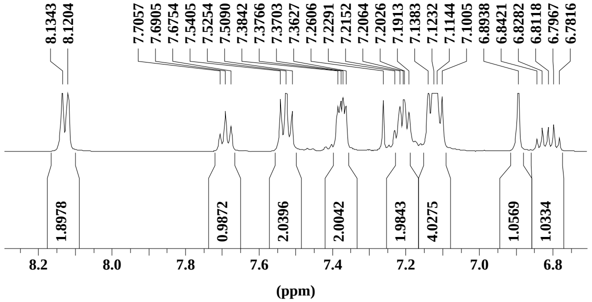

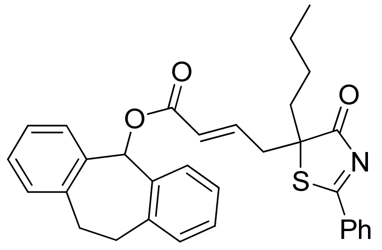

7e

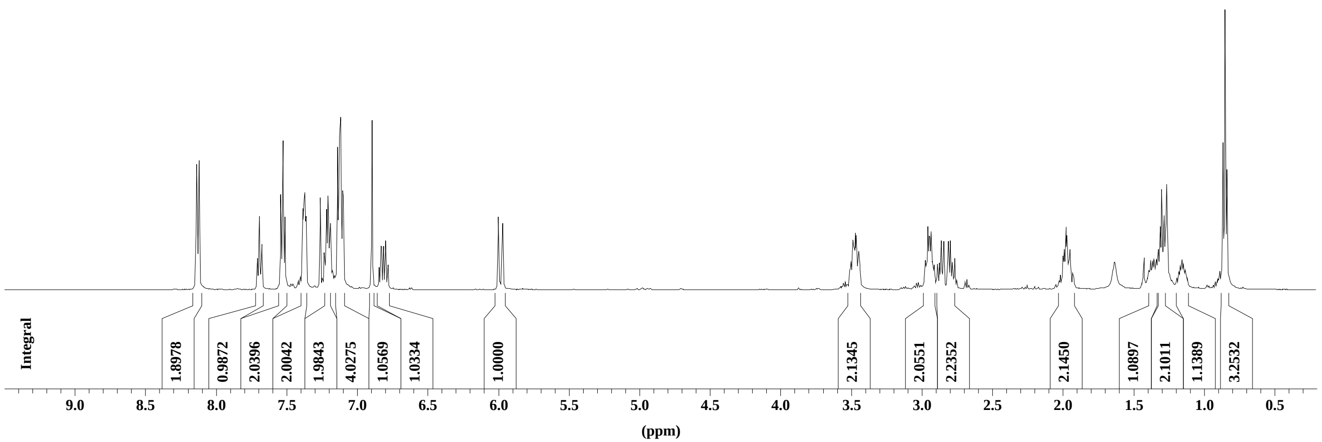

*** Current Data Parameters ***

NAME	:	wtl-0602
EXPNO	:	3
PROCNO	:	1

*** Acquisition Parameters ***

LOCNUC	:	2H
NS	:	28
NUCLEUS	:	off
O1	:	3088.51 Hz
PULPROG	:	zg30
SFO1	:	500.1330885 MHz
SOLVENT	:	CDCl3
SW	:	20.6557 ppm
TD	:	32768
TE	:	295.5 K

*** Processing Parameters ***

LB	:	0.30 Hz
SF	:	500.1300134 MHz

*** 1D NMR Plot Parameters ***

NUCLEUS	:	off

13C AMX500

wtl-961R

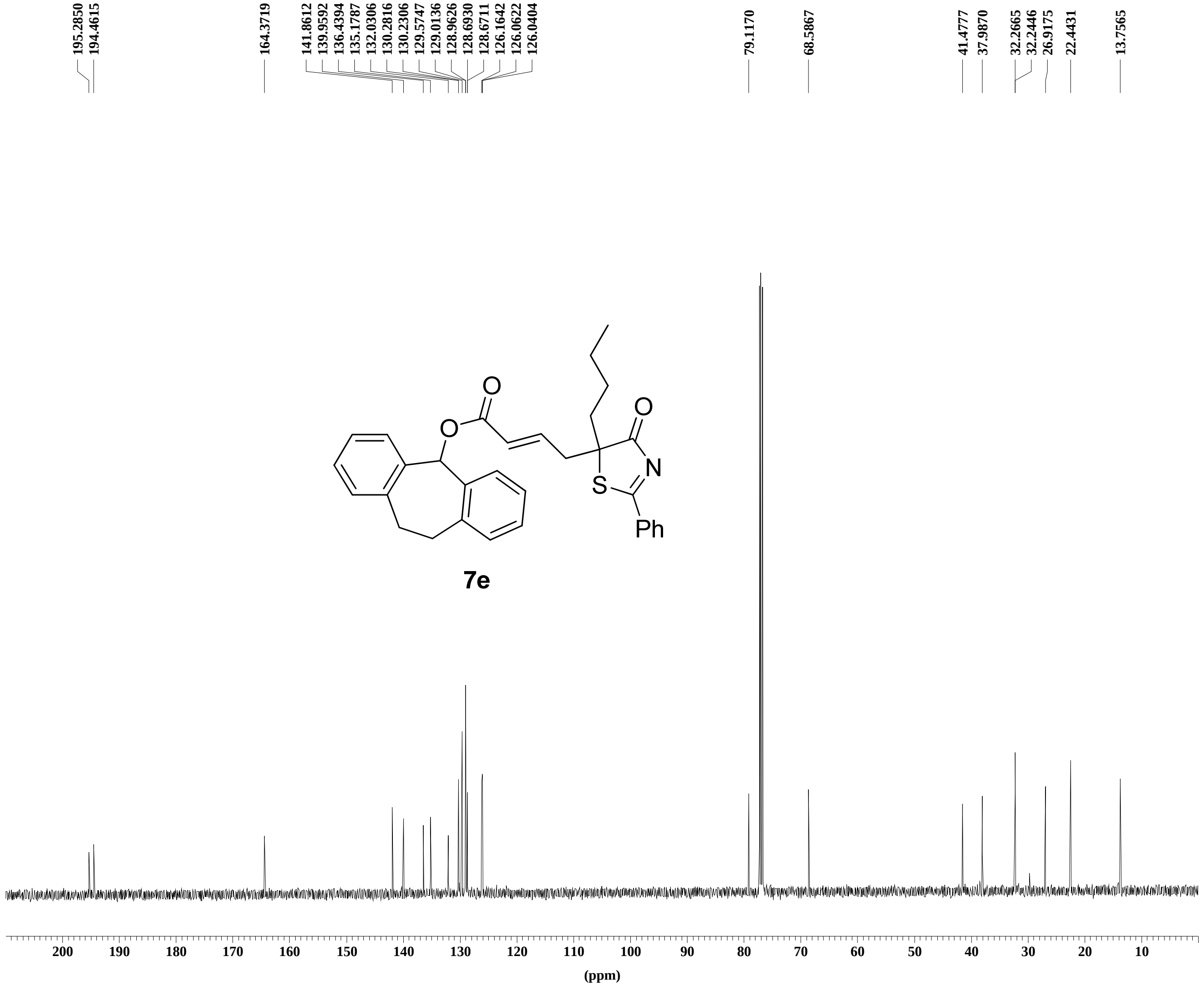

7e

*** Current Data Parameters ***

NAME : wtl-0602

EXPNO : 4

PROCNO : 1

*** Acquisition Parameters ***

LOCNUC : 2H

NS : 319

NUCLEUS : off

O1 : 13204.57 Hz

PULPROG : zgpg30

SFO1 : 125.7709936 MHz

SOLVENT : CDCl3

SW : 238.7675 ppm

TD : 65536

TE : 295.5 K

*** Processing Parameters ***

LB : 1.00 Hz

SF : 125.7577961 MHz

*** 1D NMR Plot Parameters ***

NUCLEUS : off

1H AMX500

wtl-973 R

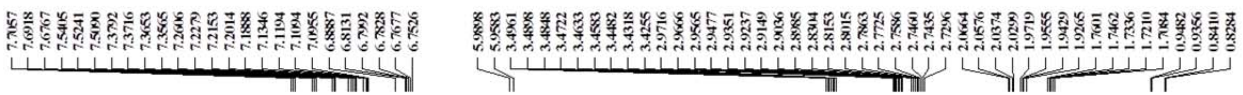

*** Current Data Parameters ***

NAME	:	wtl-0614
EXPNO	:	1
PROCNO	:	1

*** Acquisition Parameters ***

LOCNUC	:	2H
NS	:	21
NUCLEUS	:	off
O1	:	3088.51 Hz
PULPROG	:	zg30
SFO1	:	500.1330885 MHz
SOLVENT	:	CDCl3
SW	:	20.6557 ppm
TD	:	32768
TE	:	296.3 K

*** Processing Parameters ***

LB	:	0.30 Hz
SF	:	500.1300134 MHz

*** 1D NMR Plot Parameters ***

NUCLEUS	:	off

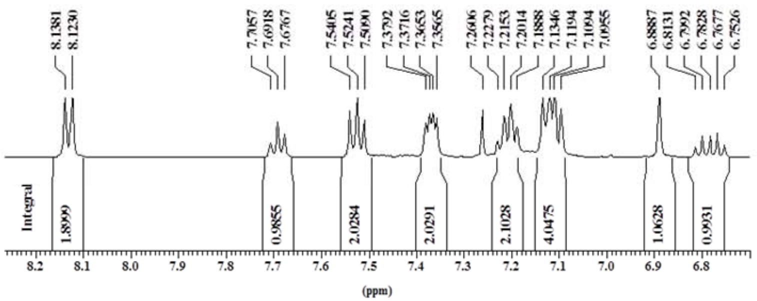

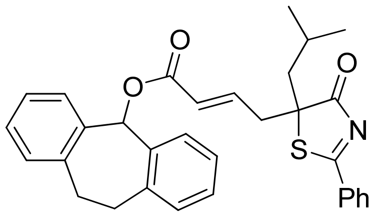

7f

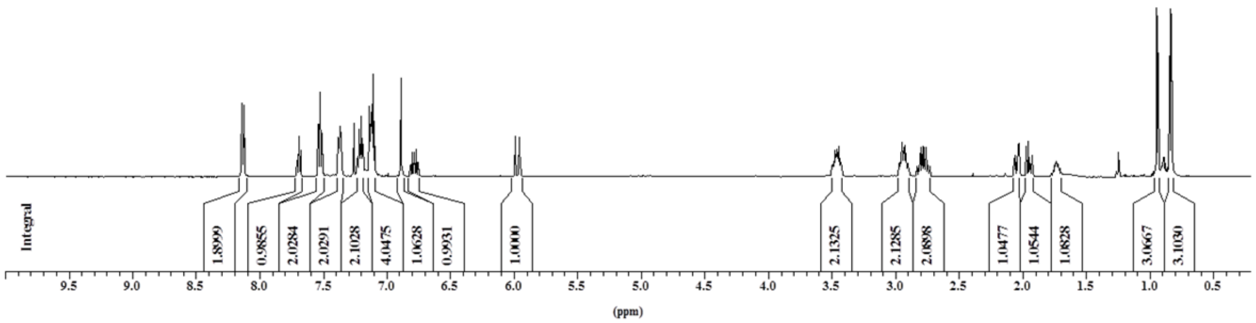

13C AMX500

wtl-973 R

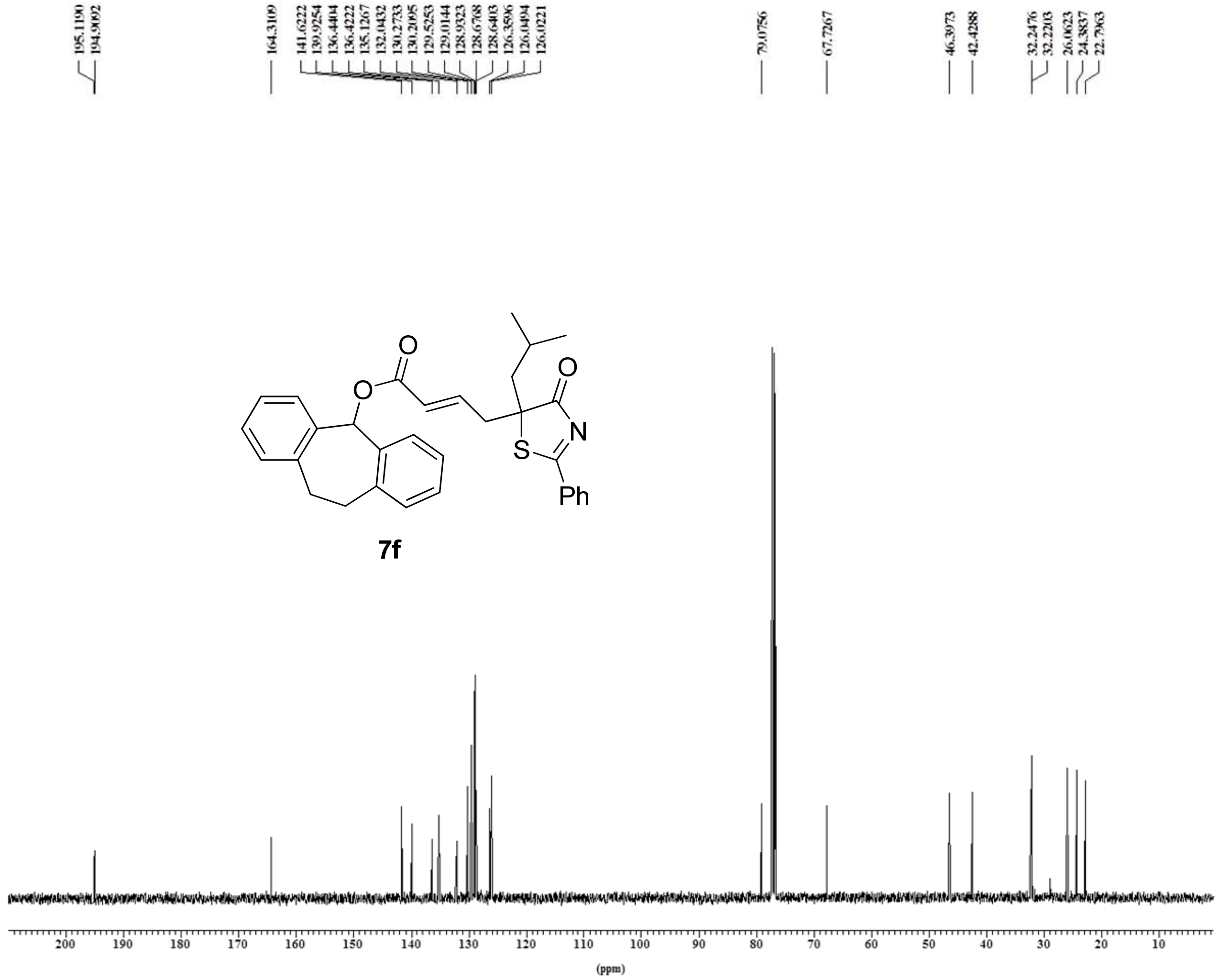

7f

*** Current Data Parameters ***

NAME : wtl-0614

EXPNO : 2

PROCNO : 1

*** Acquisition Parameters ***

LOCNUC : 2H

NS : 154

NUCLEUS : off

O1 : 18863.67 Hz

PULPROG : zgpg30

SFO1 : 125.7766527 MHz

SOLVENT : CDCl3

SW : 298.8948 ppm

TD : 65536

TE : 296.4 K

*** Processing Parameters ***

LB : 1.00 Hz

SF : 125.7577974 MHz

*** 1D NMR Plot Parameters ***

NUCLEUS : off

1H AMX500

wtl-962R

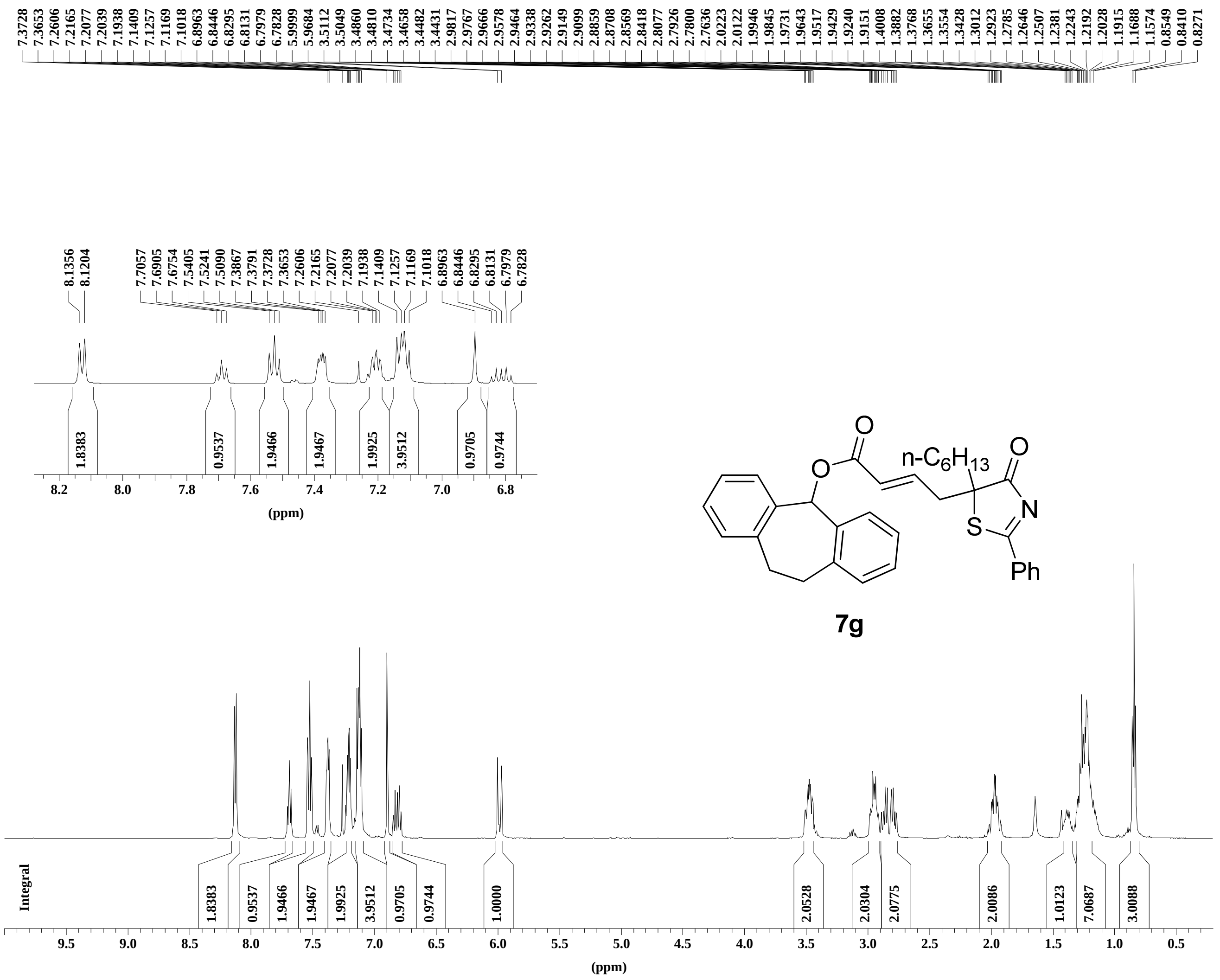

*** Current Data Parameters ***

NAME	:	wtl-0602
EXPNO	:	5
PROCNO	:	1

*** Acquisition Parameters ***

LOCNUC	:	2H
NS	:	18
NUCLEUS	:	off
O1	:	3088.51 Hz
PULPROG	:	zg30
SFO1	:	500.1330885 MHz
SOLVENT	:	CDCl3
SW	:	20.6557 ppm
TD	:	32768
TE	:	295.6 K

*** Processing Parameters ***

LB	:	0.30 Hz
SF	:	500.1300134 MHz

*** 1D NMR Plot Parameters ***

NUCLEUS	:	off

13C AMX500

wtl-962R

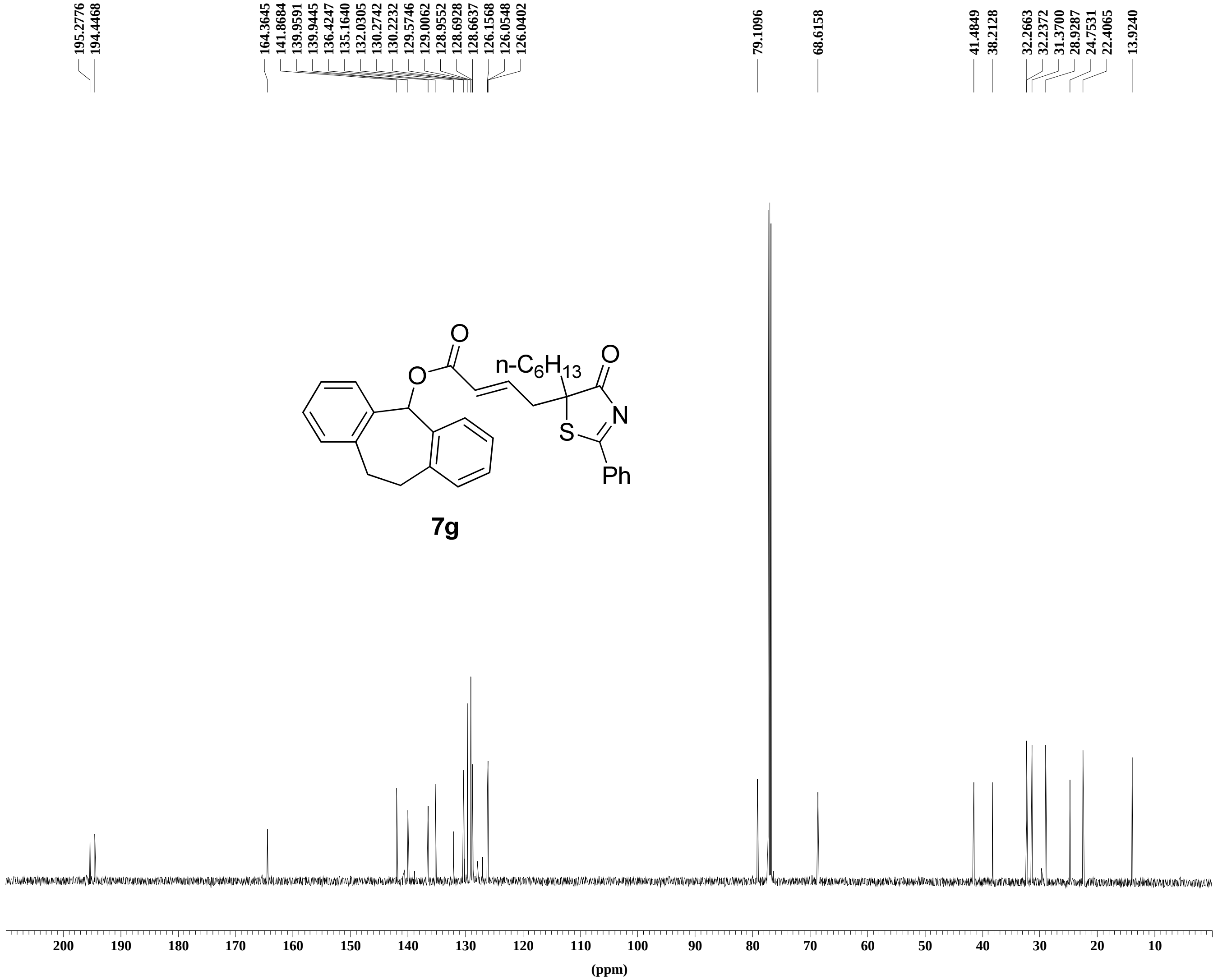

7g

*** Current Data Parameters ***

NAME	:	wtl-0602
EXPNO	:	6
PROCNO	:	1

*** Acquisition Parameters ***

LOCNUC	:	2H
NS	:	395
NUCLEUS	:	off
O1	:	13204.57 Hz
PULPROG	:	zgpg30
SFO1	:	125.7709936 MHz
SOLVENT	:	CDCl3
SW	:	238.7675 ppm
TD	:	65536
TE	:	295.6 K

*** Processing Parameters ***

LB	:	1.00 Hz
SF	:	125.7577970 MHz

*** 1D NMR Plot Parameters ***

NUCLEUS	:	off

1H AMX500

wtl-975 R

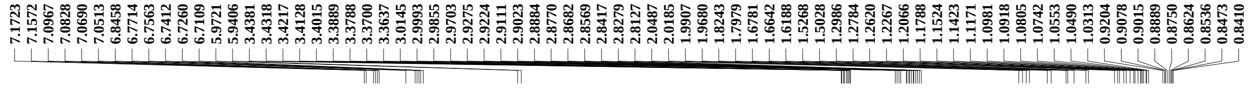

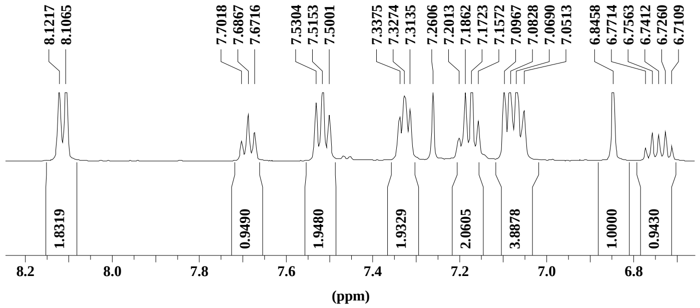

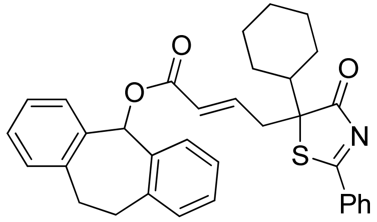

7h

****** Current Data Parameters ******		
**NAME**	**:**	**wtl-0604**
**EXPNO**	**:**	**3**
**PROCNO**	**:**	**1**
****** Acquisition Parameters ******		
**LOCNUC**	**:**	**2H**
**NS**	**:**	**15**
**NUCLEUS**	**:**	**off**
**O1**	**:**	**3088.51 Hz**
**PULPROG**	**:**	**zg30**
**SFO1**	**:**	**500.1330885 MHz**
**SOLVENT**	**:**	**CDCl3**
**SW**	**:**	**20.6557 ppm**
**TD**	**:**	**32768**
**TE**	**:**	**295.3 K**
****** Processing Parameters ******		
**LB**	**:**	**0.30 Hz**
**SF**	**:**	**500.1300134 MHz**
****** 1D NMR Plot Parameters ******		
**NUCLEUS**	**:**	**off**

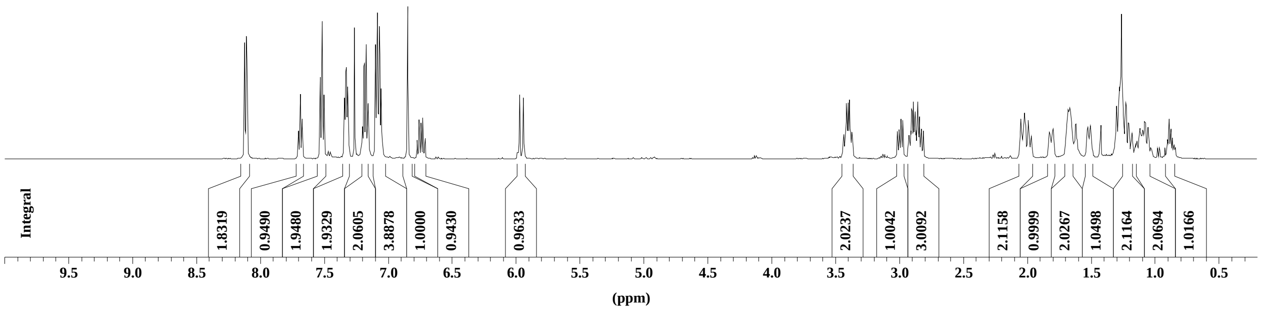

13C AMX500

wtl-975 R

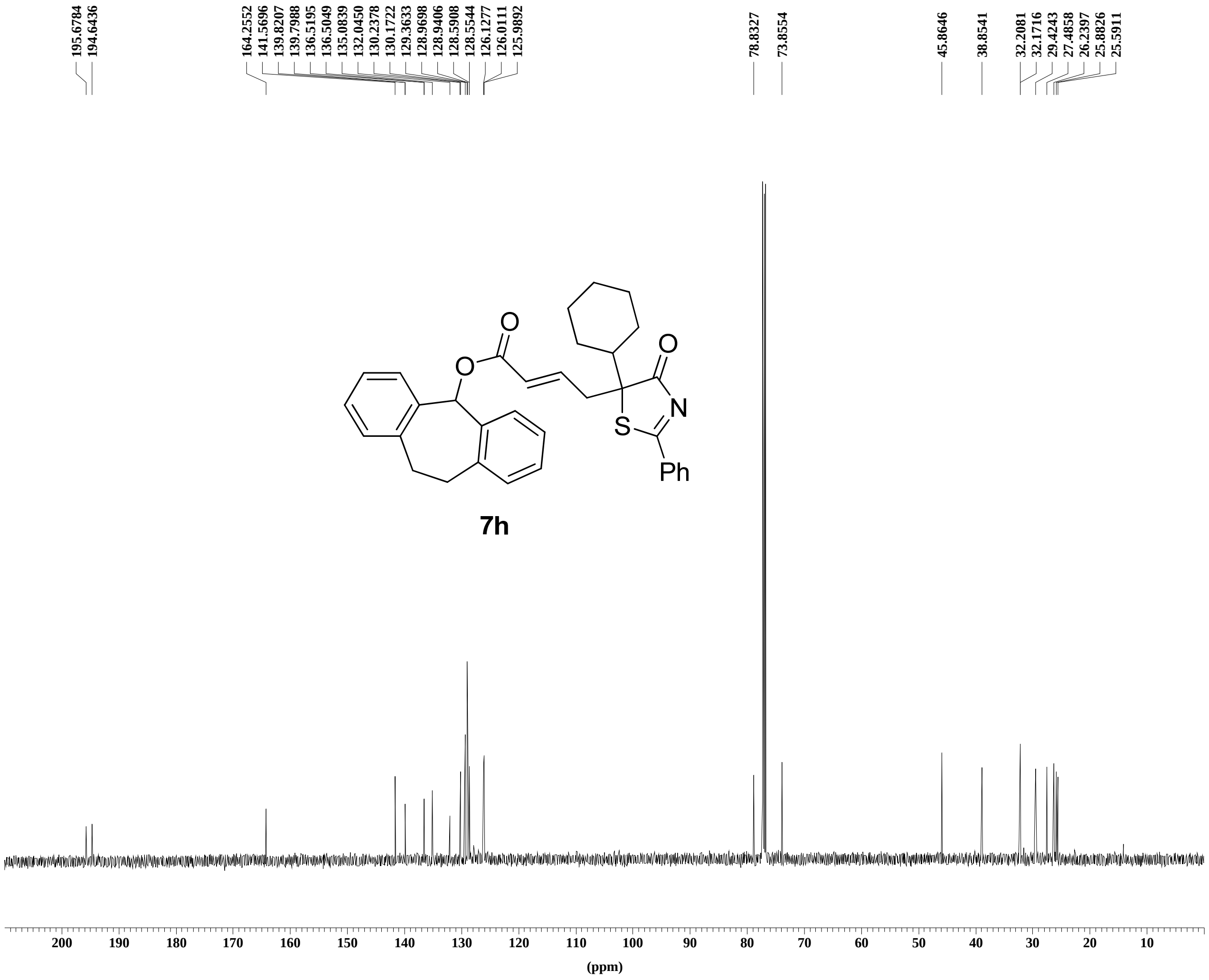

7h

*** Current Data Parameters ***

NAME : wtl-0604

EXPNO : 4

PROCNO : 1

*** Acquisition Parameters ***

LOCNUC : 2H

NS : 198

NUCLEUS : off

O1 : 13204.57 Hz

PULPROG : zgpg30

SFO1 : 125.7709936 MHz

SOLVENT : CDCl3

SW : 238.7675 ppm

TD : 65536

TE : 295.3 K

*** Processing Parameters ***

LB : 1.00 Hz

SF : 125.7577952 MHz

*** 1D NMR Plot Parameters ***

NUCLEUS : off

1H AMX500

wtl-965 R

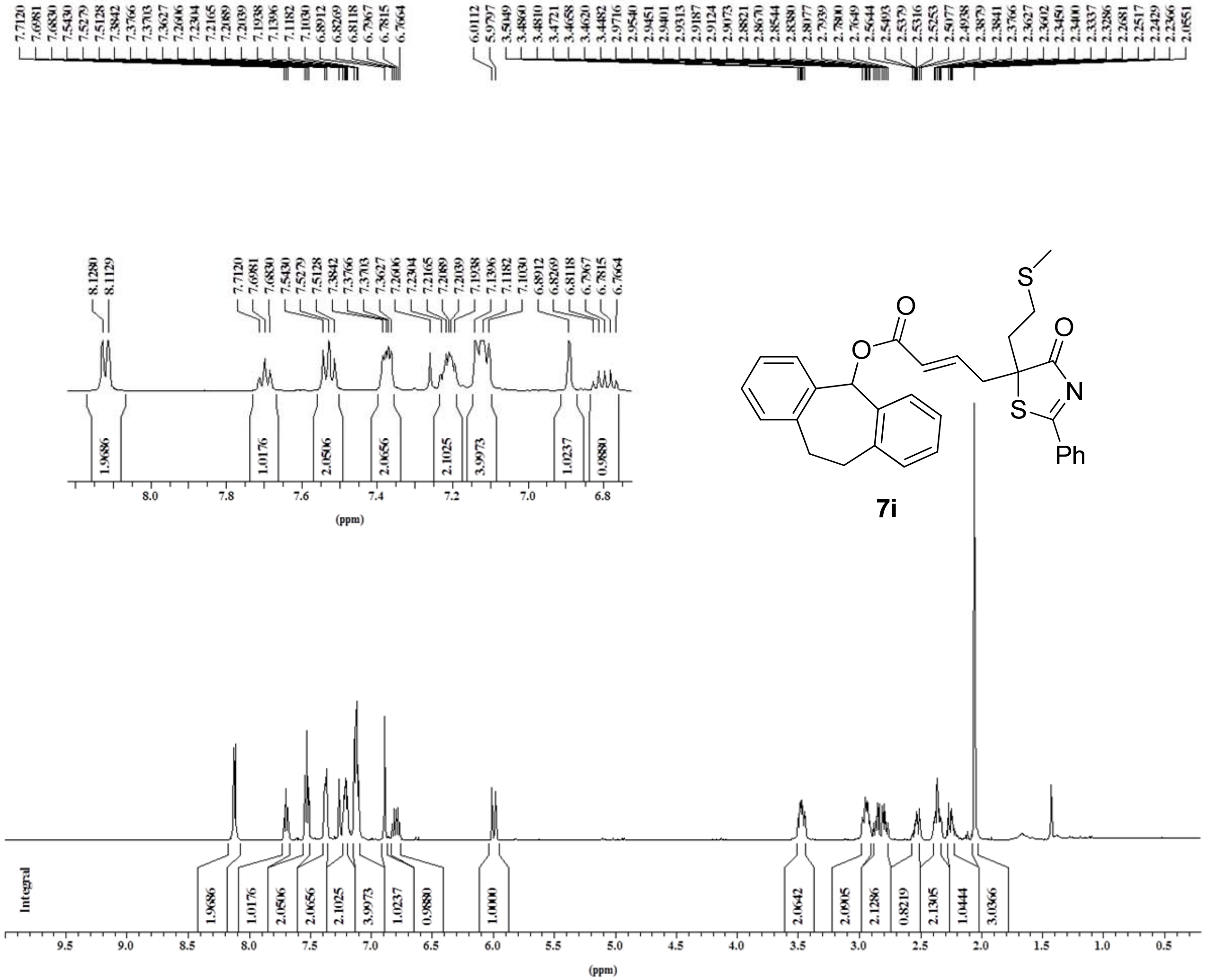

7i

*** Current Data Parameters ***		
NAME	:	wtl-0604
EXPNO	:	1
PROCNO	:	1
*** Acquisition Parameters ***		
LOCNUC	:	2H
NS	:	24
NUCLEUS	:	off
O1	:	3088.51 Hz
PULPROG	:	zg30
SFO1	:	500.1330885 MHz
SOLVENT	:	CDCl3
SW	:	20.6557 ppm
TD	:	32768
TE	:	295.2 K
*** Processing Parameters ***		
LB	:	0.30 Hz
SF	:	500.1300140 MHz
*** 1D NMR Plot Parameters ***		
NUCLEUS	:	off

13C AMX500

wtl-965 R

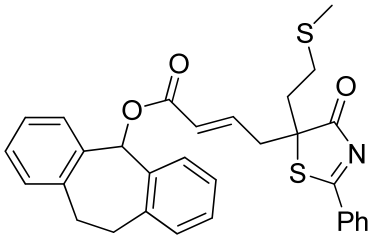

7i

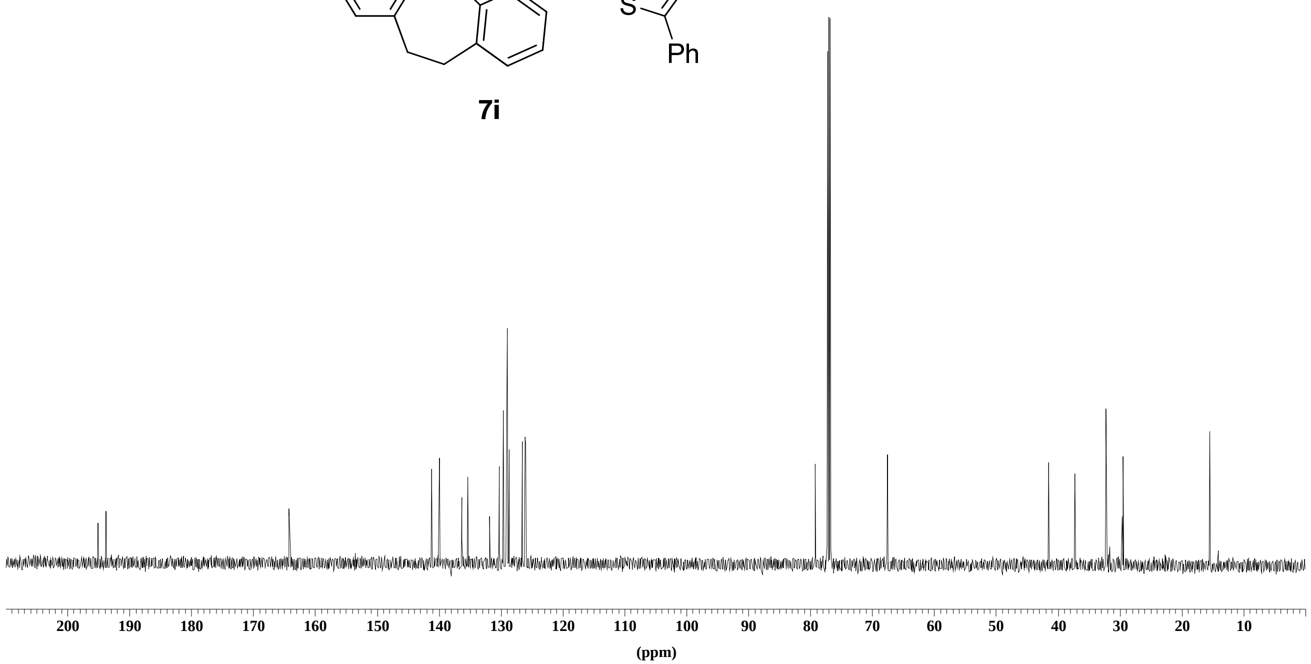

*** Current Data Parameters ***

NAME	:	wtl-0604
EXPNO	:	2
PROCNO	:	1

*** Acquisition Parameters ***

LOCNUC	:	2H
NS	:	108
NUCLEUS	:	off
O1	:	13204.57 Hz
PULPROG	:	zgpg30
SFO1	:	125.7709936 MHz
SOLVENT	:	CDCl3
SW	:	238.7675 ppm
TD	:	65536
TE	:	295.2 K

*** Processing Parameters ***

LB	:	1.00 Hz
SF	:	125.7577990 MHz

*** 1D NMR Plot Parameters ***

NUCLEUS	:	off

1H AMX500

wtl-974R

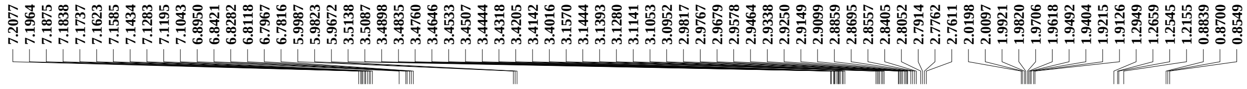

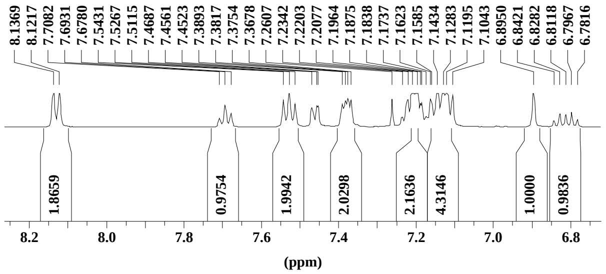

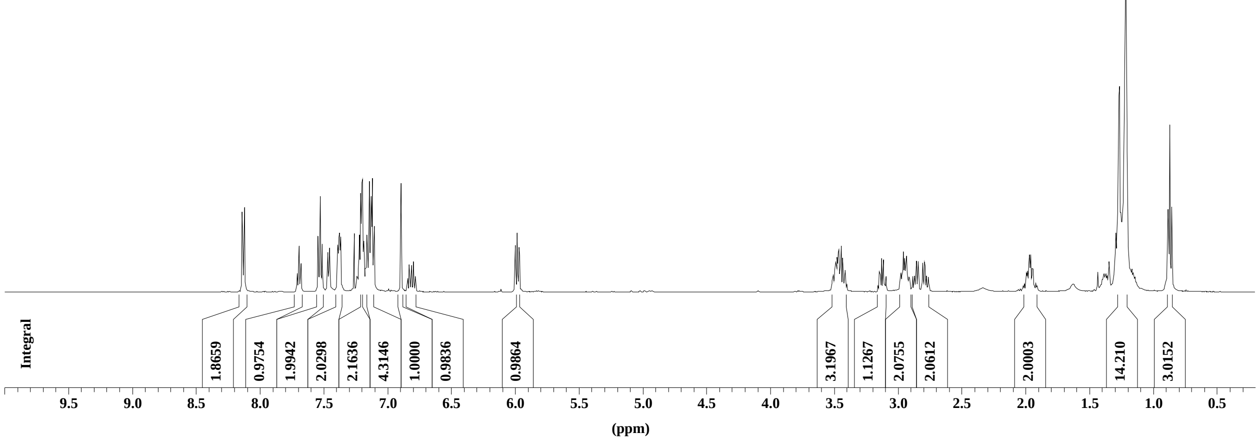

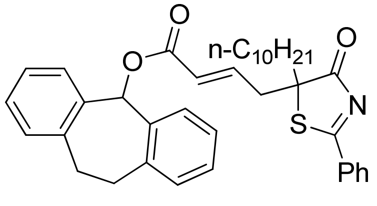

7j

***** Current Data Parameters *****

NAME	:	wtl-0616
EXPNO	:	1
PROCNO	:	1

***** Acquisition Parameters *****

LOCNUC	:	2H
NS	:	19
NUCLEUS	:	off
O1	:	3088.51 Hz
PULPROG	:	zg30
SFO1	:	500.1330885 MHz
SOLVENT	:	CDCl3
SW	:	20.6557 ppm
TD	:	32768
TE	:	293.4 K

***** Processing Parameters *****

LB	:	0.30 Hz
SF	:	500.1300134 MHz

***** 1D NMR Plot Parameters *****

NUCLEUS	:	off

13C AMX500

wtl-974R

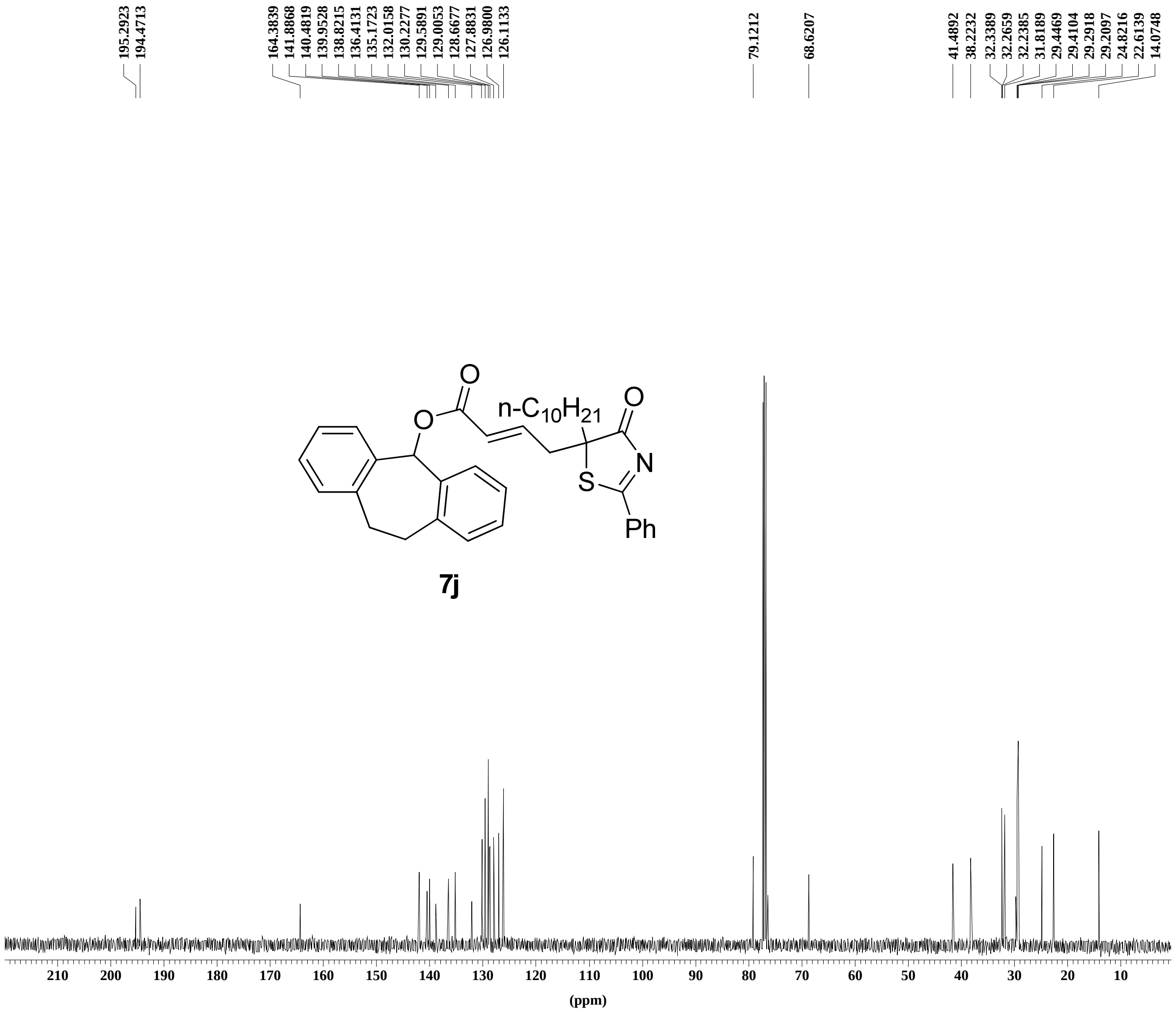

7j

***** Current Data Parameters *****

NAME	:	wtl-0616	
EXPNO	:	2	
PROCNO	:	1	

***** Acquisition Parameters *****

LOCNUC	:	2H	
NS	:	215	
NUCLEUS	:	off	
O1	:	18863.67	Hz
PULPROG	:	zgpg30	
SFO1	:	125.7766527	MHz
SOLVENT	:	CDCl3	
SW	:	298.8948	ppm
TD	:	65536	
TE	:	293.4	K

***** Processing Parameters *****

LB	:	1.00	Hz
SF	:	125.7577974	MHz

***** 1D NMR Plot Parameters *****

NUCLEUS	:	off	

1H AMX500

wtl-963 R

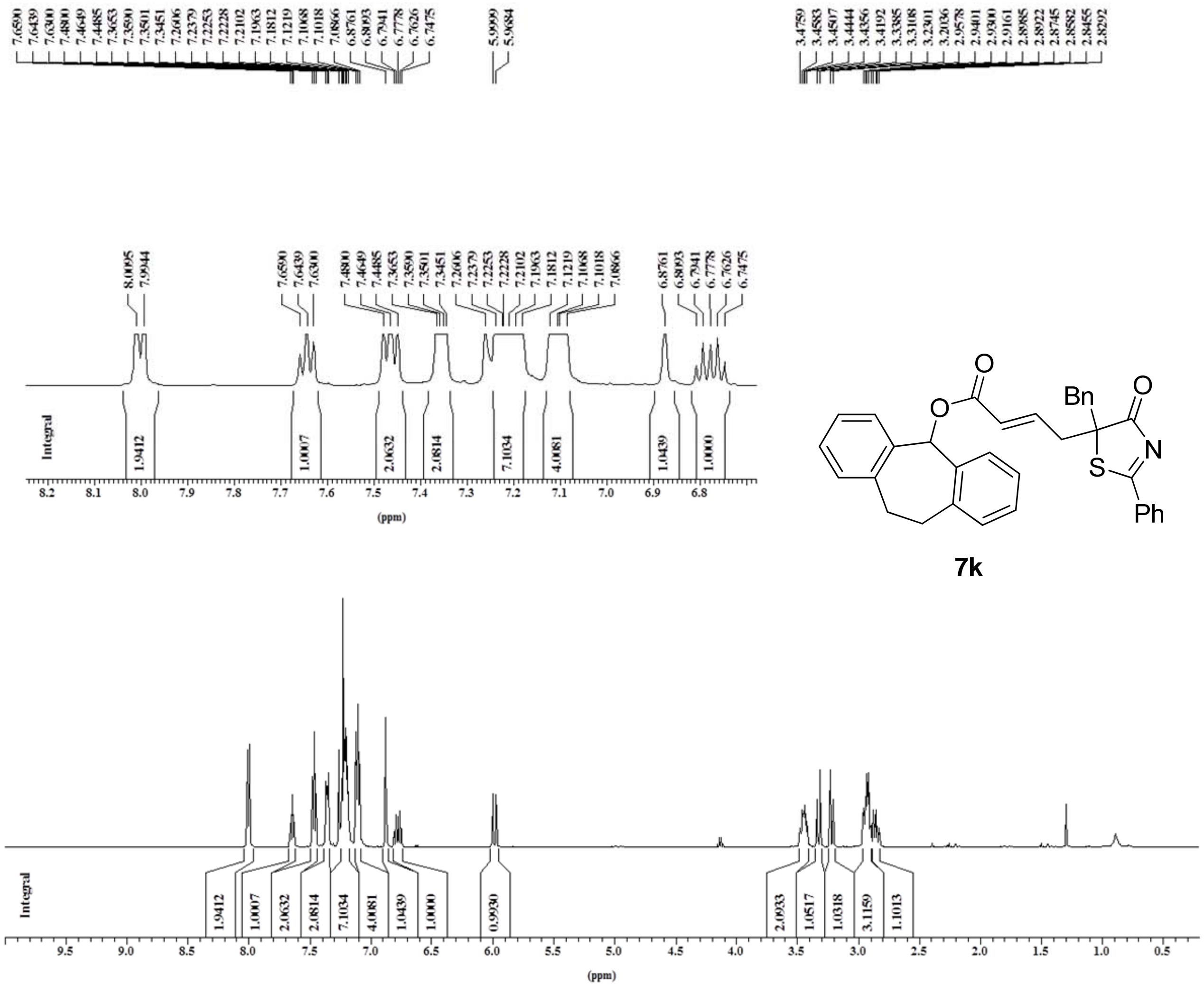

7k

*** Current Data Parameters ***

NAME : wtl-0529

EXPNO : 3

PROCNO : 1

*** Acquisition Parameters ***

LOCNUC : 2H

NS : 33

NUCLEUS : off

O1 : 3088.51 Hz

PULPROG : zg30

SFO1 : 500.1330885 MHz

SOLVENT : CDCl3

SW : 20.6557 ppm

TD : 32768

TE : 295.9 K

*** Processing Parameters ***

LB : 0.30 Hz

SF : 500.1300134 MHz

*** 1D NMR Plot Parameters ***

NUCLEUS : off

13C AMX500

wtl-963 R

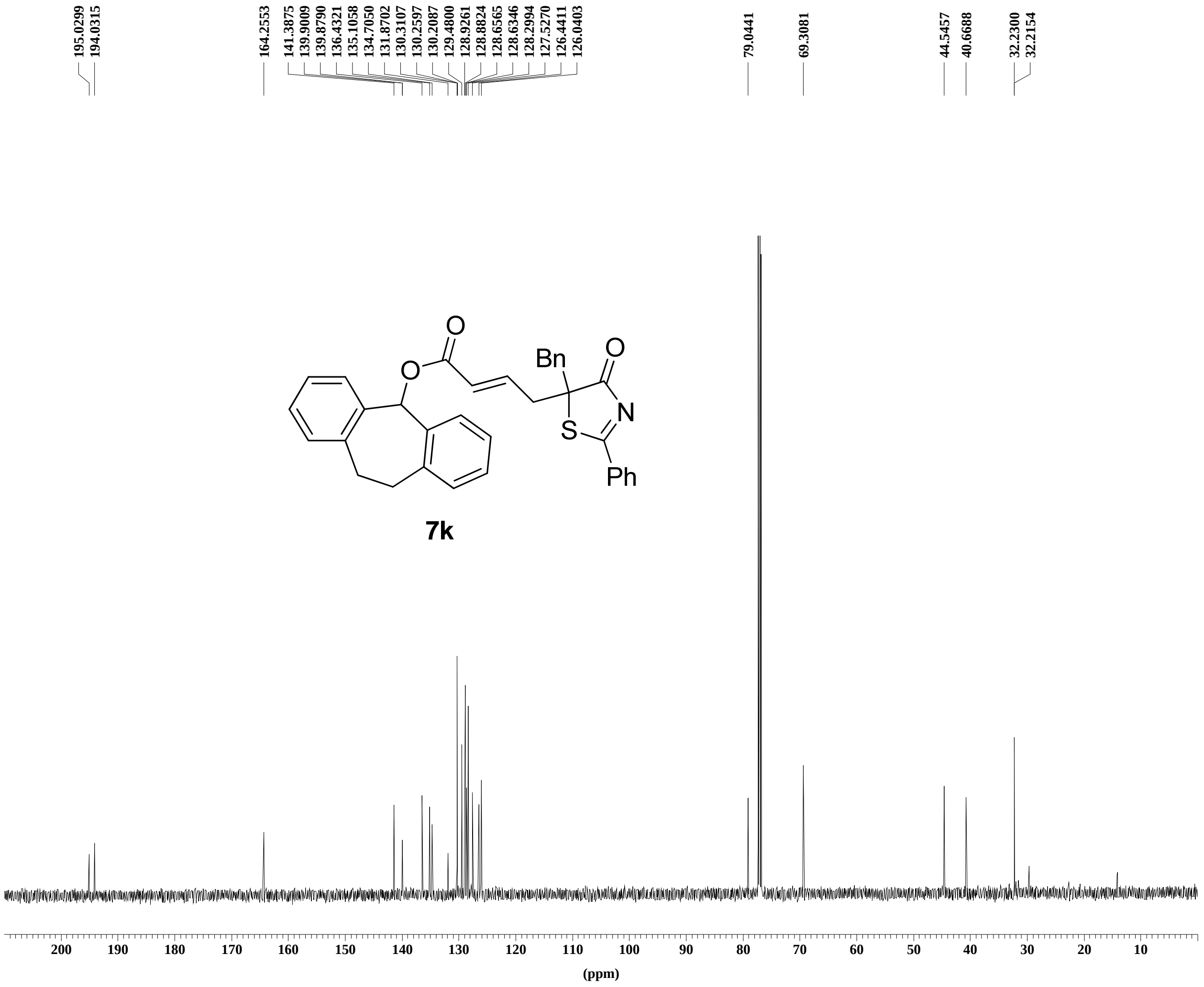

7k

*** Current Data Parameters ***

NAME	:	wtl-0529
EXPNO	:	4
PROCNO	:	1

*** Acquisition Parameters ***

LOCNUC	:	2H
NS	:	159
NUCLEUS	:	off
O1	:	13204.57 Hz
PULPROG	:	zgpg30
SFO1	:	125.7709936 MHz
SOLVENT	:	CDCl3
SW	:	238.7675 ppm
TD	:	65536
TE	:	296.0 K

*** Processing Parameters ***

LB	:	1.00 Hz
SF	:	125.7577970 MHz

*** 1D NMR Plot Parameters ***

NUCLEUS	:	off

1H AMX500

wtl-887

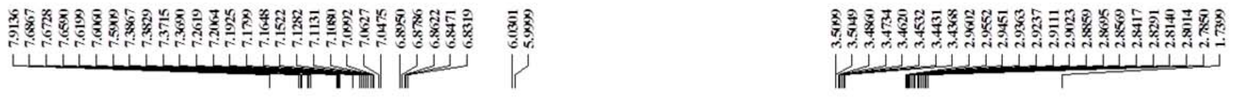

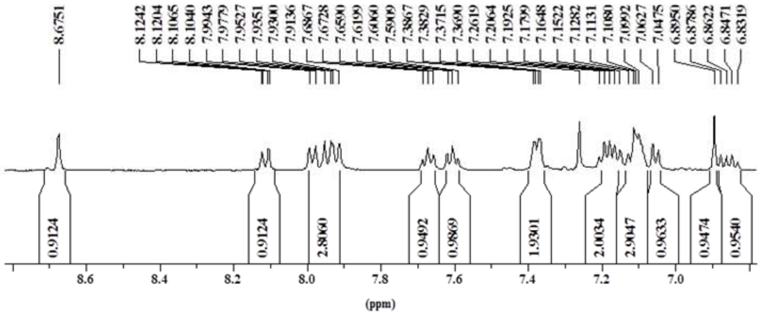

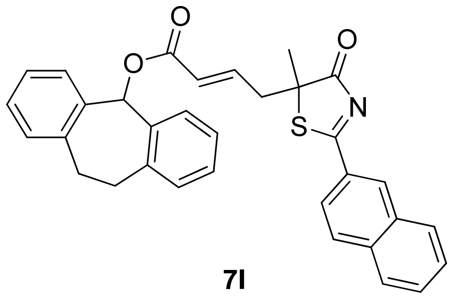

7l

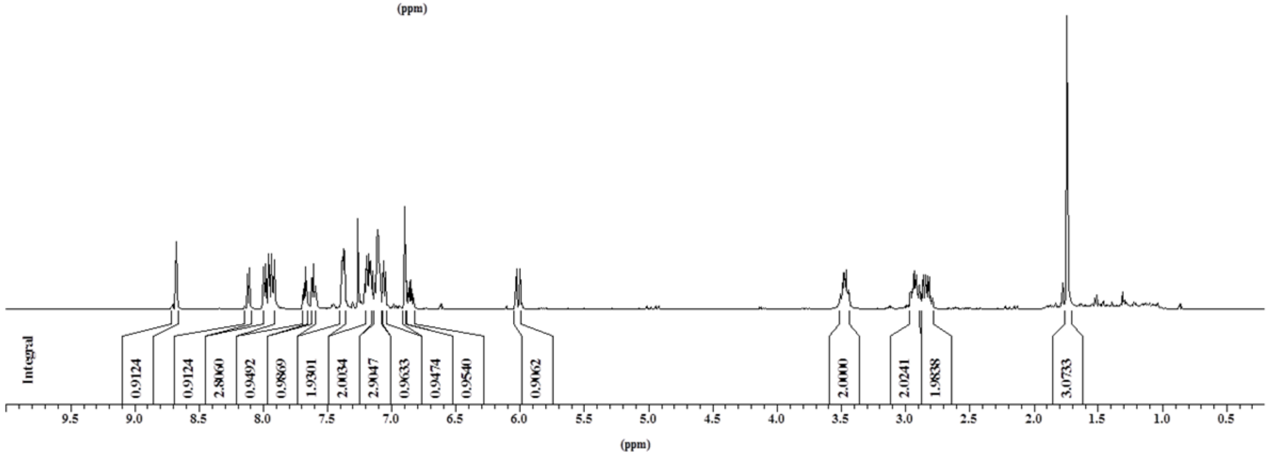

*** Current Data Parameters ***

NAME	:	wtl-0607
EXPNO	:	3
PROCNO	:	1

*** Acquisition Parameters ***

LOCNUC	:	2H
NS	:	25
NUCLEUS	:	off
O1	:	3088.51 Hz
PULPROG	:	zg30
SFO1	:	500.1330885 MHz
SOLVENT	:	CDCl3
SW	:	20.6557 ppm
TD	:	32768
TE	:	296.3 K

*** Processing Parameters ***

LB	:	0.30 Hz
SF	:	500.1300134 MHz

*** 1D NMR Plot Parameters ***

NUCLEUS	:	off

13C AMX500

wtl-887

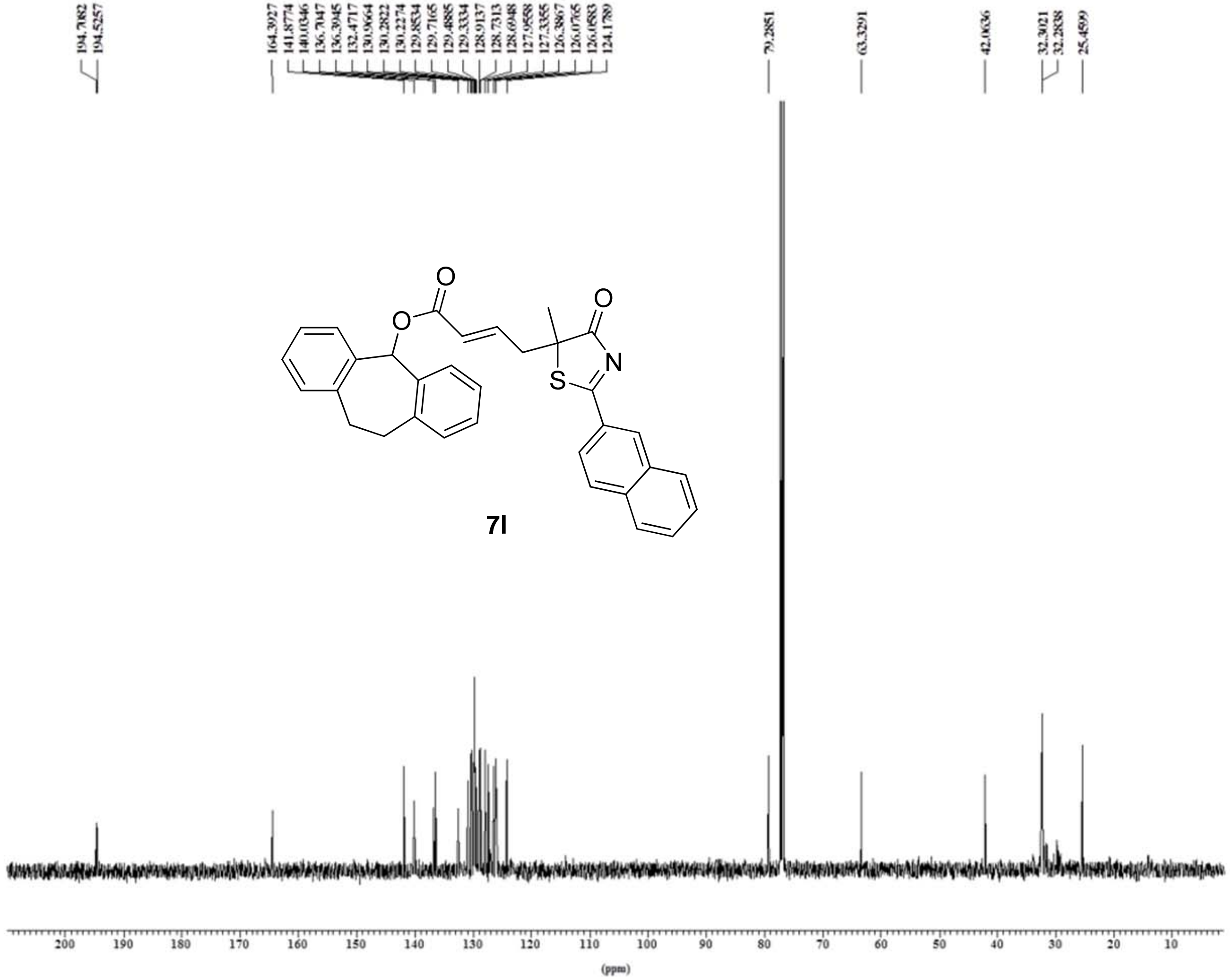

7l

*** Current Data Parameters ***

NAME	:	wtl-0607
EXPNO	:	4
PROCNO	:	1

*** Acquisition Parameters ***

LOCNUC	:	2H
NS	:	530
NUCLEUS	:	off
O1	:	18863.67 Hz
PULPROG	:	zgpg30
SFO1	:	125.7766527 MHz
SOLVENT	:	CDCl3
SW	:	298.8948 ppm
TD	:	65536
TE	:	296.3 K

*** Processing Parameters ***

LB	:	1.00 Hz
SF	:	125.7577928 MHz

*** 1D NMR Plot Parameters ***

NUCLEUS	:	off

1H AMX500

wtl-923 R

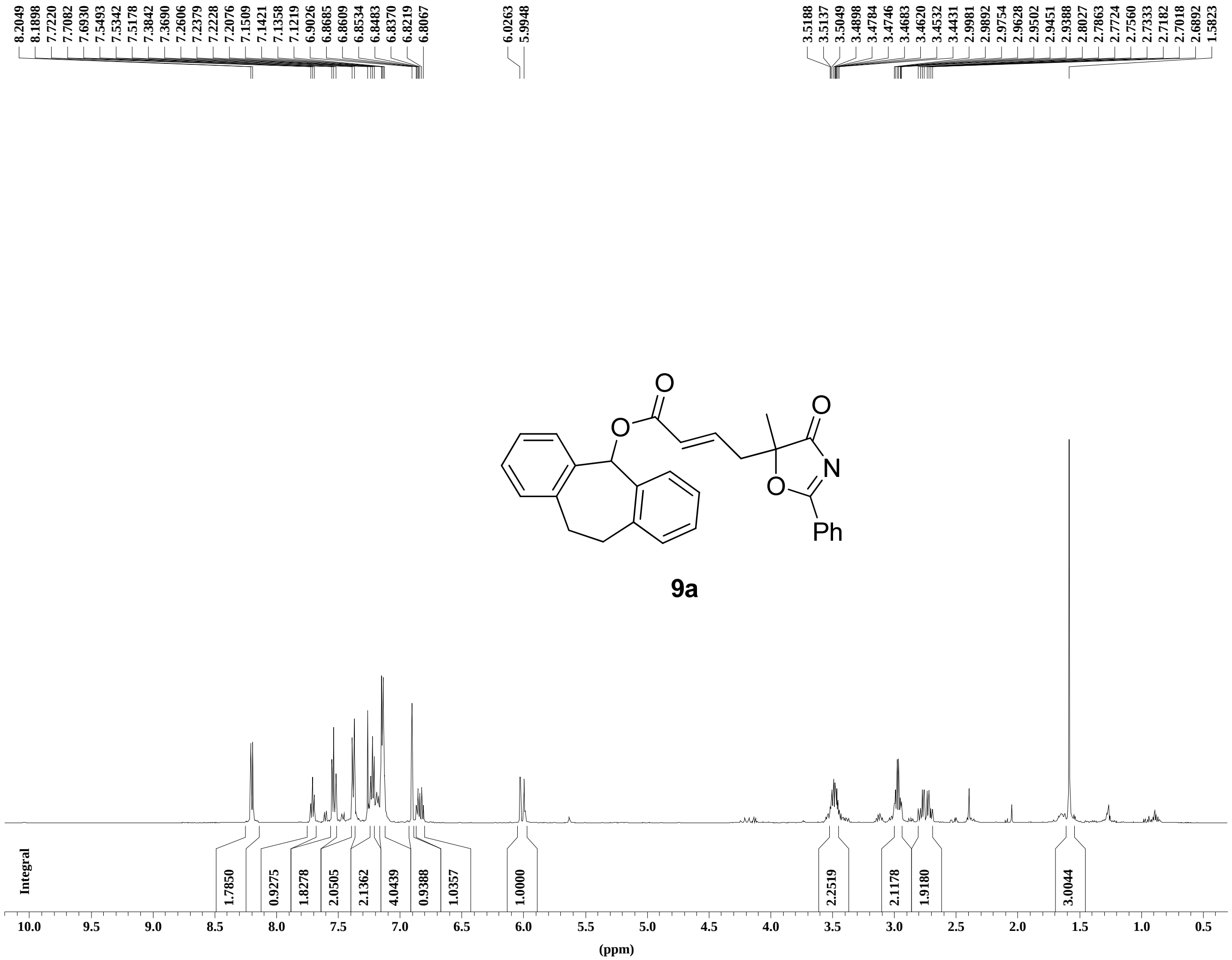

9a

***** Current Data Parameters *****

**NAME**	**:**	**wtl-0423**
**EXPNO**	**:**	**5**
**PROCNO**	**:**	**1**

***** Acquisition Parameters *****

**LOCNUC**	**:**	**2H**
**NS**	**:**	**79**
**NUCLEUS**	**:**	**off**
**O1**	**:**	**3088.51 Hz**
**PULPROG**	**:**	**zg30**
**SFO1**	**:**	**500.1330885 MHz**
**SOLVENT**	**:**	**CDCl3**
**SW**	**:**	**20.6557 ppm**
**TD**	**:**	**32768**
**TE**	**:**	**297.2 K**

***** Processing Parameters *****

**LB**	**:**	**0.30 Hz**
**SF**	**:**	**500.1300134 MHz**

***** 1D NMR Plot Parameters *****

**NUCLEUS**	**:**	**off**

13C AMX500

wtl-923 R

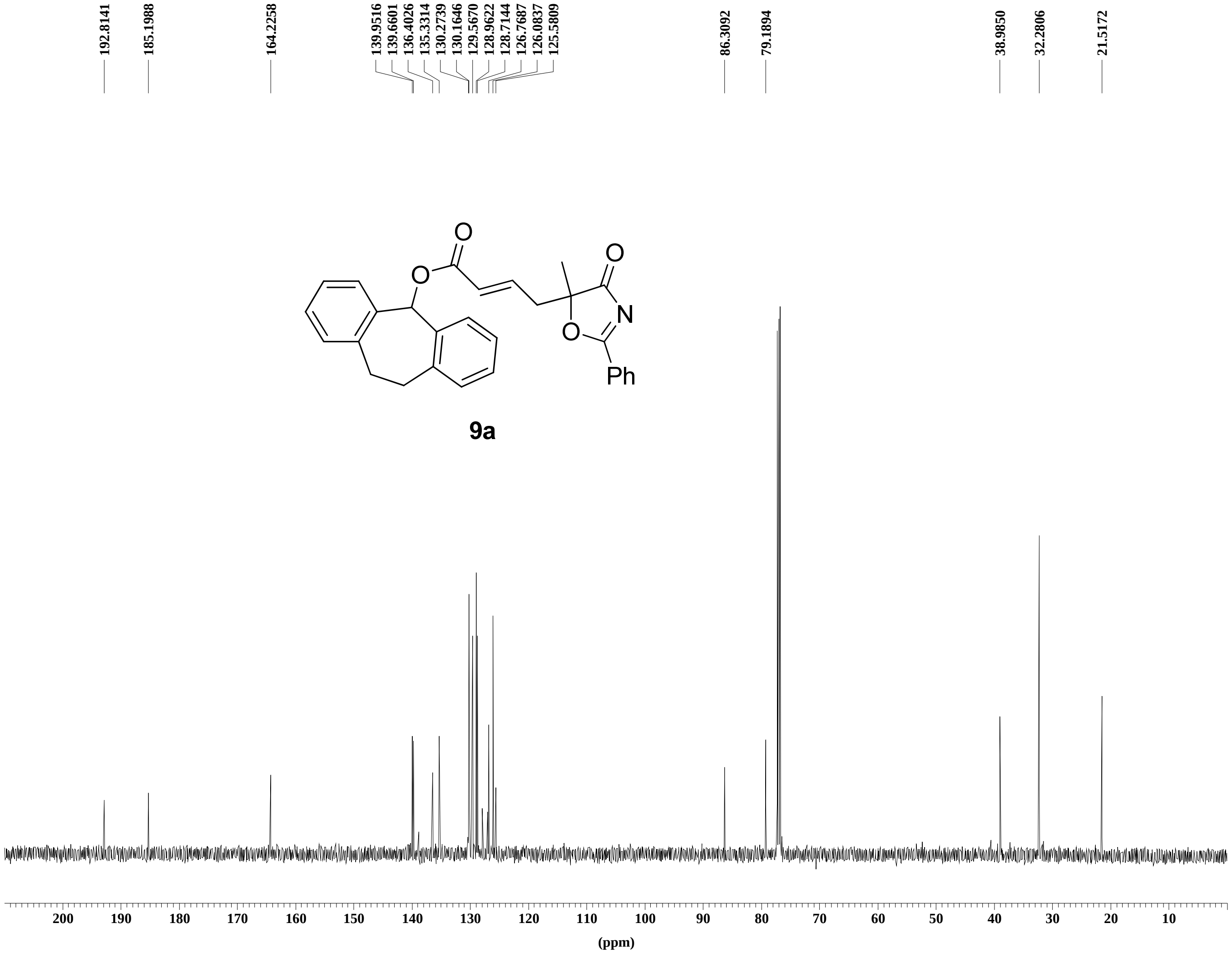

9a

***** Current Data Parameters *****

NAME	:	wtl-0423
EXPNO	:	6
PROCNO	:	1

***** Acquisition Parameters *****

LOCNUC	:	2H
NS	:	218
NUCLEUS	:	off
O1	:	13204.57 Hz
PULPROG	:	zgpg30
SFO1	:	125.7709936 MHz
SOLVENT	:	CDCl3
SW	:	238.7675 ppm
TD	:	65536
TE	:	297.2 K

***** Processing Parameters *****

LB	:	1.00 Hz
SF	:	125.7577952 MHz

***** 1D NMR Plot Parameters *****

NUCLEUS	:	off

1H AMX500

wtl-919 R

*** Current Data Parameters ***

NAME	:	wtl-0610
EXPNO	:	1
PROCNO	:	1

*** Acquisition Parameters ***

LOCNUC	:	2H
NS	:	32
NUCLEUS	:	off
O1	:	3088.51 Hz
PULPROG	:	zg30
SFO1	:	500.1330885 MHz
SOLVENT	:	CDCl3
SW	:	20.6557 ppm
TD	:	32768
TE	:	296.5 K

*** Processing Parameters ***

LB	:	0.30 Hz
SF	:	500.1300134 MHz

*** 1D NMR Plot Parameters ***

NUCLEUS	:	off

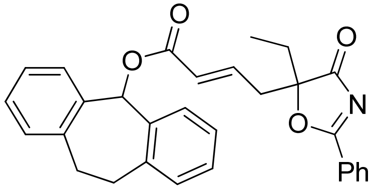

9b

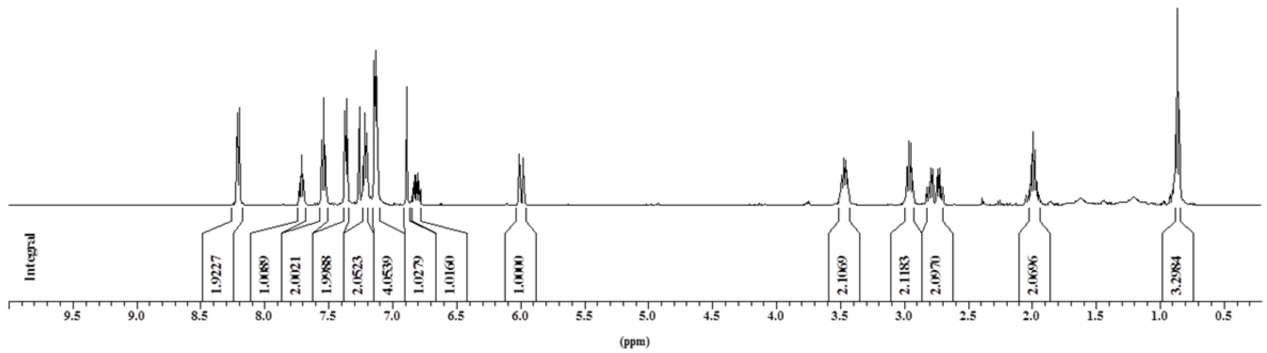

13C AMX500

wtl-919 R

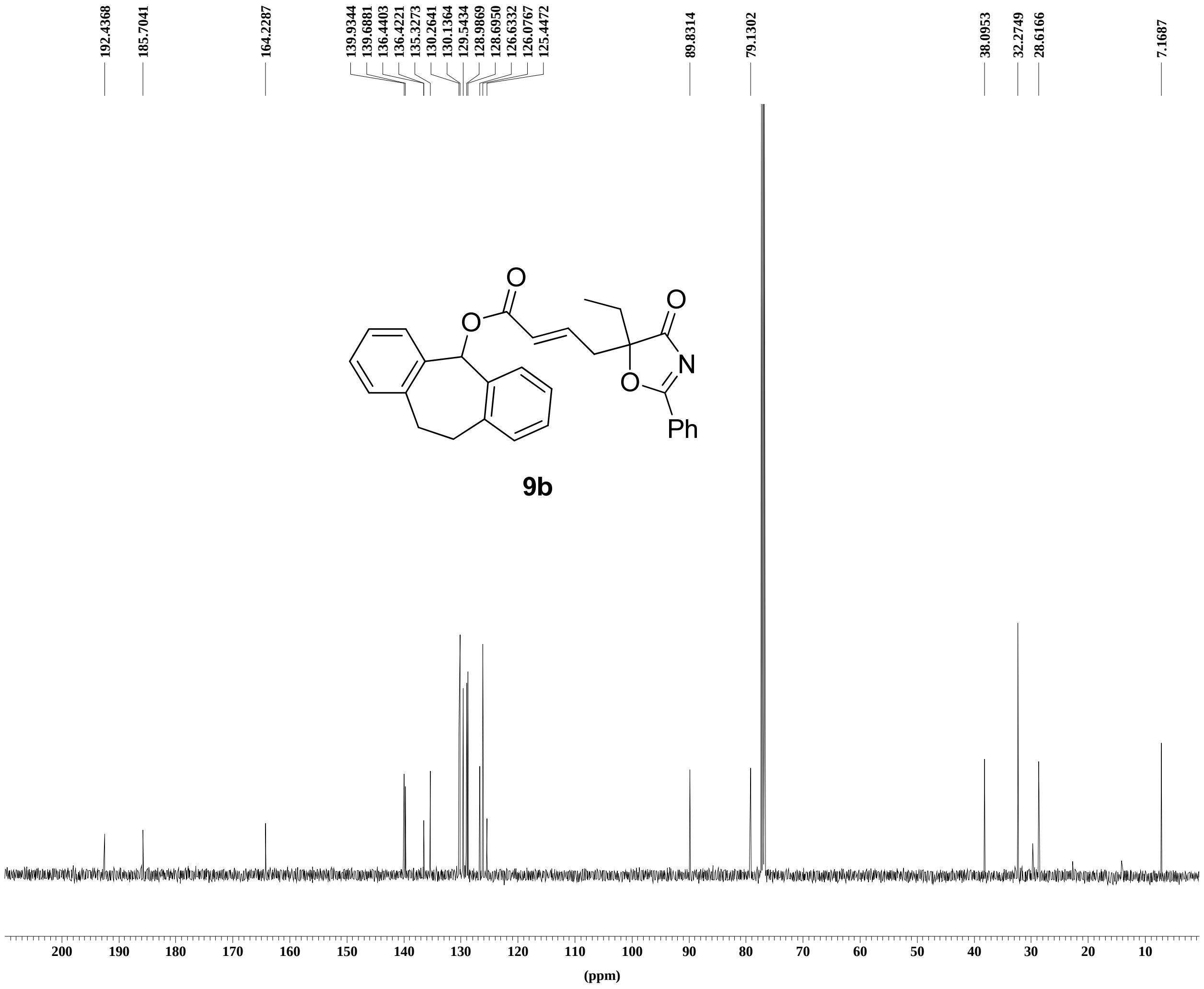

*** Current Data Parameters ***

NAME	:	wtl-0610
EXPNO	:	2
PROCNO	:	1

*** Acquisition Parameters ***

LOCNUC	:	2H
NS	:	337
NUCLEUS	:	off
O1	:	18863.67 Hz
PULPROG	:	zgpg30
SFO1	:	125.7766527 MHz
SOLVENT	:	CDCl3
SW	:	298.8948 ppm
TD	:	65536
TE	:	297.0 K

*** Processing Parameters ***

LB	:	1.00 Hz
SF	:	125.7577940 MHz

*** 1D NMR Plot Parameters ***

NUCLEUS	:	off

1H AMX500

wtl-947 R

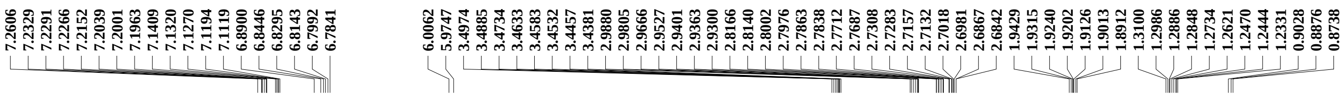

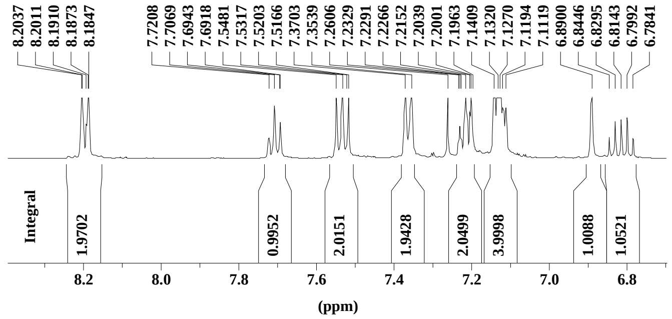

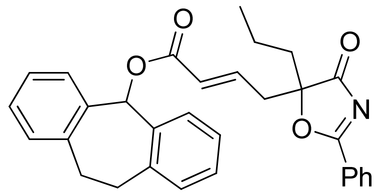

9c

*** Current Data Parameters ***

NAME	:	wtl-0519
EXPNO	:	3
PROCNO	:	1

*** Acquisition Parameters ***

LOCNUC	:	2H
NS	:	37
NUCLEUS	:	off
O1	:	3088.51 Hz
PULPROG	:	zg30
SFO1	:	500.1330885 MHz
SOLVENT	:	CDCl3
SW	:	20.6557 ppm
TD	:	32768
TE	:	300.0 K

*** Processing Parameters ***

LB	:	0.30 Hz
SF	:	500.1300134 MHz

*** 1D NMR Plot Parameters ***

NUCLEUS	:	off

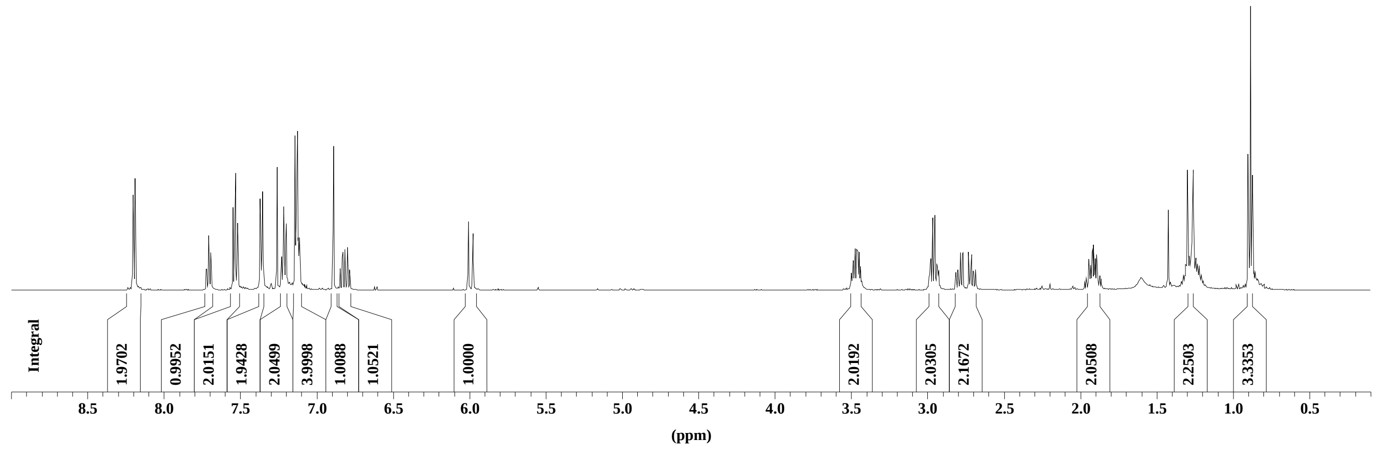

13C AMX500

wtl-947 R

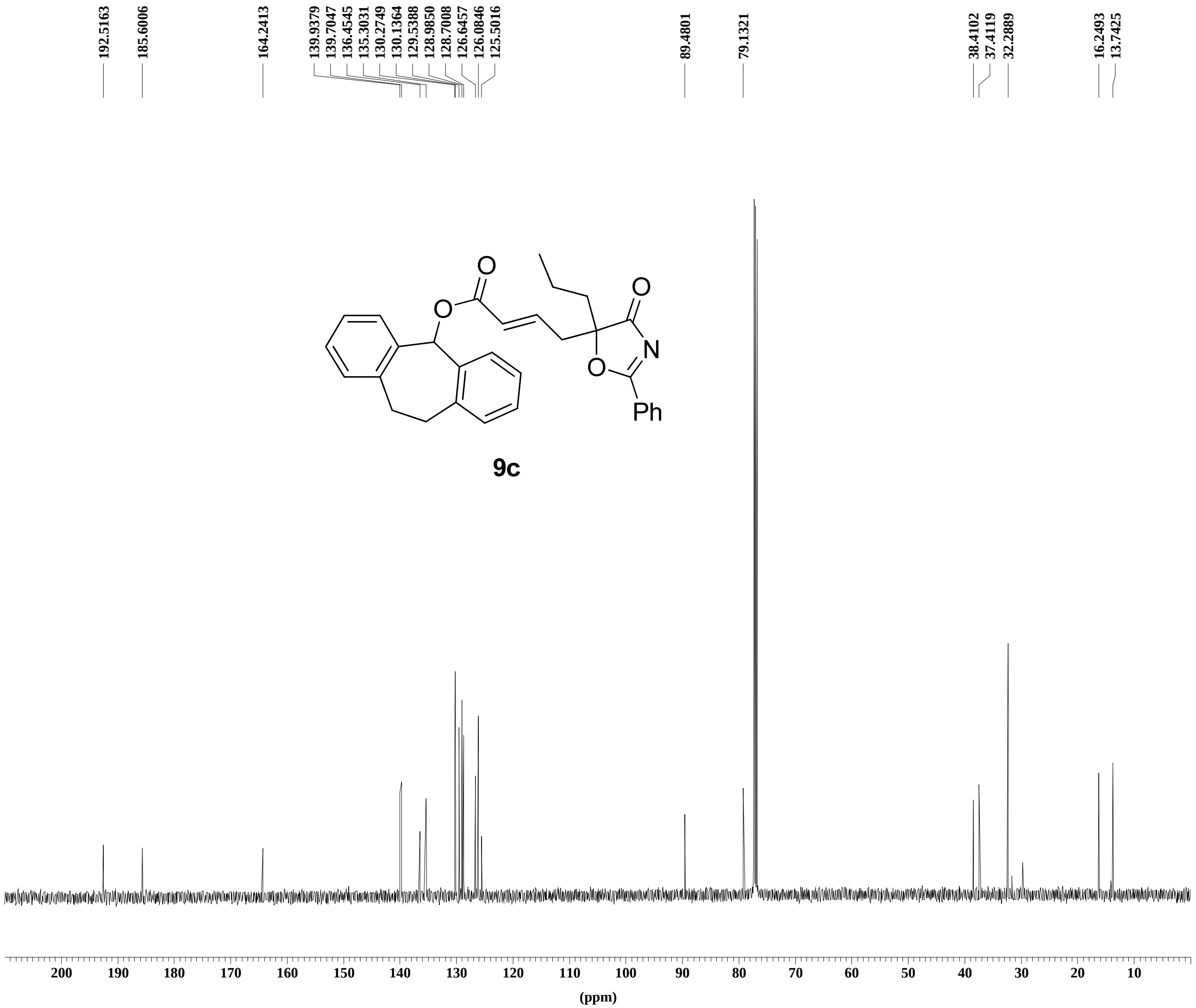

*** Current Data Parameters ***

NAME	:	wtl-0519
EXPNO	:	4
PROCNO	:	1

*** Acquisition Parameters ***

LOCNUC	:	2H
NS	:	414
NUCLEUS	:	off
O1	:	13204.57 Hz
PULPROG	:	zgpg30
SFO1	:	125.7709936 MHz
SOLVENT	:	CDCl3
SW	:	238.7675 ppm
TD	:	65536
TE	:	300.2 K

*** Processing Parameters ***

LB	:	1.00 Hz
SF	:	125.7577924 MHz

*** 1D NMR Plot Parameters ***

NUCLEUS	:	off

1H AMX500

wtl-920 R

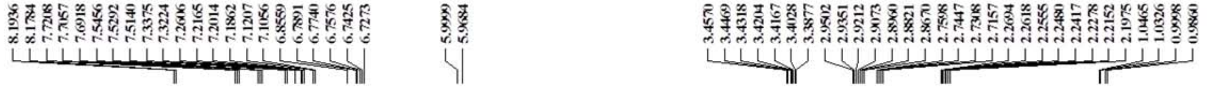

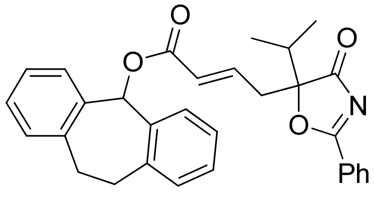

9d

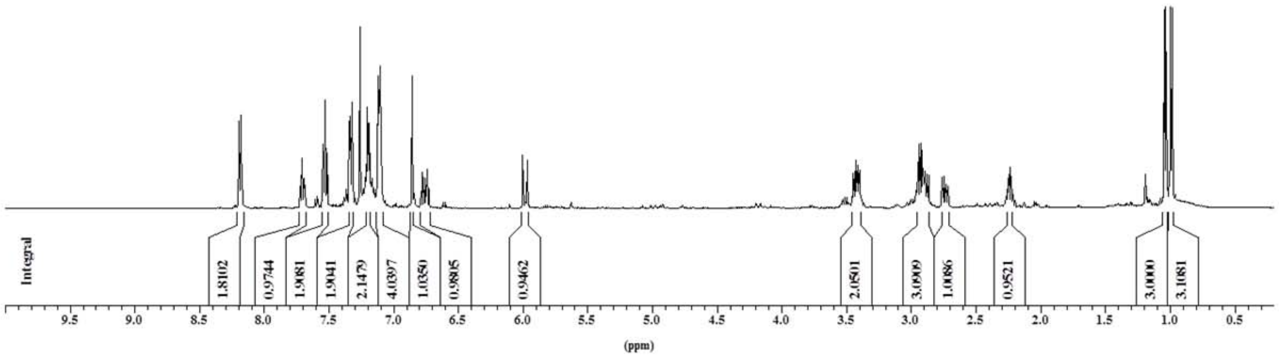

*** Current Data Parameters ***

NAME : wtl-0610

EXPNO : 3

PROCNO : 1

*** Acquisition Parameters ***

LOCNUC : 2H

NS : 31

NUCLEUS : off

O1 : 3088.51 Hz

PULPROG : zg30

SFO1 : 500.1330885 MHz

SOLVENT : CDCl3

SW : 20.6557 ppm

TD : 32768

TE : 296.5 K

*** Processing Parameters ***

LB : 0.30 Hz

SF : 500.1300140 MHz

*** 1D NMR Plot Parameters ***

NUCLEUS : off

13C AMX500

wtl-920 R

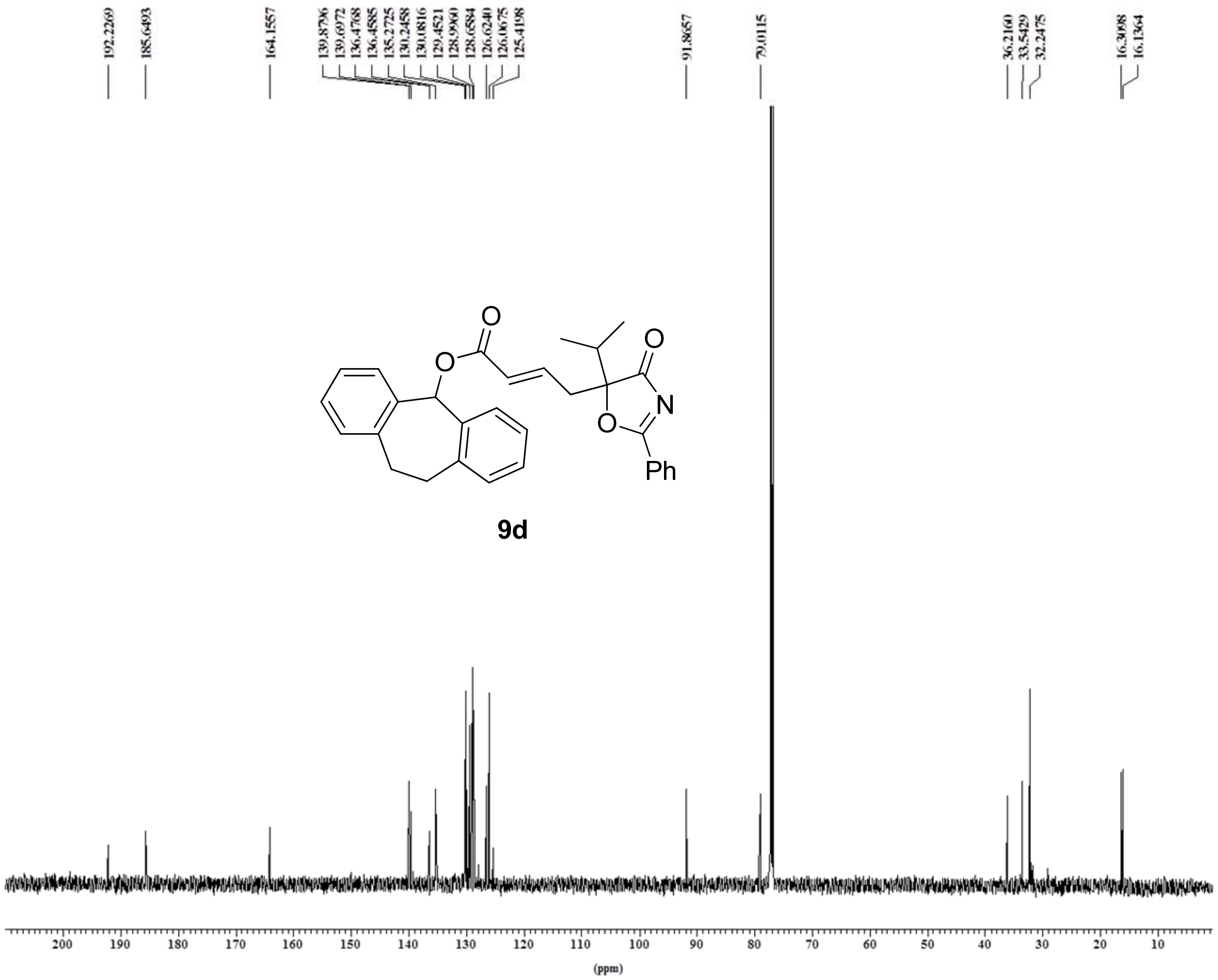

9d

*** Current Data Parameters ***

NAME : wtl-0610

EXPNO : 4

PROCNO : 1

*** Acquisition Parameters ***

LOCNUC : 2H

NS : 784

NUCLEUS : off

O1 : 18863.67 Hz

PULPROG : zgpg30

SFO1 : 125.7766527 MHz

SOLVENT : CDCl3

SW : 298.8948 ppm

TD : 65536

TE : 296.4 K

*** Processing Parameters ***

LB : 1.00 Hz

SF : 125.7577917 MHz

*** 1D NMR Plot Parameters ***

NUCLEUS : off

1H AMX500

wtl-948 R

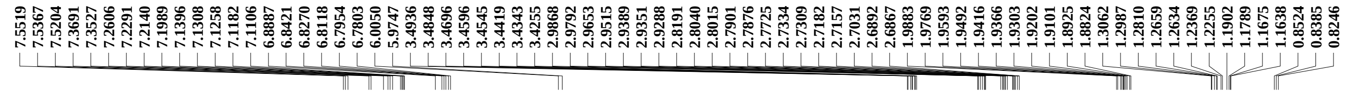

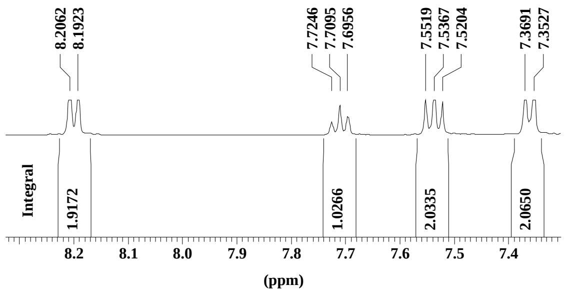

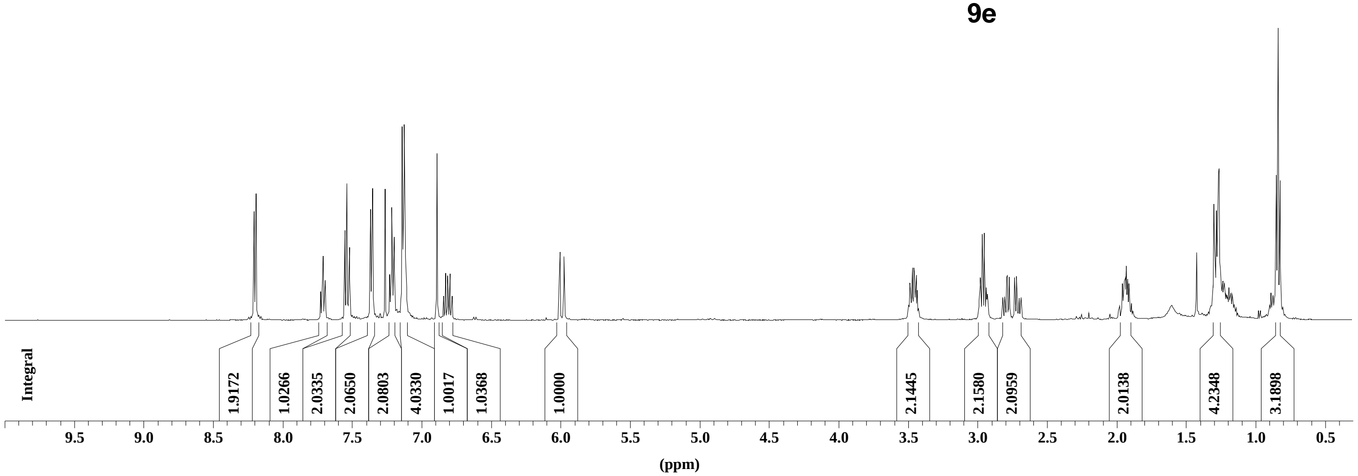

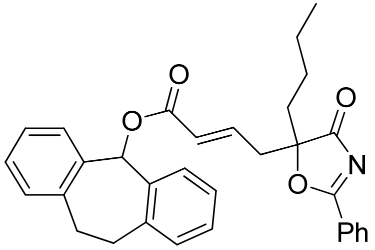

9e

***** Current Data Parameters *****

NAME	:	wtl-0519
EXPNO	:	5
PROCNO	:	1

***** Acquisition Parameters *****

LOCNUC	:	2H
NS	:	28
NUCLEUS	:	off
O1	:	3088.51 Hz
PULPROG	:	zg30
SFO1	:	500.1330885 MHz
SOLVENT	:	CDCl3
SW	:	20.6557 ppm
TD	:	32768
TE	:	300.0 K

***** Processing Parameters *****

LB	:	0.30 Hz
SF	:	500.1300134 MHz

***** 1D NMR Plot Parameters *****

NUCLEUS	:	off

13C AMX500

wtl-948 R

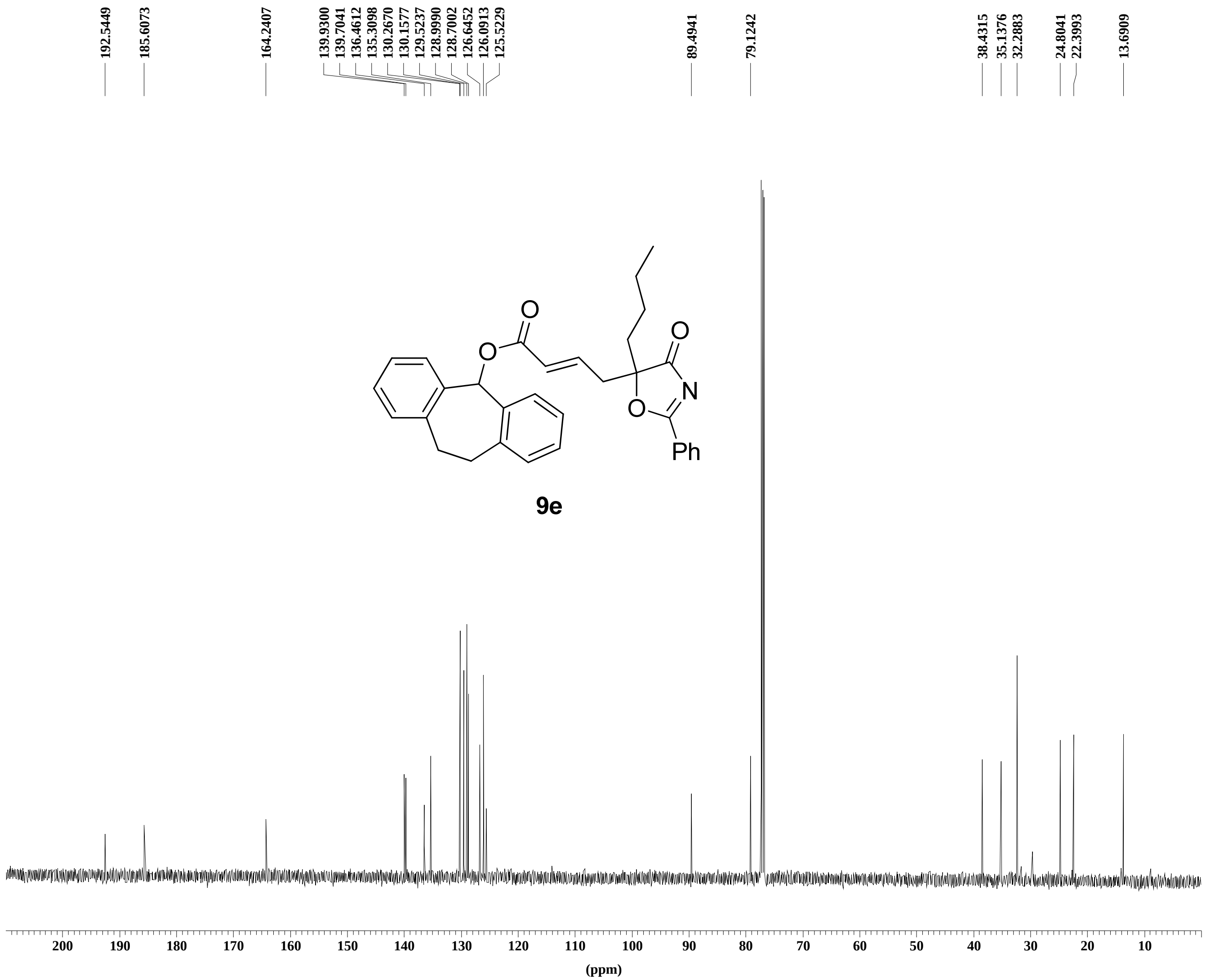

*** Current Data Parameters ***

NAME	:	wtl-0519
EXPNO	:	6
PROCNO	:	1

*** Acquisition Parameters ***

LOCNUC	:	2H
NS	:	382
NUCLEUS	:	off
O1	:	13204.57 Hz
PULPROG	:	zgpg30
SFO1	:	125.7709936 MHz
SOLVENT	:	CDCl3
SW	:	238.7675 ppm
TD	:	65536
TE	:	300.0 K

*** Processing Parameters ***

LB	:	1.00 Hz
SF	:	125.7577915 MHz

*** 1D NMR Plot Parameters ***

NUCLEUS	:	off

1H AMX500

wtl-921 R

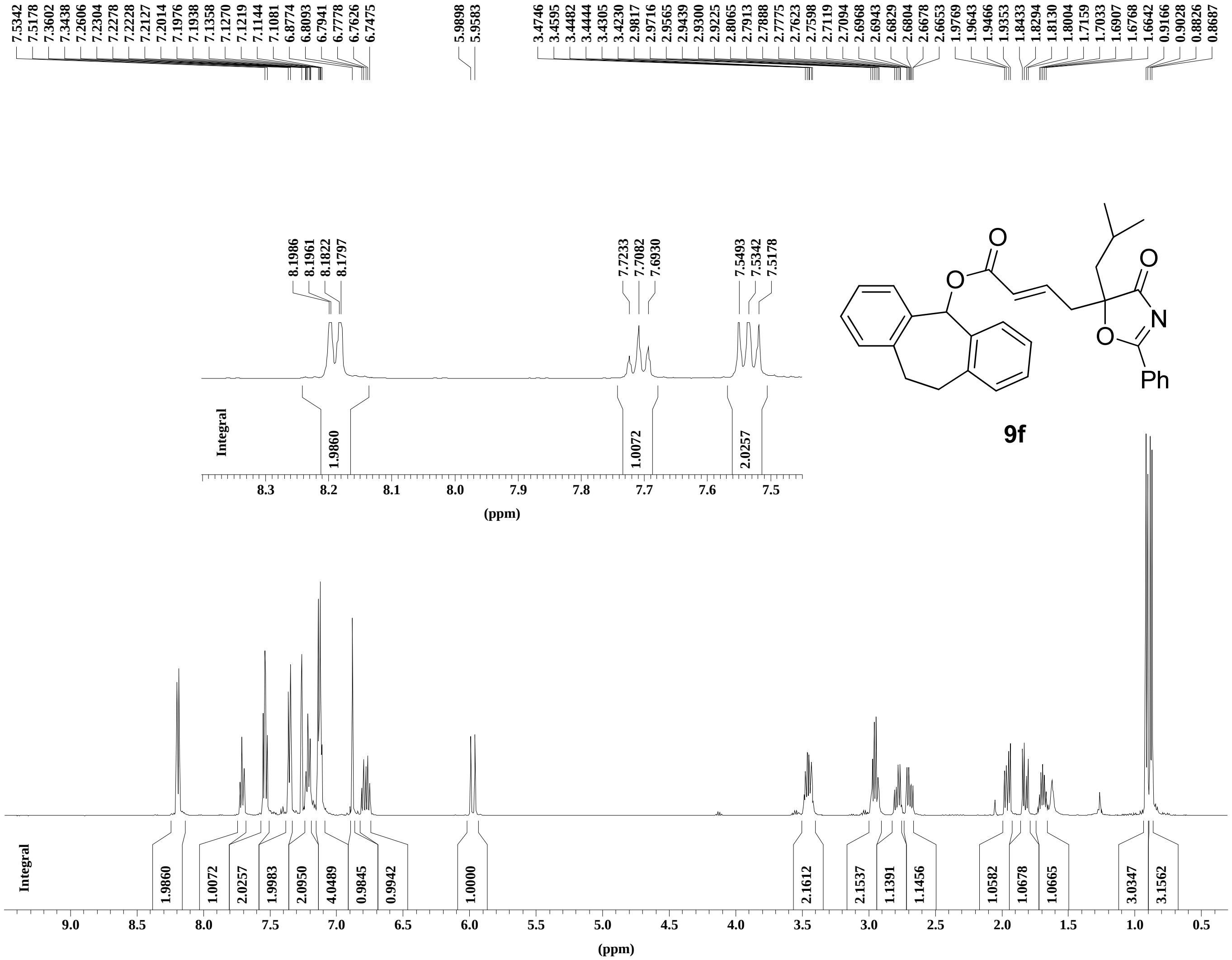

*** Current Data Parameters ***

NAME	:	wtl-0423
EXPNO	:	9
PROCNO	:	1

*** Acquisition Parameters ***

LOCNUC	:	2H
NS	:	44
NUCLEUS	:	off
O1	:	3088.51 Hz
PULPROG	:	zg30
SFO1	:	500.1330885 MHz
SOLVENT	:	CDCl3
SW	:	20.6557 ppm
TD	:	32768
TE	:	297.6 K

*** Processing Parameters ***

LB	:	0.30 Hz
SF	:	500.1300134 MHz

*** 1D NMR Plot Parameters ***

NUCLEUS	:	off

13C AMX500

wtl-921 R

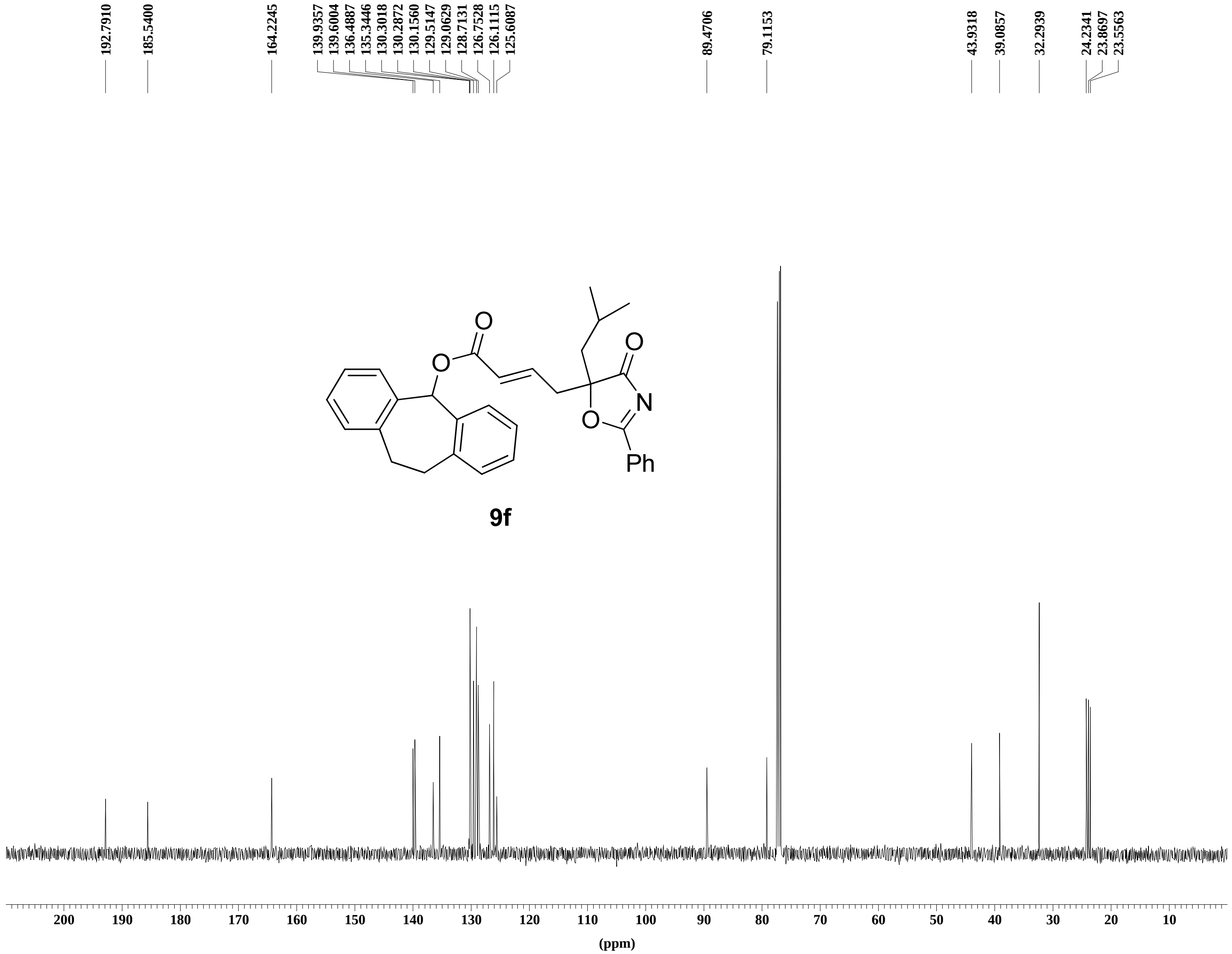

9f

*** Current Data Parameters ***

NAME	:	wtl-0423
EXPNO	:	10
PROCNO	:	1

*** Acquisition Parameters ***

LOCNUC	:	2H
NS	:	284
NUCLEUS	:	off
O1	:	13204.57 Hz
PULPROG	:	zgpg30
SFO1	:	125.7709936 MHz
SOLVENT	:	CDCl3
SW	:	238.7675 ppm
TD	:	65536
TE	:	297.6 K

*** Processing Parameters ***

LB	:	1.00 Hz
SF	:	125.7577890 MHz

*** 1D NMR Plot Parameters ***

NUCLEUS	:	off

1H AMX500

wtl-925R-b

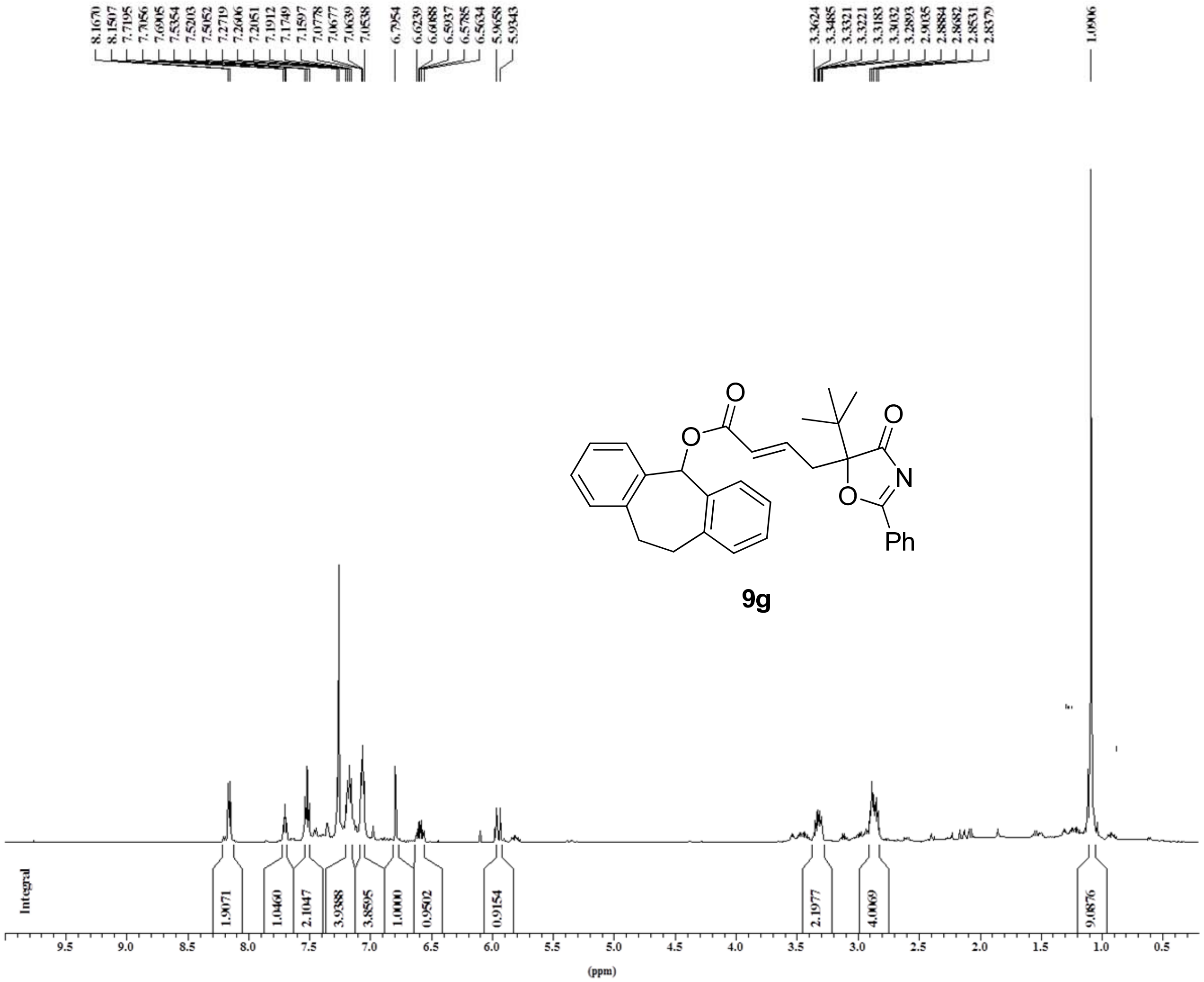

9g

*** Current Data Parameters ***

NAME	:	wtl-0616
EXPNO	:	3
PROCNO	:	1

*** Acquisition Parameters ***

LOCNUC	:	2H
NS	:	118
NUCLEUS	:	off
O1	:	3088.51 Hz
PULPROG	:	zg30
SFO1	:	500.1330885 MHz
SOLVENT	:	CDCl3
SW	:	20.6557 ppm
TD	:	32768
TE	:	293.8 K

*** Processing Parameters ***

LB	:	0.30 Hz
SF	:	500.1300140 MHz

*** 1D NMR Plot Parameters ***

NUCLEUS	:	off

13C AMX500

wtl-925R-b

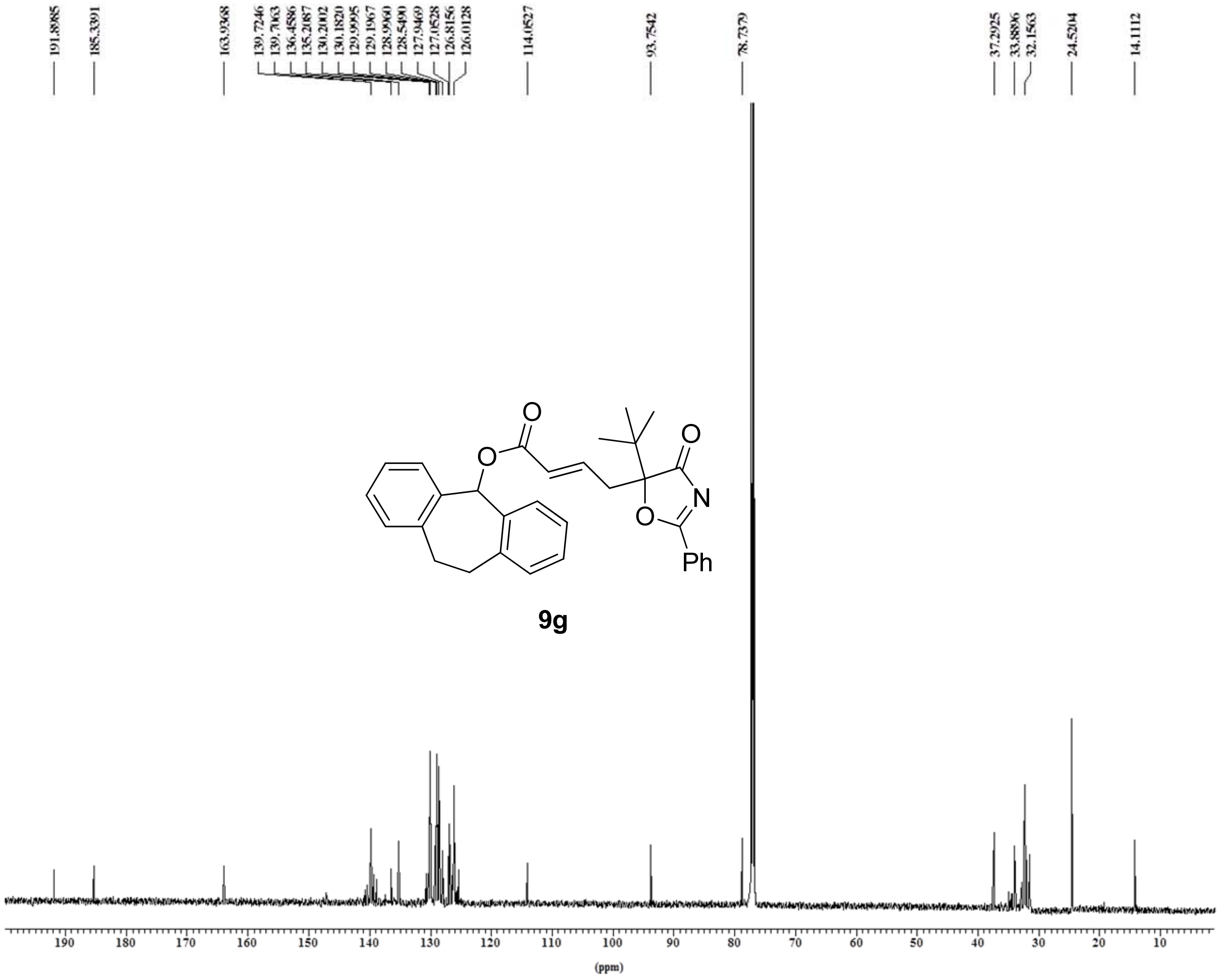

9g

*** Current Data Parameters ***

NAME	:	wtl-0616
EXPNO	:	12
PROCNO	:	1

*** Acquisition Parameters ***

LOCNUC	:	2H
NS	:	17040
NUCLEUS	:	off
O1	:	18863.67 Hz
PULPROG	:	zgpg30
SFO1	:	125.7766527 MHz
SOLVENT	:	CDCl3
SW	:	298.8948 ppm
TD	:	65536
TE	:	293.8 K

*** Processing Parameters ***

LB	:	1.00 Hz
SF	:	125.7577928 MHz

*** 1D NMR Plot Parameters ***

NUCLEUS	:	off

1H AMX500

wtl-934 R

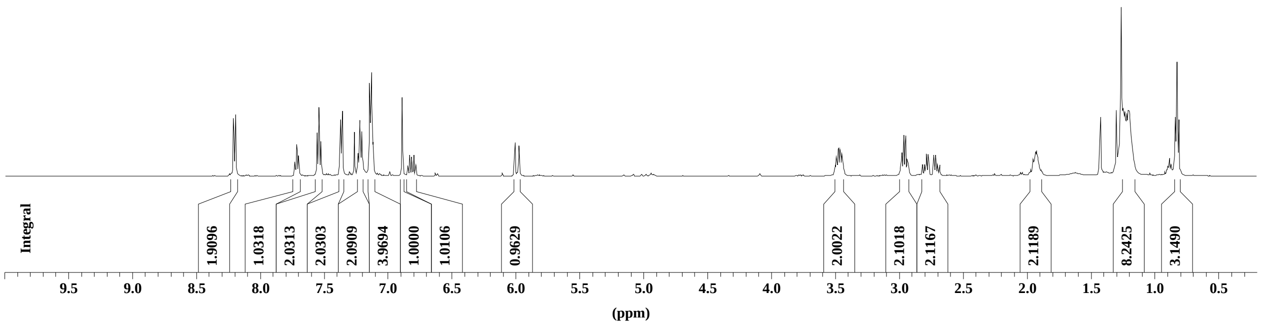

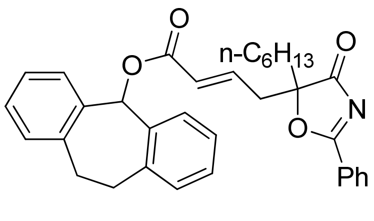

9h

*** Current Data Parameters ***
NAME : wtl-0610
EXPNO : 7
PROCNO : 1
*** Acquisition Parameters ***
LOCNUC : 2H
NS : 30
NUCLEUS : off
O1 : 3088.51 Hz
PULPROG : zg30
SFO1 : 500.1330885 MHz
SOLVENT : CDCl3
SW : 20.6557 ppm
TD : 32768
TE : 295.3 K
*** Processing Parameters ***
LB : 0.30 Hz
SF : 500.1300134 MHz
*** 1D NMR Plot Parameters ***
NUCLEUS : off

13C AMX500

wtl-934 R

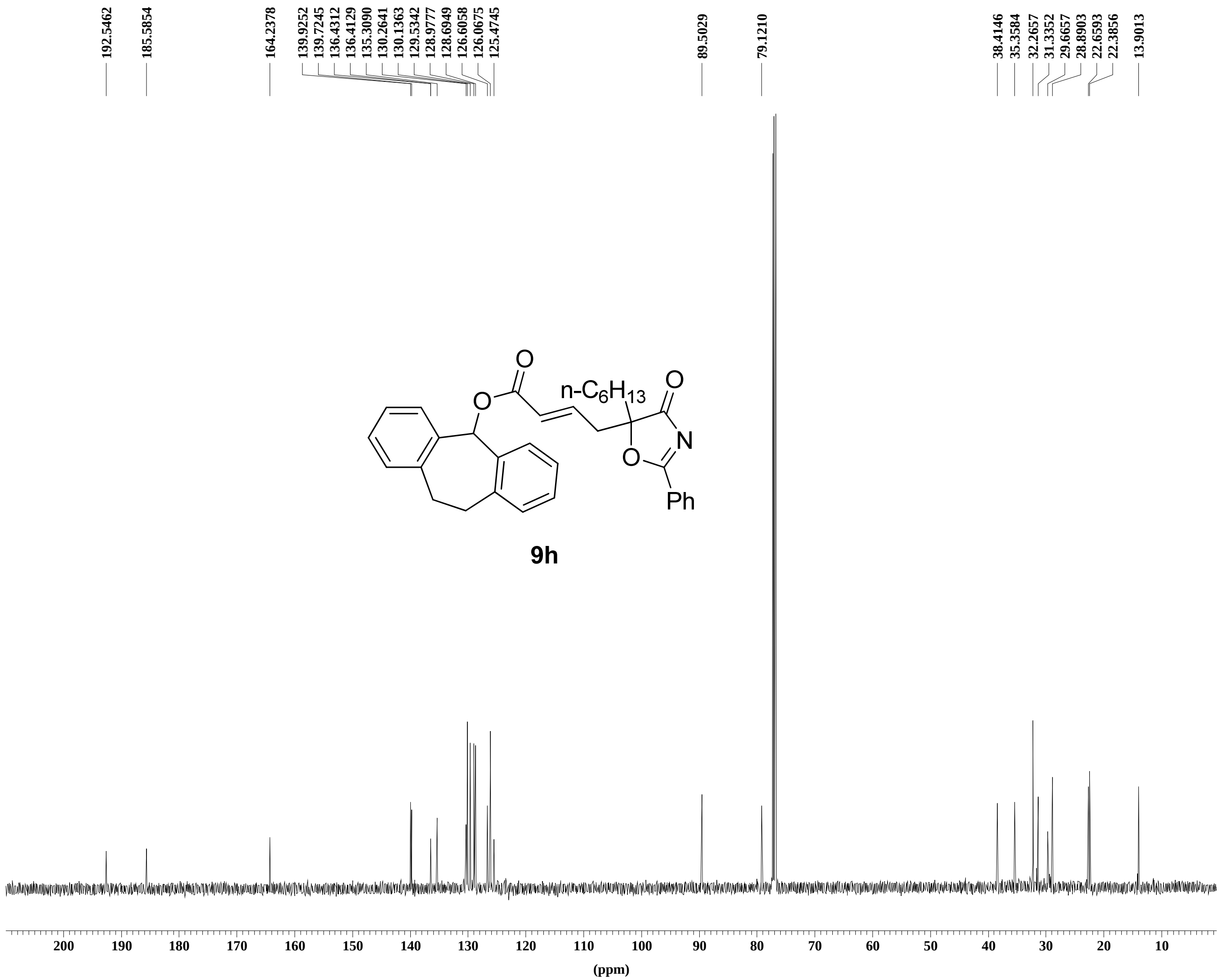

*** Current Data Parameters ***

NAME	:	wtl-0610
EXPNO	:	8
PROCNO	:	1

*** Acquisition Parameters ***

LOCNUC	:	2H
NS	:	285
NUCLEUS	:	off
O1	:	18863.67 Hz
PULPROG	:	zgpg30
SFO1	:	125.7766527 MHz
SOLVENT	:	CDCl3
SW	:	298.8948 ppm
TD	:	65536
TE	:	295.4 K

*** Processing Parameters ***

LB	:	1.00 Hz
SF	:	125.7577951 MHz

*** 1D NMR Plot Parameters ***

NUCLEUS	:	off

1H AMX500

wtl-927 R

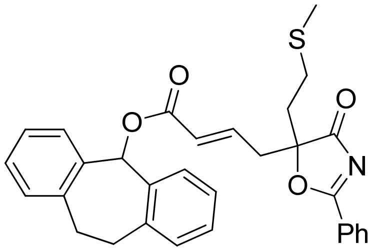

9i

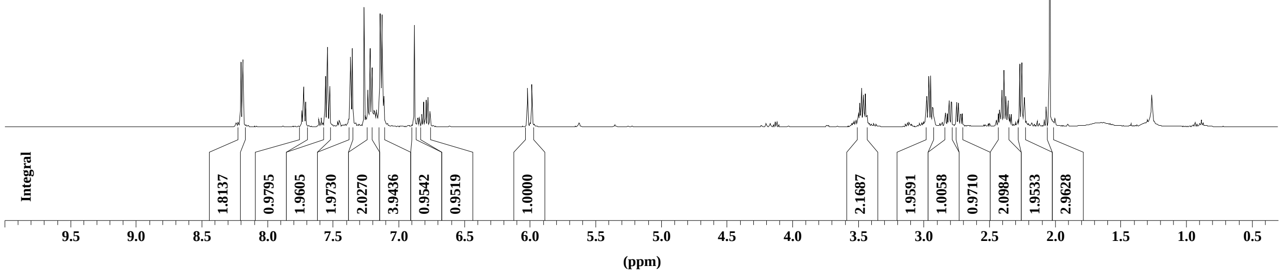

***** Current Data Parameters *****

NAME	:	wtl-0501
EXPNO	:	3
PROCNO	:	1

***** Acquisition Parameters *****

LOCNUC	:	2H
NS	:	29
NUCLEUS	:	off
O1	:	3088.51 Hz
PULPROG	:	zg30
SFO1	:	500.1330885 MHz
SOLVENT	:	CDCl3
SW	:	20.6557 ppm
TD	:	32768
TE	:	295.7 K

***** Processing Parameters *****

LB	:	0.30 Hz
SF	:	500.1300134 MHz

***** 1D NMR Plot Parameters *****

NUCLEUS	:	off

13C AMX500

wtl-927 R

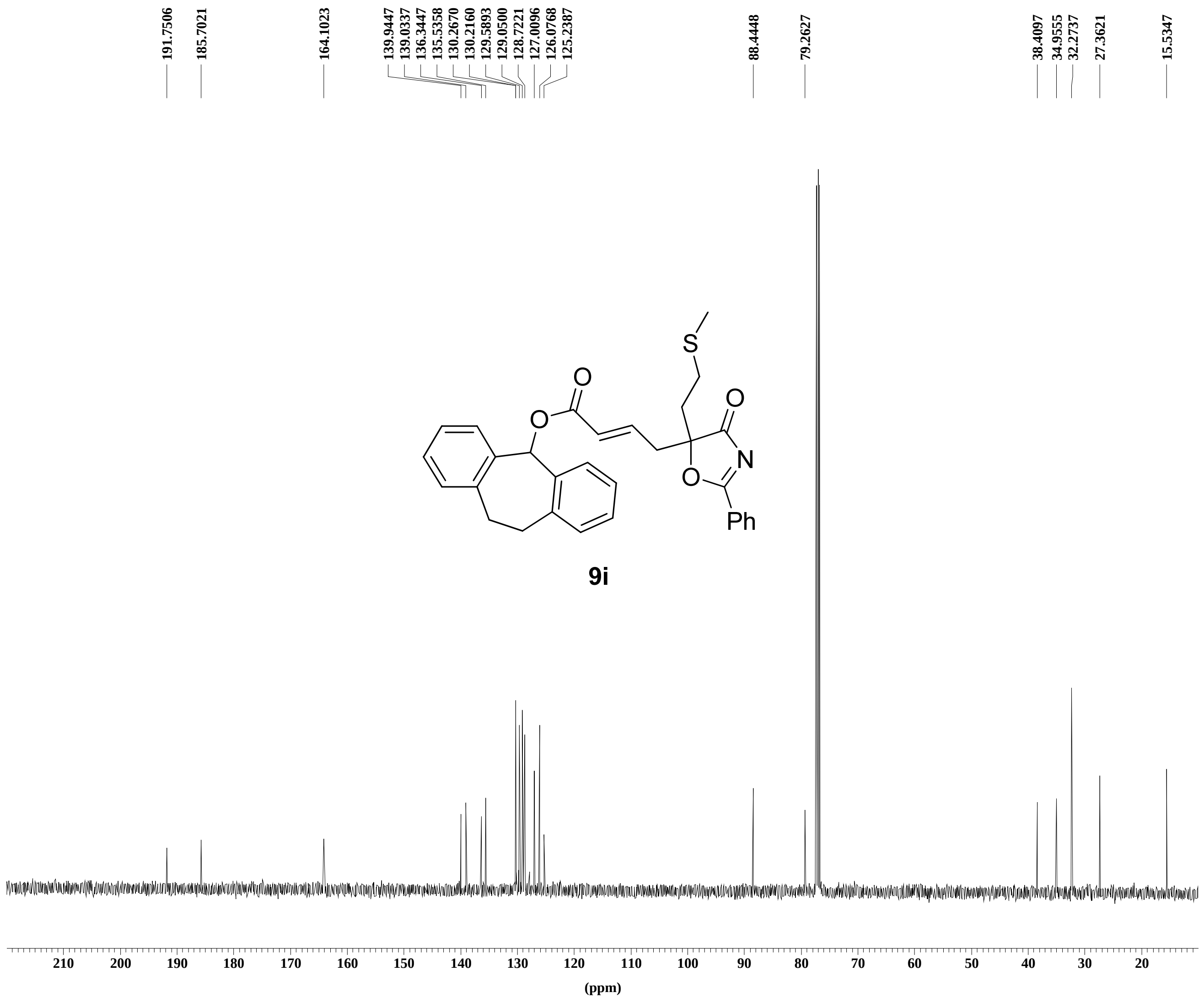

9i

*** Current Data Parameters ***

NAME	:	wtl-0501
EXPNO	:	4
PROCNO	:	1

*** Acquisition Parameters ***

LOCNUC	:	2H
NS	:	305
NUCLEUS	:	off
O1	:	13204.57 Hz
PULPROG	:	zgpg30
SFO1	:	125.7709936 MHz
SOLVENT	:	CDCl3
SW	:	238.7675 ppm
TD	:	65536
TE	:	295.9 K

*** Processing Parameters ***

LB	:	1.00 Hz
SF	:	125.7577952 MHz

*** 1D NMR Plot Parameters ***

NUCLEUS	:	off

1H AMX500

wtl-935 R

***** Current Data Parameters *****

NAME	:	wtl-0610
EXPNO	:	12
PROCNO	:	1

***** Acquisition Parameters *****

LOCNUC	:	2H
NS	:	17
NUCLEUS	:	off
O1	:	3088.51 Hz
PULPROG	:	zg30
SFO1	:	500.1330885 MHz
SOLVENT	:	CDCl3
SW	:	20.6557 ppm
TD	:	32768
TE	:	295.7 K

***** Processing Parameters *****

LB	:	0.30 Hz
SF	:	500.1300000 MHz

***** 1D NMR Plot Parameters *****

NUCLEUS	:	off

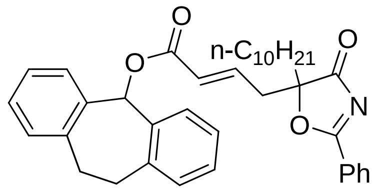

9j

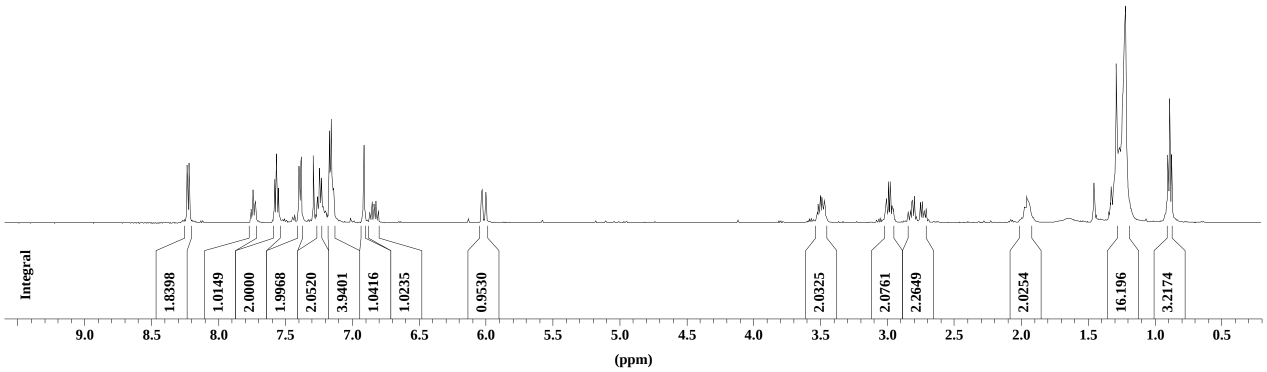

13C AMX500

wtl-935 R

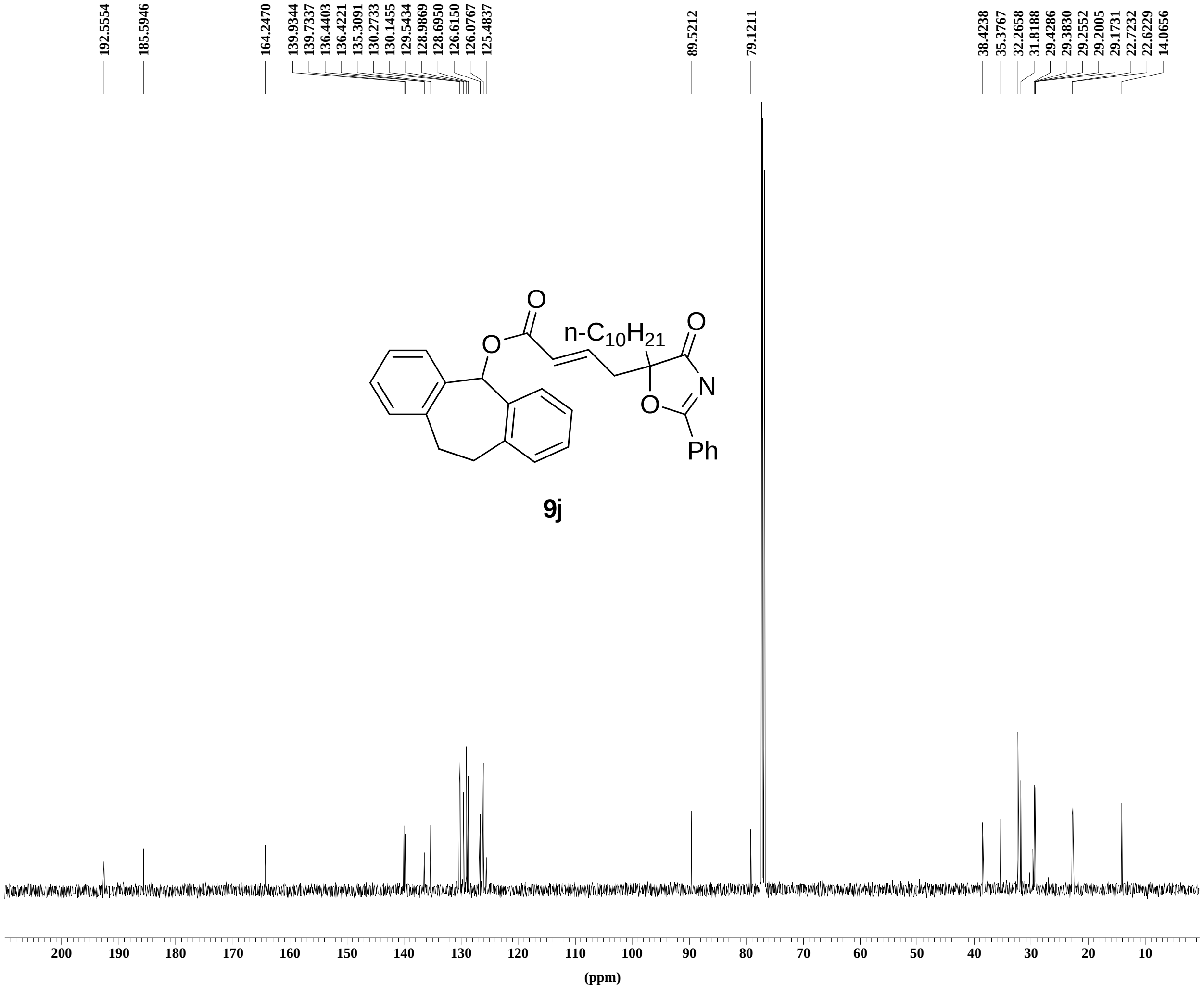

9j

*** Current Data Parameters ***

NAME : wtl-0610

EXPNO : 13

PROCNO : 1

*** Acquisition Parameters ***

LOCNUC : 2H

NS : 332

NUCLEUS : off

O1 : 18863.67 Hz

PULPROG : zgpg30

SFO1 : 125.7766527 MHz

SOLVENT : CDCl3

SW : 298.8948 ppm

TD : 65536

TE : 295.7 K

*** Processing Parameters ***

LB : 1.00 Hz

SF : 125.7577940 MHz

*** 1D NMR Plot Parameters ***

NUCLEUS : off

1H AMX500

wtl-922 R

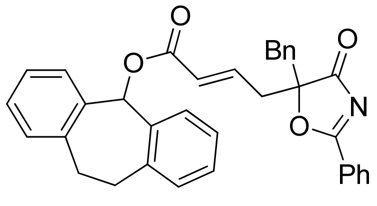

9k

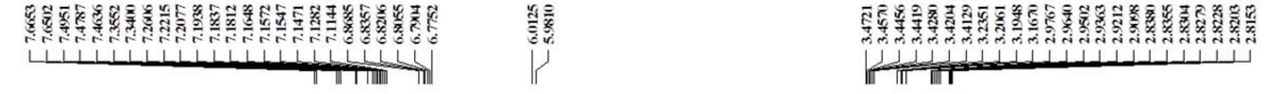

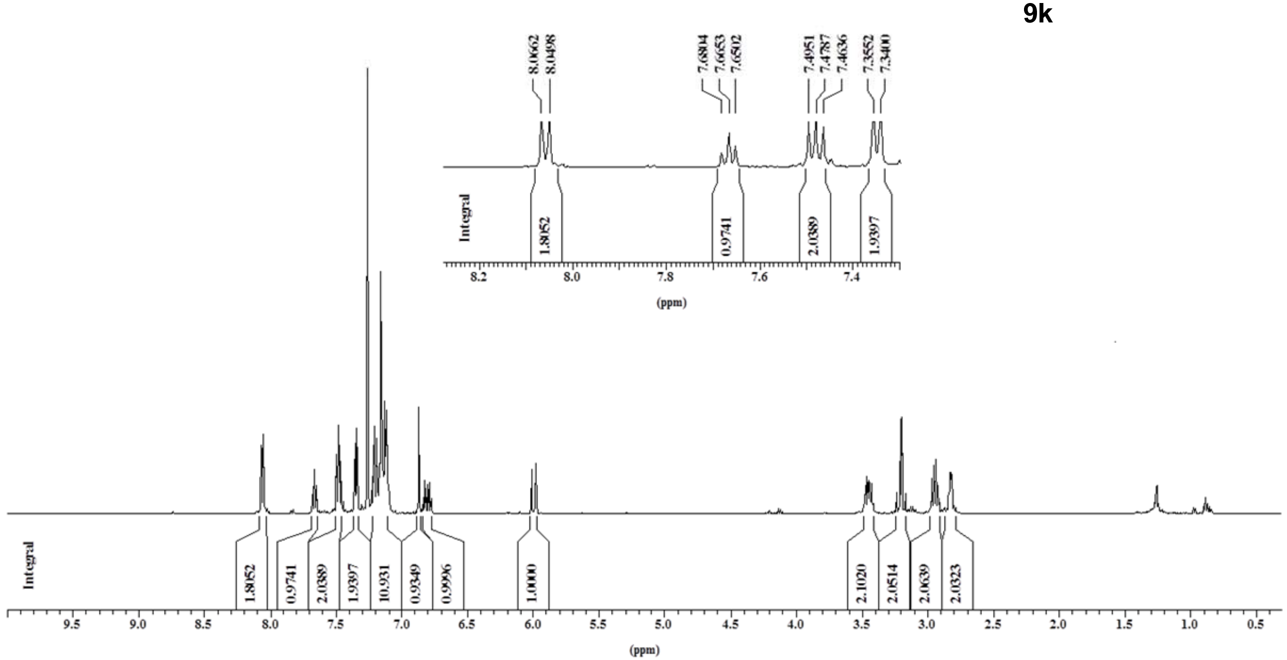

*** Current Data Parameters ***

NAME	:	wtl-0423
EXPNO	:	3
PROCNO	:	1

*** Acquisition Parameters ***

LOCNUC	:	2H
NS	:	67
NUCLEUS	:	off
O1	:	3088.51 Hz
PULPROG	:	zg30
SFO1	:	500.1330885 MHz
SOLVENT	:	CDCl3
SW	:	20.6557 ppm
TD	:	32768
TE	:	297.4 K

*** Processing Parameters ***

LB	:	0.30 Hz
SF	:	500.1300134 MHz

*** 1D NMR Plot Parameters ***

NUCLEUS	:	off

13C AMX500

wtl-922 R

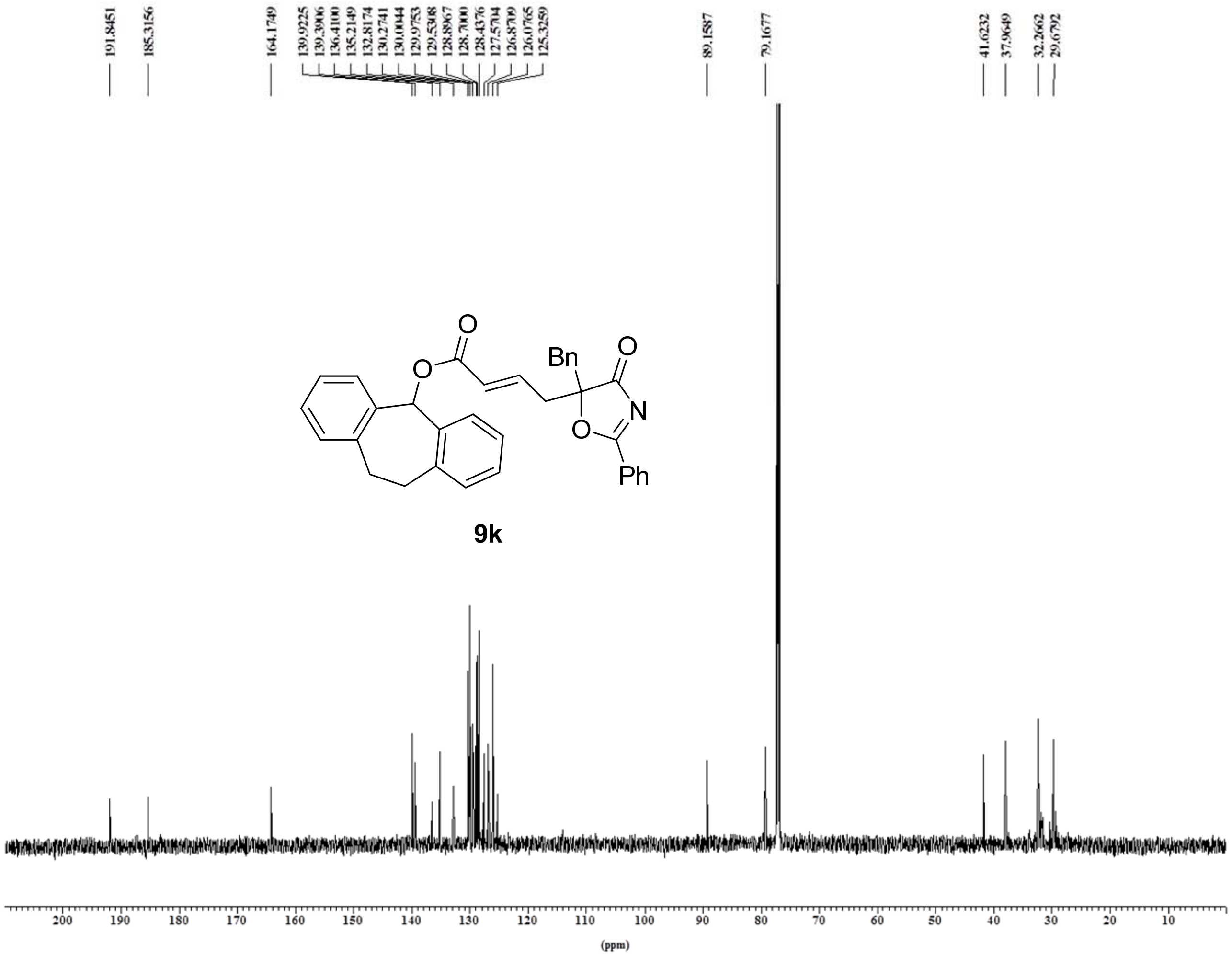

9k

*** Current Data Parameters ***

NAME	:	wtl-0423
EXPNO	:	4
PROCNO	:	1

*** Acquisition Parameters ***

LOCNUC	:	2H
NS	:	1531
NUCLEUS	:	off
O1	:	13204.57 Hz
PULPROG	:	zgpg30
SFO1	:	125.7709936 MHz
SOLVENT	:	CDCl3
SW	:	238.7675 ppm
TD	:	65536
TE	:	296.8 K

*** Processing Parameters ***

LB	:	1.00 Hz
SF	:	125.7577925 MHz

*** 1D NMR Plot Parameters ***

NUCLEUS	:	off

1H AMX500

wtl-869 (2)

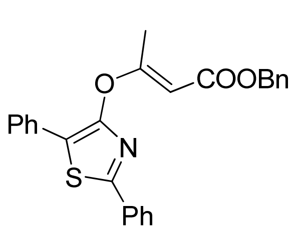

11

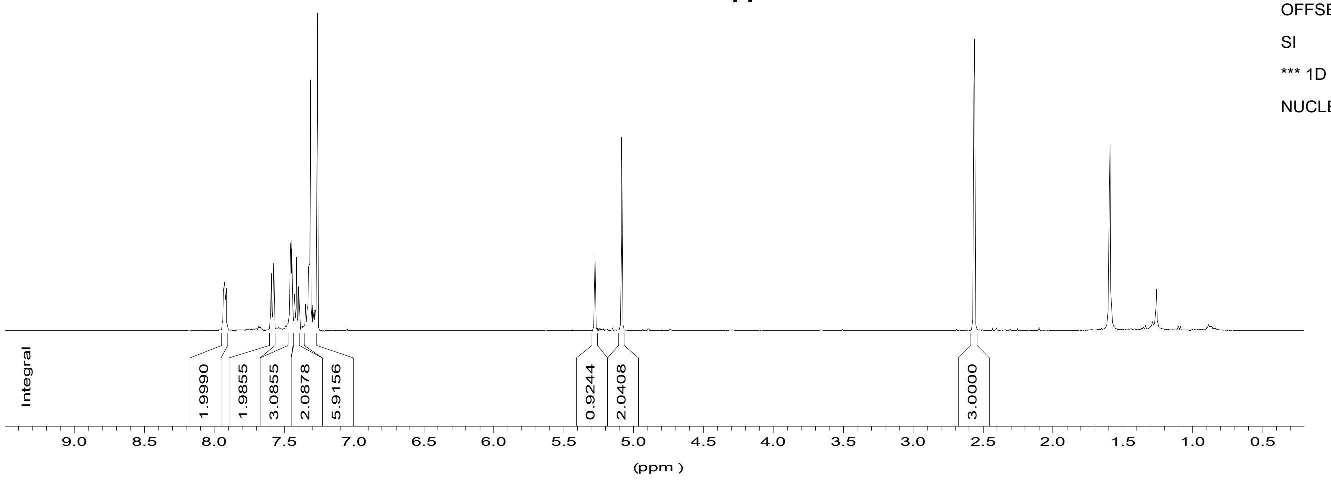

*** Current Data Parameters ***

NAME : wtl-0909

EXPNO : 20

PROCNO : 1

*** Acquisition Parameters ***

DS : 0

INSTRUM : spect

LOCNUC : 2H

NS : 30

NUCLEUS : off

O1 : 3088.51 Hz

PULPROG : zg30

SFO1 : 500.1330885 MHz

SOLVENT : CDCl3

SW : 20.6557 ppm

TD : 32768

TE : 297.2 K

*** Processing Parameters ***

LB : 0.30 Hz

OFFSET : 16.477 ppm

SI : 16384

*** 1D NMR Plot Parameters ***

NUCLEUS : off

13C AMX500

wtl-869 (2)

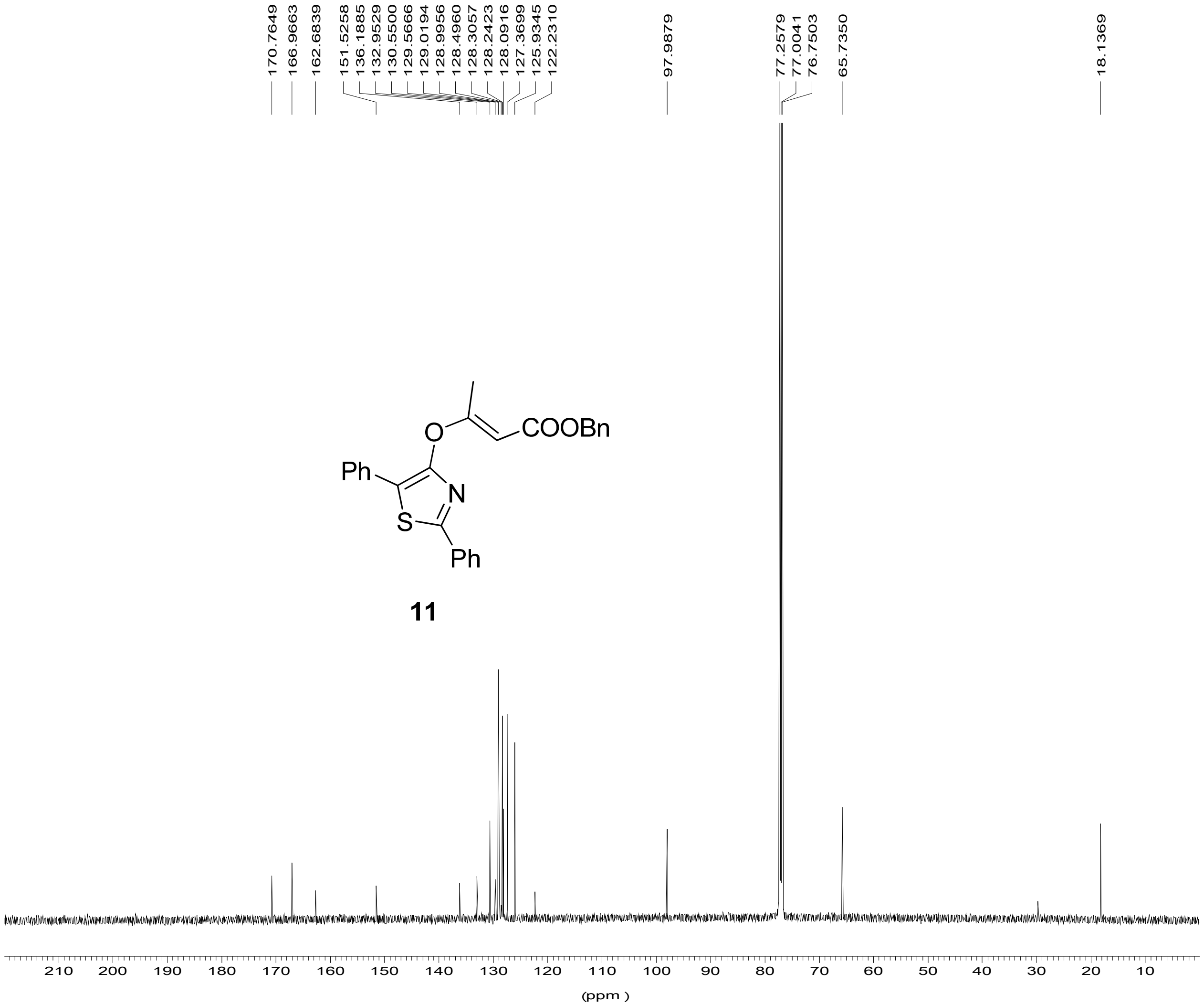

11

*** Current Data Parameters ***

NAME	:	wtl-0909
EXPNO	:	21
PROCNO	:	1

*** Acquisition Parameters ***

DS	:	0
INSTRUM	:	spect
LOCNUC	:	2H
NS	:	14691
NUCLEUS	:	off
O1	:	15090.93 Hz
PULPROG	:	zgpg30
SFO1	:	125.7728799 MHz
SOLVENT	:	CDCl3
SW	:	259.8314 ppm
TD	:	65536
TE	:	297.6 K

*** Processing Parameters ***

LB	:	1.00 Hz
OFFSET	:	249.918 ppm
SI	:	32768

*** 1D NMR Plot Parameters ***

NUCLEUS	:	off

1H AMX500

wtl-1012

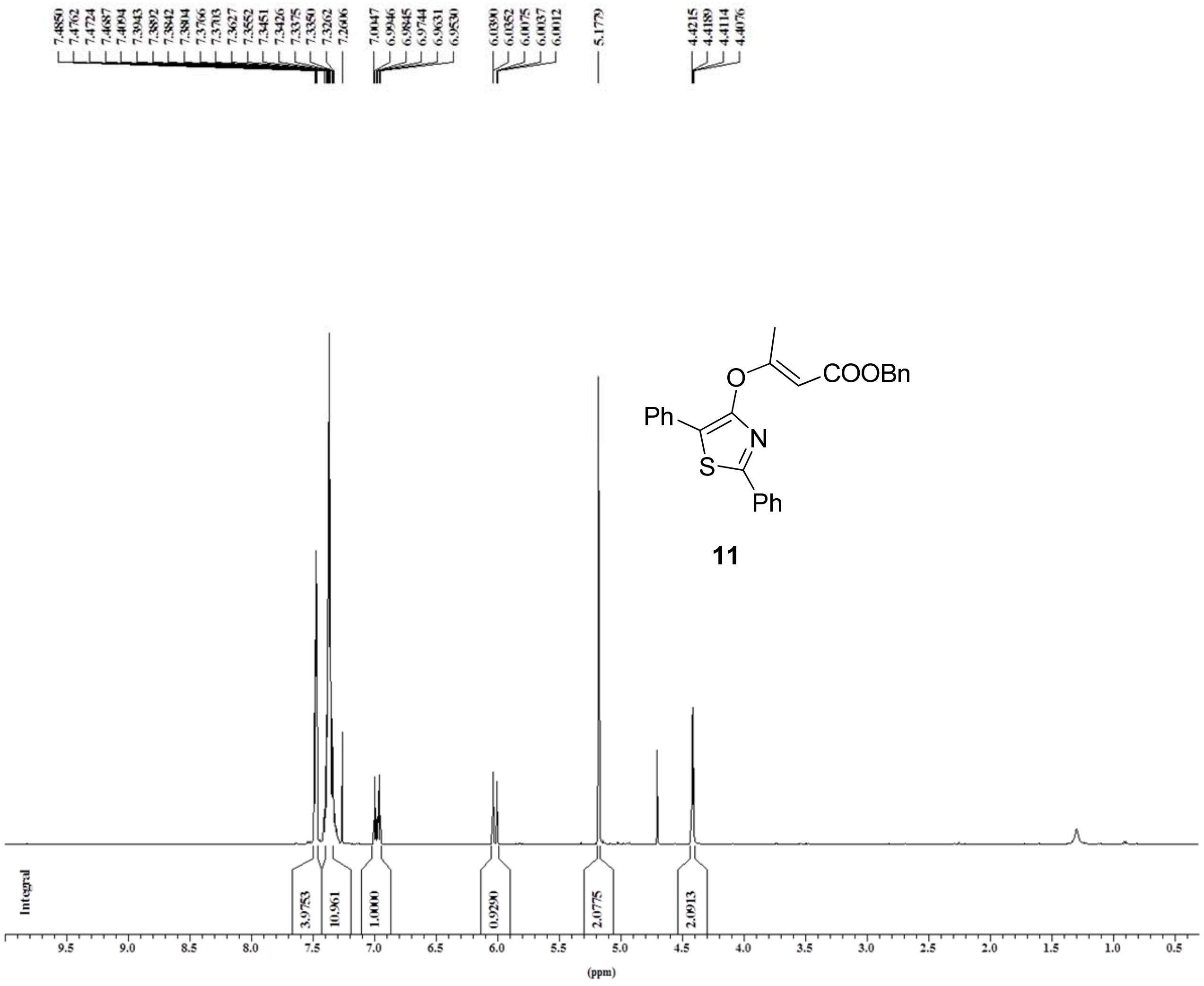

11

*** Current Data Parameters ***		
NAME	:	wtl-0904
EXPNO	:	9
PROCNO	:	1
*** Acquisition Parameters ***		
LOCNUC	:	2H
NS	:	35
NUCLEUS	:	off
O1	:	3088.51 Hz
PULPROG	:	zg30
SFO1	:	500.1330885 MHz
SOLVENT	:	CDCl3
SW	:	20.6557 ppm
TD	:	32768
TE	:	297.6 K
*** Processing Parameters ***		
LB	:	0.30 Hz
SF	:	500.1300134 MHz
*** 1D NMR Plot Parameters ***		
NUCLEUS	:	off

13C AMX500

wtl-1012

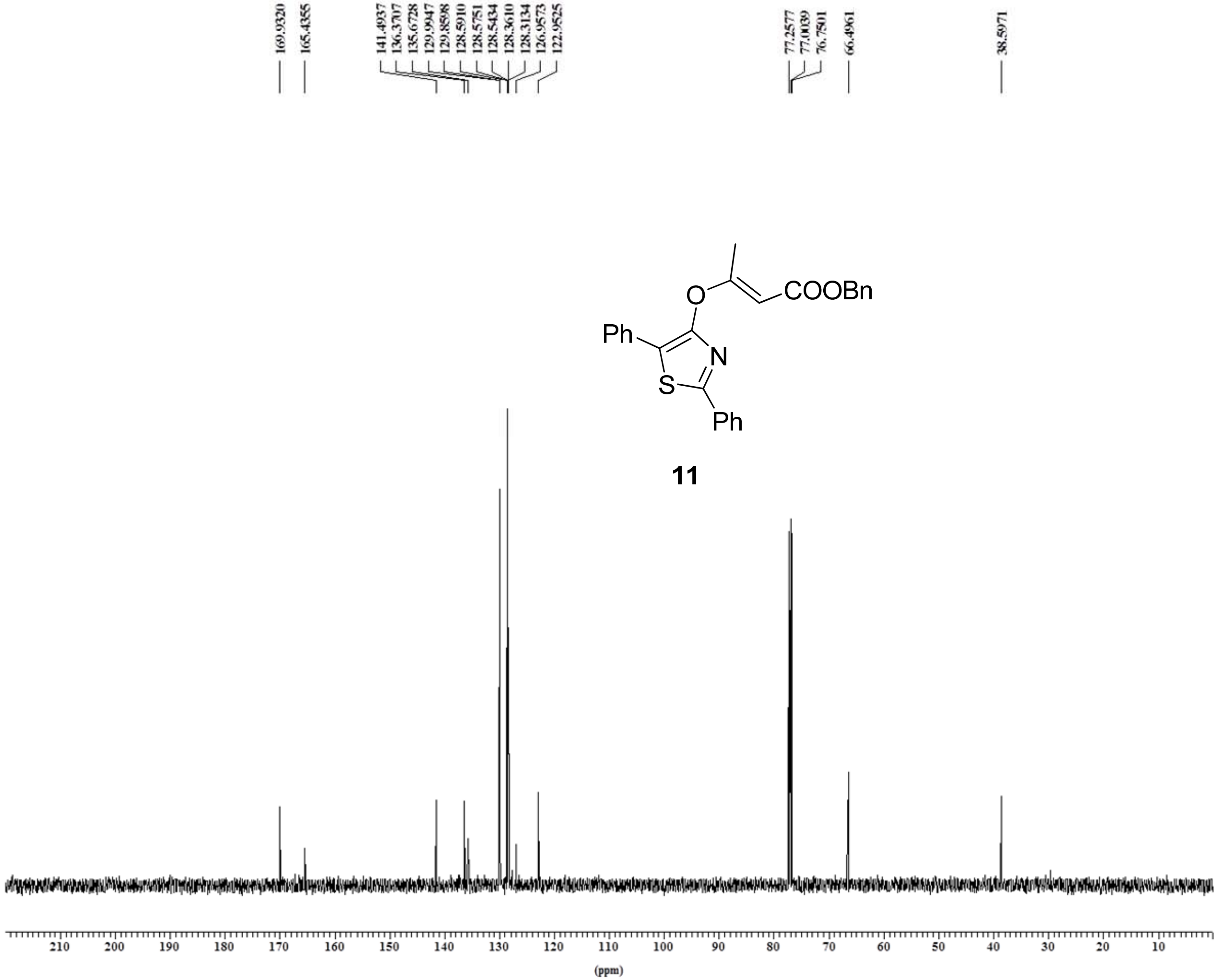

11

*** Current Data Parameters ***		
NAME	:	wtl-0904
EXPNO	:	11
PROCNO	:	1
*** Acquisition Parameters ***		
LOCNUC	:	2H
NS	:	76
NUCLEUS	:	off
O1	:	15090.93 Hz
PULPROG	:	zgpg30
SFO1	:	125.7728799 MHz
SOLVENT	:	CDCl3
SW	:	259.8314 ppm
TD	:	65536
TE	:	298.7 K
*** Processing Parameters ***		
LB	:	1.00 Hz
SF	:	125.7577937 MHz
*** 1D NMR Plot Parameters ***		
NUCLEUS	:	off

1H AMX500

wtl-985

***** Current Data Parameters *****

NAME	:	wtl-0717
EXPNO	:	3
PROCNO	:	1

***** Acquisition Parameters *****

LOCNUC	:	2H
NS	:	26
NUCLEUS	:	off
O1	:	3088.51 Hz
PULPROG	:	zg30
SFO1	:	500.1330885 MHz
SOLVENT	:	CDCl3
SW	:	20.6557 ppm
TD	:	32768
TE	:	297.3 K

***** Processing Parameters *****

LB	:	0.30 Hz
SF	:	500.1300134 MHz

***** 1D NMR Plot Parameters *****

NUCLEUS	:	off

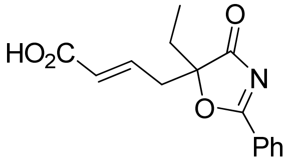

14

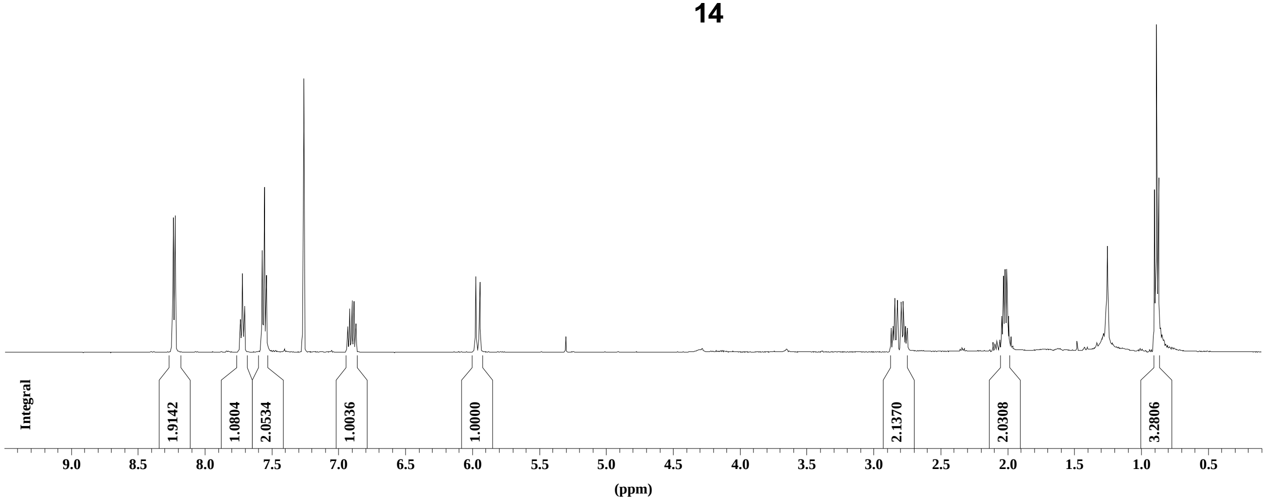

13C AMX500

wtl-985

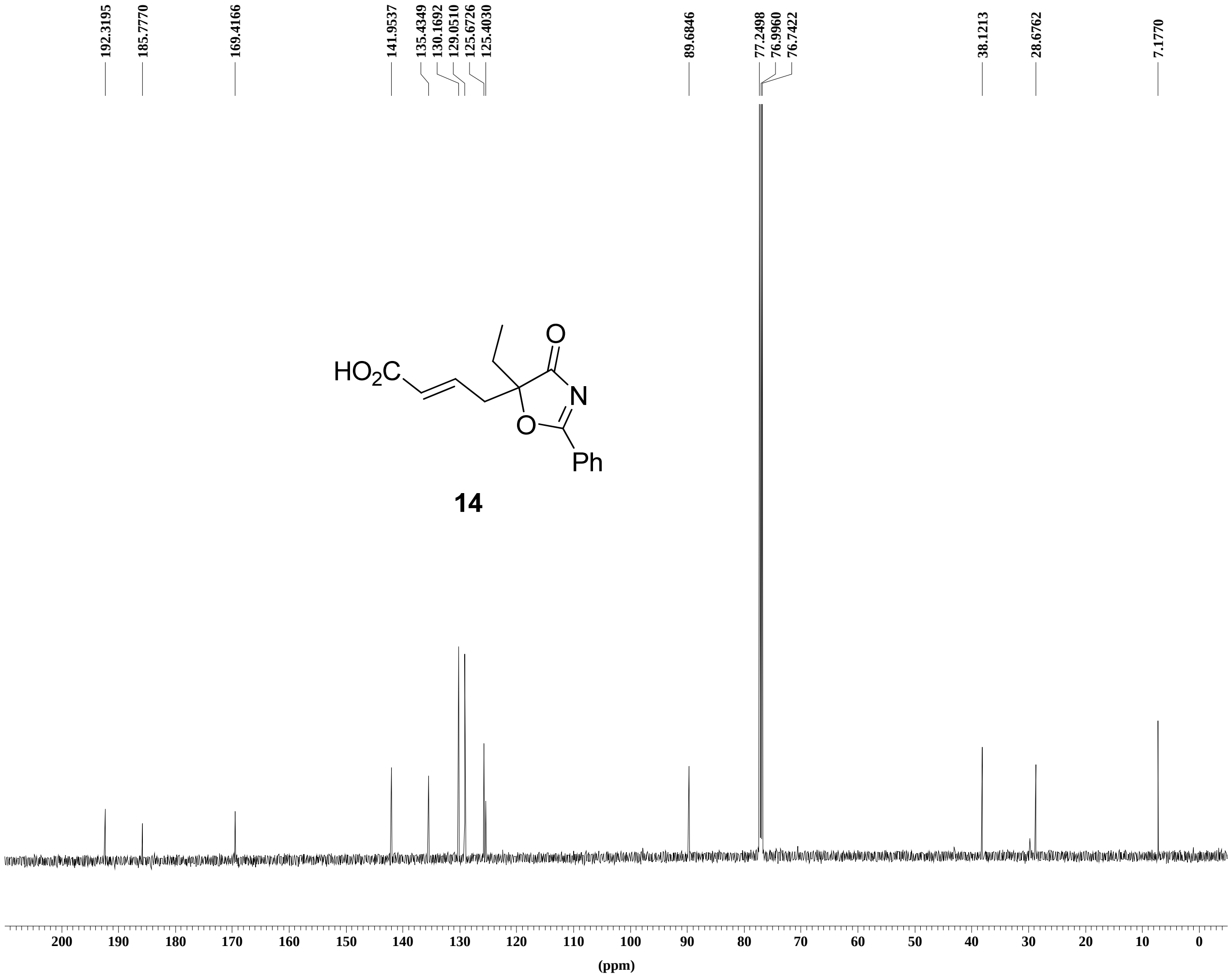

*** Current Data Parameters ***

NAME	:	wtl-0717
EXPNO	:	4
PROCNO	:	1

*** Acquisition Parameters ***

LOCNUC	:	2H
NS	:	2439
NUCLEUS	:	off
O1	:	15090.93 Hz
PULPROG	:	zgpg30
SFO1	:	125.7728799 MHz
SOLVENT	:	CDCl3
SW	:	259.8314 ppm
TD	:	65536
TE	:	297.3 K

*** Processing Parameters ***

LB	:	1.00 Hz
SF	:	125.7577917 MHz

*** 1D NMR Plot Parameters ***

NUCLEUS	:	off

1H AMX500

wtl-0828-2

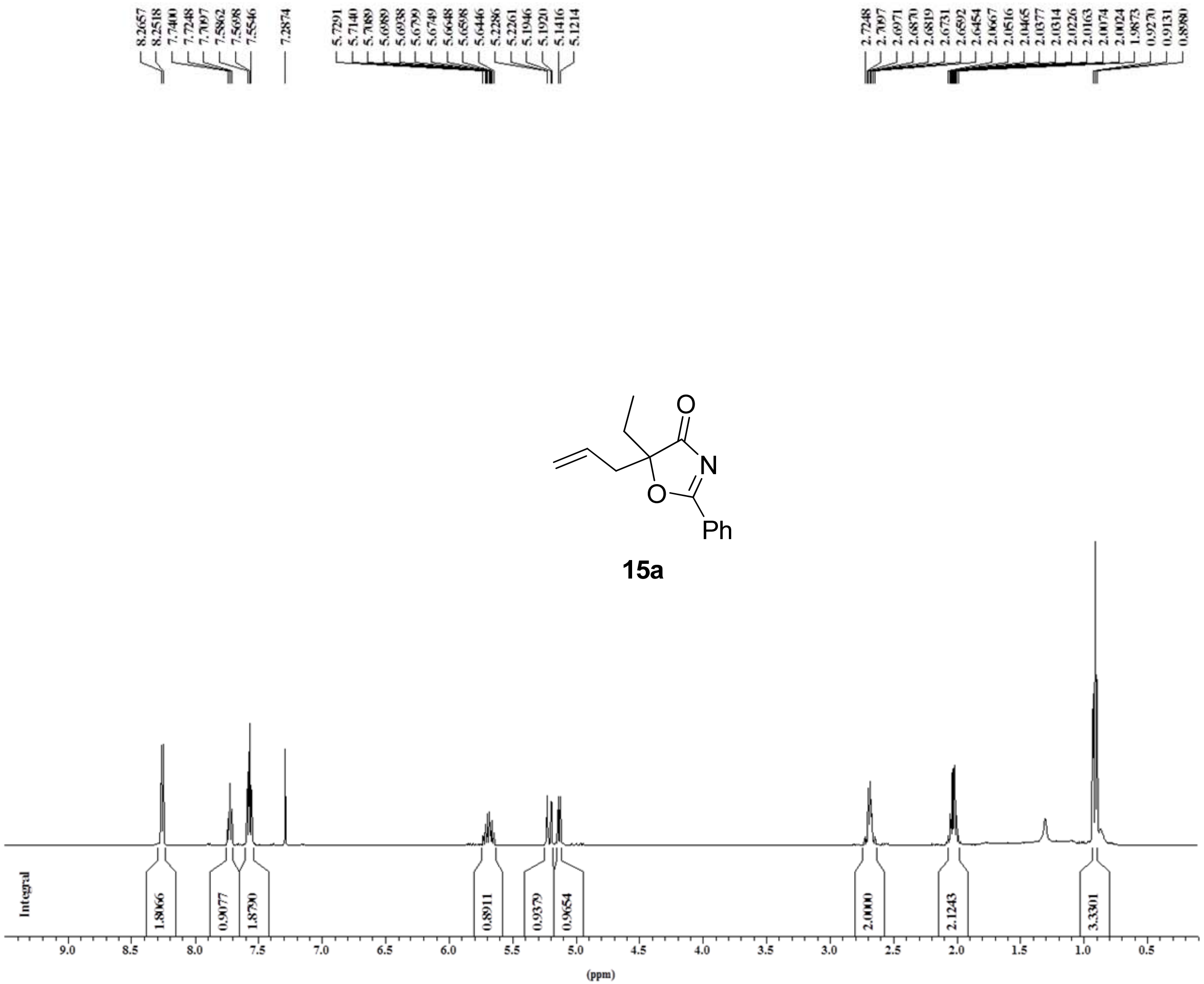

15a

*** Current Data Parameters ***

NAME	:	wtl-0828
EXPNO	:	1
PROCNO	:	1

*** Acquisition Parameters ***

LOCNUC	:	2H
NS	:	39
NUCLEUS	:	off
O1	:	3088.51 Hz
PULPROG	:	zg30
SFO1	:	500.1330885 MHz
SOLVENT	:	CDCl3
SW	:	20.6557 ppm
TD	:	32768
TE	:	298.0 K

*** Processing Parameters ***

LB	:	0.30 Hz
SF	:	500.1300000 MHz

*** 1D NMR Plot Parameters ***

NUCLEUS	:	off

13C AMX500

wtl-0828(2)

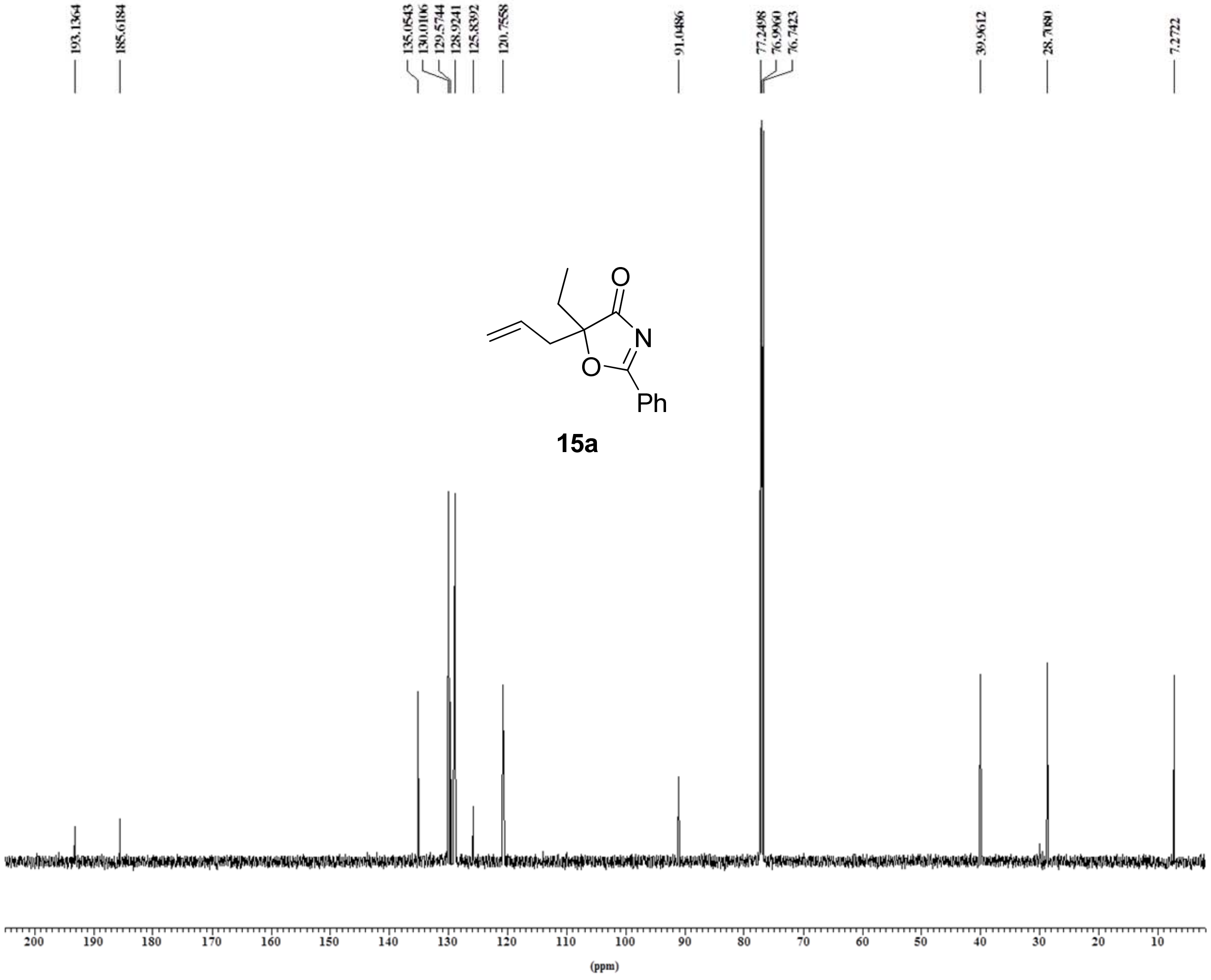

*** Current Data Parameters ***

NAME	:	wtl-0828
EXPNO	:	11
PROCNO	:	1

*** Acquisition Parameters ***

LOCNUC	:	2H
NS	:	588
NUCLEUS	:	off
O1	:	15090.93 Hz
PULPROG	:	zgpg30
SFO1	:	125.7728799 MHz
SOLVENT	:	CDCl3
SW	:	259.8314 ppm
TD	:	65536
TE	:	297.3 K

*** Processing Parameters ***

LB	:	1.00 Hz
SF	:	125.7577927 MHz

*** 1D NMR Plot Parameters ***

NUCLEUS	:	off

1H AMX500

wtl-986-b

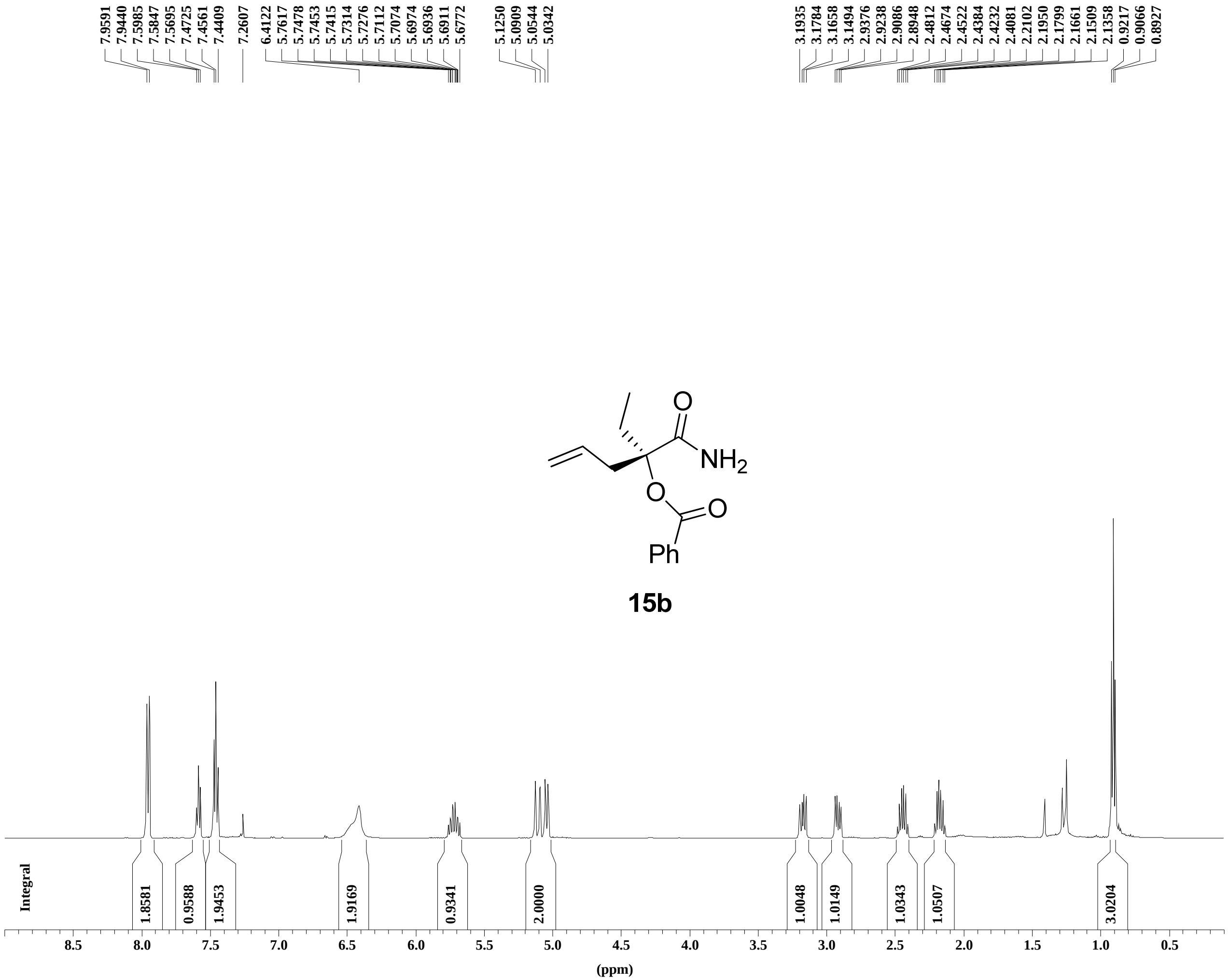

15b

***** Current Data Parameters *****

NAME	:	wtl-0721
EXPNO	:	2
PROCNO	:	1

***** Acquisition Parameters *****

LOCNUC	:	2H
NS	:	22
NUCLEUS	:	off
O1	:	3088.51 Hz
PULPROG	:	zg30
SFO1	:	500.1330885 MHz
SOLVENT	:	CDCl3
SW	:	20.6557 ppm
TD	:	32768
TE	:	297.7 K

***** Processing Parameters *****

LB	:	0.30 Hz
SF	:	500.1300134 MHz

***** 1D NMR Plot Parameters *****

NUCLEUS	:	off

13C AMX500

wtl-986-b

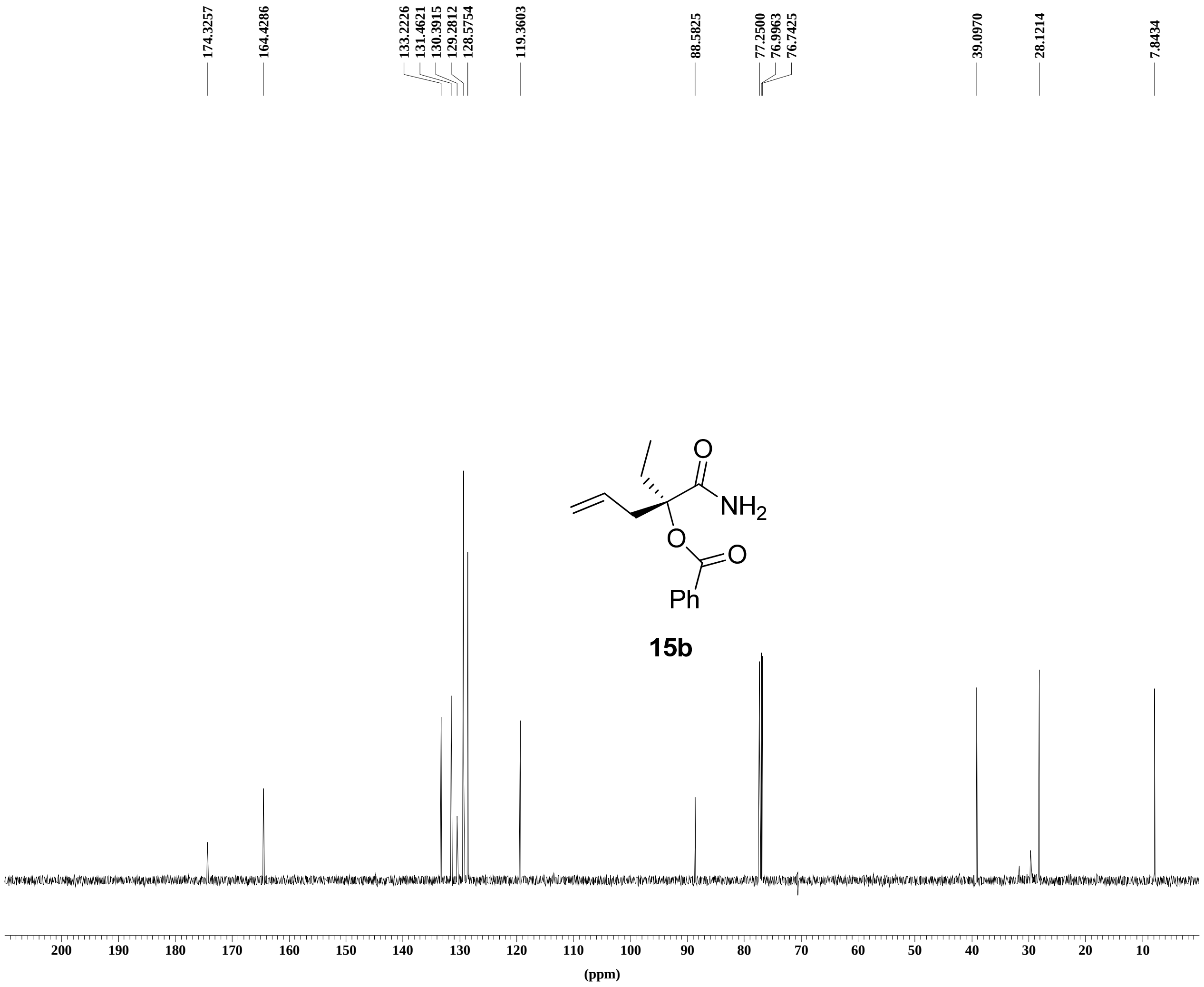

15b

*** Current Data Parameters ***

NAME	:	wtl-0721
EXPNO	:	3
PROCNO	:	1

*** Acquisition Parameters ***

LOCNUC	:	2H
NS	:	79
NUCLEUS	:	off
O1	:	15090.93 Hz
PULPROG	:	zgpg30
SFO1	:	125.7728799 MHz
SOLVENT	:	CDCl3
SW	:	259.8314 ppm
TD	:	65536
TE	:	297.7 K

*** Processing Parameters ***

LB	:	1.00 Hz
SF	:	125.7577966 MHz

*** 1D NMR Plot Parameters ***

NUCLEUS	:	off

1H normal range AC300

wtl-987-1

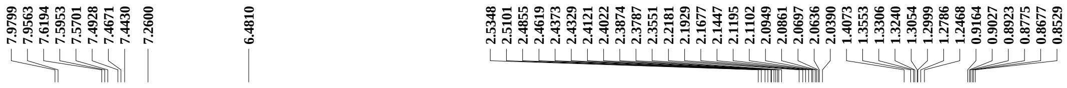
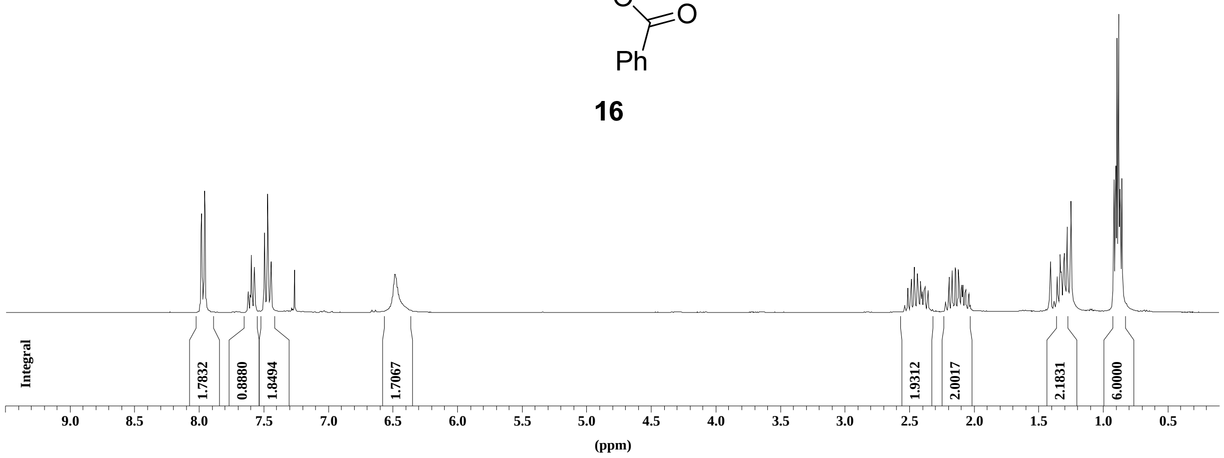

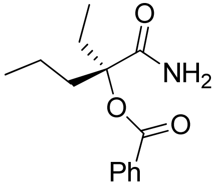

16

***** Current Data Parameters *****

NAME	:	jl21-wtl
EXPNO	:	1
PROCNO	:	1

***** Acquisition Parameters *****

LOCNUC	:	2H
NS	:	19
NUCLEUS	:	off
O1	:	1853.43 Hz
PULPROG	:	zg30
SFO1	:	300.1318534 MHz
SOLVENT	:	CDCl3
SW	:	17.9519 ppm
TD	:	32768
TE	:	298.6 K

***** Processing Parameters *****

LB	:	0.30 Hz
SF	:	300.1300124 MHz

***** 1D NMR Plot Parameters *****

NUCLEUS	:	off

13C Standard AC300

wtl-987-1

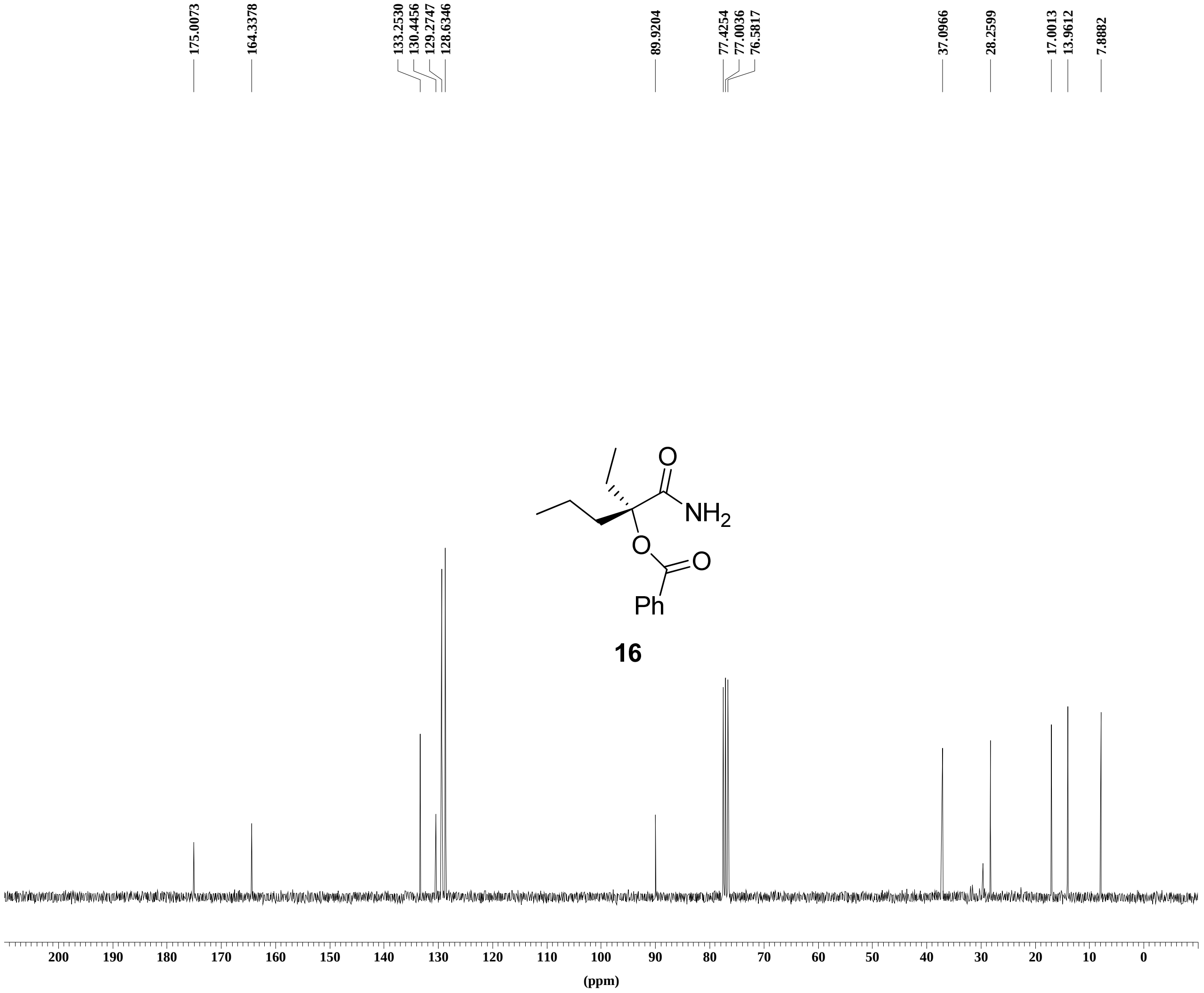

16

*** Current Data Parameters ***

NAME	:	jl21-wtl
EXPNO	:	2
PROCNO	:	1

*** Acquisition Parameters ***

LOCNUC	:	2H
NS	:	375
NUCLEUS	:	off
O1	:	7924.11 Hz
PULPROG	:	zgpg30
SFO1	:	75.4756731 MHz
SOLVENT	:	CDCl3
SW	:	238.2968 ppm
TD	:	32768
TE	:	298.6 K

*** Processing Parameters ***

LB	:	1.00 Hz
SF	:	75.4677536 MHz

*** 1D NMR Plot Parameters ***

NUCLEUS	:	off

1H AMX500

wtl-987-2

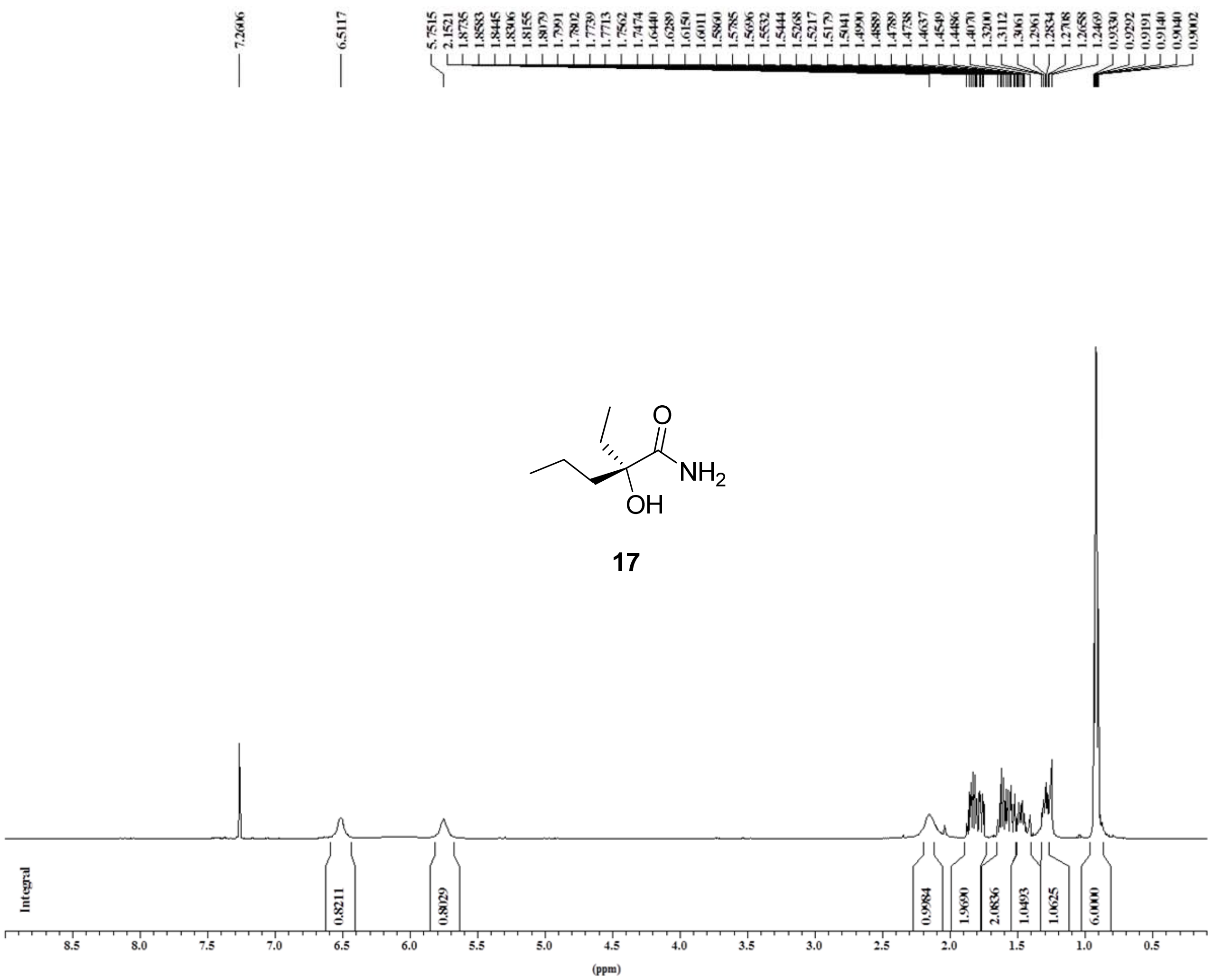

*** Current Data Parameters ***

NAME	:	wtl-0722
EXPNO	:	10
PROCNO	:	1

*** Acquisition Parameters ***

LOCNUC	:	2H
NS	:	47
NUCLEUS	:	off
O1	:	3088.51 Hz
PULPROG	:	zg30
SFO1	:	500.1330885 MHz
SOLVENT	:	CDCl3
SW	:	20.6557 ppm
TD	:	32768
TE	:	297.2 K

*** Processing Parameters ***

LB	:	0.30 Hz
SF	:	500.1300134 MHz

*** 1D NMR Plot Parameters ***

NUCLEUS	:	off

13C AMX500

wtl-987-2

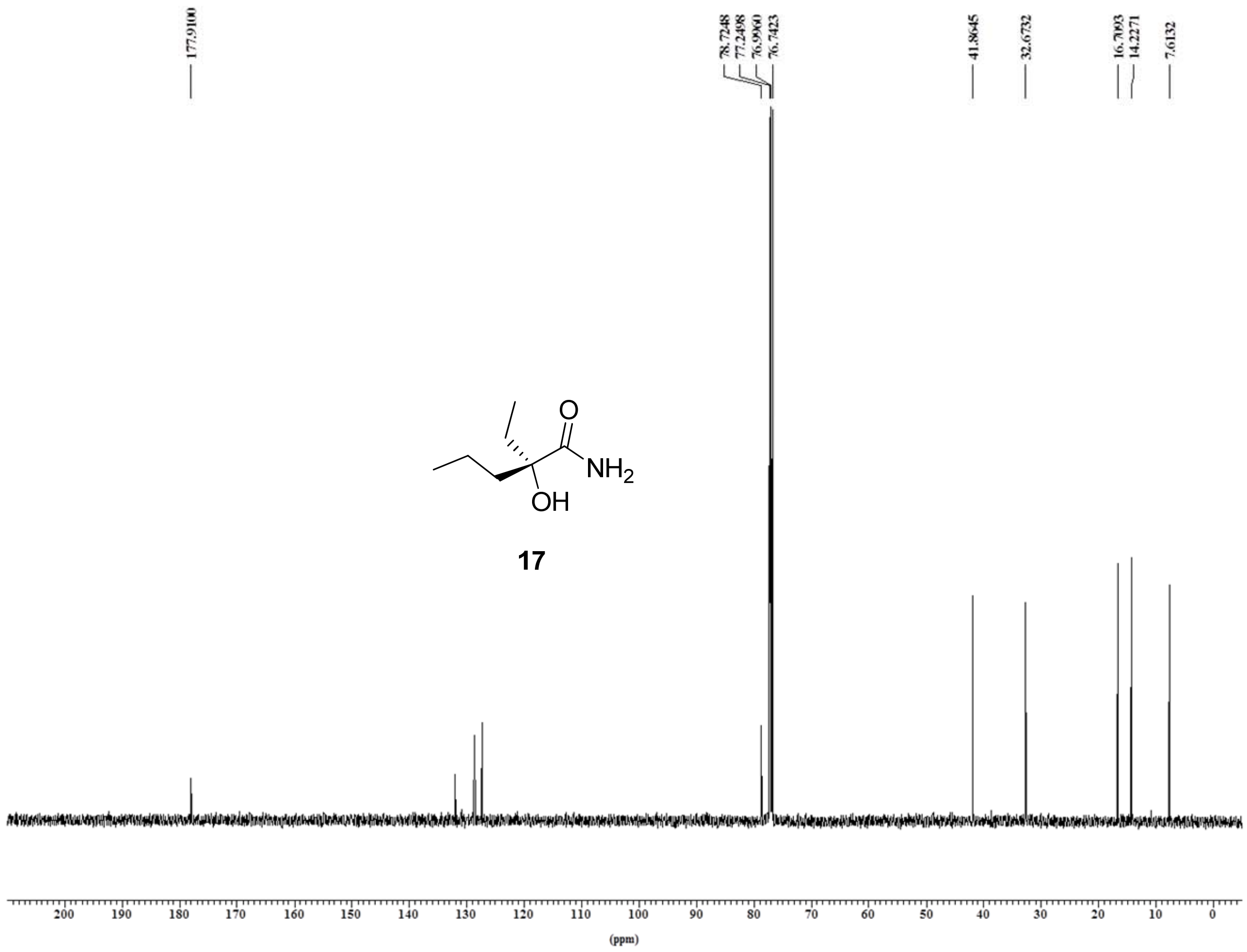

17

*** Current Data Parameters ***

NAME	:	wtl-0722
EXPNO	:	11
PROCNO	:	1

*** Acquisition Parameters ***

LOCNUC	:	2H
NS	:	423
NUCLEUS	:	off
O1	:	15090.93 Hz
PULPROG	:	zgpg30
SFO1	:	125.7728799 MHz
SOLVENT	:	CDCl3
SW	:	259.8314 ppm
TD	:	65536
TE	:	297.4 K

*** Processing Parameters ***

LB	:	1.00 Hz
SF	:	125.7577927 MHz

*** 1D NMR Plot Parameters ***

NUCLEUS	:	off

1H AMX500

wtl-0729-1(b)

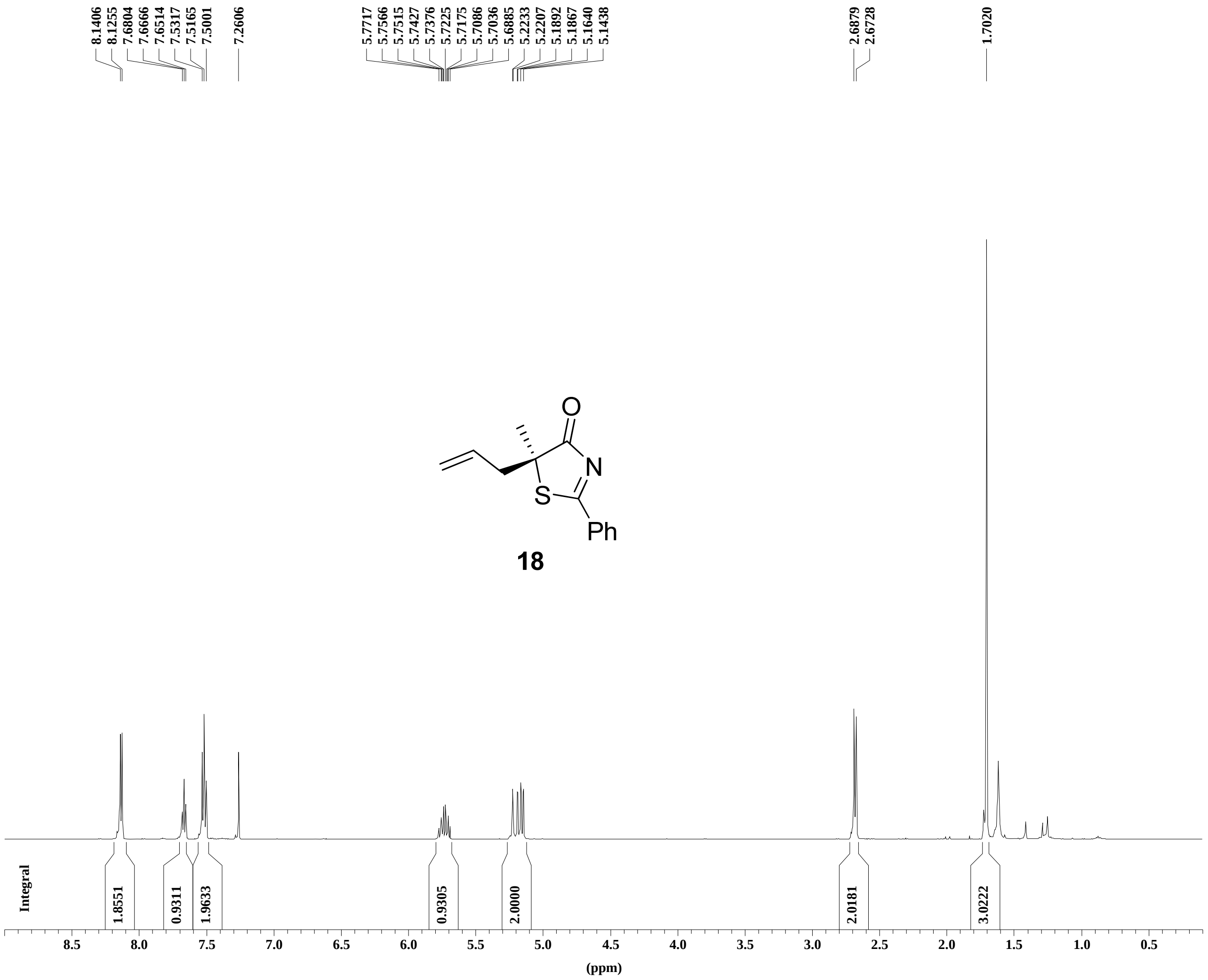

***** Current Data Parameters *****

NAME	:	wtl-0730
EXPNO	:	1
PROCNO	:	1

***** Acquisition Parameters *****

LOCNUC	:	2H
NS	:	23
NUCLEUS	:	off
O1	:	3088.51 Hz
PULPROG	:	zg30
SFO1	:	500.1330885 MHz
SOLVENT	:	CDCl3
SW	:	20.6557 ppm
TD	:	32768
TE	:	297.7 K

***** Processing Parameters *****

LB	:	0.30 Hz
SF	:	500.1300134 MHz

***** 1D NMR Plot Parameters *****

NUCLEUS	:	off

13C AMX500

wtl-0729-1(b)

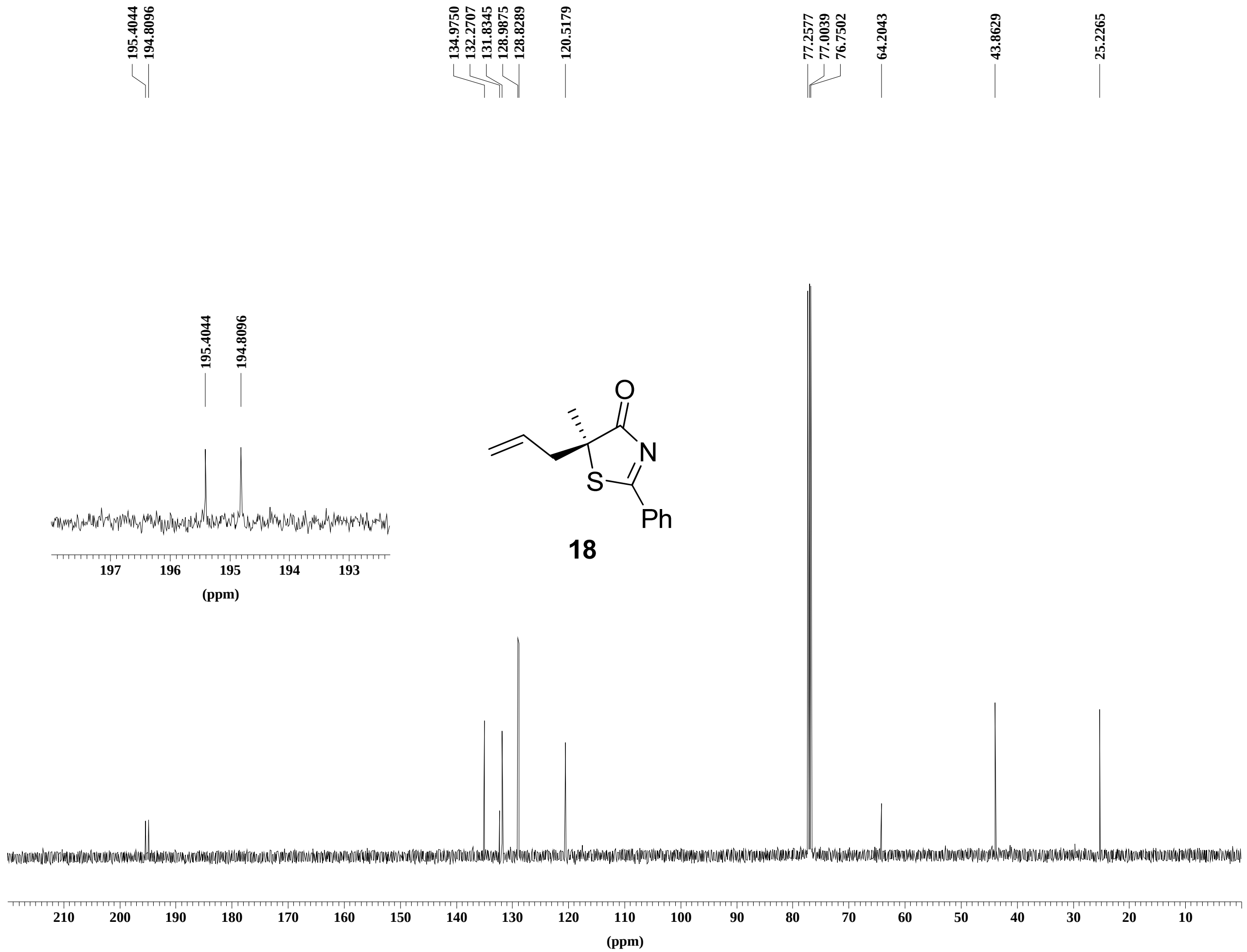

18

*** Current Data Parameters ***		
NAME	:	wtl-0730
EXPNO	:	2
PROCNO	:	1
*** Acquisition Parameters ***		
LOCNUC	:	2H
NS	:	233
NUCLEUS	:	off
O1	:	15090.93 Hz
PULPROG	:	zgpg30
SFO1	:	125.7728799 MHz
SOLVENT	:	CDCl3
SW	:	259.8314 ppm
TD	:	65536
TE	:	298.4 K
*** Processing Parameters ***		
LB	:	1.00 Hz
SF	:	125.7577917 MHz
*** 1D NMR Plot Parameters ***		
NUCLEUS	:	off

1H AMX500

wtl-0904-1b

*** Current Data Parameters ***

NAME	:	wtl-0904
EXPNO	:	3
PROCNO	:	1

*** Acquisition Parameters ***

LOCNUC	:	2H
NS	:	22
NUCLEUS	:	off
O1	:	3088.51 Hz
PULPROG	:	zg30
SFO1	:	500.1330885 MHz
SOLVENT	:	CDCl3
SW	:	20.6557 ppm
TD	:	32768
TE	:	297.8 K

*** Processing Parameters ***

LB	:	0.30 Hz
SF	:	500.1300140 MHz

*** 1D NMR Plot Parameters ***

NUCLEUS	:	off

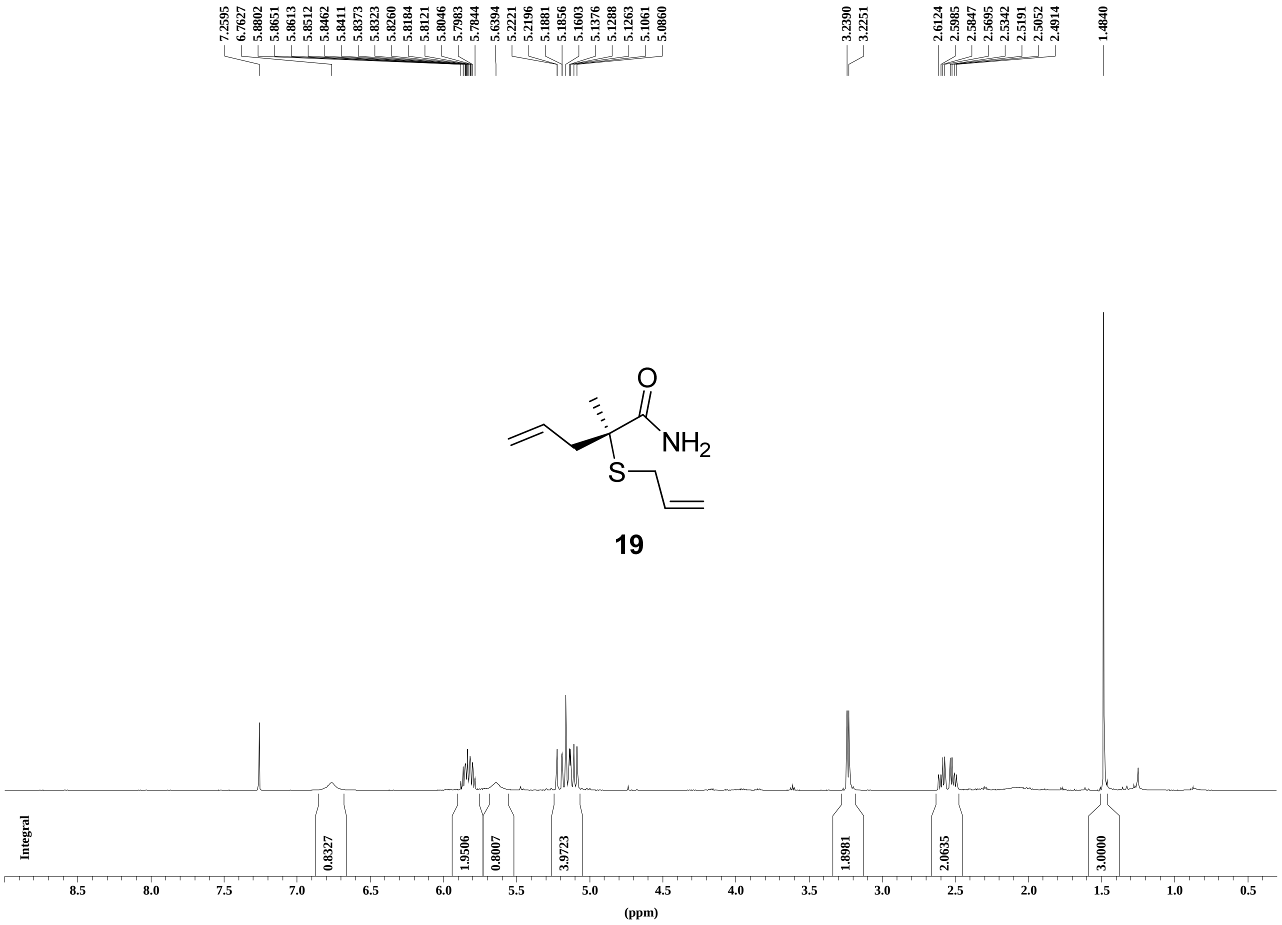

13C AMX500

wtl-0904-1b

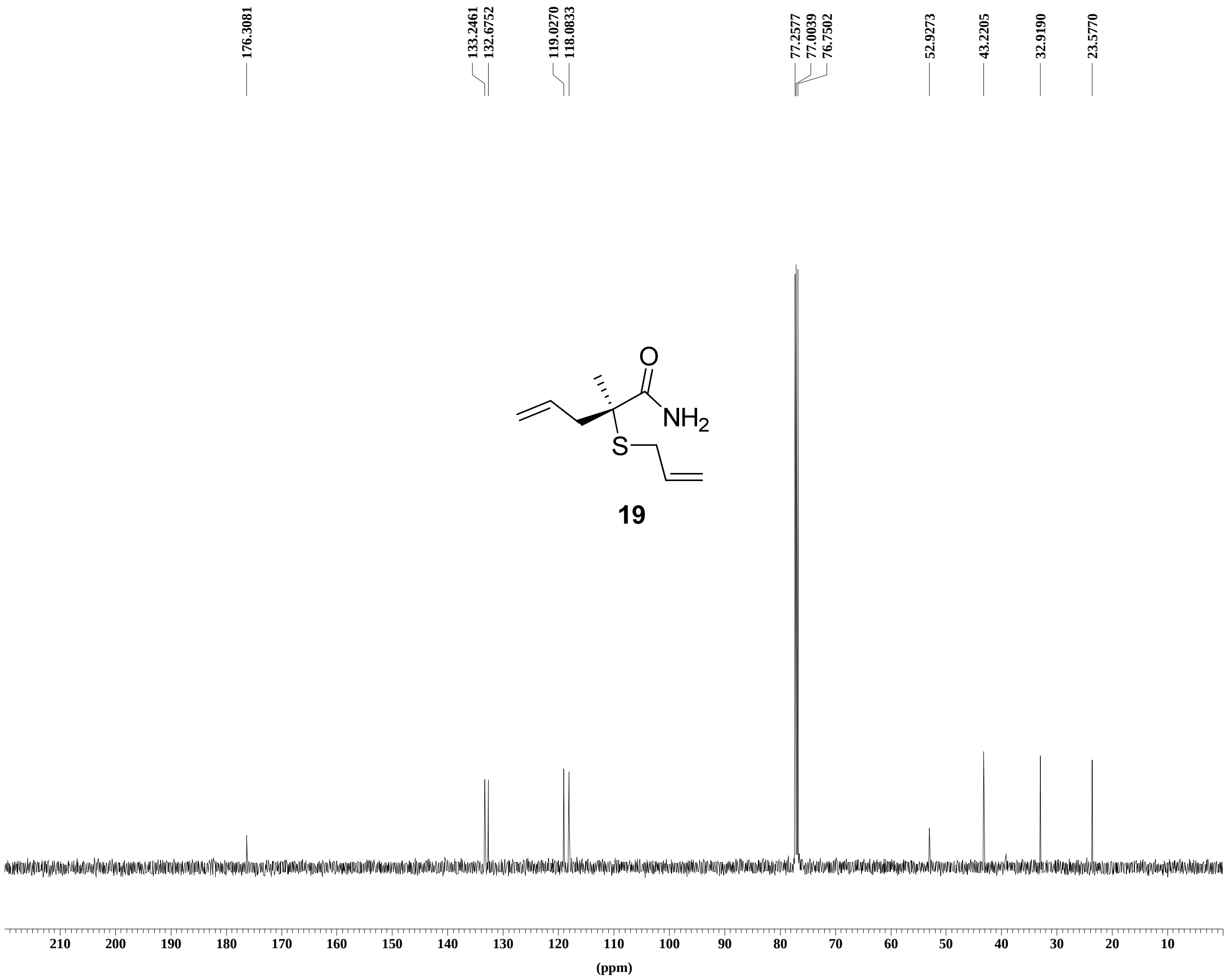

19

*** Current Data Parameters ***

NAME	:	wtl-0904
EXPNO	:	4
PROCNO	:	1

*** Acquisition Parameters ***

LOCNUC	:	2H
NS	:	255
NUCLEUS	:	off
O1	:	15090.93 Hz
PULPROG	:	zgpg30
SFO1	:	125.7728799 MHz
SOLVENT	:	CDCl3
SW	:	259.8314 ppm
TD	:	65536
TE	:	298.3 K

*** Processing Parameters ***

LB	:	1.00 Hz
SF	:	125.7577917 MHz

*** 1D NMR Plot Parameters ***

NUCLEUS	:	off
